# The effects of testing the relationships among relational concepts

**DOI:** 10.1186/s41235-022-00398-2

**Published:** 2022-05-31

**Authors:** Daniel Corral, Alice F. Healy, Matt Jones

**Affiliations:** 1 Department of Psychology, Syracuse University, 475 Huntington Hall, Syracuse, NY 13244 USA; 2 Department of Psychology and Neuroscience, University of Colorado Boulder, Muenzinger Building, Boulder, CO 80309-0345 USA

**Keywords:** Relational learning, Concept acquisition, External and internal structure, Transfer of learning, Training specificity

## Abstract

Many concepts are defined by their relationships to one another. However, instructors might teach these concepts individually, neglecting their interconnections. For instance, students learning about statistical power might learn how to define alpha and beta, but not how they are related. We report two experiments that examine whether there is a benefit to training subjects on relations among concepts. In Experiment 1, all subjects studied material on statistical hypothesis testing, half were subsequently quizzed on relationships among these concepts, and the other half were quizzed on their individual definitions; quizzing was used to highlight the information that was being trained in each condition (i.e., relations or definitions). Experiment 2 also included a mixed training condition that quizzed both relations and definitions, and a control condition that only included study. Subjects were then tested on both types of questions and on three conceptually related question types. In Experiment 1, subjects trained on relations performed numerically better on relational test questions than subjects trained on definitions (nonsignificant trend), whereas definitional test questions showed the reverse pattern; no performance differences were found between the groups on the other question types. In Experiment 2, relational training benefitted performance on relational test questions and on some question types that were not quizzed, whereas definitional training only benefited performance on test questions on the trained definitions. In contrast, mixed training did not aid learning above and beyond studying. Relational training thus seems to facilitate transfer of learning, whereas definitional training seems to produce training specificity effects.

**Supplementary Information:**

The online version contains supplementary material available at 10.1186/s41235-022-00398-2.

## Introduction

People are often required to comprehend and reason about the relationships among different ideas and events. Relational reasoning has been posited to underlie many complex tasks and is believed to play a critical role in higher-order cognition (Cattell, 1940; Hofstader, 2001; Hofstadter & Fluid Analogies Research Group, 1995; Holyoak et al., 2001; James, 1890; Penn et al., 2008; Spearman, 1927; Sternberg, 1999; Wertheimer, 1900). This type of reasoning is exemplified in scientific theories and other types of causal explanations, which posit specific relationships among numerous variables. Furthermore, many of the concepts that people encounter on a daily basis are related to one another, and it has been proposed that such concepts derive much of their meaning from these relationships (Field, 1977; Jones & Love, 2007).

The types of relational concepts that people encounter can vary, as we must often learn the internal relationships among a concept’s components (*internal relational structures*), as well as the external relationships among concepts (*external relational structures*). For instance, a student in a statistics course might be asked to learn the concept of a *type 1 error*, which is defined by the relationship between the researcher, the true state of the null hypothesis, and the researcher’s decision to reject the null hypothesis. Specifically, a type 1 error occurs when the researcher rejects the null hypothesis and the null hypothesis is true. (Likewise, a type 2 error occurs when the researcher fails to reject the null hypothesis and the null hypothesis is false.) This description details the internal relational structure of the concept, because it specifies the elements and their relationships that together constitute an instance of a type 1 error. This student might also be required to learn an external relational structure for the concept of type 1 error, meaning relations in which this concept participates. For example, the type 1 error rate (*alpha*) is related to the type 2 error rate (*beta*) and the *critical value* (the cutoff point in the test statistic sampling distribution for rejecting the null hypothesis), such that varying the critical value will have opposite effects on alpha and beta. Specifically, increasing the critical value will decrease the probability of a type 1 error and increase the probability of a type 2 error, whereas the opposite is true if the critical value is decreased. Similarly, in a biology course, students might be asked to learn the definitions (i.e., internal structure) of *random mutations*, *phenotypic* and *genotypic traits*, *environmental* and *selective pressures*, and *mate selection* and how these various concepts are interconnected (i.e., their external structures); in a physics course, students might have to learn the definitions of *force*, *mass*, and *acceleration*, as well as the relationships among these concepts (e.g., Newton’s second law).

As these examples demonstrate, an internal relational structure is defined by the manner in which a concept’s components are bound together by shared relations (Corral & Jones, 2014), whereas an external relational structure is defined by the way in which a concept is related to other concepts. Both types of knowledge are prevalent in STEM-based education (e.g., mathematics, physics, biology), wherein students are required to learn the definitions of many relational concepts (internal structures), as well as how those concepts are interconnected (external structures). Nevertheless, many of these concepts might often be taught individually, especially since the distinction we make between internal and external conceptual structure has not been recognized in the educational literature. For instance, professors teaching about statistical power might provide their students definitions of the concepts of alpha and beta, but might not emphasize how the two are related. As a result, students might learn the definitions for a given set of concepts, but fail to recognize how they are interconnected. Nevertheless, applying scientific concepts usually requires knowledge of their interrelationships. Thus, students are often tested on and expected to know how the concepts within a given conceptual system (i.e., a domain or topic) are related to one another (e.g., the relationships between a type 1 error and a type 2 error).

These types of relational concepts are quite ubiquitous in education (Goldwater & Schalk, 2016). It is therefore fitting that relational reasoning is fundamental to student learning (Alexander, 2016; Dumas et al., 2013; Resnick et al., 2017), particularly in STEM-based fields (Alexander, 2017), such as physics, chemistry, biology, medicine, mathematics, and engineering (Christensen & Schunn, 2007; Dumas, 2017; Dumas et al., 2014, 2016; Dunbar & Fugelsang, 2005; Patel et al., 2012; Pena & de Souza Andrade-Filho, 2010; Richland et al., 2007; Thagard, 1997). For these reasons, there is considerable interest in finding ways to improve relational learning and reasoning in students (Alexander, 2016). Indeed, various studies have found suggestive evidence that emphasizing the relationships between or among concepts can aid student learning (Alexander et al., 1997; Bellocchi & Ritchie, 2011; Braasch & Goldman, 2010; Goswami & Mead, 1992; Jairam & Kiewra, 2010; Mayer, 1996; McDaniel et al., 2013; Scruggs et al., 1994; Titsworth & Kiewra, 2004; Trey & Khan, 2008; Zheng et al., 2008).

Although such findings are certainly promising, this work does not appear to distinguish between concepts that are defined by internal versus external relational structures. Moreover, because much of this work is largely applied and often involves relatively complex learning paradigms consisting of multiple components that differ from those in control groups (e.g., retrieval practice, re-study, spacing, interleaving, feedback, comparison), the mechanisms that underlie improvements in relational learning are somewhat unclear. There are thus basic open questions regarding (a) how emphasizing internal versus external relations might affect relational learning, as well as (b) what mechanisms may drive such learning effects.

Previous work in the cognitive sciences has shown that similarity among relational concepts is determined by the extent to which those concepts share a common internal structure (Markman & Gentner, 1993; Medin et al., 1990). Structure-mapping theory (Gentner, 1983) posits that instances of analogous concepts are recognized by putting the corresponding elements between two scenarios into alignment, thereby allowing for their common structure to be abstracted. For example, consider two scenarios, one in which a rabbit shares a carrot with a boy and another in which a woman gives a small piece of her donut to a pigeon (cf. Markman & Gentner, 1993). Although both scenarios contain different surface features (e.g., rabbit, woman, carrot, etc.), both involve the same internal structure, such that one agent shares food with another agent. According to structure-mapping theory, people can recognize that these two scenarios are similar by aligning their corresponding elements (e.g., rabbit → woman and boy → pigeon), which leads to the abstraction of their common structure (e.g., *cause*[*give*(*agent*_*1*_,*food*), *receive*(*agent*_*2*_,*food*)], an instance of the concept *share*).

Although recent work has examined the learning and recognition of internal structure (e.g., Corral & Jones, 2014; Corral et al., 2018; Goldwater & Gentner, 2015; Goldwater et al., 2018), less is known about how people learn and recognize external relations. One question to consider is whether learning about the relations between or among concepts that participate in a shared external structure is more central to learning and comprehending a conceptual system than is learning about those individual concepts; this latter case involves learning internal structure, which can be thought of as a type of definition for a given concept (e.g., *type 1 error*).

Structure-mapping theory (Gentner, 1983) might inform this question, as it focuses on how humans represent and reason about relational concepts. Although this theory was initially intended to explain how humans represent analogies between scenarios, theoreticians have posited that it directly applies to relational concept acquisition as well (Gentner, 2010; Gentner & Hoyos, 2017; Gentner & Namy, 1999; Gentner et al., 2003; Kuehne et al., 2000; McLure et al., 2010).

Structure-mapping theory’s (Gentner, 1983) *systematicity principle* holds that learners have a preference for seeking out and discovering shared interconnections between relational systems. To exemplify this idea, consider three scenarios, one in which a planet *revolves* around a star (Scenario 1), one in which a satellite *revolves* around a planet (Scenario 2), and one in which a large sphere initially *attracts* a smaller sphere but then *repels* it (Scenario 3). The first two scenarios share three relations that are interconnected by a fourth higher-order relation: In both cases, the *larger* object *attracts* and *causes* the smaller object to *revolve* around it (*cause*[*and*{*larger*(*object*_*1*_,*object*_*2*_)*,attracts*(*object*_*1*_,*object*_*2*_)}, *revolves*(*object*_*2*_,*object*_*1*_)]; Gentner, 1983). In contrast, although the third scenario has two relations (*larger* than and *attracts*) common with the first two scenarios, it shares no interconnected relations with them. The first two scenarios thus share more interconnected relations with one another than they do with the third scenario. For this reason, according to the systematicity principle (Gentner, 1983, 2010; Gentner & Hoyos, 2017; Gentner & Markman, 1997; Markman & Gentner, 2000), learners should consider the first two scenarios to be more similar to each other than either is to the third scenario.

Applying this principle to relational learning leads to the prediction that subjects can better learn about concepts that share external relations (e.g., *alpha* and *beta*) if instruction emphasizes those relations as opposed to focusing on the individual concepts. On this view, emphasizing external relations will highlight the interconnectedness (i.e., systematicity) of the conceptual system, making the entire system easier to learn. Thus, the systematicity principle suggests the intriguing possibility that learners will acquire definitions (i.e., internal structures) of individual concepts better if given additional training on relations among those concepts rather than on the definitions themselves.

Alternatively, it is possible that prematurely learning about the relationships among a set of concepts, before subjects have fully learned those concepts’ definitions or internal structures, can impede learning. One reason for this possibility is that learning individual concepts might be more central to learning a conceptual system than is learning about how those concepts are interconnected, as various individual concepts can be learned on their own without reference to their relationships (e.g., *type 1* and *type 2 errors* both admit isolated definitions). Furthermore, concepts are logically primary to the relations in which they participate: Many or most concepts exist independently of their external relationships, whereas the relations between or among these concepts depend on the existence of the constituent concepts. Thus, learners potentially must learn a conceptual system’s individual concepts before they can learn how those concepts are related. For this reason, it is possible that by learning and comprehending the individual concepts within a given conceptual system, subjects can also discover how those concepts are interconnected. In contrast, this discovery might be hindered if subjects do not have a full grasp of the individual concepts. We elaborate on these hypotheses in the following section, where we report an experiment that investigates these questions.

## Experiment 1

In this experiment, subjects were provided study material on a given set of concepts, some were trained (via quizzes) on the individual concept definitions (internal structure) and others on the relationships among those concepts (external structure), and finally all subjects were tested on both relationships and definitions. Specifically, subjects were asked to study PowerPoint-style slides covering the logic of statistical hypothesis testing, as might be taught in an introductory undergraduate statistics course. These slides covered the following concepts: (a) the null hypothesis, (b) the alternative hypothesis, (c) alpha, (d) beta, (e) critical value, (f) test statistic, (g) type 1 error, and (h) type 2 error.

Statistical hypothesis testing was selected as the topic of study because these concepts share many rich mathematical relationships, such that the value of one concept is often a function of the values of the other concepts. For example, a higher alpha value increases the likelihood of a type 1 error but decreases the likelihood of a type 2 error. After studying, subjects were quizzed either on the concepts’ relationships to one another or on the concepts’ definitions, depending on their experimental condition. After each response, subjects were shown whether they were correct, along with the correct response. Subjects were then allowed to study the material once more. Lastly, all subjects were given a posttest, consisting of new questions that queried both definitional and relational knowledge, to assess how well the study material was learned. All materials (i.e., study slides, relational and definitional training questions, and posttest questions) used in this study are included in the Additional file 1.

Quizzing was used as a tool to examine whether emphasizing the learning of individual concepts versus emphasizing the relationships among those concepts leads to different learning outcomes. Relational and definitional quizzes were expected to make the corresponding information more salient, hence directing subjects’ attention to the relevant information during the second study stage (see McDaniel et al., 2015). One advantage of this approach is that subjects in both conditions were given identical study material, with only their focus of attention or learning strategies potentially differing. In addition, the decision to use quizzes to train subjects on either definitions or relations was motivated by the findings that retrieval practice helps subjects to better learn and retain the material that is retrieved (Carpenter, 2009; Carpenter & Yeung, 2017; Carrier & Pashler, 1992).

Extending the theoretical principles from the Introduction to the present design leads to two contrasting predictions. On the one hand, the relational training condition leverages the preference for systematicity (Gentner, 1983, 2010; Gentner & Hoyos, 2017) by highlighting the interconnections among the concepts that are being learned. One possibility is that thinking about how these concepts are related to one another (relational training) might help subjects think more deeply about the concepts and their corresponding attributes, which might facilitate comprehension of their meaning (i.e., definitions). Thus, one prediction is that subjects who receive relational training will better learn both the relationships that are shared among the to-be-learned concepts and the individual concept definitions, as compared to subjects who are trained on the concepts’ definitions.

On the other hand, an opposing prediction is that definitional training will be superior for learning both definitions and relations, because definitions are arguably more foundational to learning a conceptual system. Moreover, subjects who do not have a grasp of the individual concepts might not benefit from training on the relationships among those concepts.

Critically, both of these predictions are premised on the assumption that subjects can apply and transfer the knowledge they acquire during training to help them learn and comprehend information that is conceptually related to what they have learned, but which has not been explicitly trained. However, this type of discovery amounts to a relatively challenging form of transfer, which is somewhat rare (Barnett & Ceci, 2002; Detterman, 1993). Furthermore, the literature on transfer-appropriate processing (Morris et al., 1977) suggests that learning should be best for the types of concepts that are specifically trained. Emphasizing the concept definitions might therefore help subjects learn the internal structure of those concepts, just as emphasizing the relationships among those concepts might help subjects learn those concepts’ external structure. Thus, a third prediction (opposing both of the earlier two) is that subjects will perform better on test questions that correspond to the type of knowledge that was emphasized during training, such that definitional training will lead to better performance on definitional test questions and relational training will lead to better performance on relational test questions. The present experiment was designed to test among these three hypotheses and thus to provide insights into the comparative benefits of definition- versus relation-focused concept learning.

### Method

#### Participants

One hundred ninety undergraduate students from the University of Colorado Boulder participated in this study for course credit in an introductory psychology course. Our strategy for data collection was to run as many subjects as we could recruit during the semester in which this experiment was conducted. Once the semester was over, the minimum criterion for stopping data collection was that there were enough subjects, based on an a priori power analysis, for overall posttest differences between the training conditions, with approximately 90% power to detect a medium effect size (*f* = 0.25; *alpha* = 05).

The student population from which subjects were sampled consisted primarily of freshmen. Approximately 46% of students at this university identify as female and 68% of students are White. Approximately 66% of students are 21 years of age or younger; 16% of students from this university are classified as low-income students. This study was approved by the institutional review board at the University of Colorado Boulder.

#### Design and materials

**Fig. 1:**
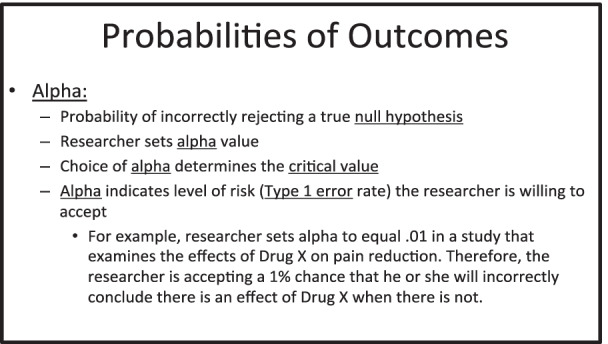
Example training slide from Experiments 1 and 2

Subjects were randomly assigned to two conditions: relational training and definitional training. All stimuli were presented on an LCD computer monitor at the center of the screen on a black background. All responses were entered using a standard computer keyboard. The training slides that subjects were presented were adapted directly from lecture material from an undergraduate statistics course taught by one of the authors (MJ). These slides were designed to be concise and were thus devoid of unnecessary information; this format was adopted from Corral et al. (2019). These slides covered all of the relations among concepts and their definitions that were tested in the quizzes and at posttest. Figure 1 shows a training slide from the study. Under each slide, a counter was presented that indicated which slide out of the total number of slides the subject was viewing (e.g., Slide 4/15).

Subjects were randomly assigned to two training conditions that differed in the quiz questions they were given after the training slides: relational training (*N* = 96) and definitional training (*N* = 94). Quizzes in the relational training condition tested subjects on the relationships among concepts; quizzes in the definitional training condition tested subjects on the individual concept definitions. Both types of quiz questions were designed to exclude extraneous information. All quiz and posttest questions were presented in multiple-choice format with four response options (labeled a–d).

**Fig. 2:**
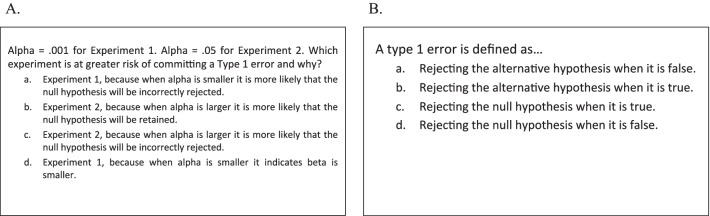
Example **A** relational and **B** definitional quiz questions from Experiments 1 and 2. In both examples, the correct response is option *c*

Relational quiz questions were presented as hypothetical scenarios that were described abstractly. Figure 2a shows a question from the relational training condition. Each of these questions tested a single relationship between two concepts and asked subjects to determine how a change in the value of one concept affects the value of the other. Six of these relations were tested during training: (a) alpha and type 1 error rate, (b) beta and type 2 error rate, (c) test statistic and support for the null hypothesis, (d) alpha and the probability of finding support for the alternative hypothesis, (e) alpha and the critical value, and (f) beta and the probability of rejecting the null hypothesis. For definitional questions, subjects were shown a single concept and asked to select the correct definition. Figure 2b shows a question from the definitional quiz condition. Six concept definitions were tested during training: (a) alpha, (b) beta, (c) critical value, (d) test statistic, (e) type 1 error, and (f) type 2 error.

**Fig. 3:**
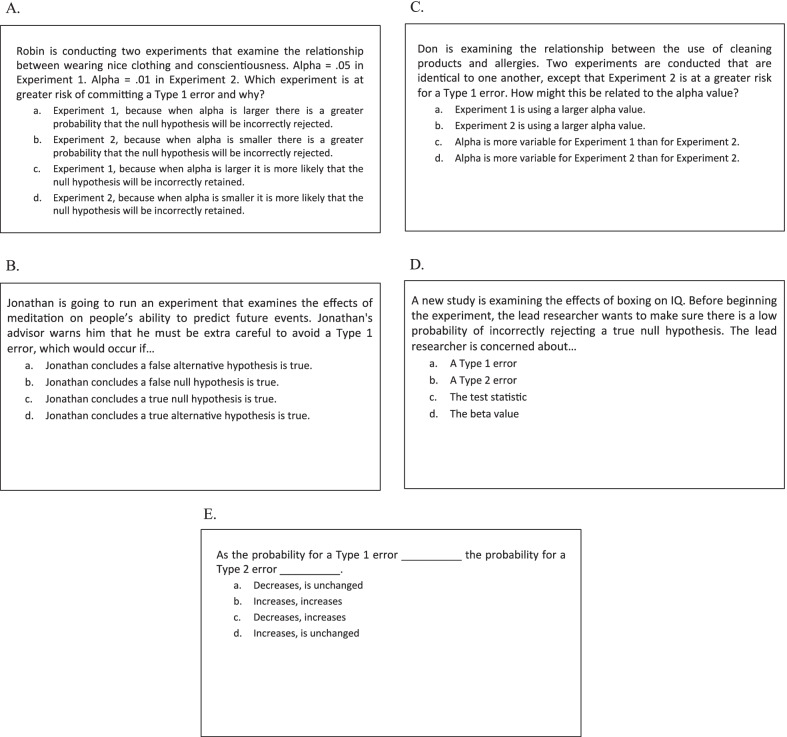
One example question from each question type from the posttest in Experiments 1 and 2. **A** Relational question (correct answer = *a*). **B** Definitional question (correct answer = *a*). **C** Inverse relational question (correct answer = *b*). **D** Inverse definitional question (correct answer = *a*). **E** Novel relations question (correct answer = *c*)

Posttest questions consisted of five question types: (a) relational, (b) definitional, (c) inverse relational, (d) inverse definitional, and (e) novel relations; question types a–d were grounded in concrete scenarios and the relations questions were described more abstractly (see Fig. 3E). There were six questions of each question type. The two primary question types of interest were the definitional and relational questions. Both of these question types tested the same concepts and relationships that were covered during training, but were presented in new scenarios. Additional steps were taken to avoid subjects’ using rote memory for the correct answer choices from the quiz and applying those answers to the corresponding posttest questions. For definitional questions, the correct answer choice for each concept that was quizzed was reworded at posttest, so as to provide a logical instantiation of the concept using different terminology (compare Figs. 2b and 3b). For relational questions, the correct response varied from quiz to posttest and depended on how the relationship was structured between the two concepts in the posttest scenarios (compare Figs. 2a and 3a).

The other three question types were included to assess how well subjects could transfer and apply their knowledge to unfamiliar question types. Each inverse relational question tested the opposite relation between the concepts that was tested during training. For example, if a relational quiz question asked how Concept A affects Concept B, its inverse would ask how Concept B affects Concept A. Each inverse definitional question provided the definition of a concept and subjects were asked to select the corresponding term. Lastly, the questions on novel relations tested relationships between pairs of concepts that were not quizzed (but were covered in the study slides). This question type tested relations among the following pairs of concepts: (a) type 1 and type 2 error rates, (b) type 2 error rate and alpha, (c) test statistic and the alternative hypothesis, (d) critical value and the test statistic, (e) alpha and the null hypothesis, and (f) beta and alpha. Figure 3 shows example questions for each of the five posttest question types.

#### Procedure

This experiment lasted a maximum of 55 min. At the start of the experiment, all subjects were instructed that they would be presented with 15 slides on hypothesis testing. Subjects were told that they would be given 20 min to study and were asked to carefully review each slide and do the best they could to learn the material. Subjects were also notified that they would be given a short quiz once the study session was complete. Subjects were told that they could move to the previous slide by pressing the left arrow key and to the following slide by pressing the right arrow key. Subjects were asked to press the spacebar when they were ready to begin.

Subjects were shown a total of 15 study slides, which were presented one at a time. If subjects attempted to move beyond the final slide before the study time was complete, the screen was cleared and they were shown the remaining study time and were asked to continue to study. In such cases, subjects were asked to press the spacebar to continue, which took them back to the slide they were previously viewing. After the 20-min study time expired and subjects attempted to view another slide (i.e., pressed the left or right arrow key), the screen was cleared and subjects moved to the next phase of the experiment.

In the quiz phase, subjects were given a short quiz that consisted of six questions (relational or definitional, depending on the subject’s condition). For each question, subjects were asked to select the correct response by pressing the key that corresponded to the correct answer choice (a–d). After each response, subjects were shown whether they were correct, along with the letter of the correct response. Feedback was displayed directly below the question and answers and remained on the screen for 5 s.

Once subjects completed the quiz, they were asked to go back and study the training slides for an additional 10 min. This additional study phase was included for two reasons. The first reason was to increase the chance that subjects would be able to learn the study materials. The second reason was to emphasize the knowledge that was trained in each condition, as subjects are likely to focus their re-study on the content they are quizzed on (McDaniel et al., 2015). Thus, subjects who were quizzed on relations might be more likely to attend to the relationships among concepts during re-study, whereas subjects who were quizzed on definitions might instead focus on the concepts’ definitions. The re-study phase was followed by a posttest that consisted of 30 questions (six each from the five types described earlier). Subjects were not provided feedback on the posttest. There was a 300-ms interval that followed each quiz and posttest question. The orders in which quiz and posttest questions were presented were randomized for each subject. Once subjects completed the posttest, they were given a summary sheet that explained the study and were thanked for their participation.

### Results and discussion

#### Quiz performance

First, we examined how subjects performed on the quiz during the learning phase. A *t* test revealed that subjects in the definitional condition performed better on the definitional quiz (*M* = 0.631) than subjects in the relational condition performed on the relational quiz (*M* = 0.399), *t*(188) = 6.13, *p* < 0.001, *d* = 0.894, *MSE* = 0.038. This result suggests that the relational questions were more challenging to learn than were the definitional questions.

Two separate linear regression models were also conducted (one for each training condition), which revealed that performance on the quiz was positively related to posttest performance for subjects in both the relational and definitional training conditions (both *p*s < 0.001), as subjects who performed better on the quiz also performed better on the posttest (*β*_definitional_ = 0.448 and *β*_relational_ = 0.397). Additionally, for each condition, five separate linear regressions were conducted with quiz performance as a predictor of performance on each posttest question type. As in the previous set of analyses, these results found a positive relationship between these two factors, such that subjects who performed better on the quiz also performed better on all the posttest question types (all *β*s > 0.323 and all *p*s < 0.002).

#### Posttest performance

**Fig. 4:**
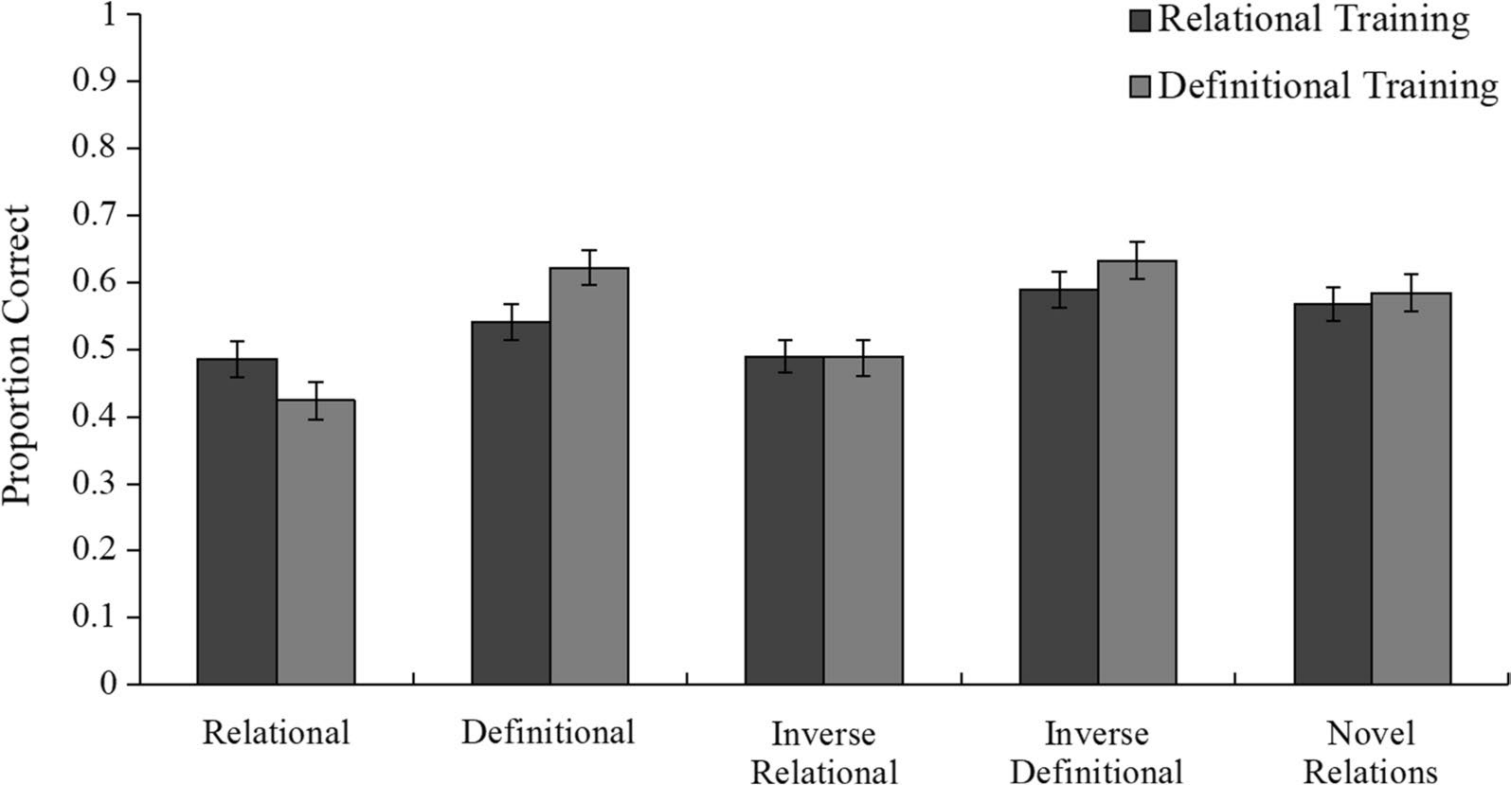
Mean performance and standard error of the mean for each condition on each question type in Experiment 1. Chance performance is 25%

Next, we examined subjects’ posttest performance. Figure 4 shows each group’s mean posttest performance on each question type. First, performance on the posttest was analyzed using a mixed-model ANOVA with posttest question type as a within-subject factor (relational, definitional, inverse relational, inverse definitional, novel relations) and training condition as a between-subjects factor (relational vs. definitional). This analysis showed an interaction, *F*(4, 752) = 3.930, *p* = 0.004, ${\eta }_{\mathrm{p}}^{2}$ = 0.02, *MSE* = 0.035, such that subjects who received relational quizzes performed numerically better on relational questions than did subjects who received definitional quizzes, whereas the opposite pattern held for definitional questions.

Five separate *t* tests were conducted to evaluate the simple effect of condition for each question type. A nonsignificant trend was found for the difference between the two conditions on relational questions, *t*(188) = 1.60, *p* = 0.11, *SE* = 0.027, *d* = 0.23, and a significant difference was found on definitional questions, *t*(188) = − 2.14, *p* = 0.034, *SE* = 0.027, *d* = 0.31. No differences were found between the two groups on any of the other question types (i.e., inverse relational, inverse definitional, and novel relations; all *p*s > 0.24).

These results show that emphasizing definitions and relations during training can indeed help subjects learn this knowledge. However, subjects exhibited a high degree of training specificity, as those who were trained on definitions performed better at test on definitional questions that tested the same definitions, whereas those who were trained on relations tended to perform (numerically) better at test on relational questions that tested the same relations.

Furthermore, there were no differences in performance between the conditions on any of the three transfer question types. This finding is somewhat surprising, given that the inverses of the relational and definitional questions covered essentially the same information that subjects were quizzed on. One might expect that training that improves performance on questions asking how Concept A is related to Concept B would also improve performance on questions that ask how Concept B is related to Concept A. Likewise, training that improves performance on recognizing which definition corresponds to a given term might also be expected to improve performance on questions that test which term corresponds to a given definition. However, neither of these outcomes was observed, indicating that the advantage conferred by training in each condition was specific to the way in which the questions were asked (for related findings see Pan et al., 2016a, 2016b; Rickard et al., 1994). These results are thus in line with the predictions that follow from transfer-appropriate processing, as subjects performed better on the same information when it was tested in the way it was trained.

## Experiment 2

Experiment 1 findings suggest that training subjects on individual concept definitions does not help them better learn how those concepts are related to each other (compared to relational training). Similarly, training subjects on the relationships among concepts does not seem to help them better learn the definitions for those concepts (compared to definitional training). Thus, emphasizing only relations or only definitions might be insufficient for subjects to fully learn a conceptual knowledge system. For this reason, it might be that both relational and definitional knowledge should be emphasized during the learning process, so as to better facilitate the transfer of learning when these types of principles are subsequently encountered in novel scenarios.

On the other hand, in Experiment 1, each of the principles that were quizzed in the learning phase was queried only once, which may not have been sufficient for subjects to learn and comprehend these principles well enough to demonstrate differential knowledge transfer on the posttest. This possibility raises the question of what kind of learning and transfer differences might occur under a stronger training manipulation than that used in Experiment 1.

A related question is whether the lack of transfer effects observed in Experiment 1 was due to subjects in the training conditions not learning and comprehending the to-be-learned principles well enough to transfer them to novel scenarios or whether both training conditions aided concept acquisition and transfer equally well. That is, it is possible that emphasizing external relations among concepts and internal concept definitions both facilitate the learning and transfer of knowledge but do so equally well, which may account for the lack of differences between the training conditions on the transfer items in Experiment 1.

To examine these possibilities, a second experiment was conducted, which was similar to Experiment 1, but which consisted of three primary additions. First, to give subjects more ample opportunity to learn and comprehend the knowledge that was quizzed, subjects were given three different sets of quizzes during the learning phase, which each tested the same information (see Butler et al., 2017, for a similar approach).

Second, a mixed training condition was included, such that during the learning phase, subjects in this condition were quizzed on both external relations among concepts and concept definitions. Subjects in the mixed condition are thus quizzed on relational and definitional knowledge in the same way that this knowledge is assessed on the relational and definitional posttest questions. For this reason, based on the findings from Experiment 1, along with theories on transfer-appropriate processing (Morris et al., 1977), it was predicted that subjects in the mixed condition would perform better on the definitional questions than would subjects in the relational condition, and better on the relational questions than would subjects in the definitional condition.

Third, a control condition was included, in which subjects were presented the same PowerPoint-like slides for study that subjects in the training conditions were presented, but were not quizzed on any of this knowledge. This condition was added to enable measurement of the absolute levels of learning engendered by the quizzes in the training conditions, separate from any differences between those conditions. If the lack of condition differences in Experiment 1 on inverse definition, inverse relation, and novel relation questions reflected subjects’ inability to transfer their knowledge to these questions, then there should be no differences between training and control conditions on these questions. However, if the quiz-based training was beneficial for these questions in Experiment 1 (equally in the two training conditions), then the training groups should outperform the control group in Experiment 2 on these questions.

If the benefits of quizzing are restricted to the knowledge that is quizzed, subjects should demonstrate a selective training advantage. Specifically, subjects who receive definitional training should outperform control subjects only on questions that assess definitional knowledge (i.e., definitional and inverse definitional questions) and subjects who receive relational training should outperform control subjects only on questions that assess relational knowledge (i.e., relations, inverse relations, and novel relations). Furthermore, because subjects in the mixed training condition are trained on both types of knowledge, they should outperform control subjects on all question types.

On the other hand, theories on transfer-appropriate processing (Morris et al., 1977) predict that transfer will be restricted to the manner in which knowledge was quizzed during training. By this account, in comparison with the control subjects, definitional training subjects should perform better on only definitional questions (and not inverse definitional questions), relational training subjects should perform better on only relational questions (and not inverse or novel relations), and mixed training subjects should perform better on both relational and definitional questions.

### Methods

#### Participants

Two hundred seventy undergraduate students participated in this experiment for course credit in an introductory psychology course at Syracuse University. We used the same data collection strategy and minimum criterion for stopping data collection in the present experiment as in Experiment 1.

The student population from which subjects were sampled consisted mainly of freshmen. Approximately 54% of students at this university identify as female and approximately 56% of students are White. Approximately 61% of students are 21 years of age or younger. This study was approved by the institutional review board at Syracuse University.

#### Design and materials

Subjects were randomly assigned to four conditions: relational training (*N* = 68), definitional training (*N* = 70), mixed training (*N* = 68), and control (*N* = 64). The materials consisted of the same study slides as used in Experiment 1 and six quizzes: three relational quizzes and three definitional quizzes. Two of the quizzes were the relational and definitional quizzes from Experiment 1. Four additional quizzes were therefore developed, two relational quizzes and two definitional quizzes.

**Fig. 5:**
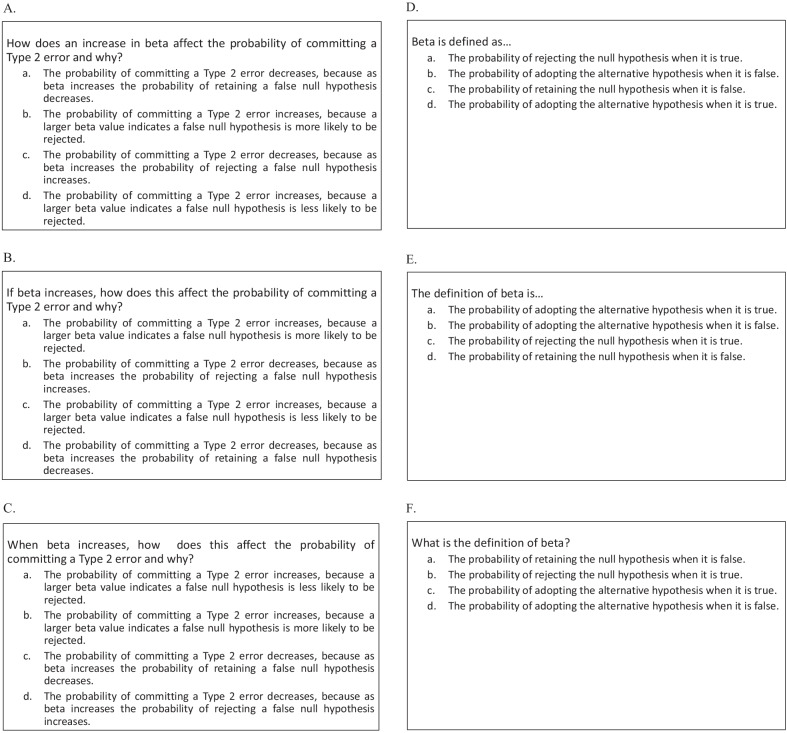
Example questions from each quiz in Experiment 2 that test the same relation (Panel **A**–**C**) and the same definition (Panel **D**–**F**). **A** correct answer = *d*. **B** correct answer = *c*. **C** correct answer = *a*. **D** correct answer = *c*. **E** correct answer = *d*. **F** correct answer = *a*

To keep the quizzes during the learning phase abstract, the new quizzes were highly similar to their corresponding quizzes from Experiment 1. That is, all relational quizzes were highly similar to one another and all definitional quizzes were highly similar to one another. Specifically, the same relations were tested in all three relational quizzes, and the same definitions were tested in all three definitional quizzes. For all of the questions that tested each principle, the primary differences across quizzes were minor changes in wording of the prompt and a change in the order of response alternatives (see Fig. 5A–F). Lastly, the posttest was identical to the one used in Experiment 1.

#### Procedure

The procedure for the training conditions (relational, definitional, and mixed) was similar to that of Experiment 1, with the exception that each subject completed three quizzes as opposed to one. To accommodate the time required for these additional quizzes, the amount of time subjects were given to study was reduced by 5 min. The first study phase was set to 18 min (reduced by 2 min), and the second study phase was set to 7 min (reduced by 3 min).

As in Experiment 1, subjects in the training conditions (i.e., all but the control condition) first studied the PowerPoint-like slides and were then quizzed and given a self-paced rest break, after which they were asked to study these slides again. Next, subjects in the training conditions were given another self-paced rest break and were presented instructions that notified them that they would be given a second multiple-choice quiz on the material. After completing this quiz, subjects were given another self-paced rest break, which was followed by a third multiple-choice quiz. Thus, subjects in the training conditions completed two rounds of studying and three rounds of quizzing. For each quiz question, after subjects entered a response, they were shown correct answer feedback, which was presented on the screen for 7 s (an increase of 2 s from Experiment 1; this change was made to give subjects more time to process feedback and thereby provide them greater opportunity to learn from the quizzes).

For each subject, the order that they completed the three quizzes was randomized, as was the order that the questions on each quiz were presented. For the relational and definitional conditions, each of the principles that were quizzed was tested three times (once on each quiz).

In the mixed training condition, on each quiz, subjects were presented three relational questions and three definitional questions. For each subject, the principles that were tested on the first quiz were randomly selected, subject to the constraint that three were relational and that three were definitional. On the second quiz, the subject was tested on the three relations and three definitions that were not tested on the first quiz. For the third quiz, the three relations and three definitions that each subject was tested on were randomly selected.

Control subjects were not quizzed and only completed the two study phases, separated by a self-paced rest break.

After the learning phase, all subjects were given a self-paced rest break, after which they completed the posttest (as in Experiment 1). Lastly, after completing the posttest, subjects were asked to fill out a short questionnaire about the number of courses they had completed in (a) mathematics, (b) statistics, (c) science, and (d) research methodology. All other procedures in the learning phase and posttest were identical to those used in Experiment 1.

### Results and discussion

First, as a check of random assignment, we examined whether subjects in the four conditions differed more than would be expected by chance in the number of mathematics, statistics, science, and research method courses they had taken. A multivariate ANOVA was conducted with condition as a between-subjects factor (relational vs. definitional vs. mixed vs. control) and four dependent variables: (a) math courses, (b) statistics courses, (c) science courses, and (d) research method courses. The result revealed no statistically reliable differences among the conditions, Wilks’ Lambda = 0.238, *F*(12, 795) = 0.781, *p* = 0.667, *η*_*p*_^*2*^ = 0.012. We also note that the numbers of these courses that students had completed were not predictive of performance on the posttest (multiple regression, omnibus *p* = 0.757). Overall, subjects reported having taken very few of these courses (*M*_math_ = 0.993, *SD* = 0.823; *M*_statistics_ = 0.637, *SD* = 0.567; *M*_science_ = 1.09, *SD* = 0.847; *M*_research methods_ = 0.407, *SD* = 0.631).

#### Quiz performance

Next, we examined subjects’ quiz performance using a mixed-model ANOVA, wherein quiz number was included as a within-subject factor (first vs. second vs. third quiz) and training condition was included as a between-subjects factor (relational vs. definitional vs. mixed). The results revealed a main effect of quiz number, *F*(2, 406) = 44.66, *p* < 0.001, ${\eta }_{\mathrm{p}}^{2}$ = 0.180, MSE = 0.035. Specifically, least significant difference (LSD) post hoc comparisons revealed that subjects (collapsing across training condition) performed better on the second quiz (*M* = 0.584, *SE* = 0.018) than on the first (*M* = 0.480, *SE* = 0.018; *p* < 0.001) and better on the third quiz (*M* = 0.652, *SE* = 0.018) than on the second (*p* < 0.001). This finding suggests that the training procedures helped subjects improve their knowledge of the material as they progressed through the learning phase.

A main effect of training condition was also observed, *F*(2, 406) = 44.60, *p* < 0.001, ${\eta }_{\mathrm{p}}^{2}$ = 0.180, *MSE* = 0.035, wherein subjects who received definitional quizzes (*M* = 0.704, *SE* = 0.026) performed better than subjects in the relational (*M* = 0.503, *SE* = 0.027) and mixed training (*M* = 0.508, *SE* = 0.027) conditions (both *p*s < 0.001); no differences on quiz performance were observed between the relational and mixed training conditions (*p* = 0.879). Hence, as in Experiment 1, it seems that the definitional quiz questions were less challenging than the relational quiz questions. In line with this interpretation, a paired-samples *t* test revealed that subjects in the mixed training condition performed better on the definitional quiz questions (*M* = 0.600, *SE* = 0.029) than on the relational quiz questions (*M* = 0.400, *SE* = 0.026), *t*(67) < 0.001, *d* = 0.780, *SE* = 0.031. No interaction was observed between quiz number and training condition, *F*(4, 406) = 1.98, *p* = 0.097, ${\eta }_{\mathrm{p}}^{2}$ = 0.019, *MSE* = 0.035.

Lastly, we conducted separate linear regressions for subjects in each of the training conditions, with quiz performance as a predictor of posttest performance. These analyses showed that quiz performance was positively related to performance on the posttest, such that subjects who performed better on the quiz also performed better on the posttest (all *β*s > 0.571 and all *p*s < 0.001). These regression models were further partitioned by posttest question type (i.e., five separate linear regressions were conducted for each training condition), which showed the same positive relationship as the previous set of analyses between quiz performance and performance on each of the posttest question types for each training condition (all *β*s > 0.550 and all *p*s < 0.001).

#### Posttest performance

**Fig. 6:**
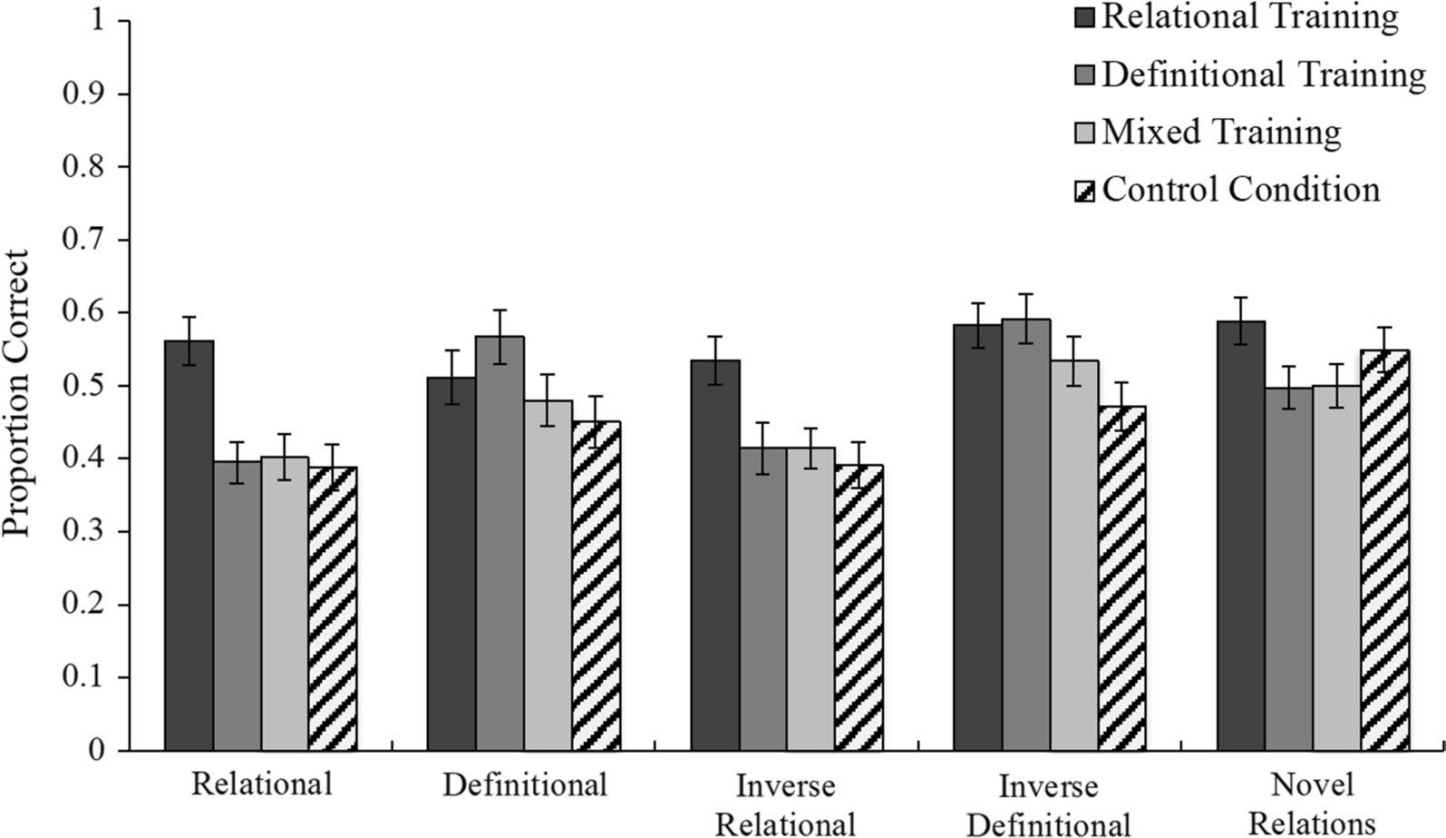
Mean performance and standard error of the mean for each condition on each question type in Experiment 2. Chance performance is 25%

For the primary analysis, we examined posttest performance in the training and control conditions. Figure 6 shows each condition’s mean posttest performance on each question type. A mixed ANOVA was conducted with question type (relational, definitional, inverse relational, inverse definitional, novel relations) as a within-subject factor and training condition (relational vs. definitional vs. mixed vs. control) as a between-subjects factor. The results revealed a statistically significant interaction between question type and training condition, *F*(12, 1064) = 3.79, *p* < 0.001, ${\eta }_{\mathrm{p}}^{2}$ = 0.041, *MSE* = 0.035, indicating that the differences in posttest performance among the training conditions varied by question type.

To remind the reader, the training conditions are intended to address a fundamentally different set of questions than the control condition. For this reason, we report separate follow-up analyses (a) restricted to the training conditions and (b) comparing the training conditions to the control conditions.

##### Comparison among training conditions

When the control condition was not included in the mixed ANOVA model, a statistically significant interaction was still observed between question type and training condition, *F*(8, 812) = 4.24, *p* < 0.001, ${\eta }_{\mathrm{p}}^{2}$ = 0.040, *MSE* = 0.035. Follow-up LSD post hoc comparisons showed that subjects in the relational training condition outperformed subjects in the definitional and mixed training conditions on the relational (*M*_relational_ = 0.561, *SE*_relational_ = 0.032; *M*_definitional_ = 0.395, *SE*_definitional_ = 0.028; *M*_mixed_ = 0.402, *SE*_mixed_ = 0.032), inverse relational (*M*_relational_ = 0.534, *SE*_relational_ = 0.033; *M*_definitional_ = 0.414, *SE*_definitional_ = 0.035; *M*_mixed_ = 0.414, *SE*_mixed_ = 0.028), and novel relations questions (*M*_relational_ = 0.588, *SE*_relational_ = 0.032; *M*_definitional_ = 0.498, *SE*_definitional_ = 0.029; *M*_mixed_ = 0.500, *SE*_mixed_ = 0.030; all *p*s < 0.042, all *d*s > 0.349). Additionally, subjects in the definitional training condition (*M* = 0.567, *SE* = 0.034) marginally outperformed subjects in the mixed training condition (*M* = 0.480, *SE* = 0.035) on the definitional questions, *t*(136) = 1.72, *p* = 0.084, *d* = 0.305, *SE* = 0.049. No other performance differences on any of the question types were found among the training conditions (all *p*s > 0.241).

Given these results, it seems that the two additional quizzes included in Experiment 2 strengthened the efficacy of the relational training relative to that of definitional training. As shown in Table 1, the contrast between these conditions favored the relational training condition more in Experiment 2 than in Experiment 1 for all five question types. In particular, although Experiment 2 subjects in the definitional training condition (*M* = 0.567, *SE* = 0.034) performed numerically better on definitional questions than did subjects in the relational training condition (*M* = 0.512, *SE* = 0.037), this difference was not statistically reliable, *t*(136) = 1.09, *p* = 0.279, *d* = 0.187, *SE* = 0.050. We discuss possible explanations for these results and how they relate to those of Experiment 1 in the Limitations and Future Directions subsection of the General Discussion.

**Table 1:** Effect size for each pairwise comparison between conditions partitioned by question type in Experiments 1 and 2

	Question type				
	Relational	Definitional	Inverse relational	Inverse definitional	Novel relations
*Experiment 1*					
Relational versus definitional	*d* = 0.232	*d* = − 0.312*	*d* = 0.008	*d* = − 0.167	*d* = − 0.063
*Experiment 2*					
Relational versus definitional	*d* = 0.664*	*d* = −0.186	*d* = 0.428*	*d* = − 0.026	*d* = 0.367*
Relational versus mixed	*d* = 0.610*	*d* = 0.110	*d* = 0.474*	*d* = 0.184	*d* = 0.350*
Definitional versus mixed	*d* = − 0.027	*d* = 0.305	*d* = 0.004	*d* = 0.201	*d* = − 0.001
Relational versus control	*d* = 0.672*	*d* = 0.212	*d* = 0.549*	*d* = 0.433*	*d* = 0.153
Definitional versus control	*d* = 0.030	*d* = 0.412*	*d* = 0.088	*d* = 0.437*	*d* = − 0.213
Mixed versus control	*d* = 0.056	*d* = 0.106	*d* = 0.098	*d* = 0.233	*d* = − 0.202

^*^Indicates *p* < 0.05

##### Training conditions versus control condition

As explained above, the control condition was included to enable determination of whether any null differences among the training conditions were due to all three training types producing similar levels of learning and transfer, or whether they were the result of floor effects wherein none of the training types aided learning beyond study of the to-be-learned material. To address this question, we conducted a series of pre-planned supplementary analyses, wherein for each of the question types, performance in each of the training conditions was compared to that in the control condition.

##### Relational training versus control condition

The first set of analyses revealed that subjects in the relational training condition outperformed control subjects on relational, inverse relational, and inverse definitional questions (all *p*s < 0.016, all *d*s > 0.432). However, no reliable performance differences were observed between these conditions on the definitional or novel relations questions (both *ps* > 0.229, both *d*s < 0.213). Taken together, these results suggest that relational training helped subjects better learn and comprehend both the principles they were quizzed on and some principles that were not directly quizzed (i.e., inverse definitional questions), well enough to transfer and apply them to novel scenarios.

##### Definitional training versus control condition

The second set of analyses revealed that subjects in the definitional training condition outperformed control subjects on definitional and inverse definitional questions (both *ps* < 0.020, both *d*s > 0.411). No reliable performance differences were observed between these conditions on the relational, inverse relational, or novel relations questions (all *ps* > 0.223, all *d*s < 0.213). Thus, subjects who received definitional training were able to learn and transfer the knowledge they were quizzed on to novel scenarios and to new question types. However, this benefit applied only to the definitional knowledge on which subjects were trained, and not to relational knowledge. These results thus demonstrate that definitional training once again (as in Experiment 1) produced a selective training benefit.

##### Mixed training versus control condition

Lastly, the third set of analyses revealed no performance differences between subjects in the mixed training and control conditions (all *p*s > 0.188, all *d*s > 0.234). For this reason, it does not appear that subjects in the mixed training condition were able to learn and comprehend the to-be-learned material any better than control subjects and thus did not appear to benefit from being quizzed on both relations and definitions.

#### Summary

The results from Experiment 2 show that subjects in the relational training condition outperformed all other subjects on relational and inverse relational questions. These findings suggest that relational training better aids learning and transfer of relational knowledge than does definitional or mixed training. Moreover, subjects who received relational training outperformed subjects in the definitional and mixed training conditions on novel relation questions, and outperformed control subjects on inverse definitional questions. Thus, relational training not only seems to benefit the learning and transfer of the knowledge that is quizzed, but it also seems to benefit the learning and transfer of some knowledge that is not directly quizzed. These results are therefore in line with the predictions that follow from the systematicity principle (Gentner, 1983, 2010; Gentner & Hoyos, 2017), as highlighting the relations among concepts (through quizzing) seemed to facilitate better concept learning and transfer than emphasizing the internal structure of concept definitions.

By contrast, definitional training showed a benefit over studying alone only for definitional and inverse definitional questions, indicating the specificity of this type of learning. The mixed condition showed no benefits over the control condition at all, suggesting some manner of interference effect between relational and definitional information that should be a focus of future research.

## General discussion

We report two experiments using a learning paradigm that alternates between studying and quizzing to emphasize the learning of definitions of concepts (internal structures) and relations among those concepts (external structures). In Experiment 1, one group was quizzed on definitions and the other on relations. The results revealed a selective training advantage, wherein subjects who were quizzed on definitions performed significantly better on definitional questions than subjects who were quizzed on relations, whereas subjects who were quizzed on relations performed numerically better on relational questions than subjects who were trained on definitions.

To strengthen this manipulation, Experiment 2 incorporated three quizzes that all tested the same information. A mixed training condition was also included, which quizzed both relations and definitions, as was a control condition in which subjects only studied. The stronger manipulation led to a robust advantage of relational training, as its benefits generalized to four of the five posttest question types (relational, inverse relational, novel relations, and inverse definitional questions). In contrast, the benefits of definitional training generalized only to definitional and inverse definitional questions (i.e., knowledge that was quizzed). Furthermore, mixed training did not aid learning, which points to an important limitation of quizzing subjects on both relations and definitions at once. In sum, relational training helped subjects not only to better learn and transfer the knowledge they were quizzed on to novel scenarios, but also to better learn and transfer material that was not quizzed.

### Theoretical implications

The systematicity principle (Gentner, 1983, 2010; Gentner & Hoyos, 2017; Gentner & Markman, 1997; Markman & Gentner, 2000) predicts that relational training should produce the best posttest performance on all question types, because relational quizzes were intended to leverage subjects’ preference for systematicity by highlighting the interconnections among the to-be-learned concepts. In contrast, definitional training emphasized concept definitions, which are arguably more central to learning a conceptual system than relations, which leads to the prediction that definitional training should produce the best performance on all question types. Alternatively, transfer-appropriate processing (Morris et al., 1977) holds that learning should be best for the manner in which knowledge is assessed during training. Thus, subjects who trained on definitions should perform best on definitional questions, and subjects who trained on relations should perform best on relational questions. According to this account, mixed training should therefore lead to better performance on definitional questions than relational training, and better performance on relational questions than definitional training.

However, none of these predictions fully materialized. Although the selective training advantage from Experiment 1 provides partial support for the predictions from transfer-appropriate processing, Experiment 2 results did not support this prediction and in fact provide evidence against it. Specifically, the benefits of relational and definitional (to a lesser extent) training extended beyond the specific ways in which these principles were assessed during training.

Altogether, Experiment 2 results seem to most closely align with the predictions from the systematicity principle, as the benefits of relational training were quite strong and led to the best transfer of learning. However, Experiment 2 also showed that the benefits that quizzing relational knowledge has on learning definitional knowledge do not outweigh (though may be comparable to) the benefits of quizzing that definitional knowledge. Nevertheless, the utility of emphasizing the definitions of concepts seems somewhat modest when compared to emphasizing the relations among those concepts, as the latter seems to produce similar benefits to the former, while also producing superior transfer of learning.

One particularly informative finding was that the relational quizzes were more challenging than definitional quizzes. Relational quizzes may have therefore engaged greater depth of processing than definitional quizzes, and in doing so, facilitated better learning and comprehension of the quizzed material (see Bjork, 1994). This possibility may help explain why relational training led to superior transfer of learning than definitional training (although it does not explain the poor performance of the mixed condition).

#### External versus internal concept acquisition

An important claim of the present work is that it is possible for people to learn the external relationships among a set of concepts even if they have not yet learned those concepts’ internal structure. For example, students can learn the relationships among *alpha*, *beta*, and the *critical value* if they have not learned what each of those concepts is (i.e., the concepts’ definitions). This position is motivated by theories of conceptual development. According to Carey’s (2011) theory of Quinian bootstrapping, a new concept is initially represented as an empty symbol (e.g., a label), which serves as a temporary placeholder for the concept. Initially, people learn the relations between this symbol and other concepts (i.e., external relations), which are then built into a conceptual network. Through analogical reasoning, people eventually learn to recognize concrete scenarios that correspond to this symbol, and the structure of these scenarios comes to form the internal structure of the concept. Moreover, according to this theory, it is possible for all concepts within a conceptual network to be novel and for people to nevertheless learn how they are related to one another. For instance, students may have no understanding of what *alpha* or the *critical value* is, but upon encountering concrete examples that specify the relationship between these concept labels, they can likely learn that the two are inversely related without ever learning the internal structure of either concept.

Under the view of Quinian bootstrapping (Carey, 2011), the learning of external structure thus can precede the acquisition of internal relations. The learning of external relations might therefore be more central to concept acquisition than is the learning of internal structure, which might help to further explain the superiority of relational training that was observed in Experiment 2.

### Limitations and future directions

#### Selective advantage of definitional training

One notable discrepancy in the findings between the two experiments is that in Experiment 1, subjects who received definitional training performed better on definitional questions than subjects who received relational training, but this effect was not statistically significant in Experiment 2. The primary difference between the two experiments regarding these conditions was that Experiment 1 used one quiz during the learning phase, whereas Experiment 2 used three. Taken together, these results may point to an interaction between the type of training subjects receive and the number of quizzes they complete. That is, relational quizzing may continue to gain potency with repetition, as compared to definitional quizzing.

As noted in the previous subsection, the material on relational quizzes appeared to be more challenging than the material on definitional quizzes. One speculative possibility is that this greater difficulty left room for further learning from repetitions of the quiz. In contrast, a single quiz may be sufficient to reap the available benefits of definitional training.

If this conjecture is correct, then the greater impact of relational training in Experiment 2 might have partially masked the benefit of definitional over relational training on the definitional questions. Nevertheless, when compared to control subjects, definitional training produced robust transfer of definitional knowledge (and not of relational knowledge), which suggests there was indeed a selective advantage of definitional training in Experiment 2.

#### Mixed training condition

Despite being trained on relational and definitional knowledge, mixed training did not benefit learning and transfer any more than simply studying. One possibility is that quizzing both relational and definitional knowledge concurrently leads to interference, which may strain working memory and impede learning. One way to test this hypothesis would be to compare the present design that interleaved relational and definitional quiz questions with a blocked design in which subjects are trained on relational and definitional knowledge separately (e.g., relational quizzes in Block 1, definitional quizzes in Block 2).

Another possible explanation for the poor performance in the mixed condition is that, unlike subjects in the relational and definitional training conditions, who were tested on each of the quizzed principles three times, subjects in the mixed training condition were quizzed only twice on half of the quizzed principles and only once on the other half. This difference was necessary in order to quiz subjects in the mixed training condition on all of the principles that subjects in the relational and definitional training conditions were quizzed on. Although it would have been possible to test the mixed training condition on each of the quizzed principles three times, doing so would have required providing this condition twice as many quizzes as those that were provided to the other training conditions. As a result of this design choice, however, it is possible that subjects in the mixed training condition did not complete enough quizzes to fully learn and comprehend the information they were quizzed on.

On the other hand, although subjects in Experiment 1 were quizzed only once on each principle that was tested during training, definitional training led to better performance on the definitional questions than did relational training. This finding thus demonstrates that a single round of quizzing is sufficient for subjects to learn and transfer (beyond pure study) at least some of the principles that were quizzed.

Nevertheless, it is important to note that this finding from Experiment 1 offers only indirect evidence against the hypothesis that subjects in the mixed condition of Experiment 2 did not complete enough quizzes to fully learn the quizzed material. To more directly test this hypothesis, future work could modify the Experiment 2 design by quizzing the mixed training condition on the same six principles (three relational and three definitional) on all three quizzes and restrict the posttest analyses to the corresponding test questions. This approach would ensure that all training subjects complete the same number of quiz items relevant to the posttest items that are analyzed. On the other hand, this approach would quiz subjects on only half of the relational and half of the definitional quiz questions. Consequently, these subjects might develop a relatively narrow scope of the relational and definitional material that they should try to learn, which may negatively impact learning (particularly for the material that is not quizzed). Addressing these questions extends beyond the scope of the present paper, and we thus leave them for future research to investigate.

### Educational implications

The present findings may help instructors better understand how different types of training and teaching strategies (e.g., emphasizing definitions or relations) can influence student learning. Taken together, our findings suggest that emphasizing relations is the most effective method of training. Although there is likely also a benefit of definitional training, instructors should perhaps avoid using it in conjunction with relational training, as this mixed approach appears to be ineffective.

Furthermore, we highlight that all posttest questions involved novel scenarios that were not encountered during training, and thus provide a test of the transfer of learning. That relational and definitional training support the transfer of learning is of critical importance, as it has been a long-standing goal in education to find methods that facilitate this process (Ellis, 1965; Hajian, 2019; National Research Council, 2012), and has thus far proven fairly elusive in the cognitive and learning sciences (Barnett & Ceci, 2002; Detterman, 1993).

This takeaway is particularly noteworthy given the complexity of our materials, which were taken from an undergraduate upper division statistics and research methods course; these concepts are also taught in graduate level statistics courses. As many instructors who teach such courses can likely affirm, even after an entire semester of instruction, some students cannot learn these concepts. That subjects were able to learn and transfer such complex materials in a single training session that lasted about 25 min speaks to the potent learning efficacy of the training conditions (particularly relational training). It might therefore be useful to translate the relational or definitional training procedures into computer-assisted learning software to help students learn complex relational knowledge systems.

One reason our training procedures might be particularly effective is that they incorporate various well-established learning principles. Specifically, by interspersing quizzing with studying, subjects can alternate between tasks, which can offset boredom and increase engagement during learning (formally known as *cognitive antidote*; see Healy et al., 2017; Kole et al., 2008). This task alternation also allows for studying to be spaced between quizzes, which leverages *spacing effect* principles, wherein learning and memory are better for spaced over massed study (Dempster, 1988; Glenberg, 1979). Additionally, the quizzing component of the training phase incorporates principles of retrieval practice, in which retrieving to-be-learned material improves learning and memory of said content (Carpenter, 2009; Carrier & Pashler, 1992). Quizzing also incorporates principles of feedback learning, as feedback has been shown to be critical for concept acquisition (Benassi et al., 2014; Corral & Carpenter, 2020). Together, these principles may provide a particularly potent boost to learning and transfer.

The astute reader might notice that both the relational and definitional training conditions implement these same principles. However, as discussed earlier, there are two potential additional benefits to training students on relations over definitions. First, quizzing relations might be beneficial because of the critical role of relational reasoning in higher cognition. Second, relations might be better learned because of people’s preference for systematically interrelated knowledge, and they might highlight the concept definitions in addition to their relationships, helping students to learn both. Third, because relational questions seem to be more challenging than definitional questions, relational training might have a more desirable level of difficulty than definitional training. As a result, the former might engage greater depths of processing than the latter, which might thereby better facilitate learning and comprehension (see Bjork, 1994).

The most straightforward method for translating this work to the classroom might be to follow a similar approach to the one we use here, wherein instructors quiz students on the relations among concepts within a given to-be-learned conceptual system. Nevertheless, it is important to note that various outstanding questions remain about how best to translate the present findings to an educational setting. For example, what is the best way to word relational questions so as to optimize their learning benefits? What is the ideal number of relations that students should be taught within a given learning session? Does learning some sets of relations help or hinder the learning of other sets of relations? How should instructors identify the key relations among concepts that will best promote learning? One possible answer to this last question (based on the systematicity principle) is that instructors should focus on highlighting relations that share a greater number of interconnections with other relations (e.g., higher-order relations). For instructors to best utilize relational training in their teaching, it will be important for future research to address these questions.

## Conclusion

Previous work has shown that quizzing learners on concept definitions can help them better learn those concepts (McDaniel et al., 2013). The present paper builds on this work and extends it by examining whether subjects are better able to learn core principles from the academic topic of statistical hypothesis testing through training on relationships among a set of corresponding concepts, through training on the individual concept definitions, or through training on both. The primary finding is that, although there was some degree of training specificity, once the manipulation was strengthened (Experiment 2), relational training was mostly superior to definitional and mixed training, as it led to more robust learning and transfer. This takeaway is particularly relevant to education and instruction, as students are typically required to learn definitions of concepts, as well as how those concepts are related to one another. The present results suggest that instructors should primarily train students on how to-be-learned concepts are related, as this approach seems to aid in learning their definitions and their relations.

## Supplementary Information

**Additional file 1.** All of the materials used in Experiments 1 and 2.

## Data Availability

All data and stimuli presented here are available from the corresponding author upon request.
