# Supplementary material for: The effects of testing the relationships among relational concepts
Source: Cogn Res Princ Implic. 2022 May 31;7:47. doi: 10.1186/s41235-022-00398-2 (PMC9156590; doi:10.1186/s41235-022-00398-2)
Supplement: Supplementary file 1 — Additional file 1. All of the materials used in Experiments 1 and 2. [file 41235_2022_398_MOESM1_ESM.pptx]

## Slide 1
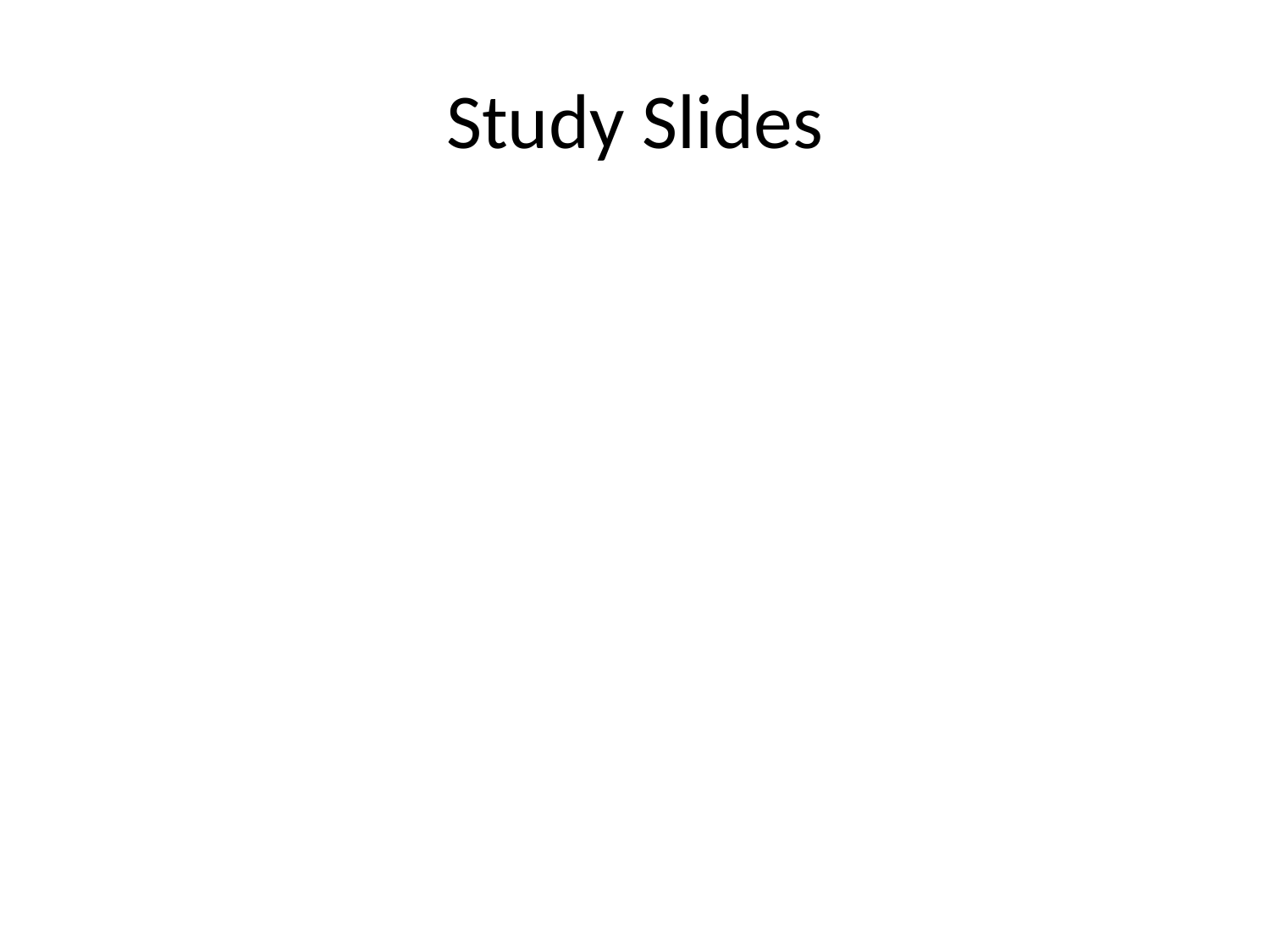

# Study Slides

## Slide 2
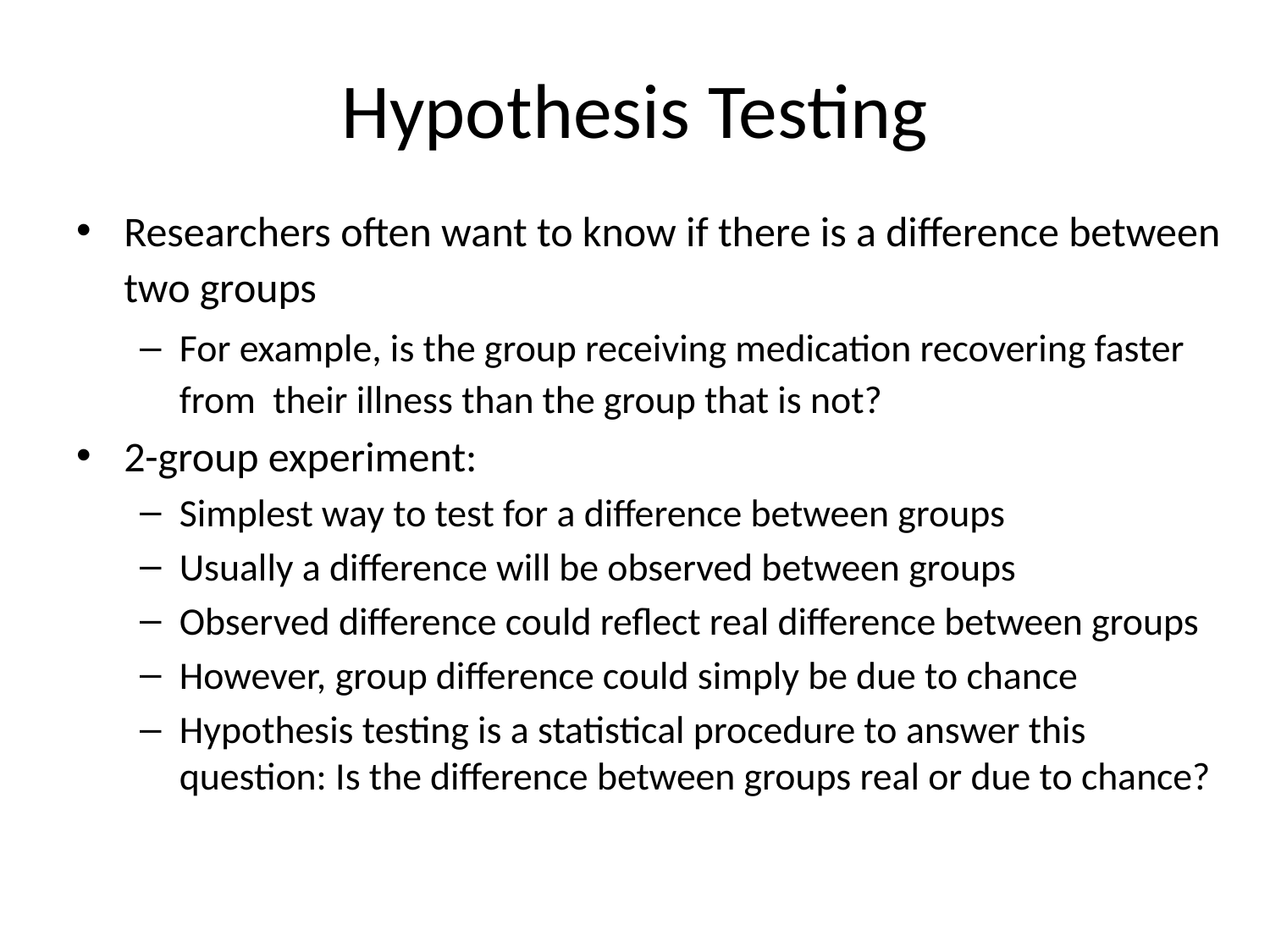

# Hypothesis Testing
Researchers often want to know if there is a difference between two groups
For example, is the group receiving medication recovering faster from their illness than the group that is not?
2-group experiment:
Simplest way to test for a difference between groups
Usually a difference will be observed between groups
Observed difference could reflect real difference between groups
However, group difference could simply be due to chance
Hypothesis testing is a statistical procedure to answer this question: Is the difference between groups real or due to chance?

## Slide 3
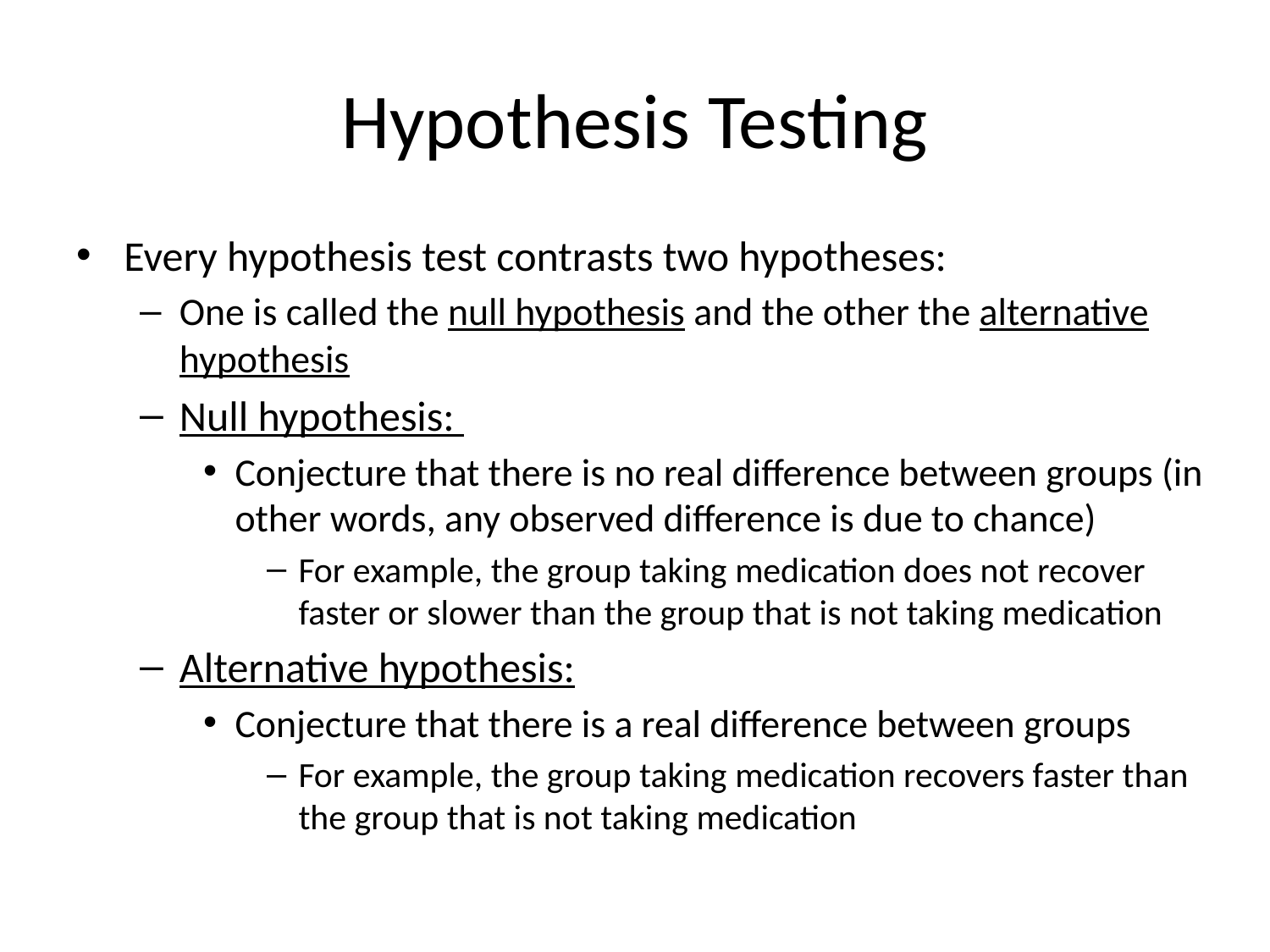

# Hypothesis Testing
Every hypothesis test contrasts two hypotheses:
One is called the null hypothesis and the other the alternative hypothesis
Null hypothesis:
Conjecture that there is no real difference between groups (in other words, any observed difference is due to chance)
For example, the group taking medication does not recover faster or slower than the group that is not taking medication
Alternative hypothesis:
Conjecture that there is a real difference between groups
For example, the group taking medication recovers faster than the group that is not taking medication

## Slide 4
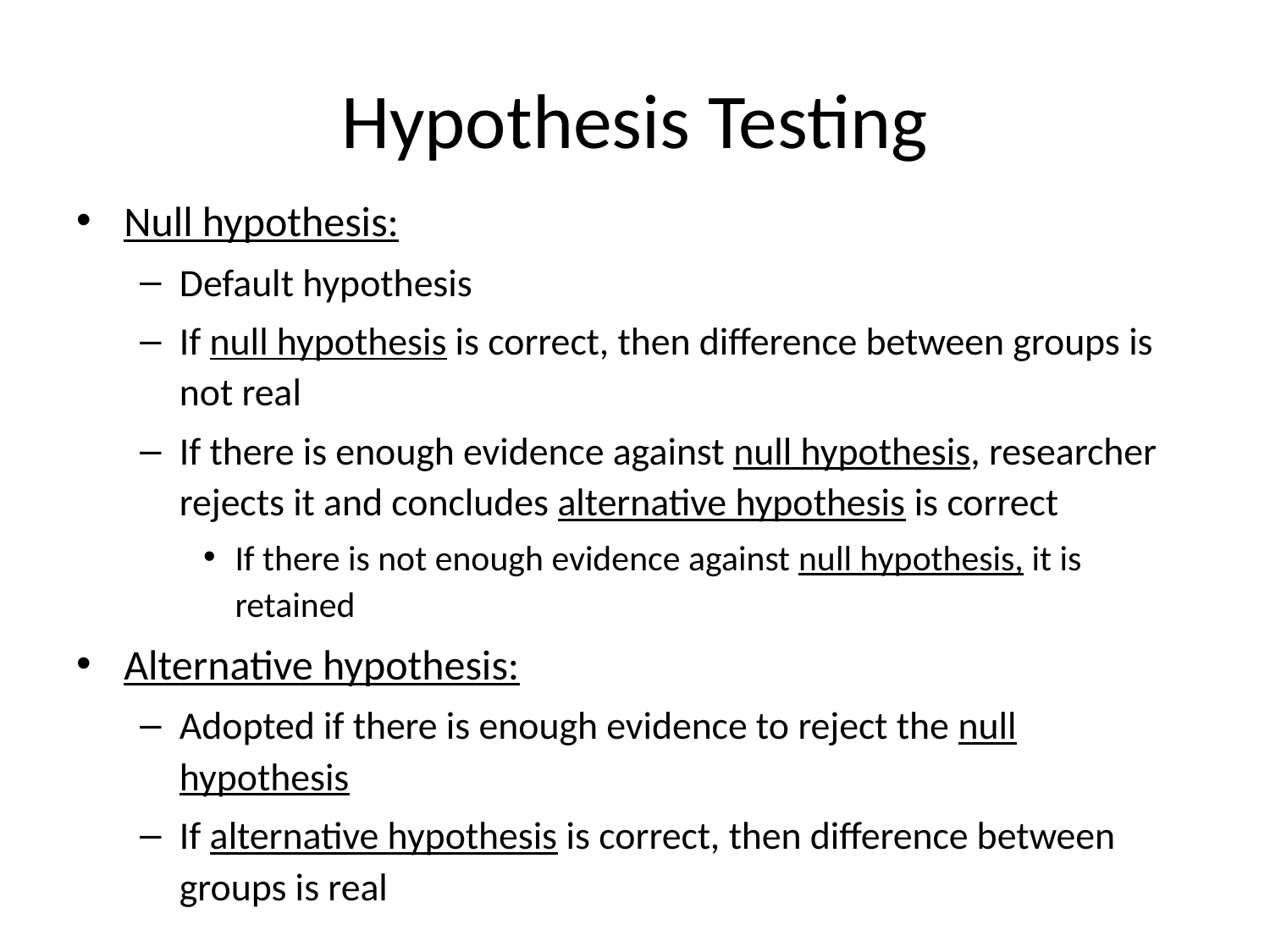

# Hypothesis Testing
Null hypothesis:
Default hypothesis
If null hypothesis is correct, then difference between groups is not real
If there is enough evidence against null hypothesis, researcher rejects it and concludes alternative hypothesis is correct
If there is not enough evidence against null hypothesis, it is retained
Alternative hypothesis:
Adopted if there is enough evidence to reject the null hypothesis
If alternative hypothesis is correct, then difference between groups is real

## Slide 5
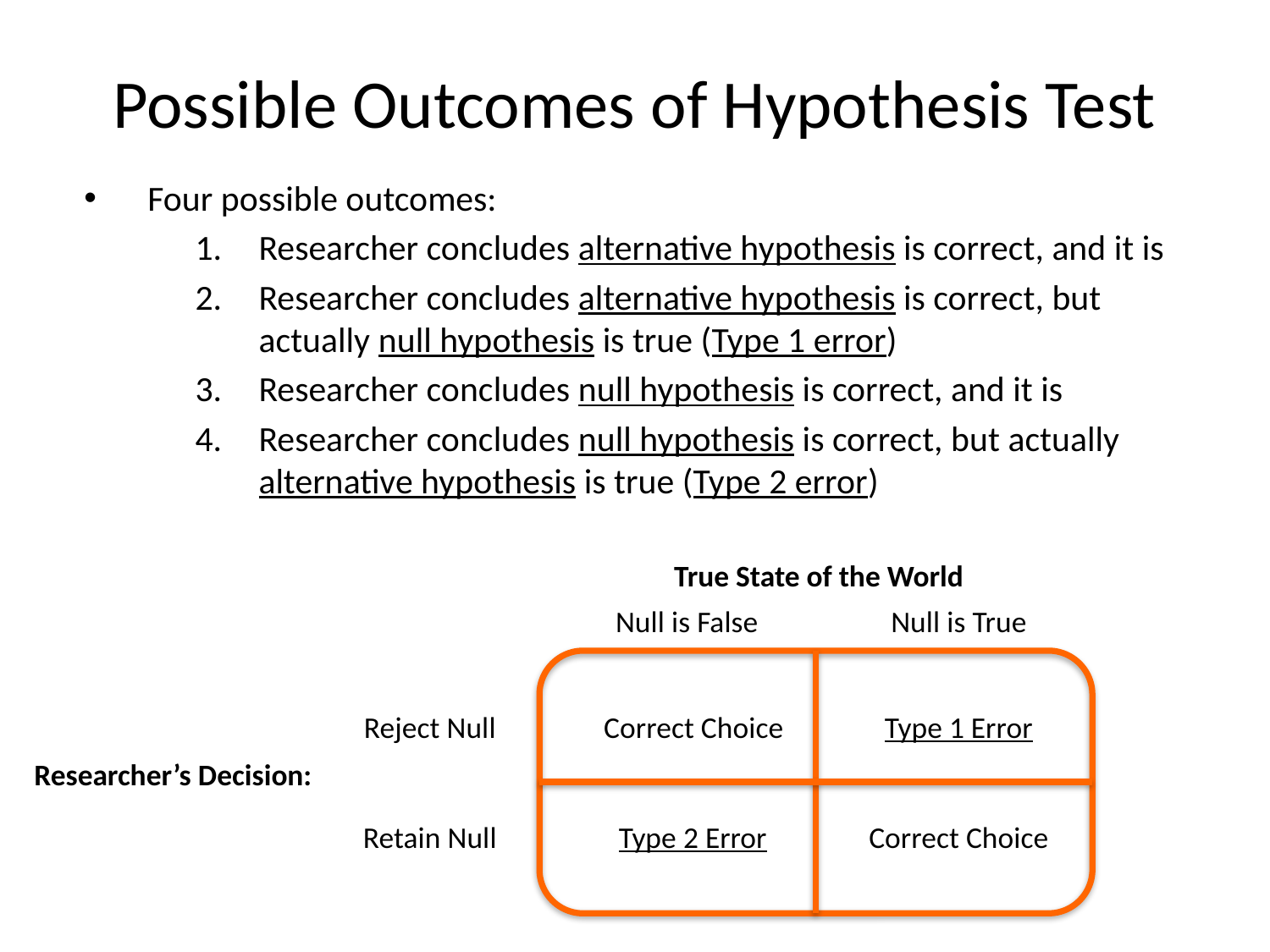

# Possible Outcomes of Hypothesis Test
Four possible outcomes:
Researcher concludes alternative hypothesis is correct, and it is
Researcher concludes alternative hypothesis is correct, but actually null hypothesis is true (Type 1 error)
Researcher concludes null hypothesis is correct, and it is
Researcher concludes null hypothesis is correct, but actually alternative hypothesis is true (Type 2 error)
True State of the World
Null is False
Null is True
Reject Null
Correct Choice
Type 1 Error
Retain Null
Type 2 Error
Correct Choice
Researcher’s Decision:

## Slide 6
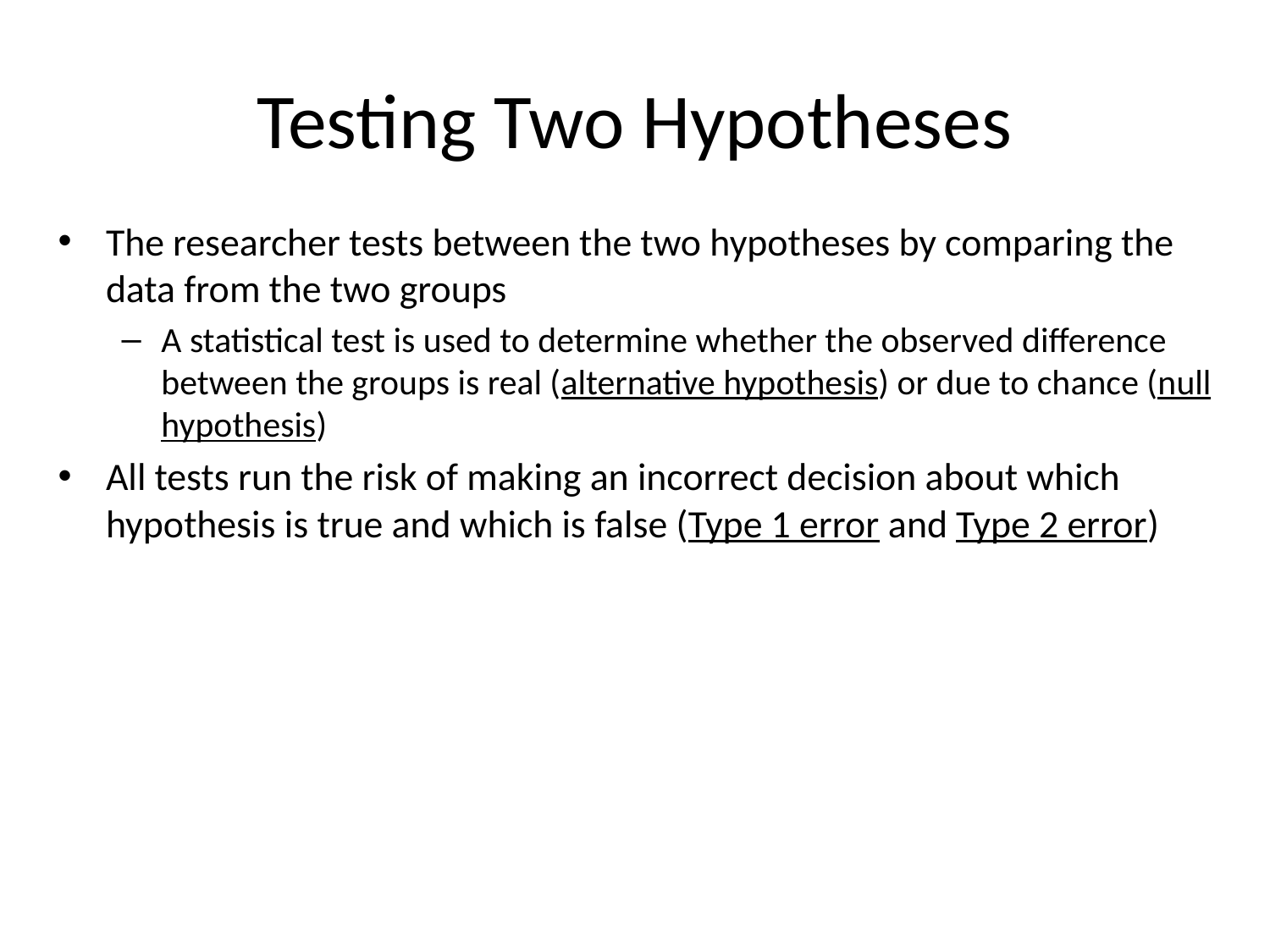

# Testing Two Hypotheses
The researcher tests between the two hypotheses by comparing the data from the two groups
A statistical test is used to determine whether the observed difference between the groups is real (alternative hypothesis) or due to chance (null hypothesis)
All tests run the risk of making an incorrect decision about which hypothesis is true and which is false (Type 1 error and Type 2 error)

## Slide 7
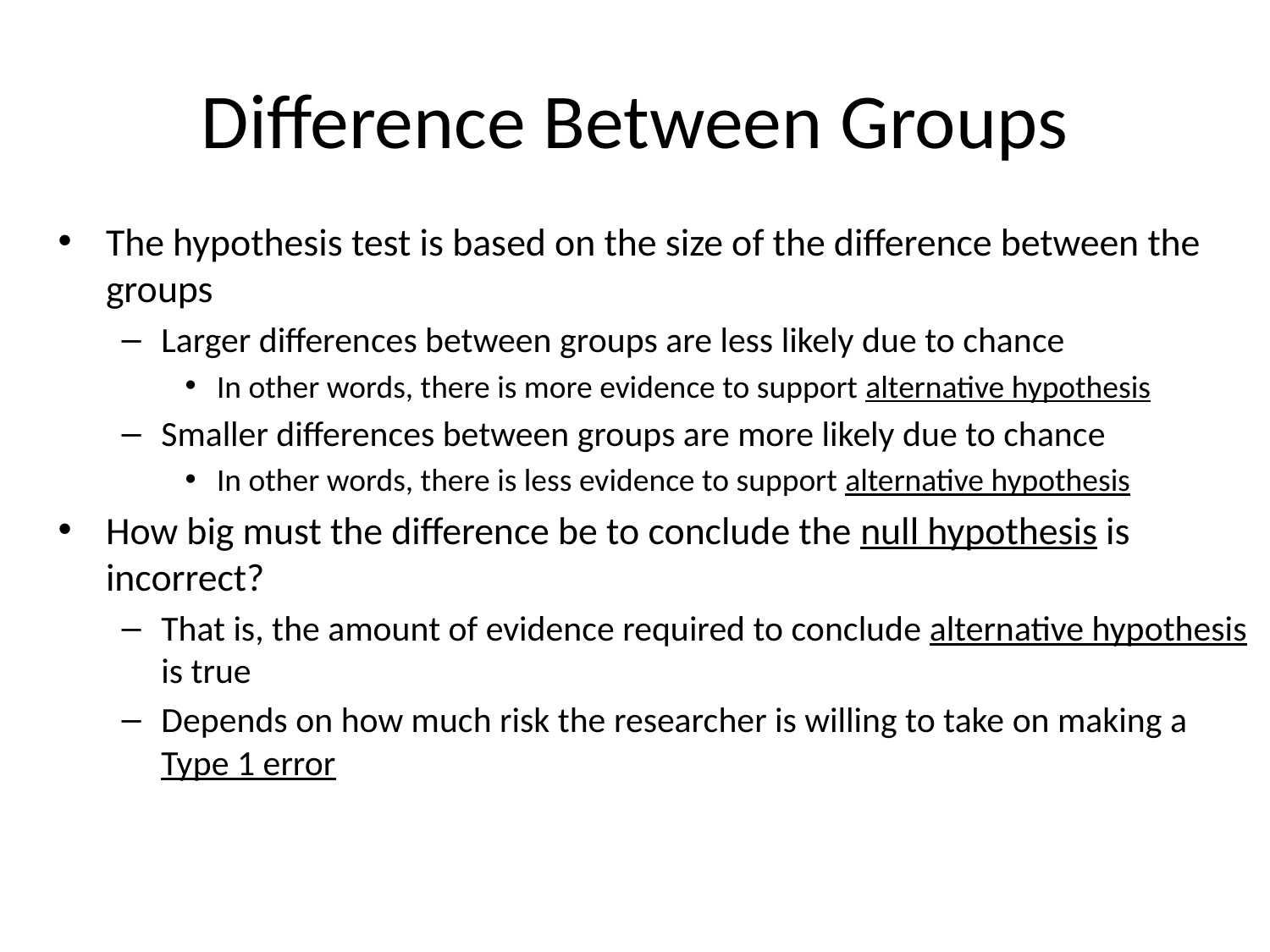

# Difference Between Groups
The hypothesis test is based on the size of the difference between the groups
Larger differences between groups are less likely due to chance
In other words, there is more evidence to support alternative hypothesis
Smaller differences between groups are more likely due to chance
In other words, there is less evidence to support alternative hypothesis
How big must the difference be to conclude the null hypothesis is incorrect?
That is, the amount of evidence required to conclude alternative hypothesis is true
Depends on how much risk the researcher is willing to take on making a Type 1 error

## Slide 8
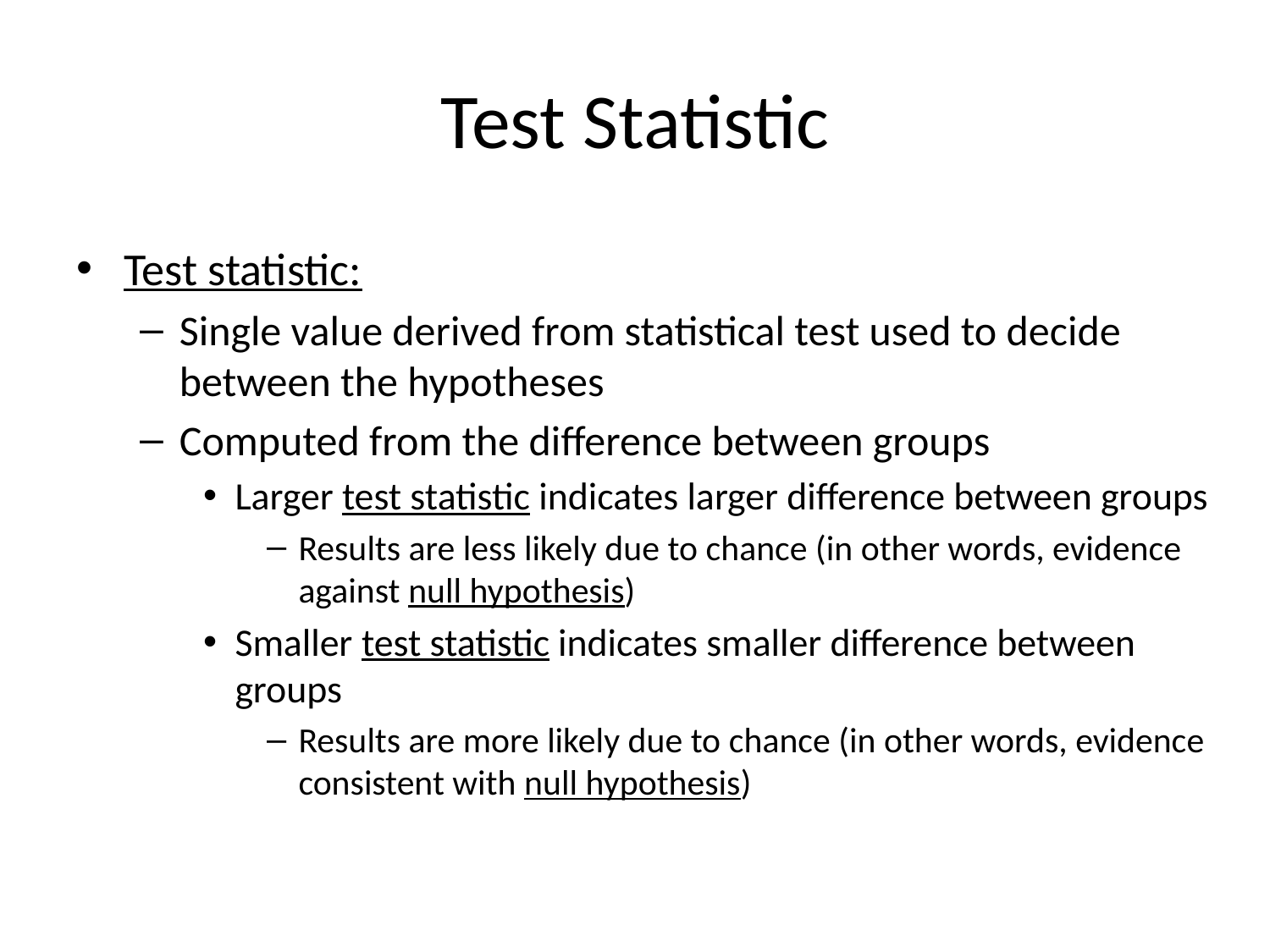

# Test Statistic
Test statistic:
Single value derived from statistical test used to decide between the hypotheses
Computed from the difference between groups
Larger test statistic indicates larger difference between groups
Results are less likely due to chance (in other words, evidence against null hypothesis)
Smaller test statistic indicates smaller difference between groups
Results are more likely due to chance (in other words, evidence consistent with null hypothesis)

## Slide 9
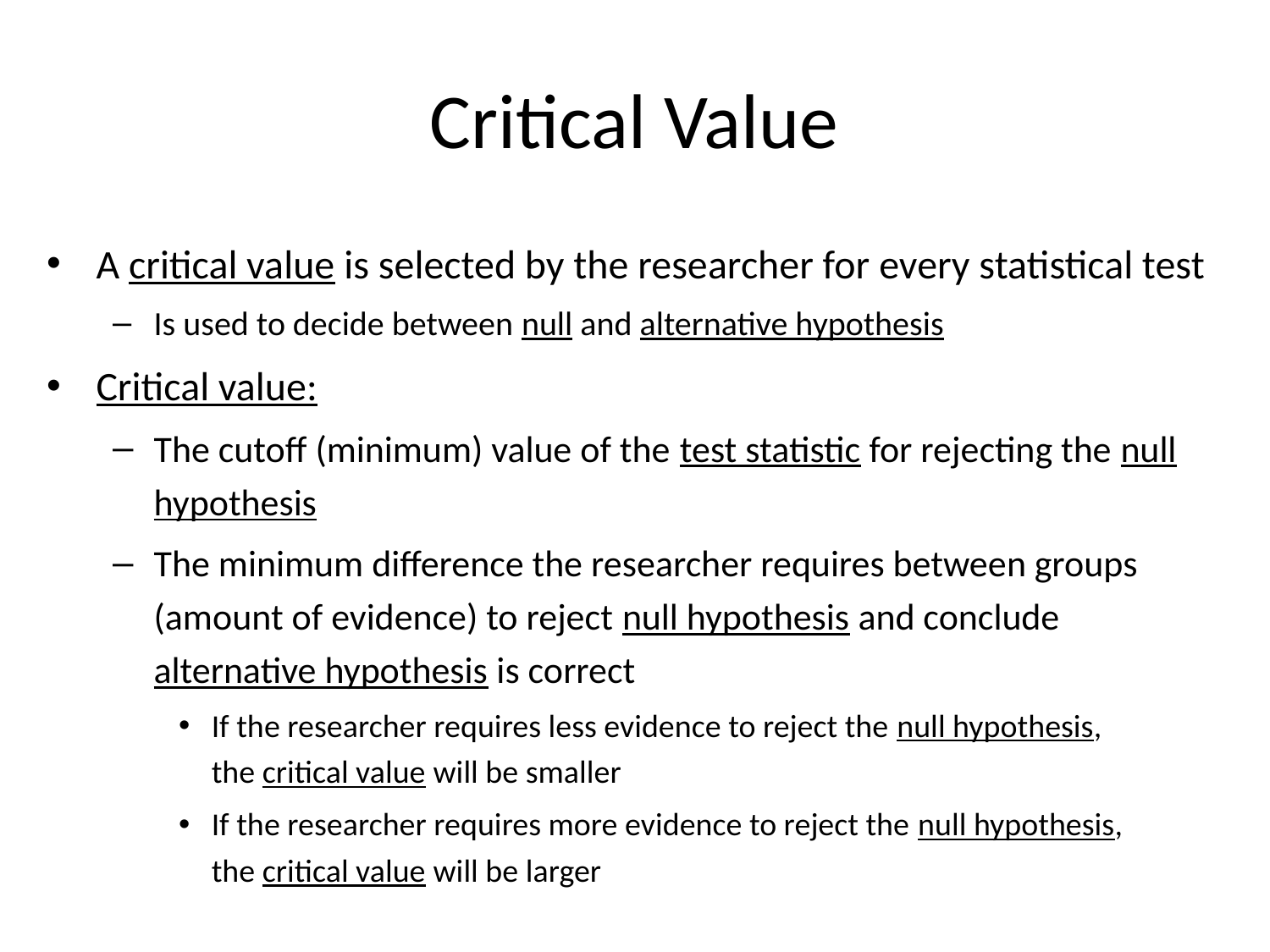

# Critical Value
A critical value is selected by the researcher for every statistical test
Is used to decide between null and alternative hypothesis
Critical value:
The cutoff (minimum) value of the test statistic for rejecting the null hypothesis
The minimum difference the researcher requires between groups (amount of evidence) to reject null hypothesis and conclude alternative hypothesis is correct
If the researcher requires less evidence to reject the null hypothesis,
the critical value will be smaller
If the researcher requires more evidence to reject the null hypothesis,
the critical value will be larger

## Slide 10
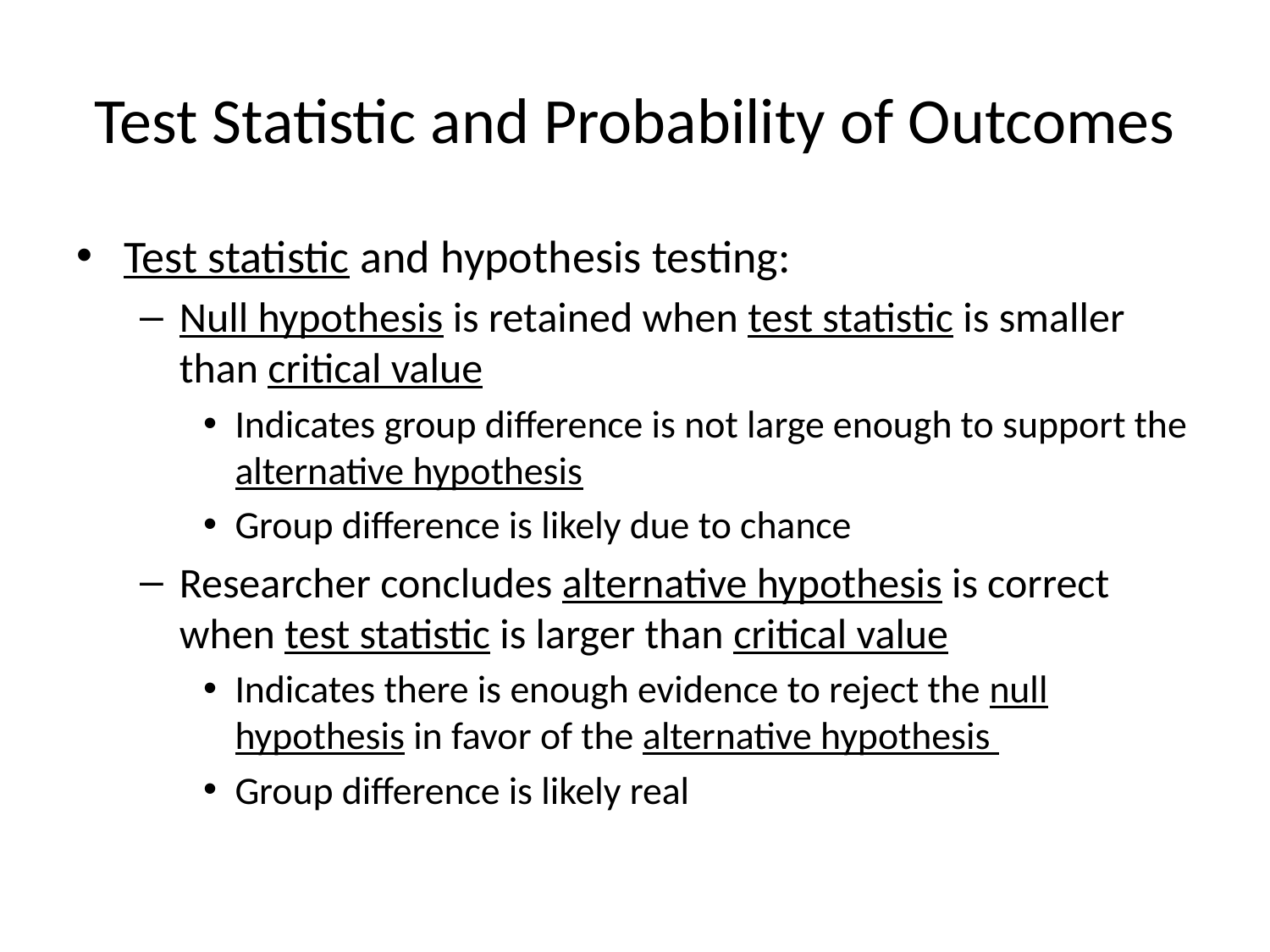

# Test Statistic and Probability of Outcomes
Test statistic and hypothesis testing:
Null hypothesis is retained when test statistic is smaller than critical value
Indicates group difference is not large enough to support the alternative hypothesis
Group difference is likely due to chance
Researcher concludes alternative hypothesis is correct when test statistic is larger than critical value
Indicates there is enough evidence to reject the null hypothesis in favor of the alternative hypothesis
Group difference is likely real

## Slide 11
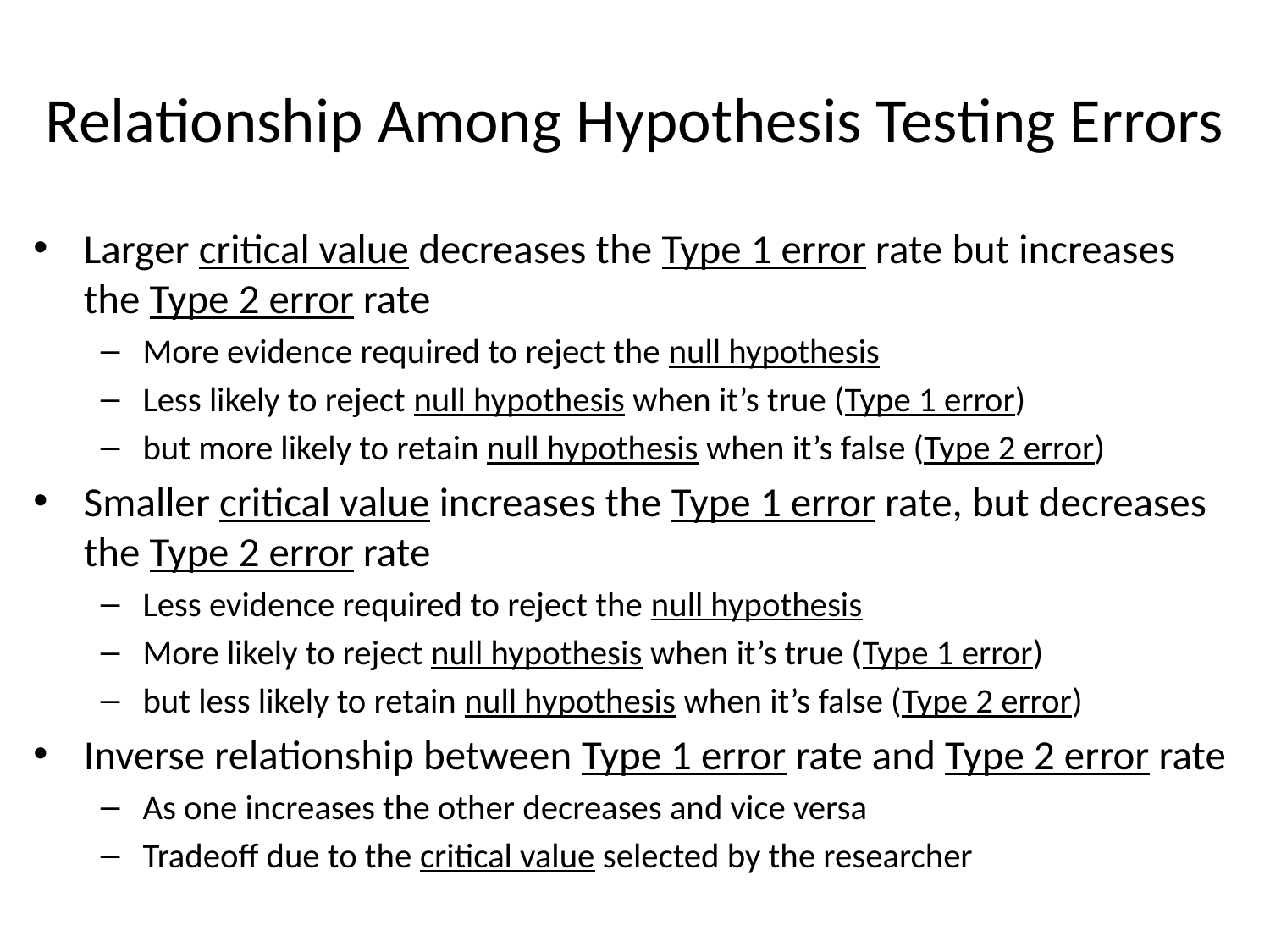

# Relationship Among Hypothesis Testing Errors
Larger critical value decreases the Type 1 error rate but increases the Type 2 error rate
More evidence required to reject the null hypothesis
Less likely to reject null hypothesis when it’s true (Type 1 error)
but more likely to retain null hypothesis when it’s false (Type 2 error)
Smaller critical value increases the Type 1 error rate, but decreases the Type 2 error rate
Less evidence required to reject the null hypothesis
More likely to reject null hypothesis when it’s true (Type 1 error)
but less likely to retain null hypothesis when it’s false (Type 2 error)
Inverse relationship between Type 1 error rate and Type 2 error rate
As one increases the other decreases and vice versa
Tradeoff due to the critical value selected by the researcher

## Slide 12
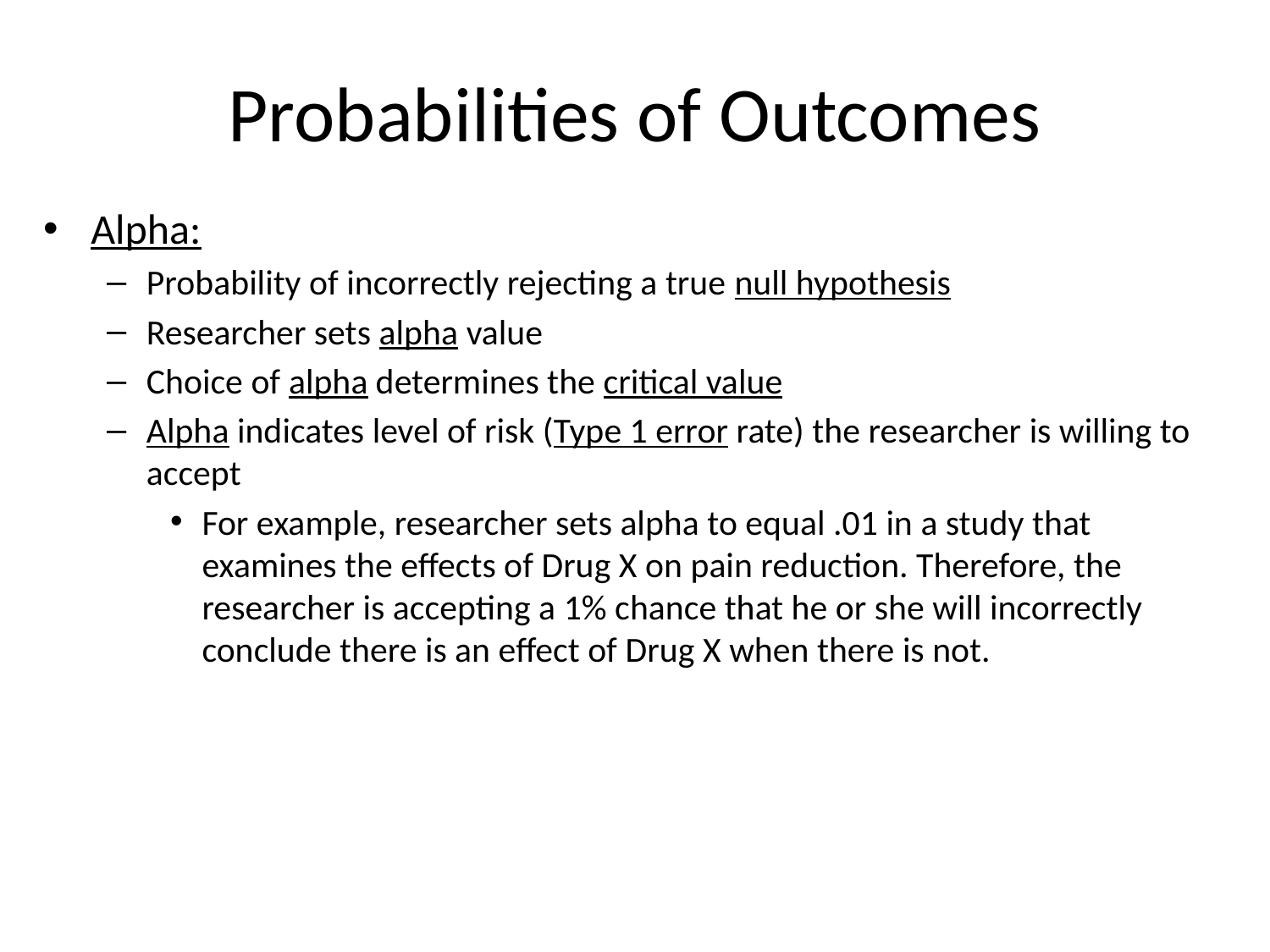

# Probabilities of Outcomes
Alpha:
Probability of incorrectly rejecting a true null hypothesis
Researcher sets alpha value
Choice of alpha determines the critical value
Alpha indicates level of risk (Type 1 error rate) the researcher is willing to accept
For example, researcher sets alpha to equal .01 in a study that examines the effects of Drug X on pain reduction. Therefore, the researcher is accepting a 1% chance that he or she will incorrectly conclude there is an effect of Drug X when there is not.

## Slide 13
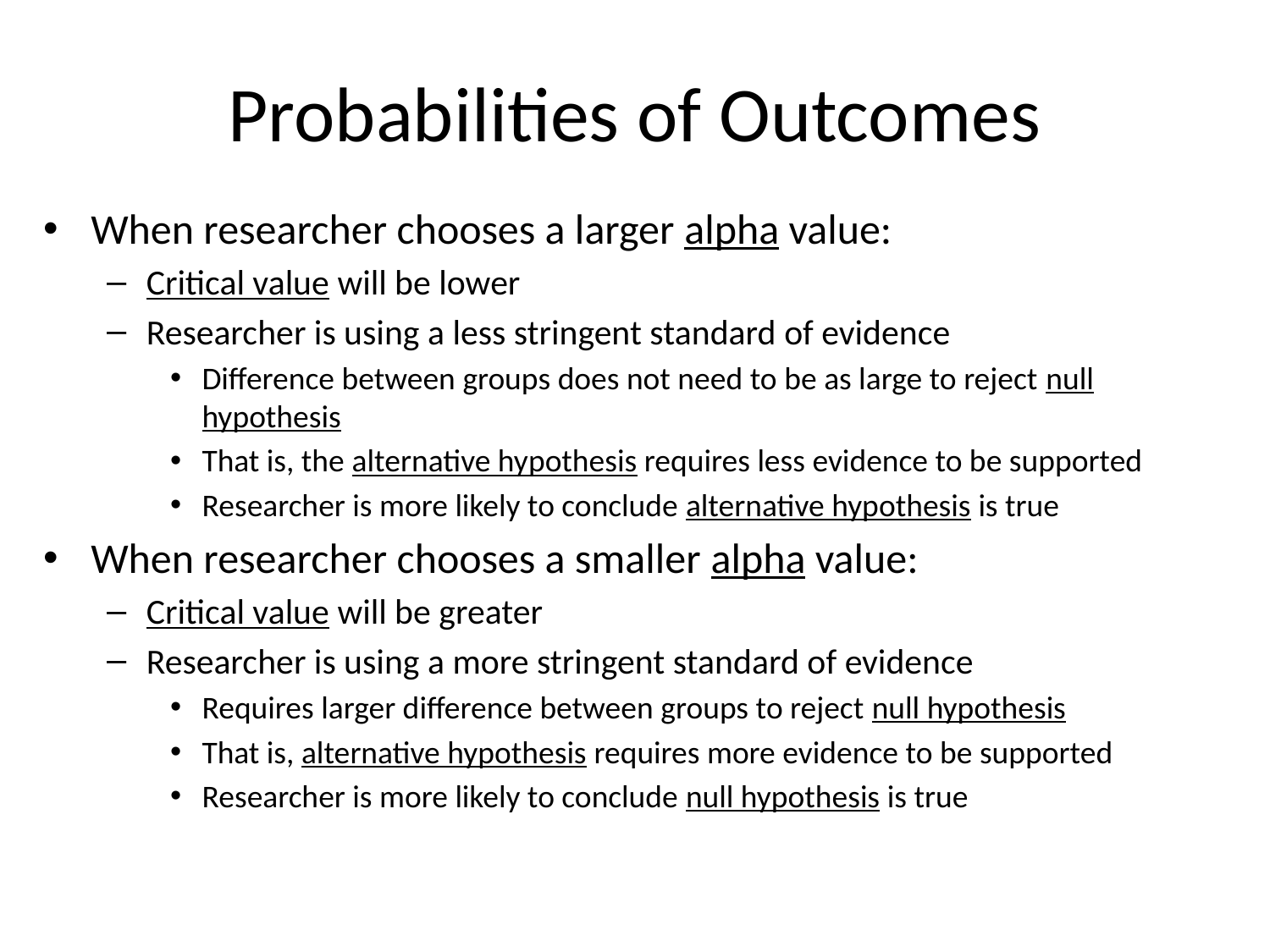

# Probabilities of Outcomes
When researcher chooses a larger alpha value:
Critical value will be lower
Researcher is using a less stringent standard of evidence
Difference between groups does not need to be as large to reject null hypothesis
That is, the alternative hypothesis requires less evidence to be supported
Researcher is more likely to conclude alternative hypothesis is true
When researcher chooses a smaller alpha value:
Critical value will be greater
Researcher is using a more stringent standard of evidence
Requires larger difference between groups to reject null hypothesis
That is, alternative hypothesis requires more evidence to be supported
Researcher is more likely to conclude null hypothesis is true

## Slide 14
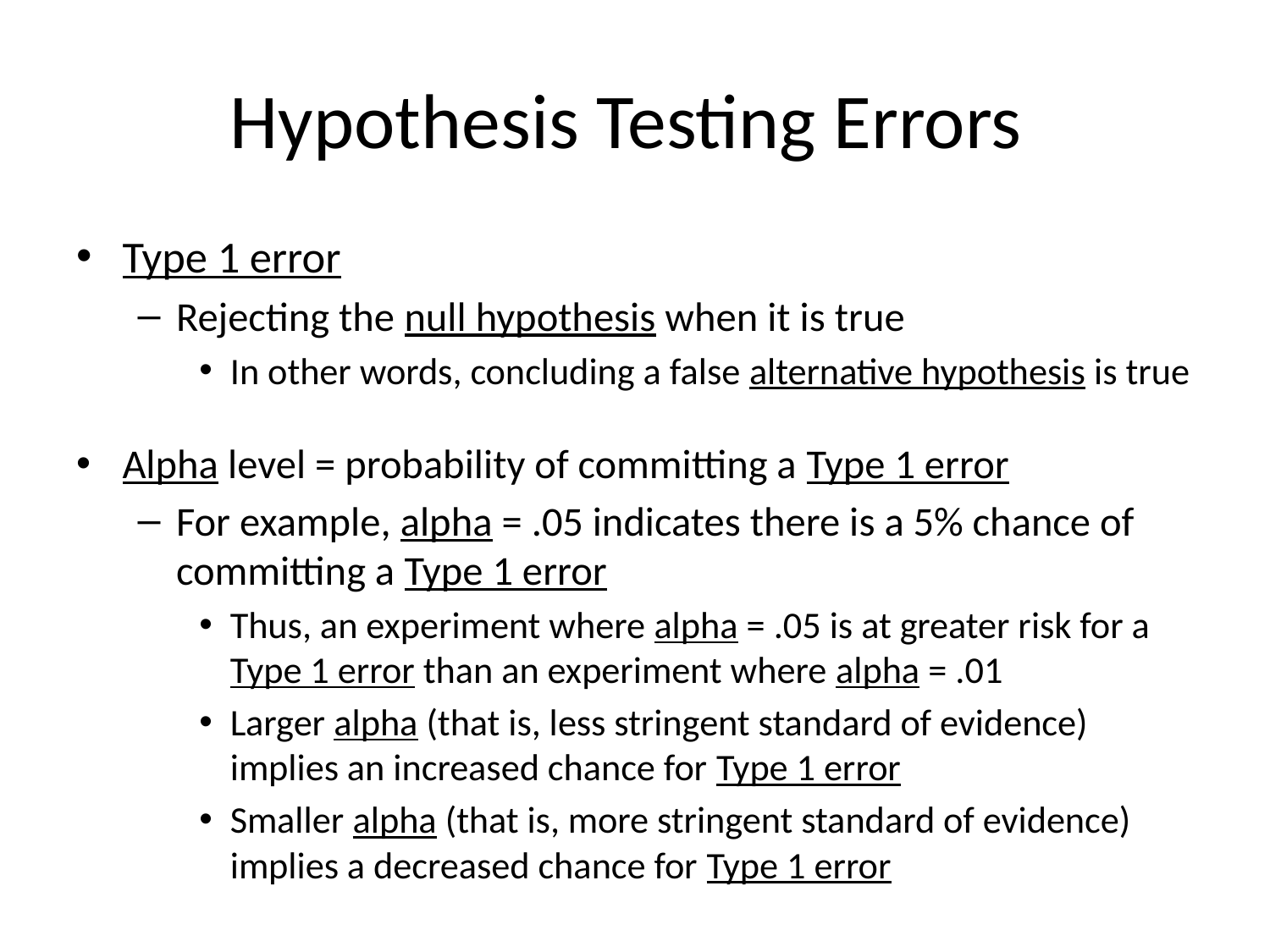

# Hypothesis Testing Errors
Type 1 error
Rejecting the null hypothesis when it is true
In other words, concluding a false alternative hypothesis is true
Alpha level = probability of committing a Type 1 error
For example, alpha = .05 indicates there is a 5% chance of committing a Type 1 error
Thus, an experiment where alpha = .05 is at greater risk for a Type 1 error than an experiment where alpha = .01
Larger alpha (that is, less stringent standard of evidence) implies an increased chance for Type 1 error
Smaller alpha (that is, more stringent standard of evidence) implies a decreased chance for Type 1 error

## Slide 15
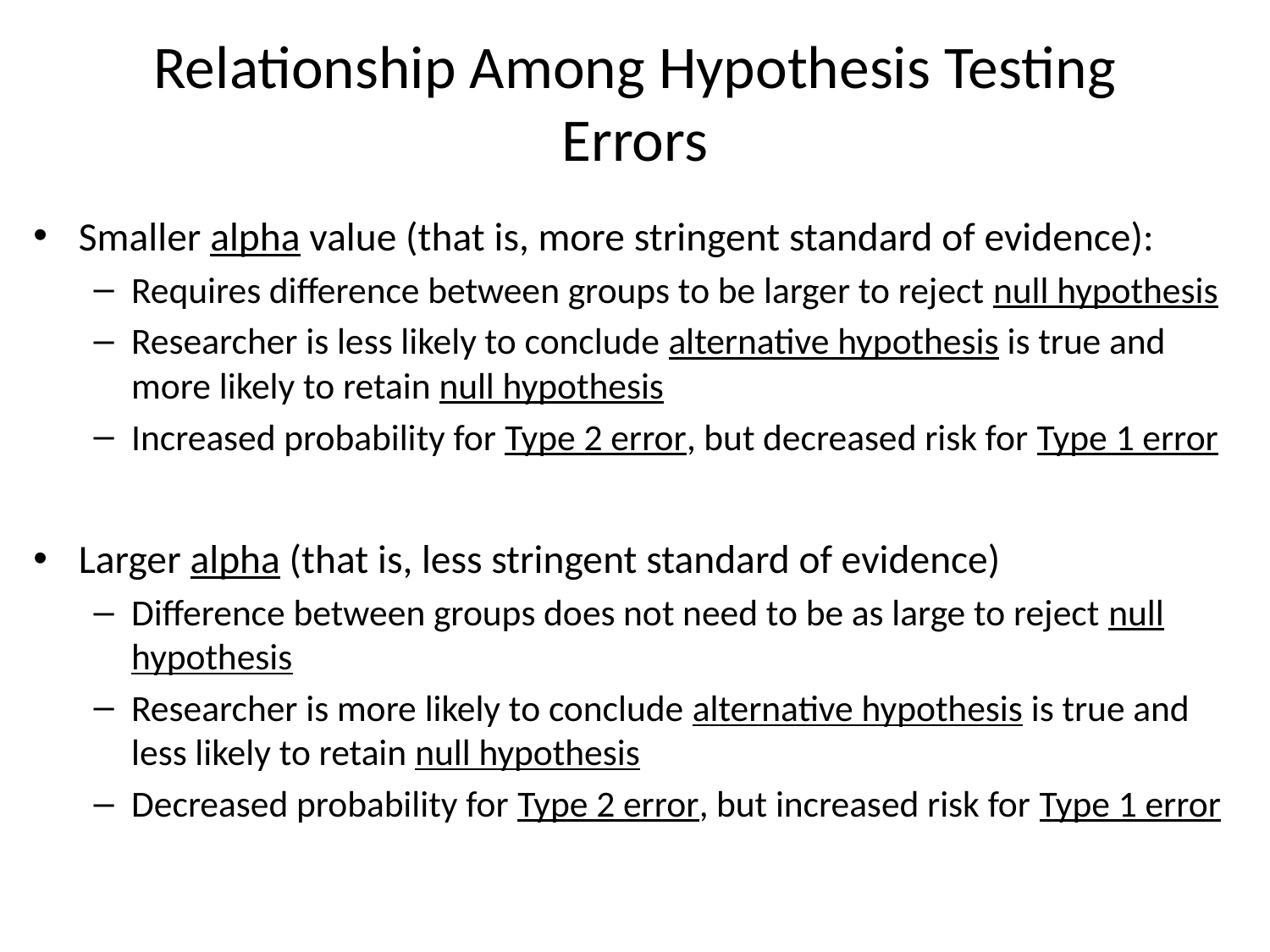

# Relationship Among Hypothesis Testing Errors
Smaller alpha value (that is, more stringent standard of evidence):
Requires difference between groups to be larger to reject null hypothesis
Researcher is less likely to conclude alternative hypothesis is true and more likely to retain null hypothesis
Increased probability for Type 2 error, but decreased risk for Type 1 error
Larger alpha (that is, less stringent standard of evidence)
Difference between groups does not need to be as large to reject null hypothesis
Researcher is more likely to conclude alternative hypothesis is true and less likely to retain null hypothesis
Decreased probability for Type 2 error, but increased risk for Type 1 error

## Slide 16
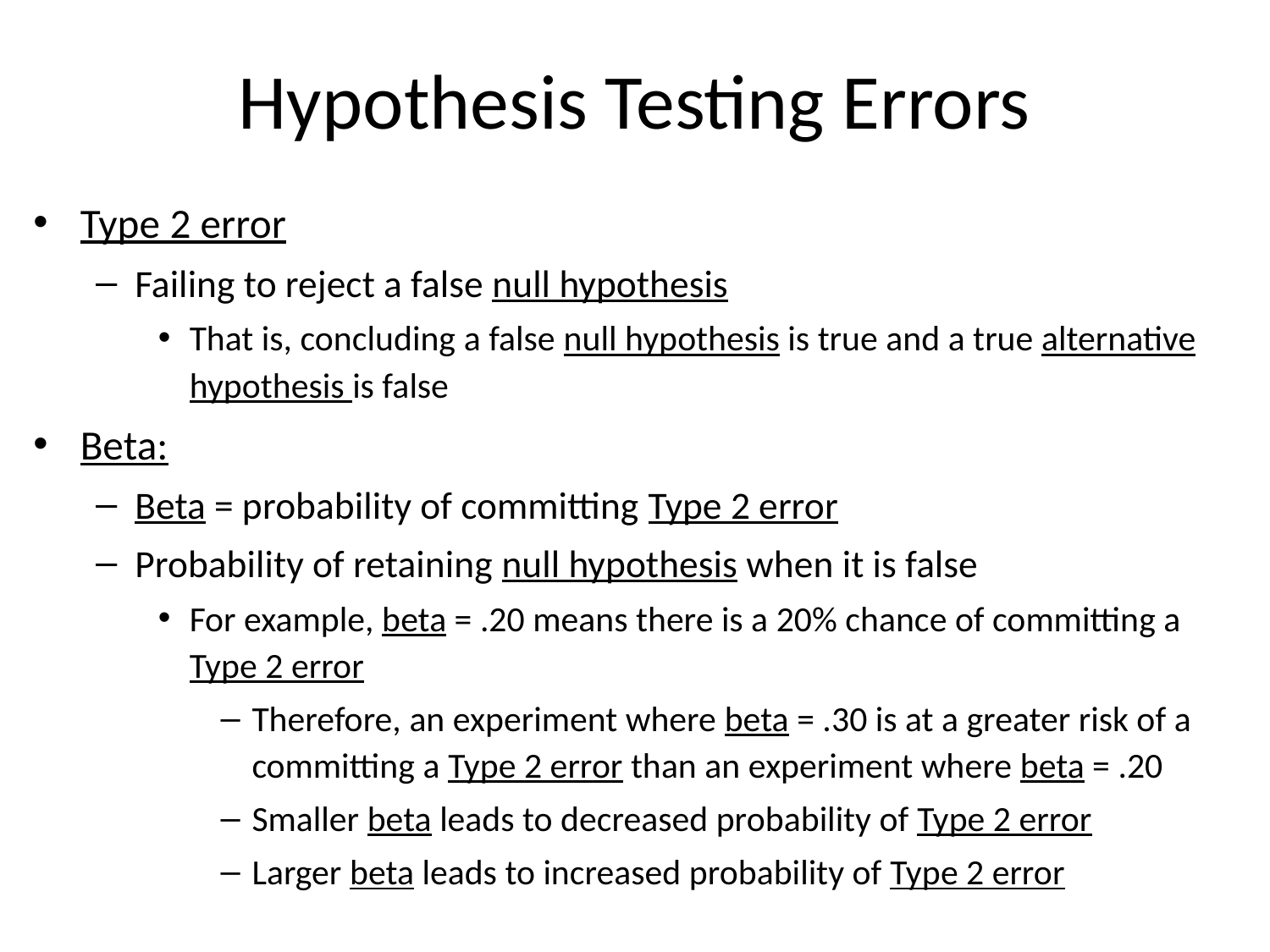

# Hypothesis Testing Errors
Type 2 error
Failing to reject a false null hypothesis
That is, concluding a false null hypothesis is true and a true alternative hypothesis is false
Beta:
Beta = probability of committing Type 2 error
Probability of retaining null hypothesis when it is false
For example, beta = .20 means there is a 20% chance of committing a Type 2 error
Therefore, an experiment where beta = .30 is at a greater risk of a committing a Type 2 error than an experiment where beta = .20
Smaller beta leads to decreased probability of Type 2 error
Larger beta leads to increased probability of Type 2 error

## Slide 17
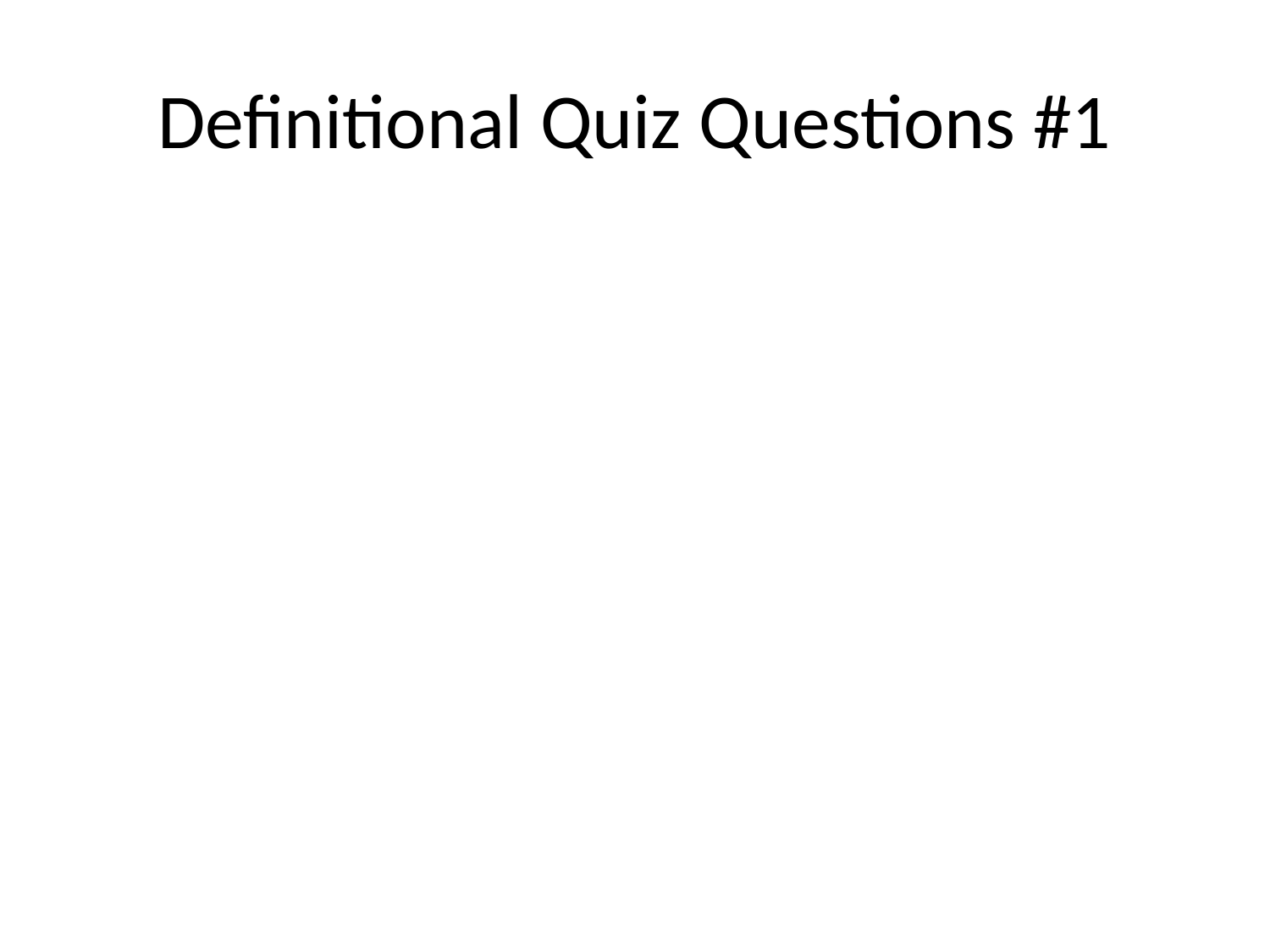

# Definitional Quiz Questions #1

## Slide 18
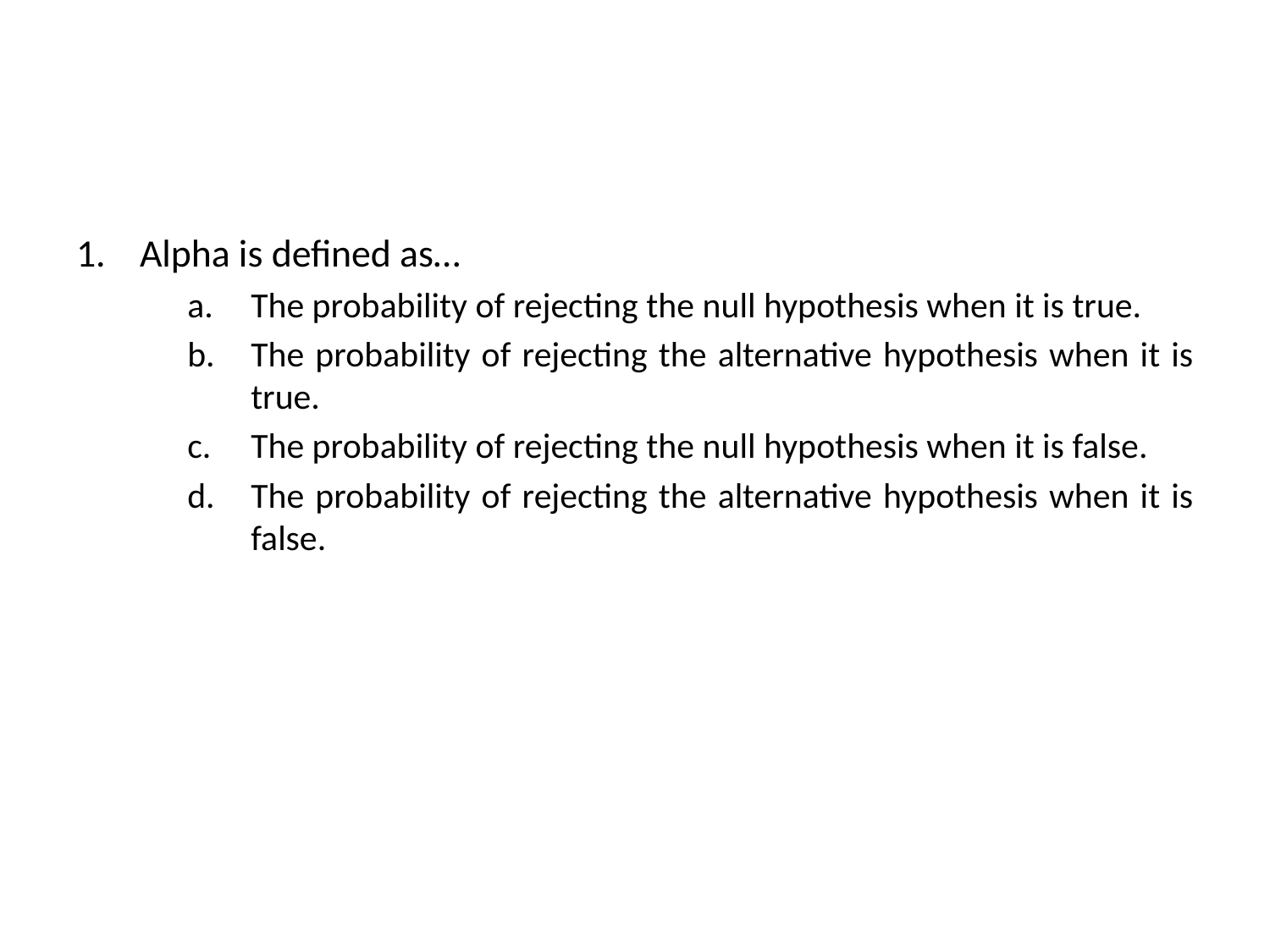

#
Alpha is defined as…
The probability of rejecting the null hypothesis when it is true.
The probability of rejecting the alternative hypothesis when it is true.
The probability of rejecting the null hypothesis when it is false.
The probability of rejecting the alternative hypothesis when it is false.

## Slide 19
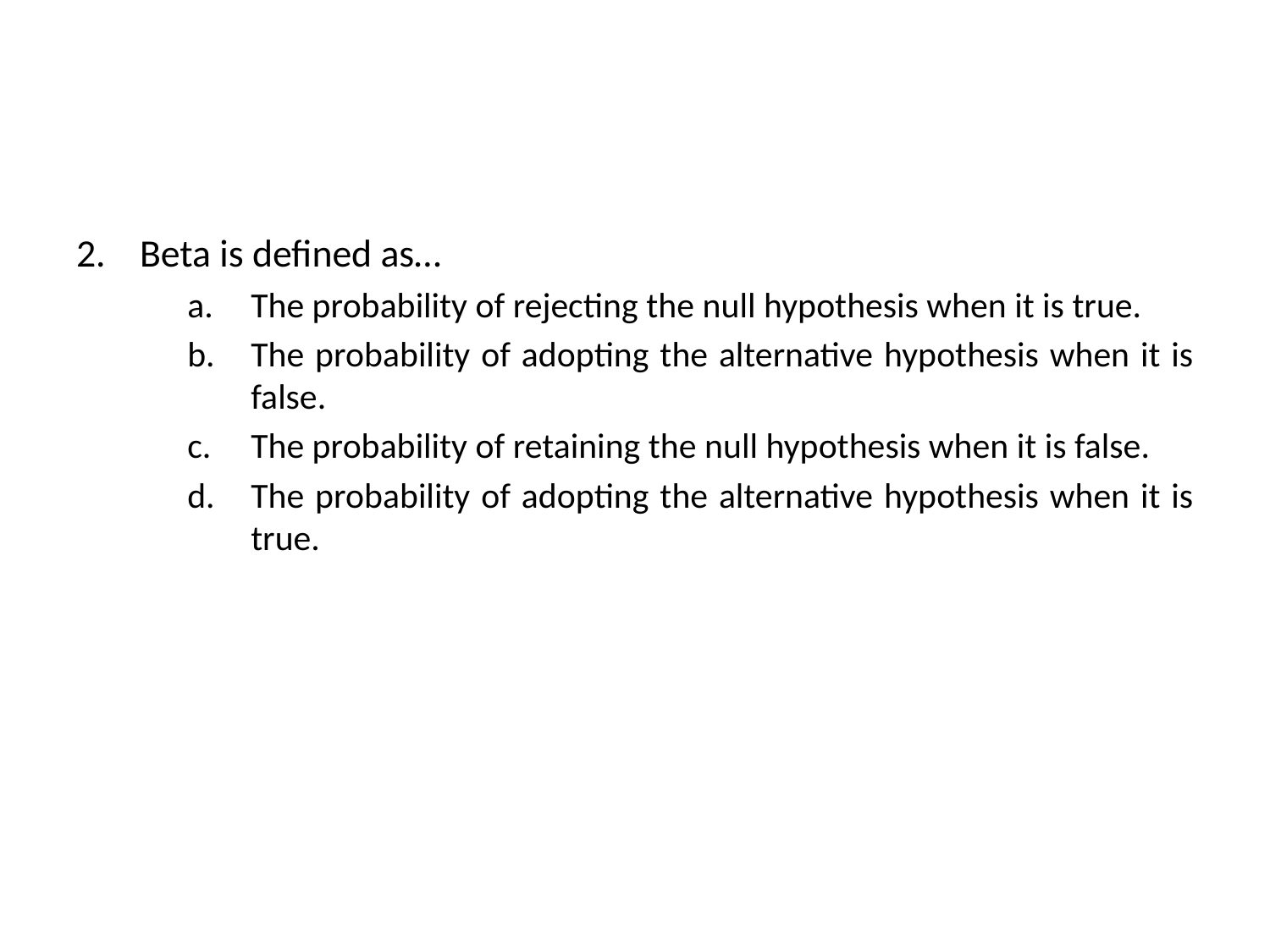

#
Beta is defined as…
The probability of rejecting the null hypothesis when it is true.
The probability of adopting the alternative hypothesis when it is false.
The probability of retaining the null hypothesis when it is false.
The probability of adopting the alternative hypothesis when it is true.

## Slide 20
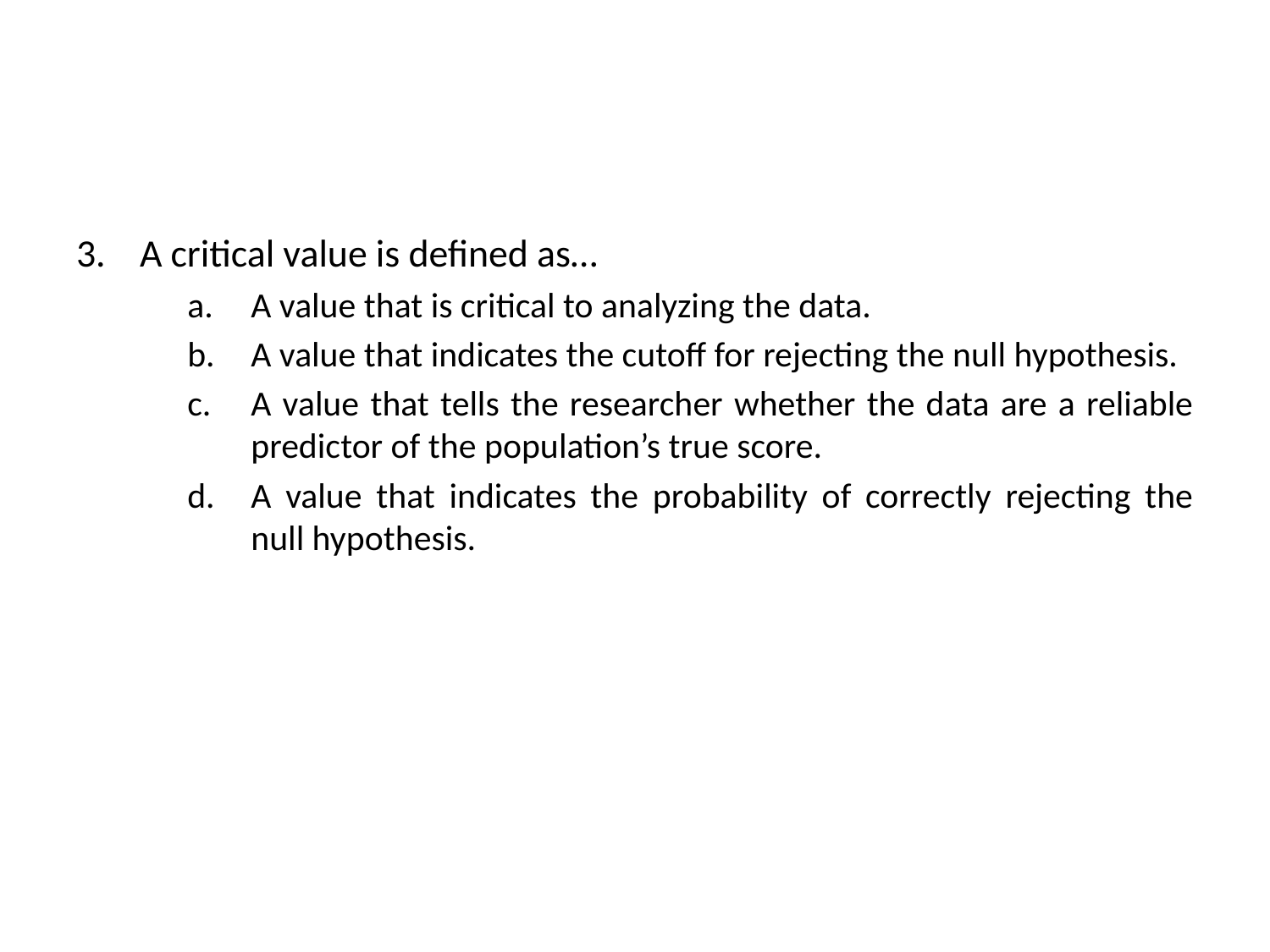

#
A critical value is defined as…
A value that is critical to analyzing the data.
A value that indicates the cutoff for rejecting the null hypothesis.
A value that tells the researcher whether the data are a reliable predictor of the population’s true score.
A value that indicates the probability of correctly rejecting the null hypothesis.

## Slide 21
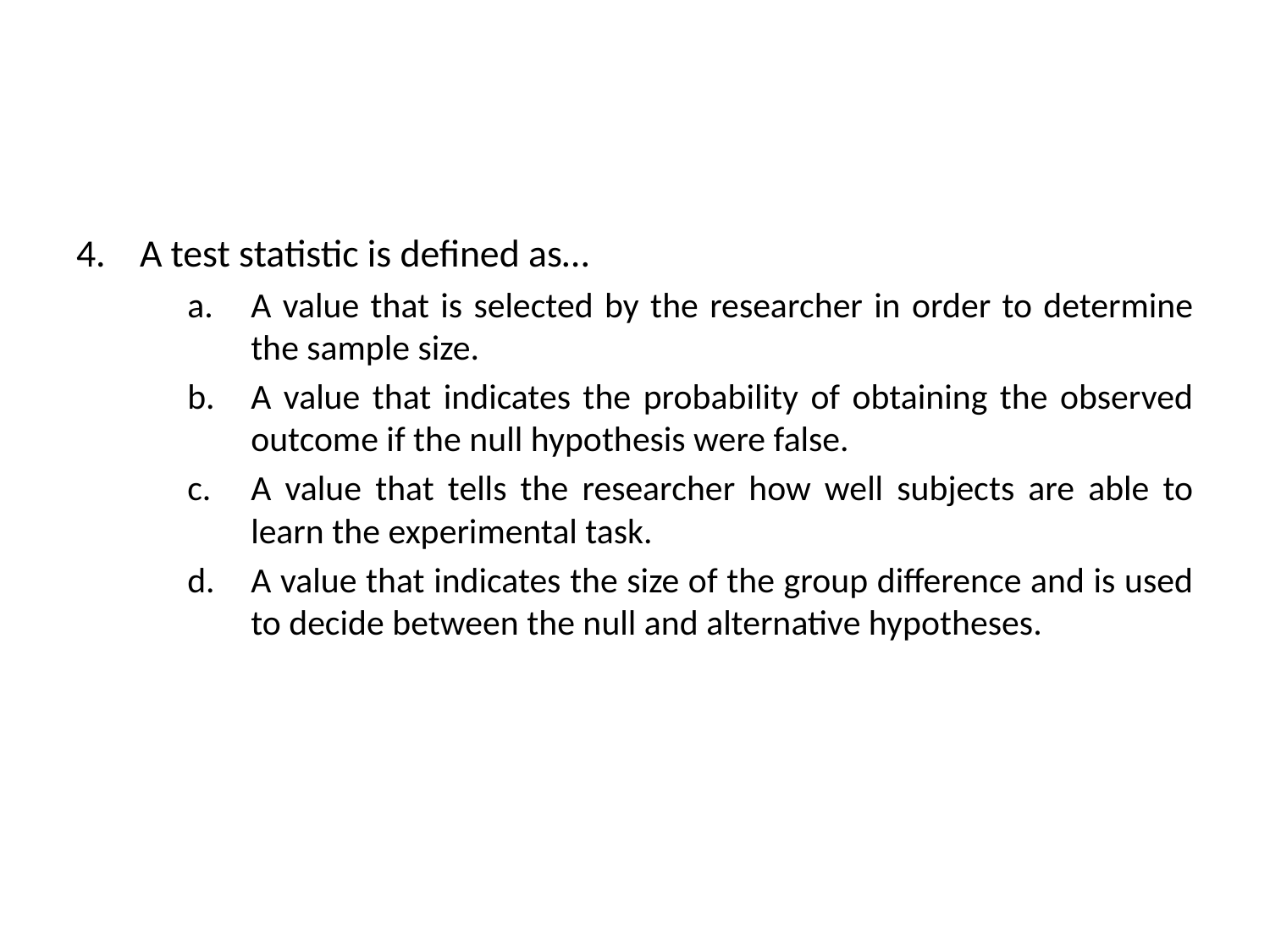

#
A test statistic is defined as…
A value that is selected by the researcher in order to determine the sample size.
A value that indicates the probability of obtaining the observed outcome if the null hypothesis were false.
A value that tells the researcher how well subjects are able to learn the experimental task.
A value that indicates the size of the group difference and is used to decide between the null and alternative hypotheses.

## Slide 22
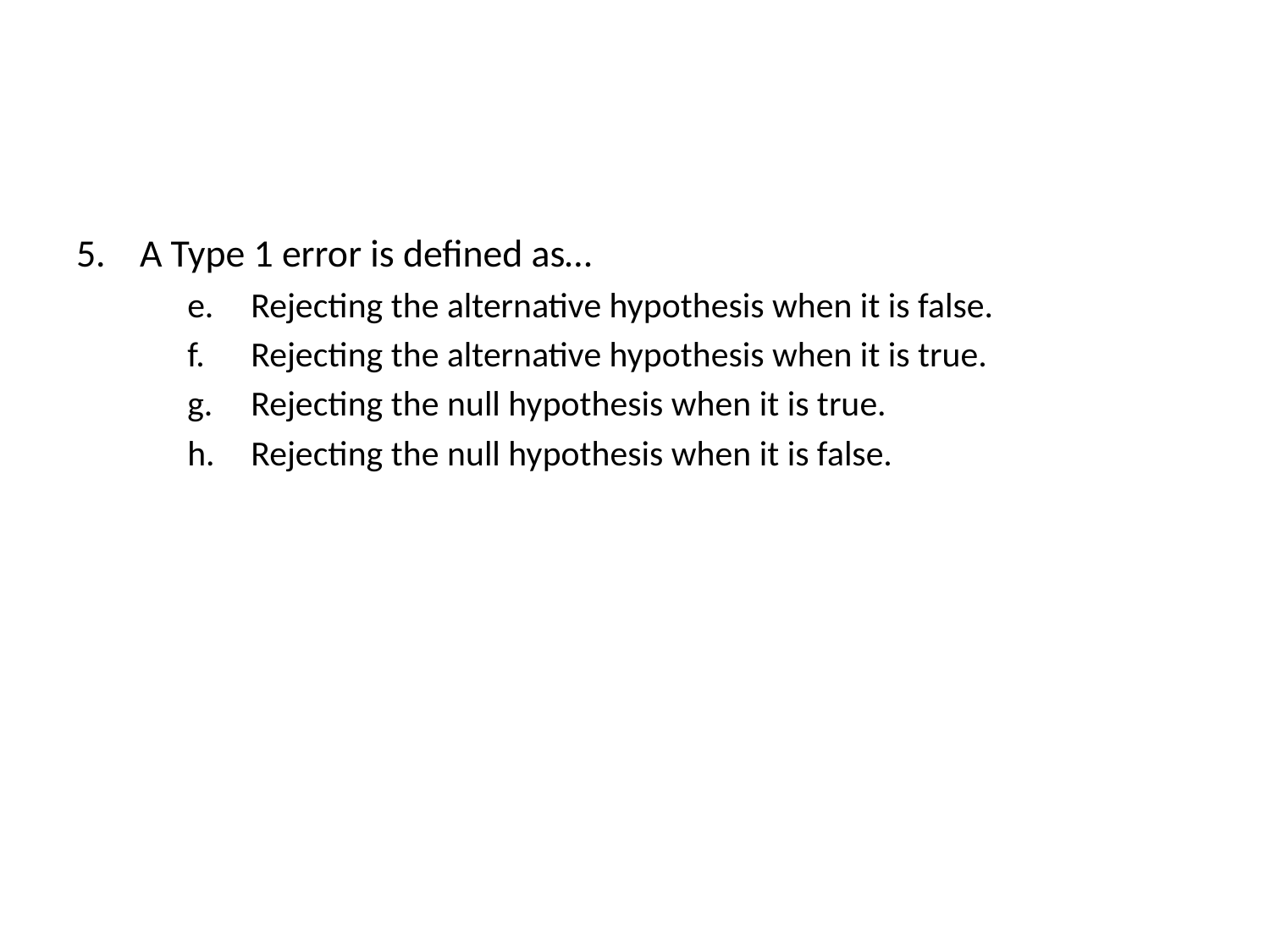

#
A Type 1 error is defined as…
Rejecting the alternative hypothesis when it is false.
Rejecting the alternative hypothesis when it is true.
Rejecting the null hypothesis when it is true.
Rejecting the null hypothesis when it is false.

## Slide 23
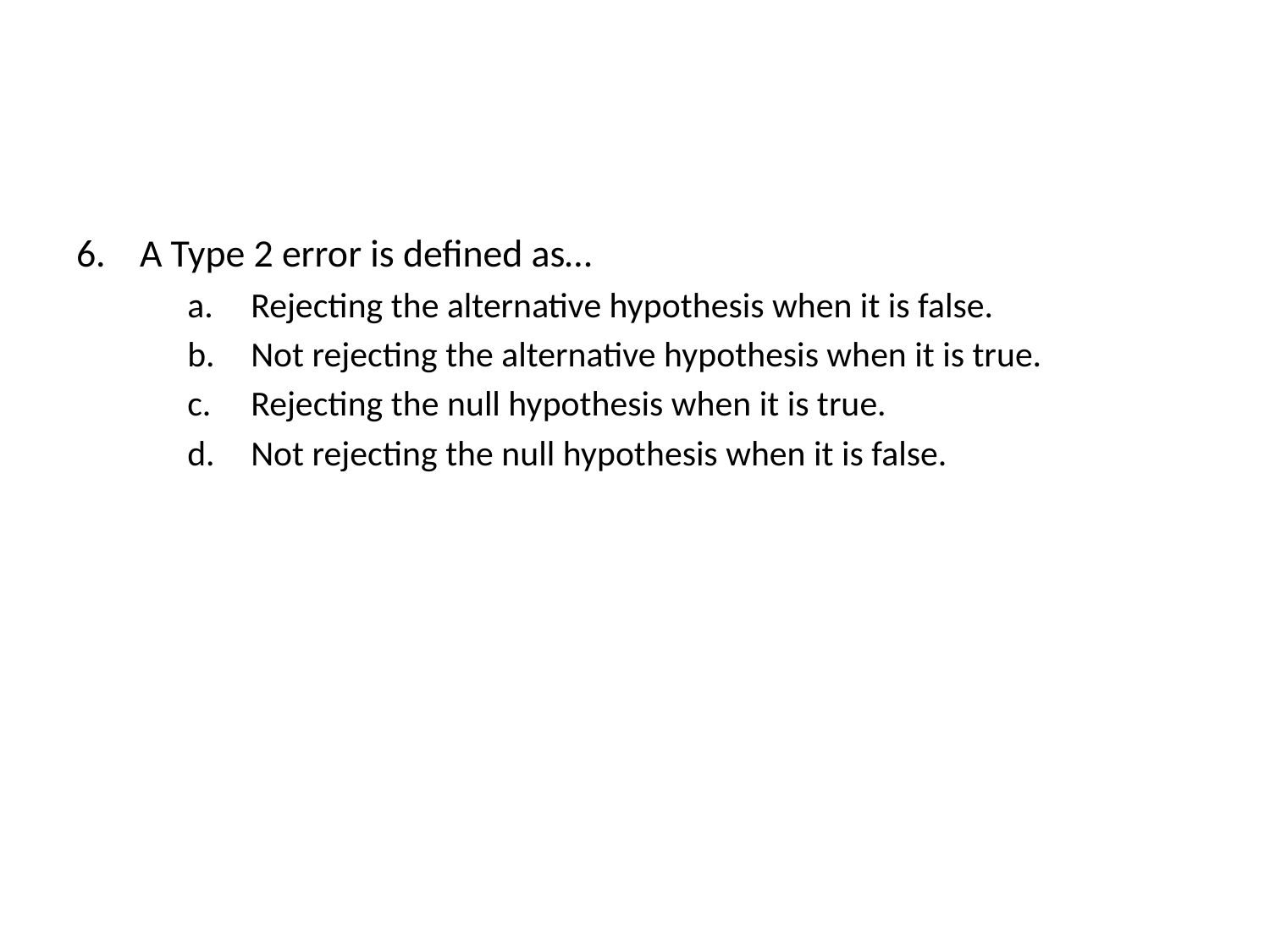

#
A Type 2 error is defined as…
Rejecting the alternative hypothesis when it is false.
Not rejecting the alternative hypothesis when it is true.
Rejecting the null hypothesis when it is true.
Not rejecting the null hypothesis when it is false.

## Slide 24
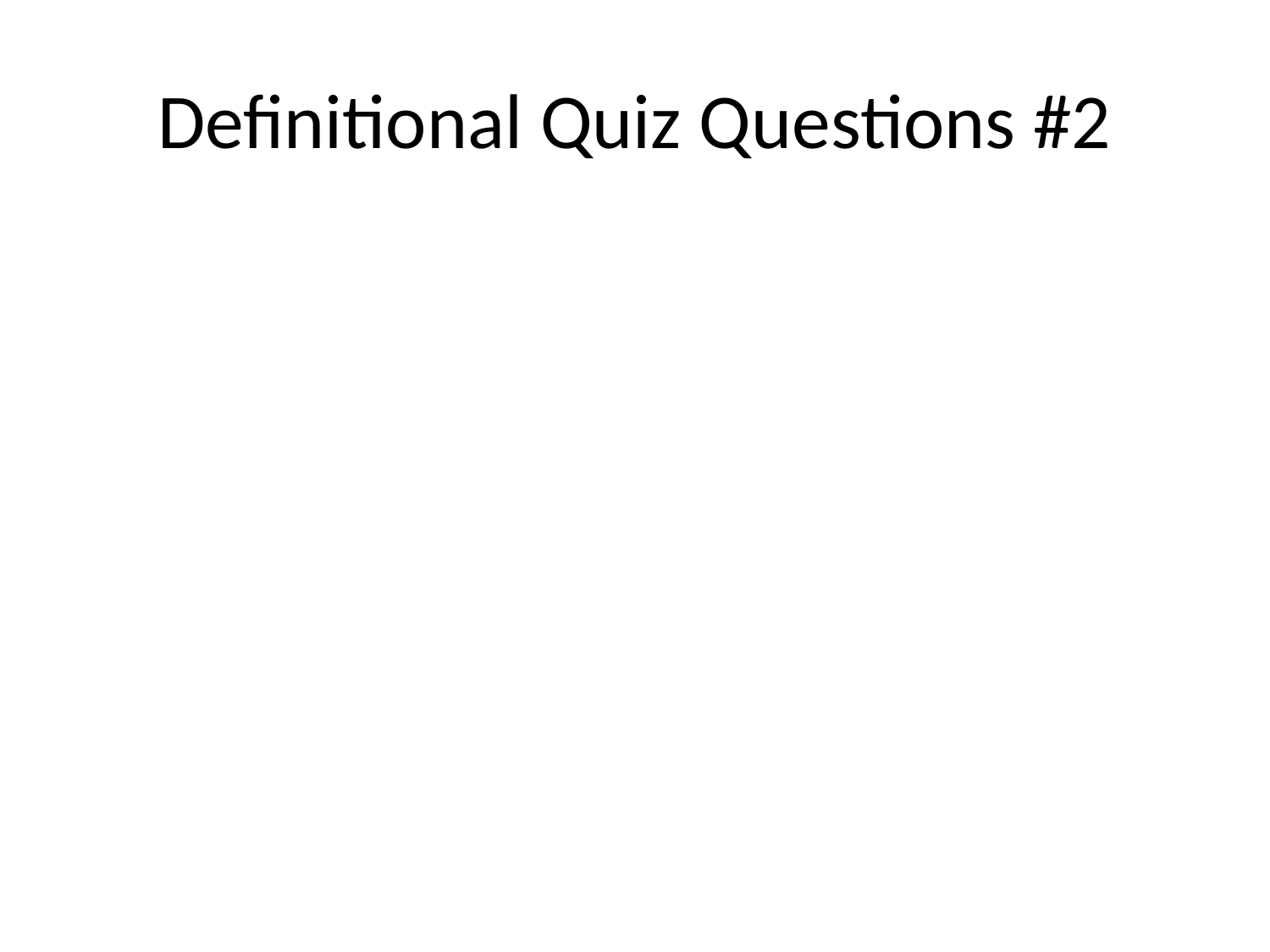

# Definitional Quiz Questions #2

## Slide 25
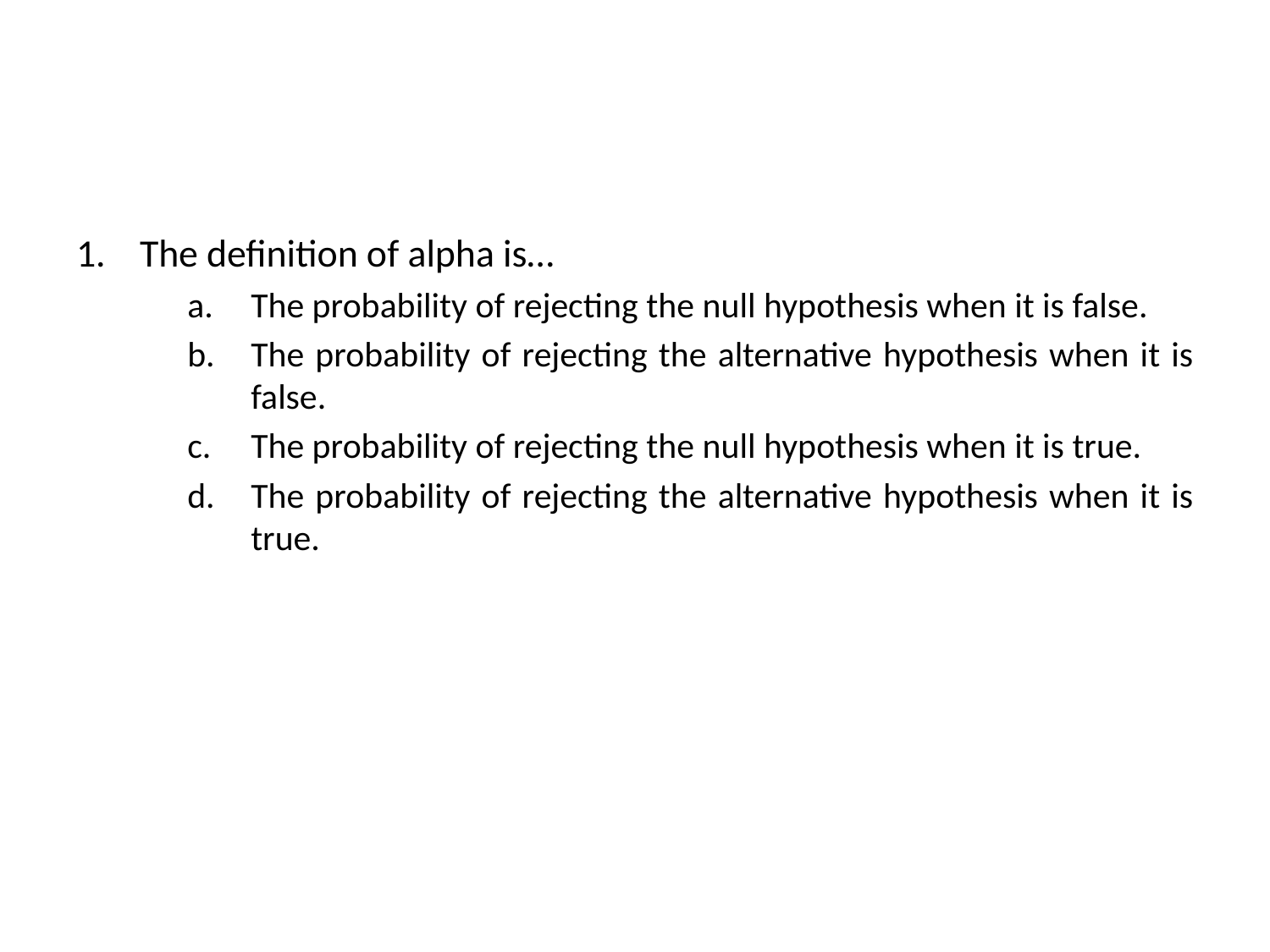

#
The definition of alpha is…
The probability of rejecting the null hypothesis when it is false.
The probability of rejecting the alternative hypothesis when it is false.
The probability of rejecting the null hypothesis when it is true.
The probability of rejecting the alternative hypothesis when it is true.

## Slide 26
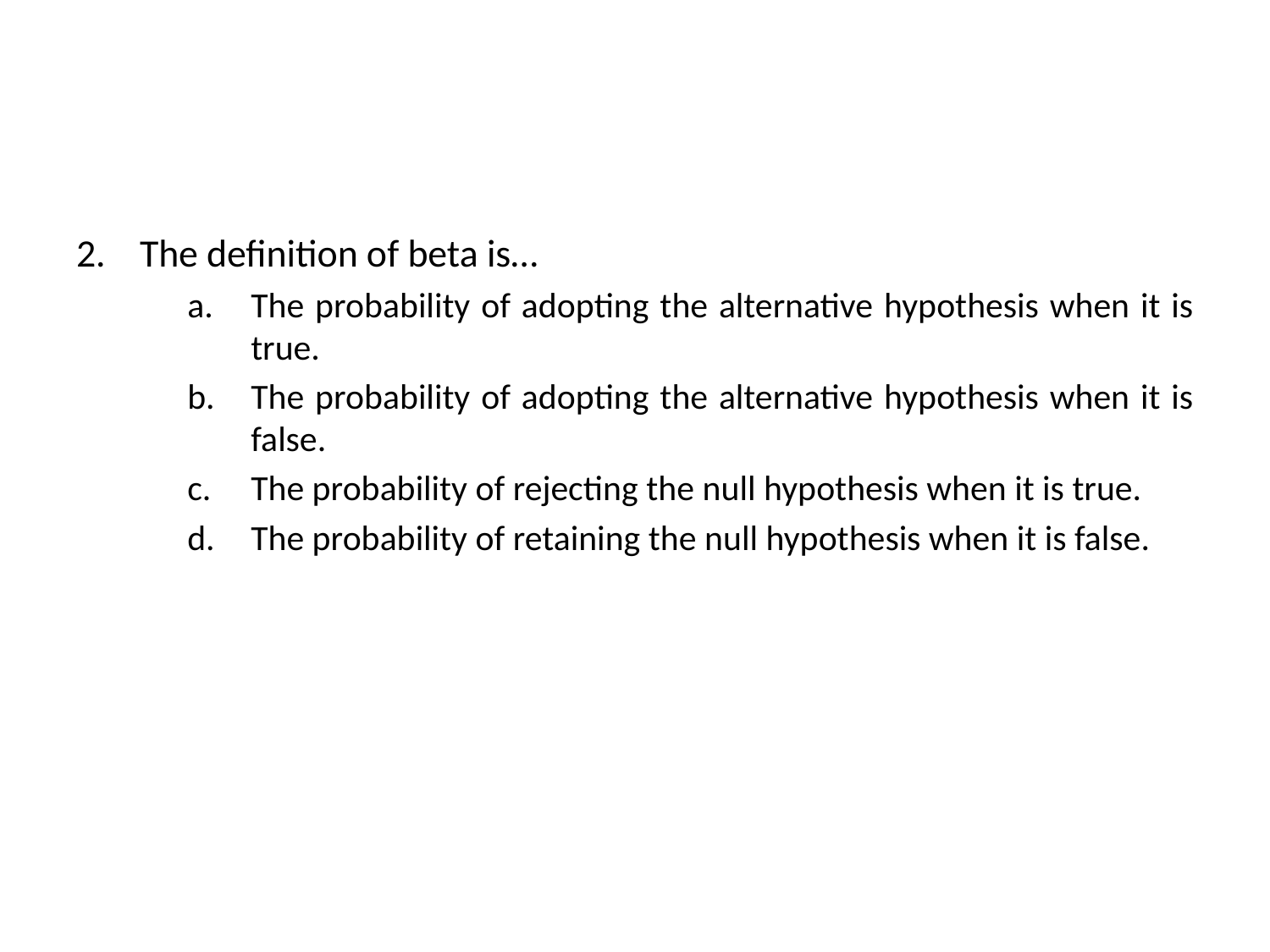

#
The definition of beta is…
The probability of adopting the alternative hypothesis when it is true.
The probability of adopting the alternative hypothesis when it is false.
The probability of rejecting the null hypothesis when it is true.
The probability of retaining the null hypothesis when it is false.

## Slide 27
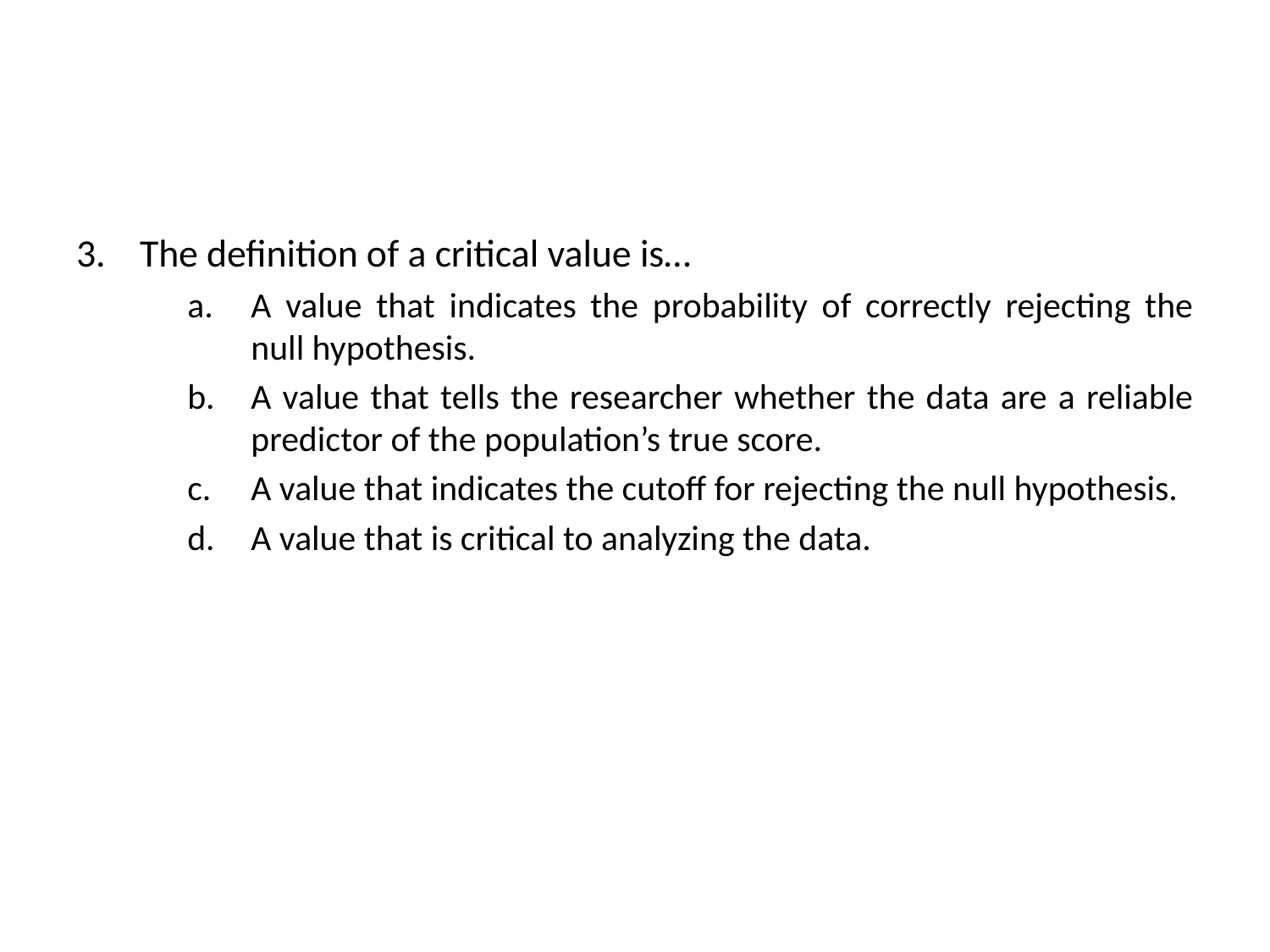

#
The definition of a critical value is…
A value that indicates the probability of correctly rejecting the null hypothesis.
A value that tells the researcher whether the data are a reliable predictor of the population’s true score.
A value that indicates the cutoff for rejecting the null hypothesis.
A value that is critical to analyzing the data.

## Slide 28
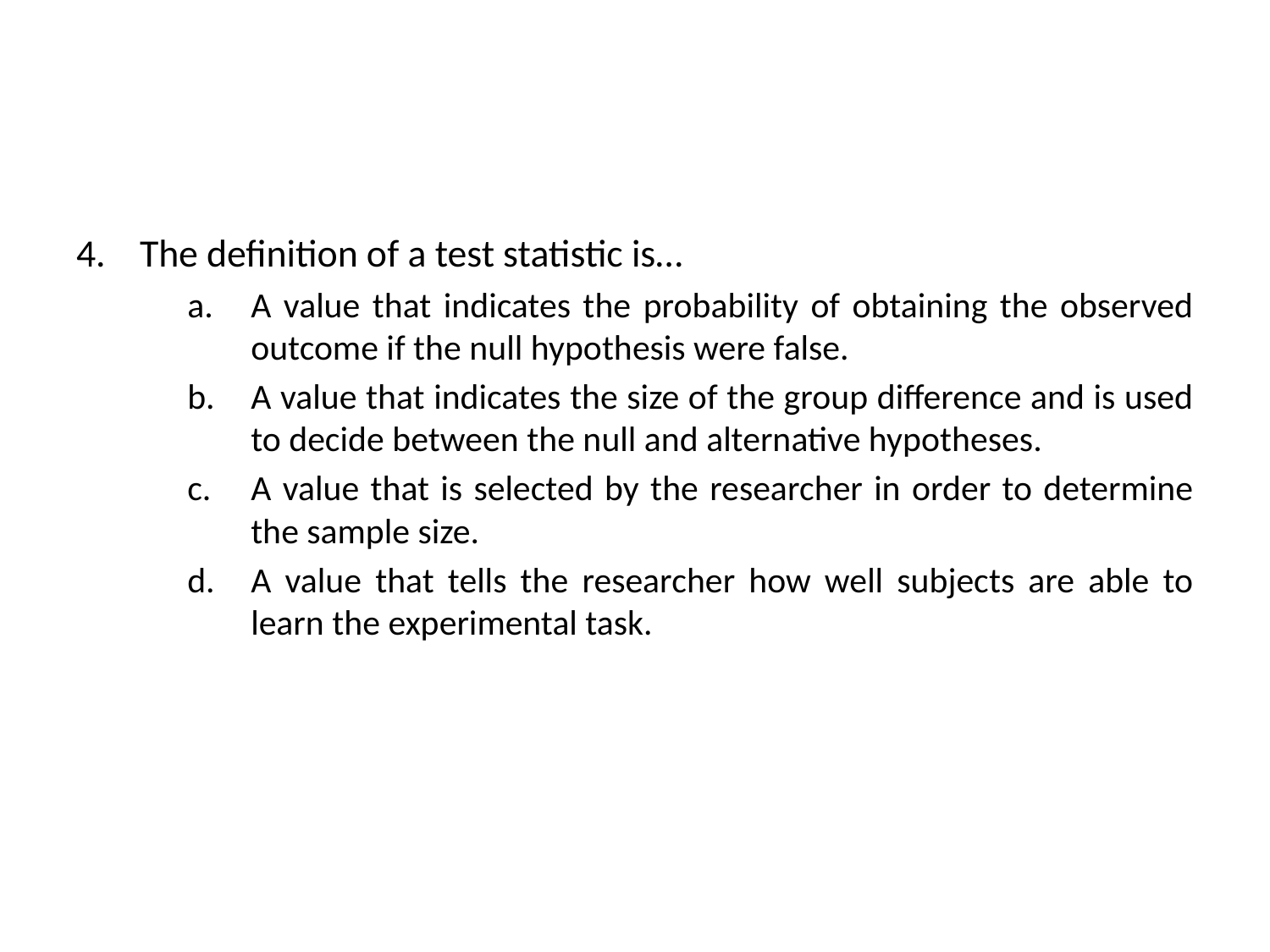

#
The definition of a test statistic is…
A value that indicates the probability of obtaining the observed outcome if the null hypothesis were false.
A value that indicates the size of the group difference and is used to decide between the null and alternative hypotheses.
A value that is selected by the researcher in order to determine the sample size.
A value that tells the researcher how well subjects are able to learn the experimental task.

## Slide 29
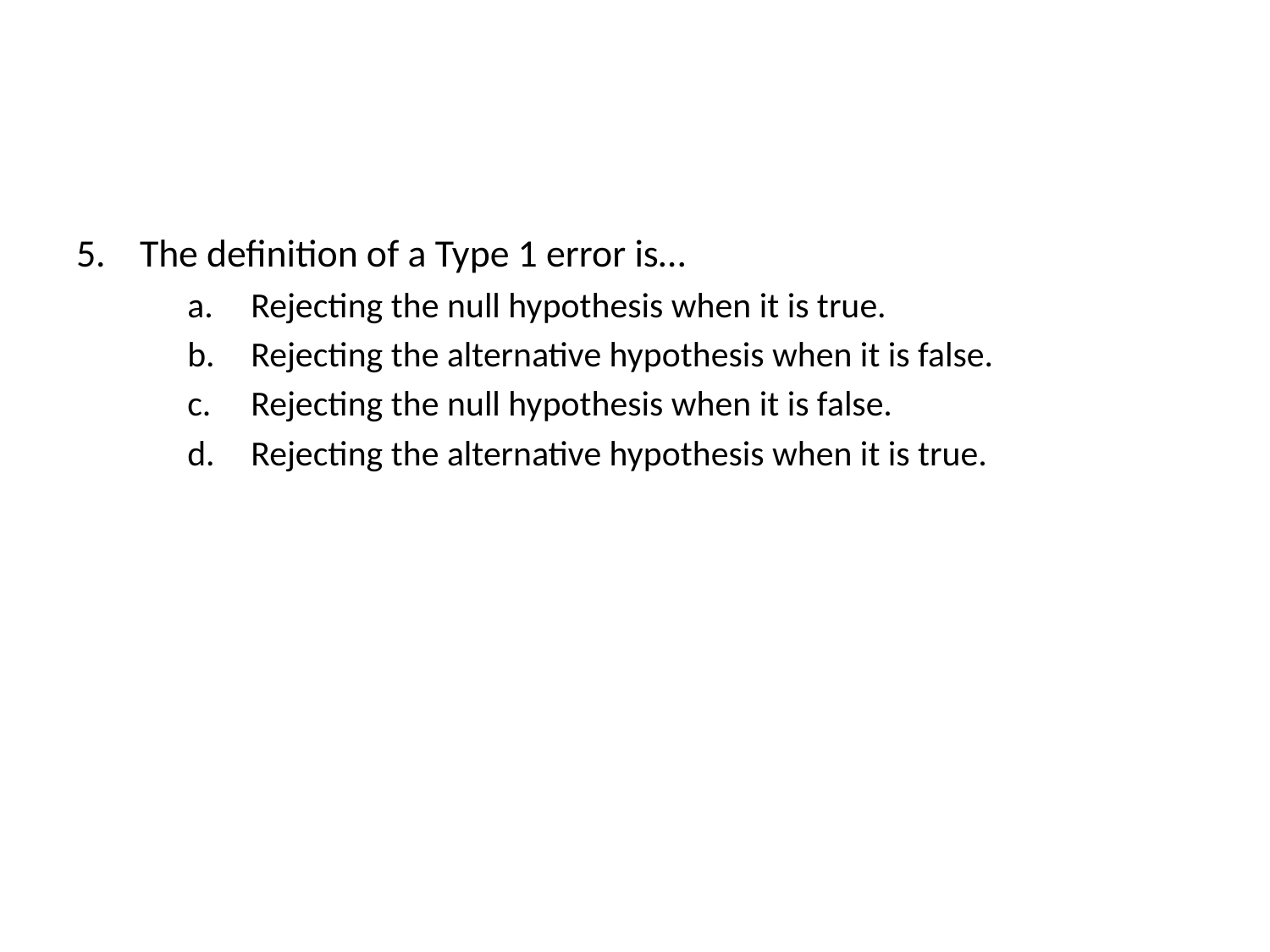

#
The definition of a Type 1 error is…
Rejecting the null hypothesis when it is true.
Rejecting the alternative hypothesis when it is false.
Rejecting the null hypothesis when it is false.
Rejecting the alternative hypothesis when it is true.

## Slide 30
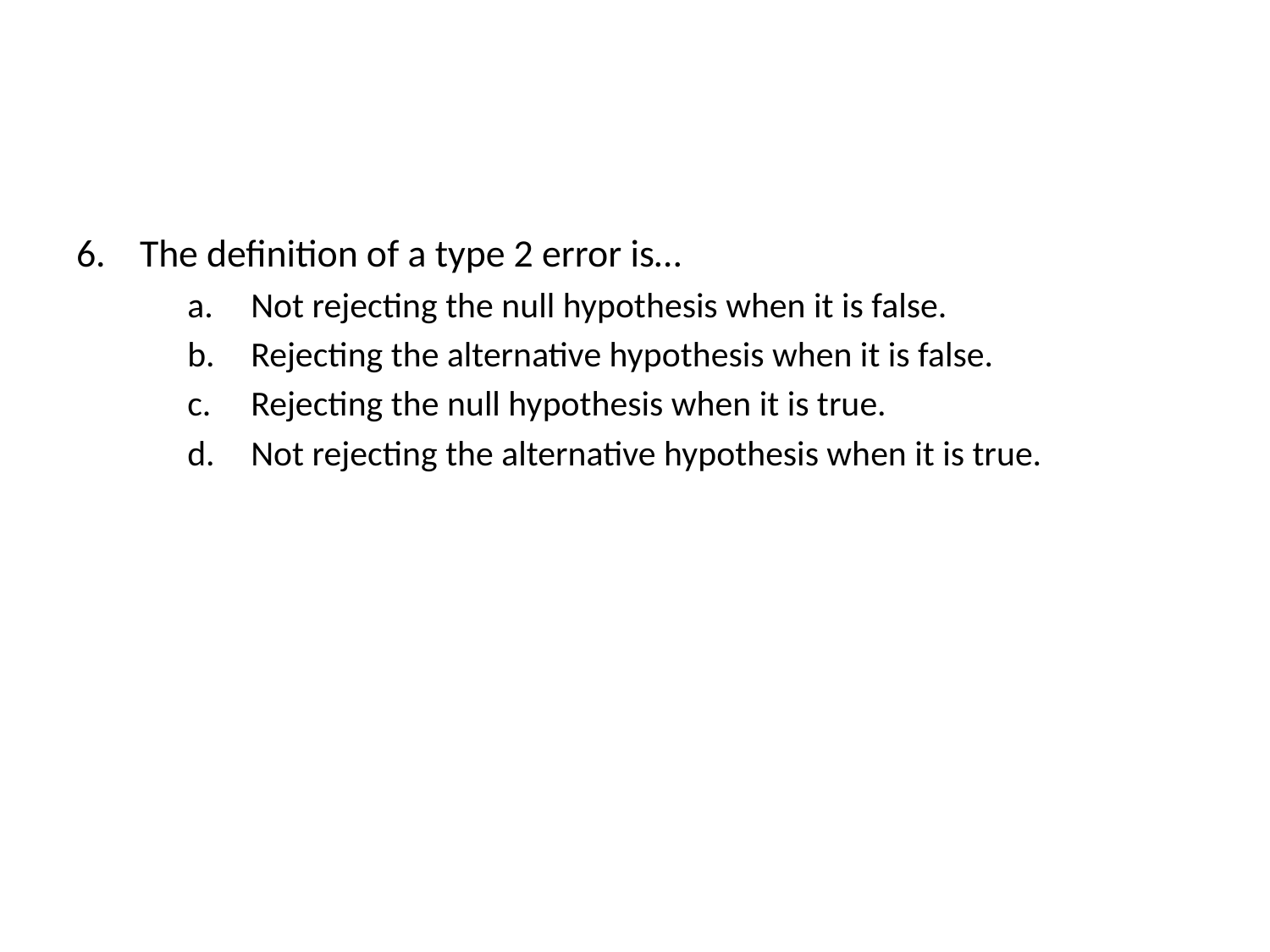

#
The definition of a type 2 error is…
Not rejecting the null hypothesis when it is false.
Rejecting the alternative hypothesis when it is false.
Rejecting the null hypothesis when it is true.
Not rejecting the alternative hypothesis when it is true.

## Slide 31
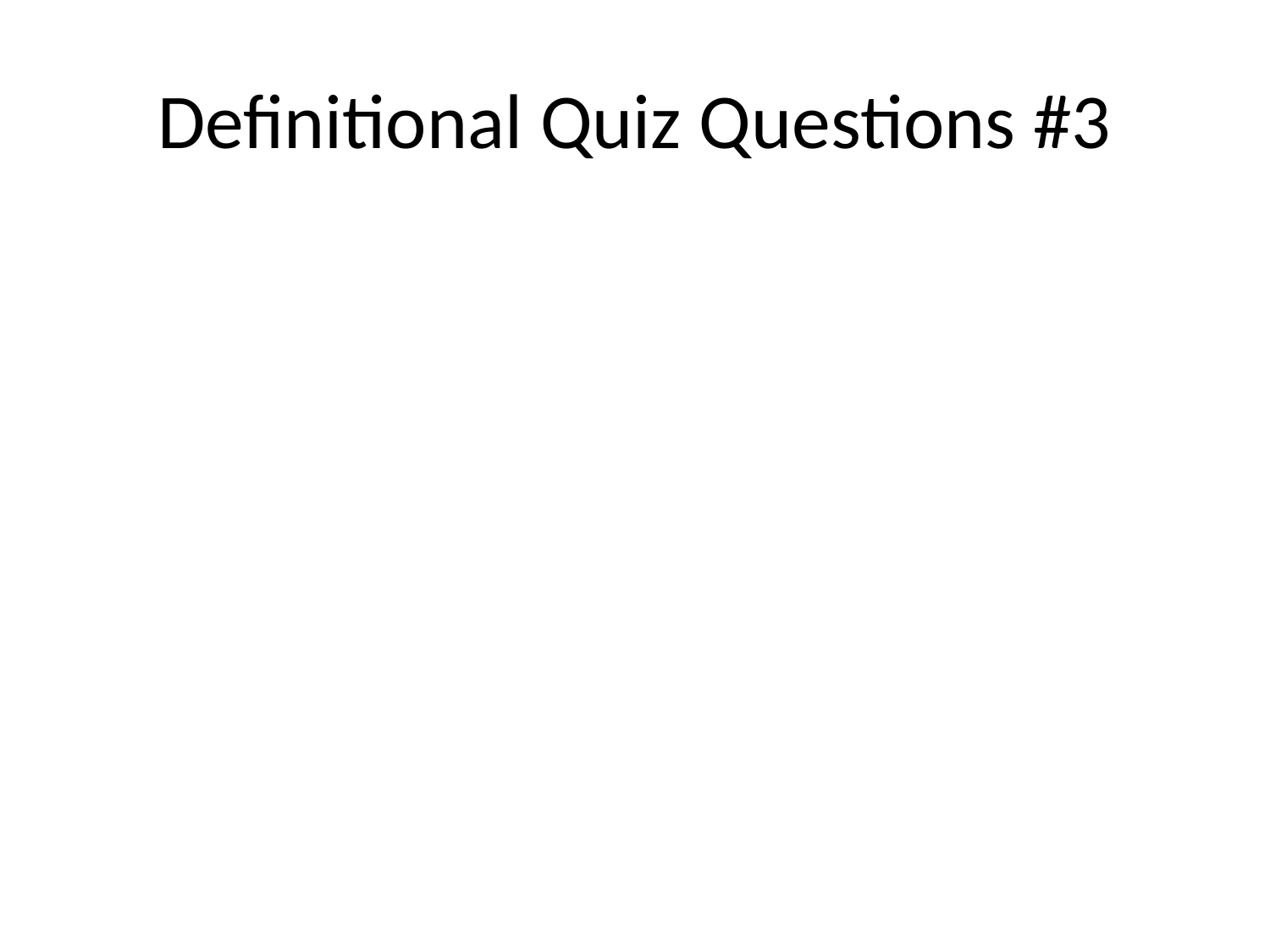

# Definitional Quiz Questions #3

## Slide 32
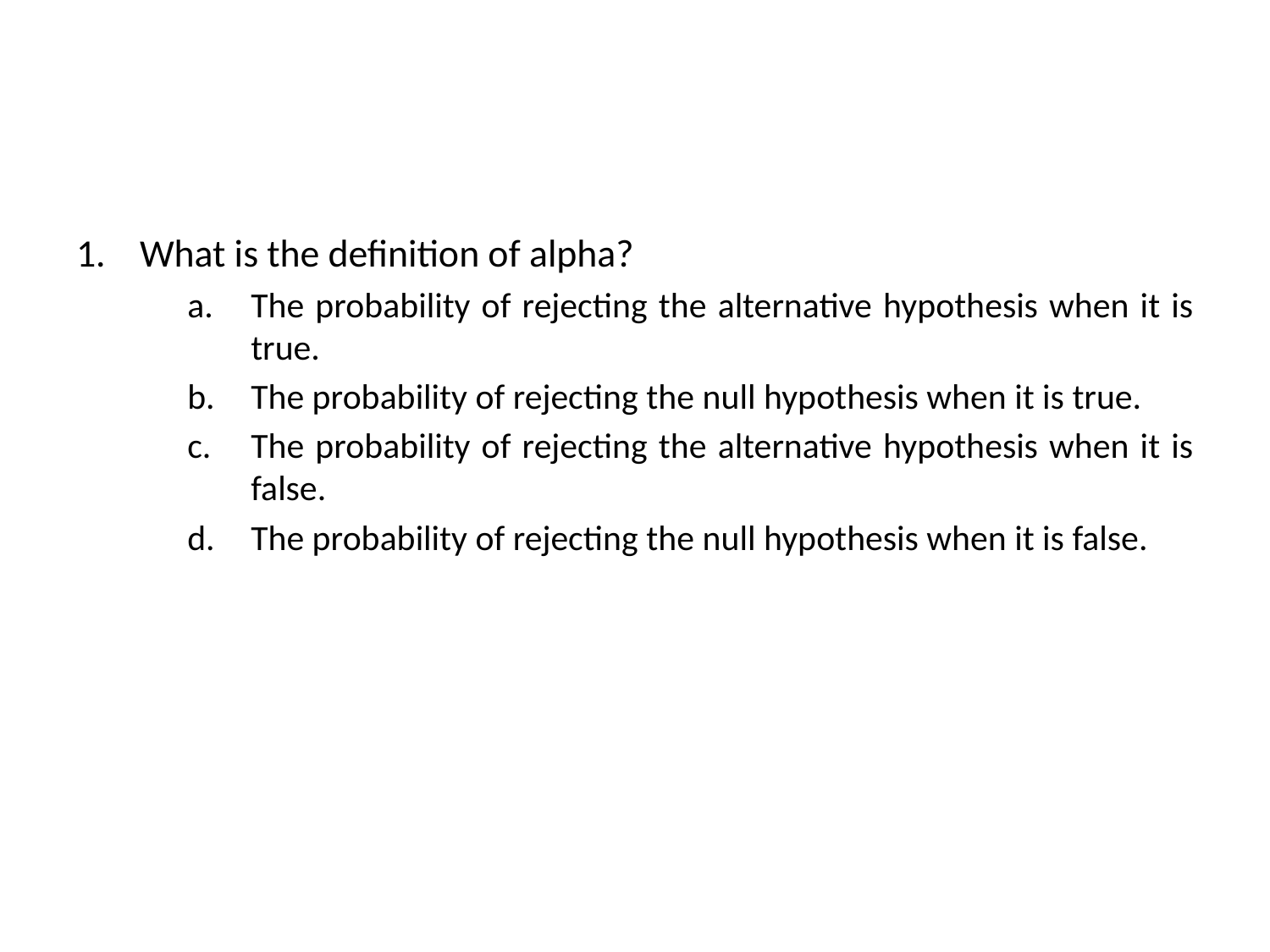

#
What is the definition of alpha?
The probability of rejecting the alternative hypothesis when it is true.
The probability of rejecting the null hypothesis when it is true.
The probability of rejecting the alternative hypothesis when it is false.
The probability of rejecting the null hypothesis when it is false.

## Slide 33
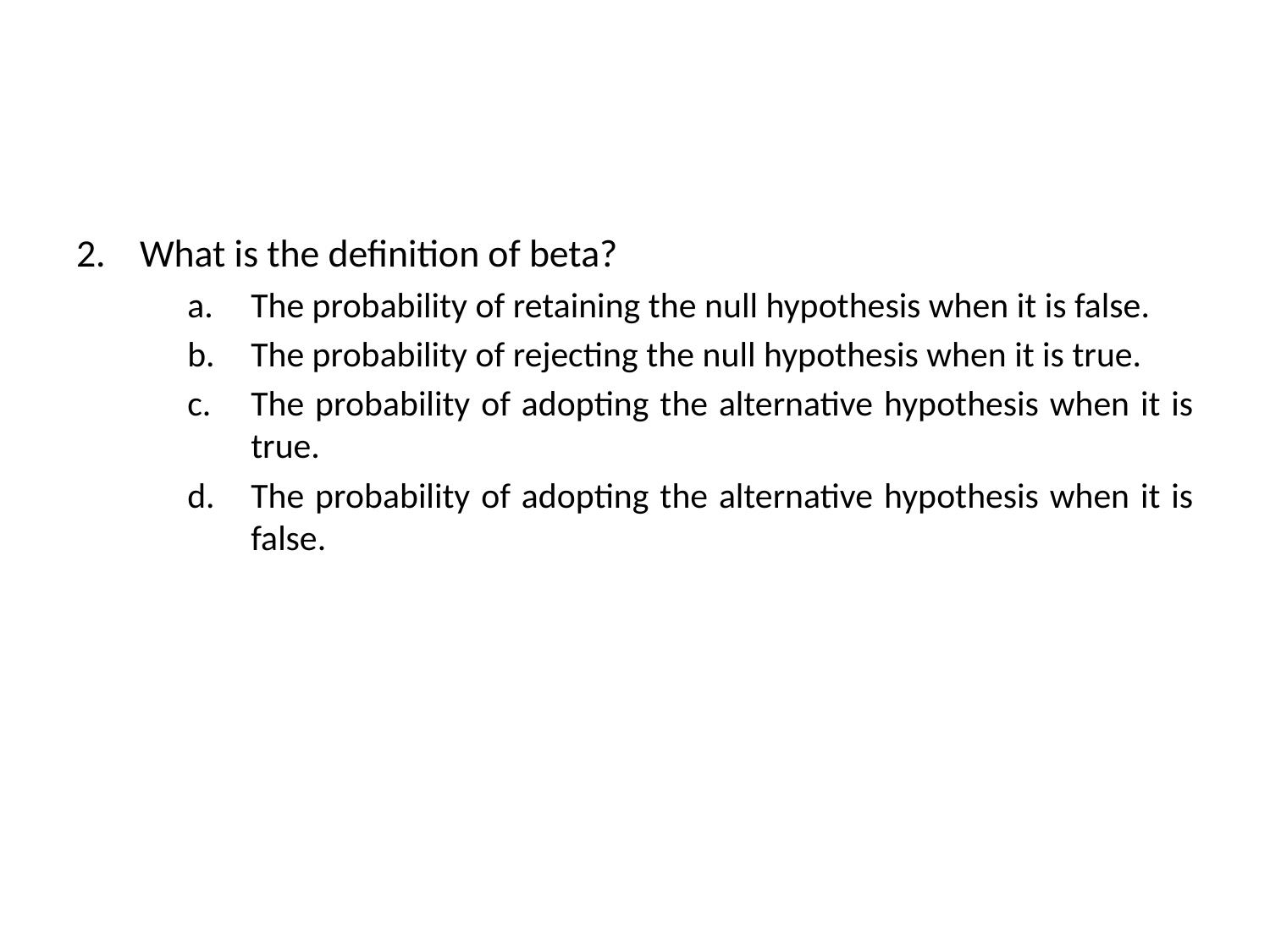

#
What is the definition of beta?
The probability of retaining the null hypothesis when it is false.
The probability of rejecting the null hypothesis when it is true.
The probability of adopting the alternative hypothesis when it is true.
The probability of adopting the alternative hypothesis when it is false.

## Slide 34
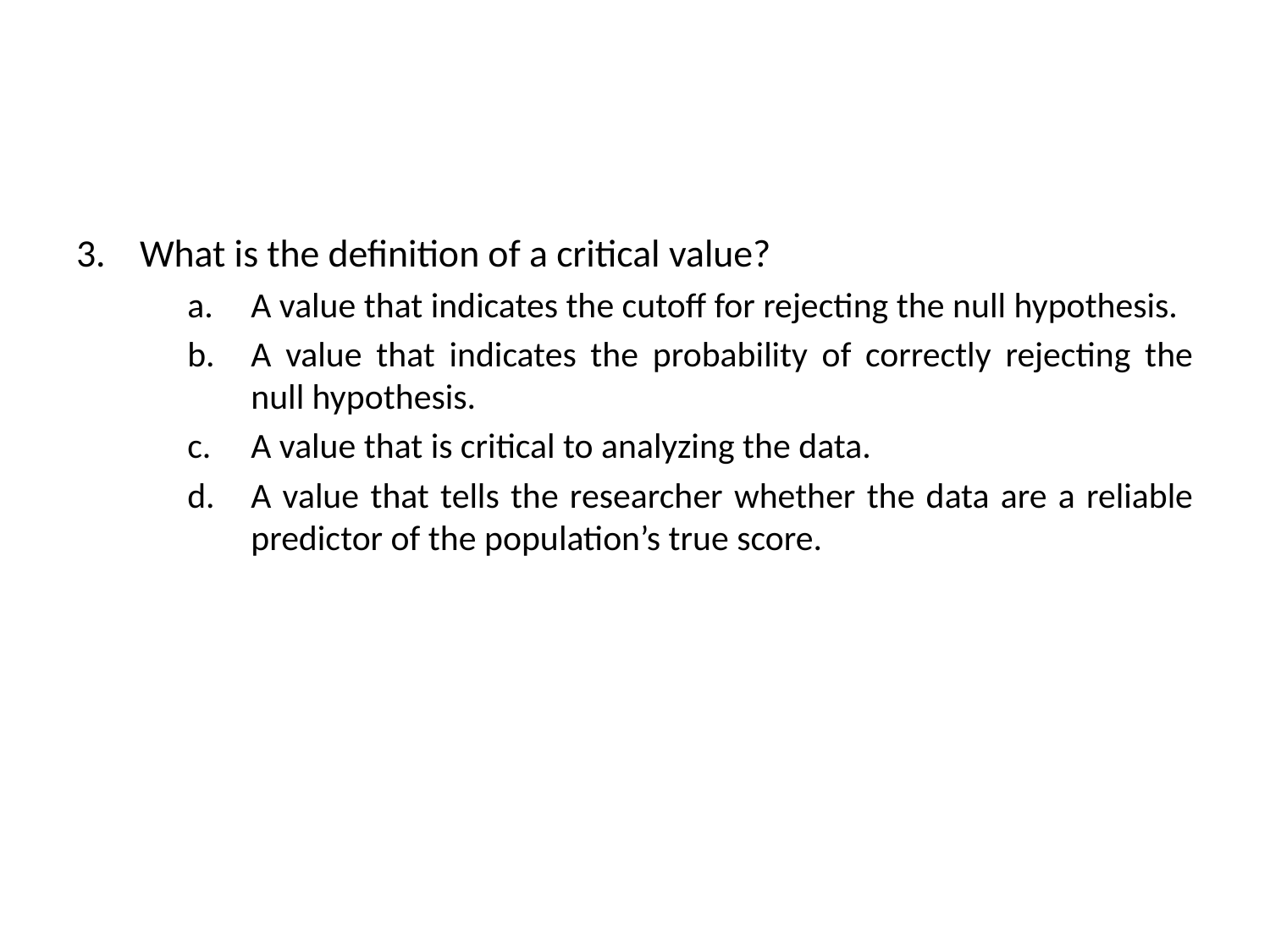

#
What is the definition of a critical value?
A value that indicates the cutoff for rejecting the null hypothesis.
A value that indicates the probability of correctly rejecting the null hypothesis.
A value that is critical to analyzing the data.
A value that tells the researcher whether the data are a reliable predictor of the population’s true score.

## Slide 35
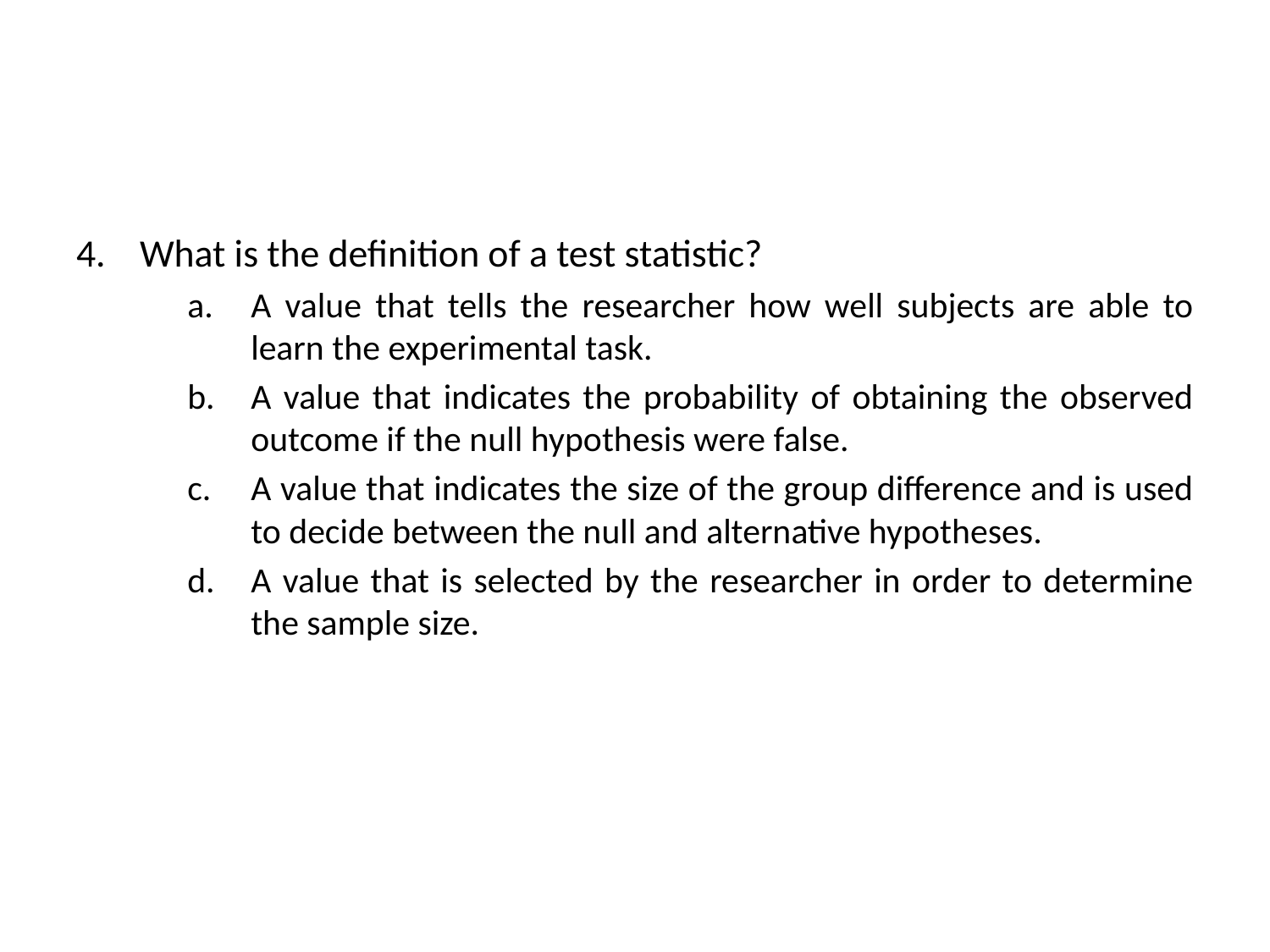

#
What is the definition of a test statistic?
A value that tells the researcher how well subjects are able to learn the experimental task.
A value that indicates the probability of obtaining the observed outcome if the null hypothesis were false.
A value that indicates the size of the group difference and is used to decide between the null and alternative hypotheses.
A value that is selected by the researcher in order to determine the sample size.

## Slide 36
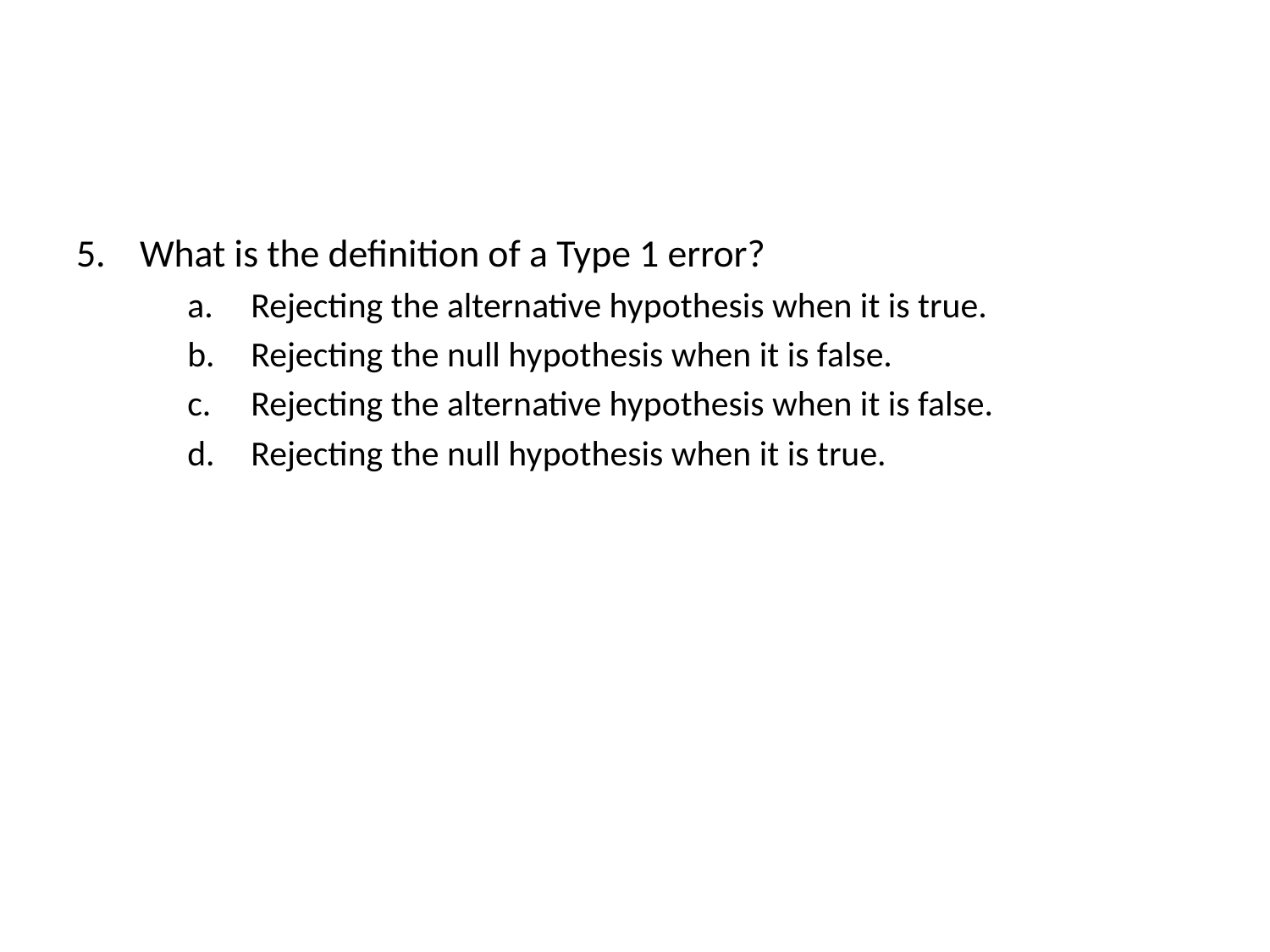

#
What is the definition of a Type 1 error?
Rejecting the alternative hypothesis when it is true.
Rejecting the null hypothesis when it is false.
Rejecting the alternative hypothesis when it is false.
Rejecting the null hypothesis when it is true.

## Slide 37
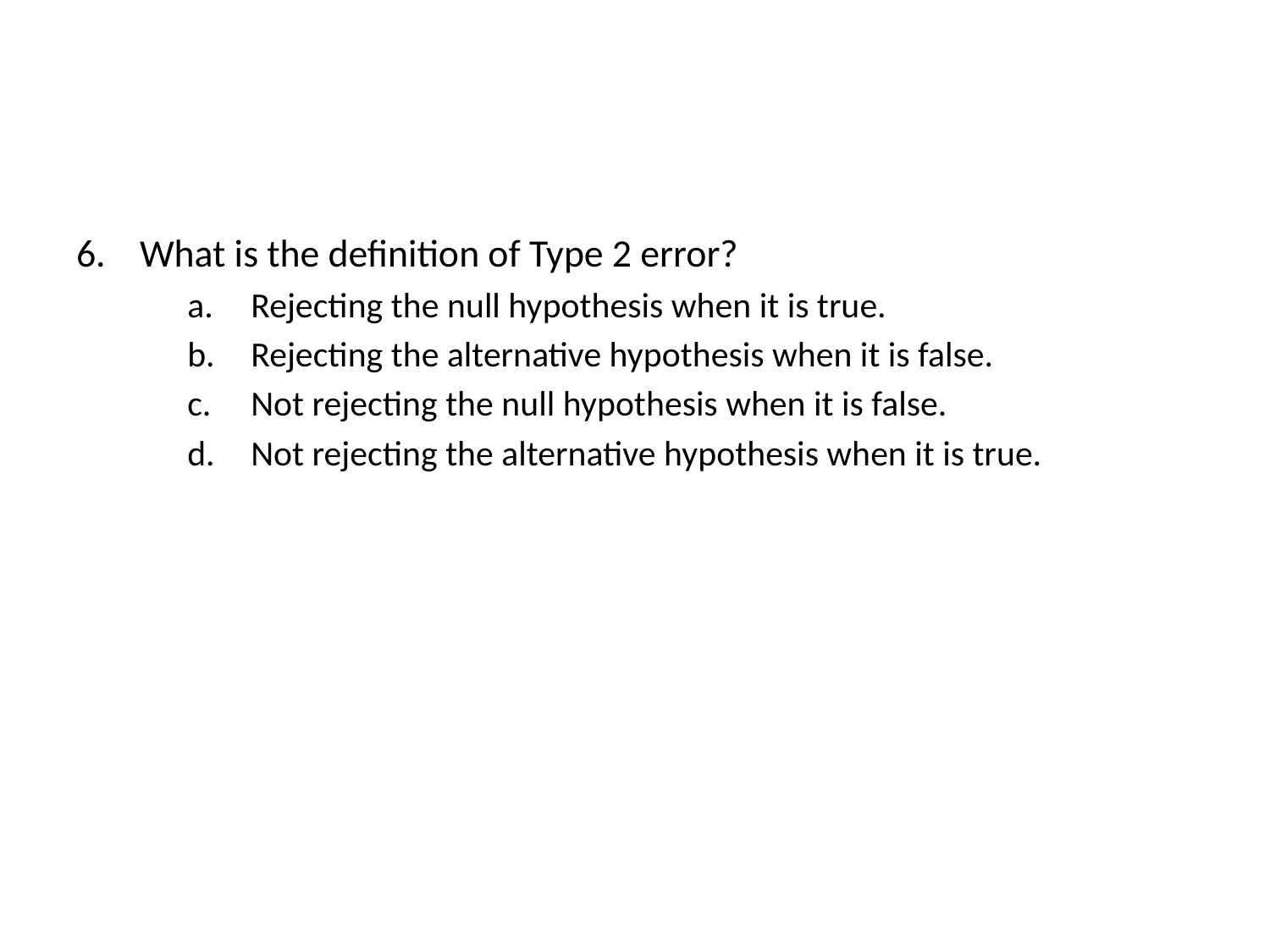

#
What is the definition of Type 2 error?
Rejecting the null hypothesis when it is true.
Rejecting the alternative hypothesis when it is false.
Not rejecting the null hypothesis when it is false.
Not rejecting the alternative hypothesis when it is true.

## Slide 38
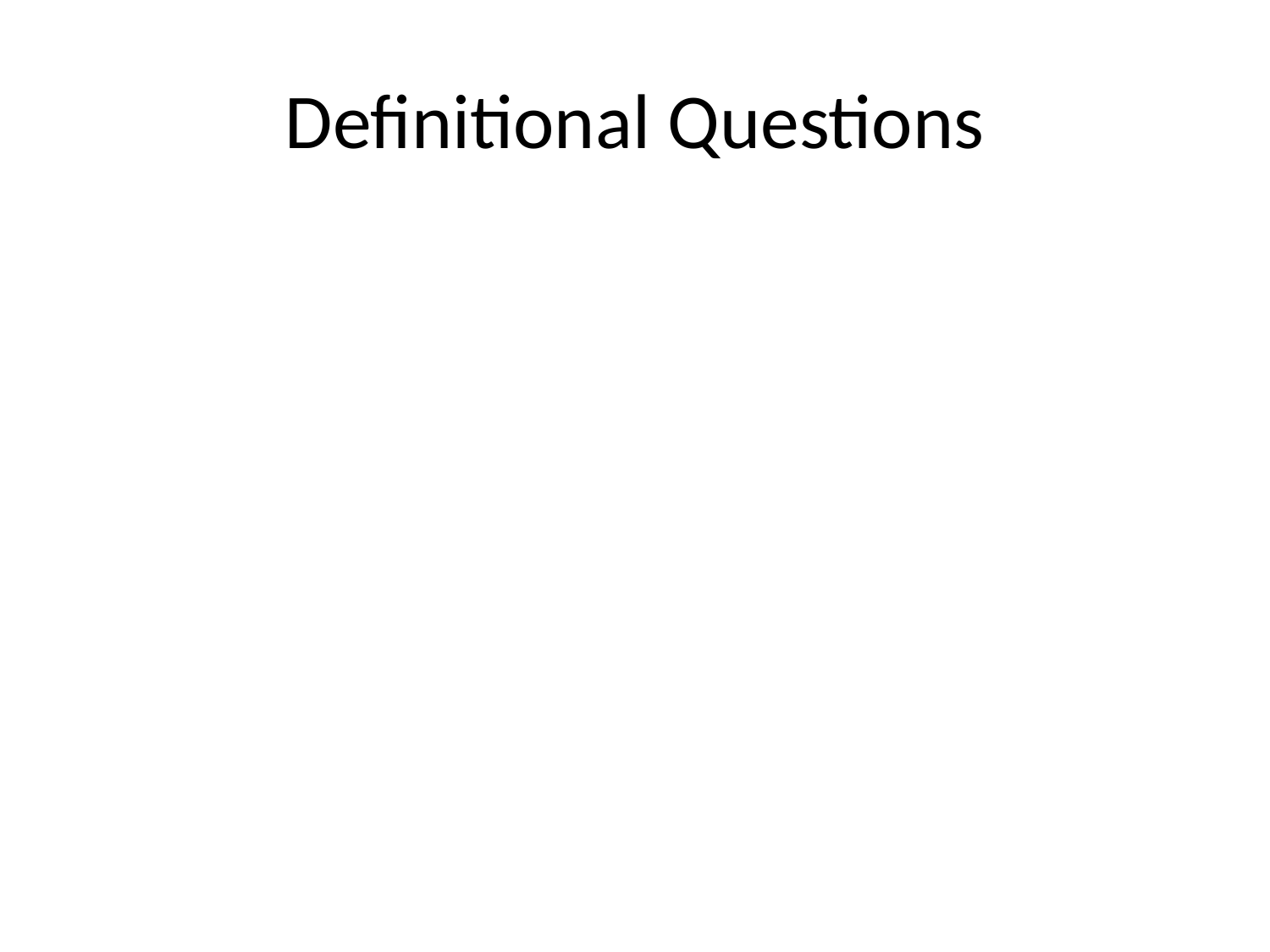

# Definitional Questions

## Slide 39
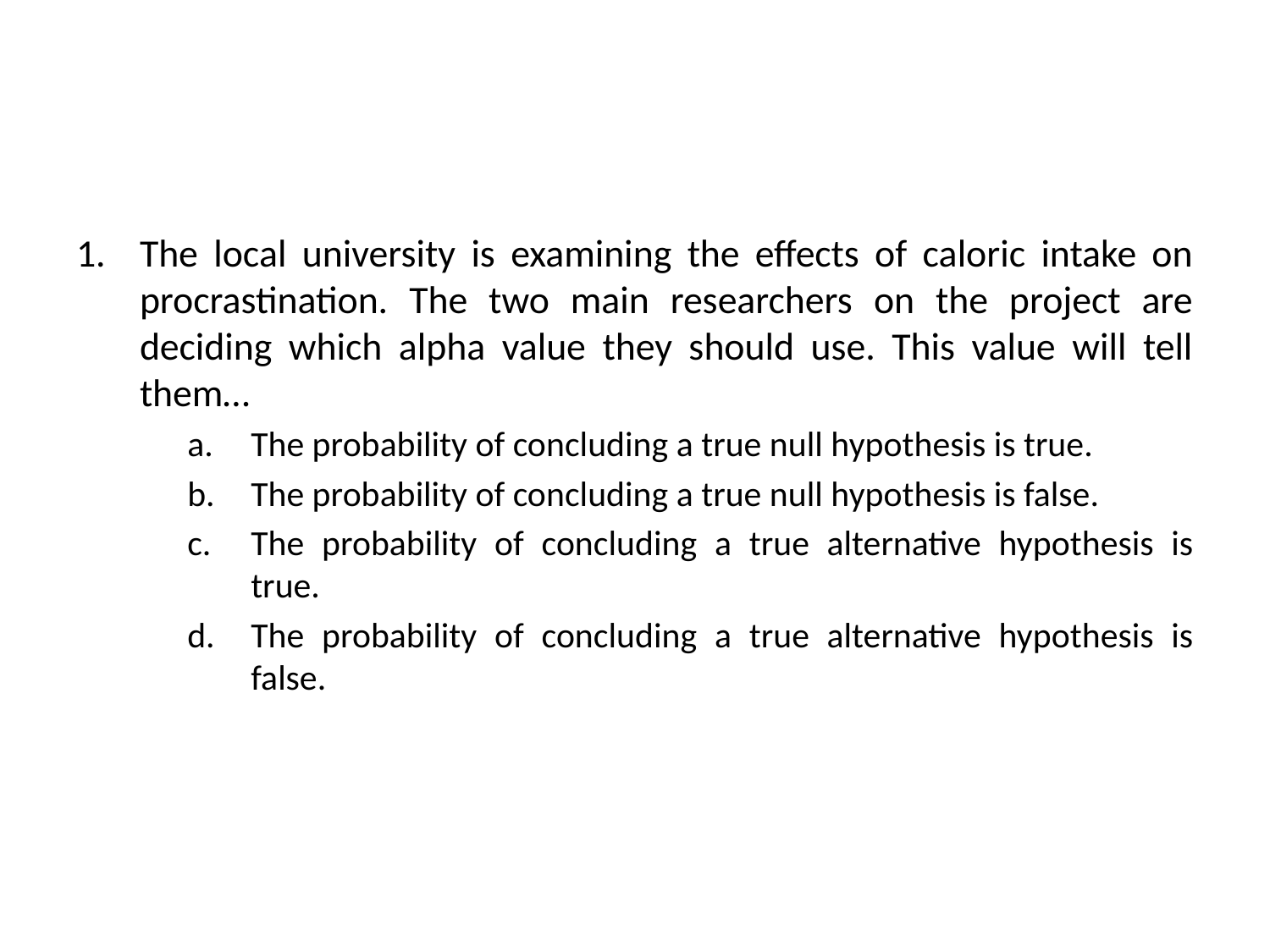

#
The local university is examining the effects of caloric intake on procrastination. The two main researchers on the project are deciding which alpha value they should use. This value will tell them…
The probability of concluding a true null hypothesis is true.
The probability of concluding a true null hypothesis is false.
The probability of concluding a true alternative hypothesis is true.
The probability of concluding a true alternative hypothesis is false.

## Slide 40
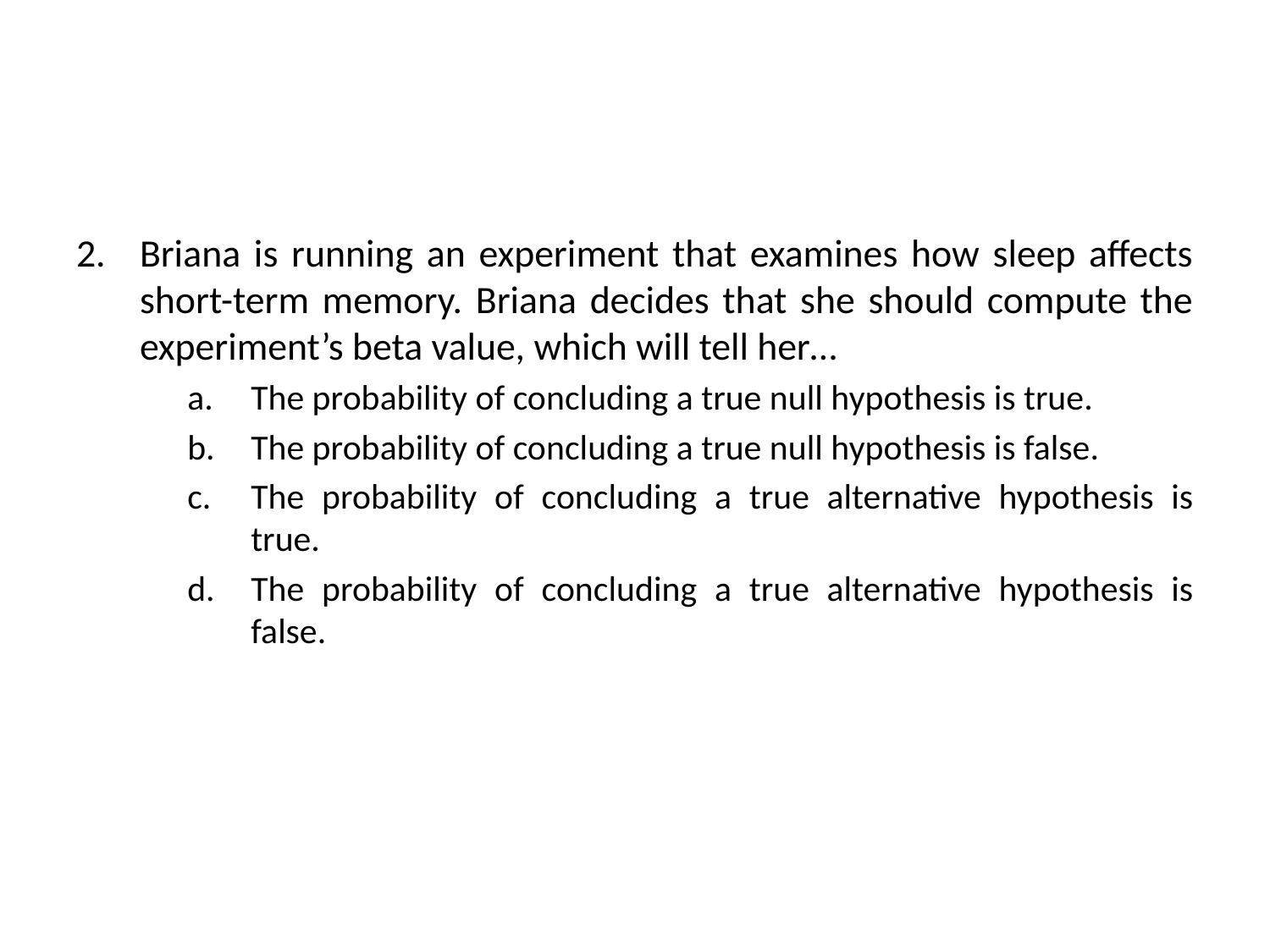

#
Briana is running an experiment that examines how sleep affects short-term memory. Briana decides that she should compute the experiment’s beta value, which will tell her…
The probability of concluding a true null hypothesis is true.
The probability of concluding a true null hypothesis is false.
The probability of concluding a true alternative hypothesis is true.
The probability of concluding a true alternative hypothesis is false.

## Slide 41
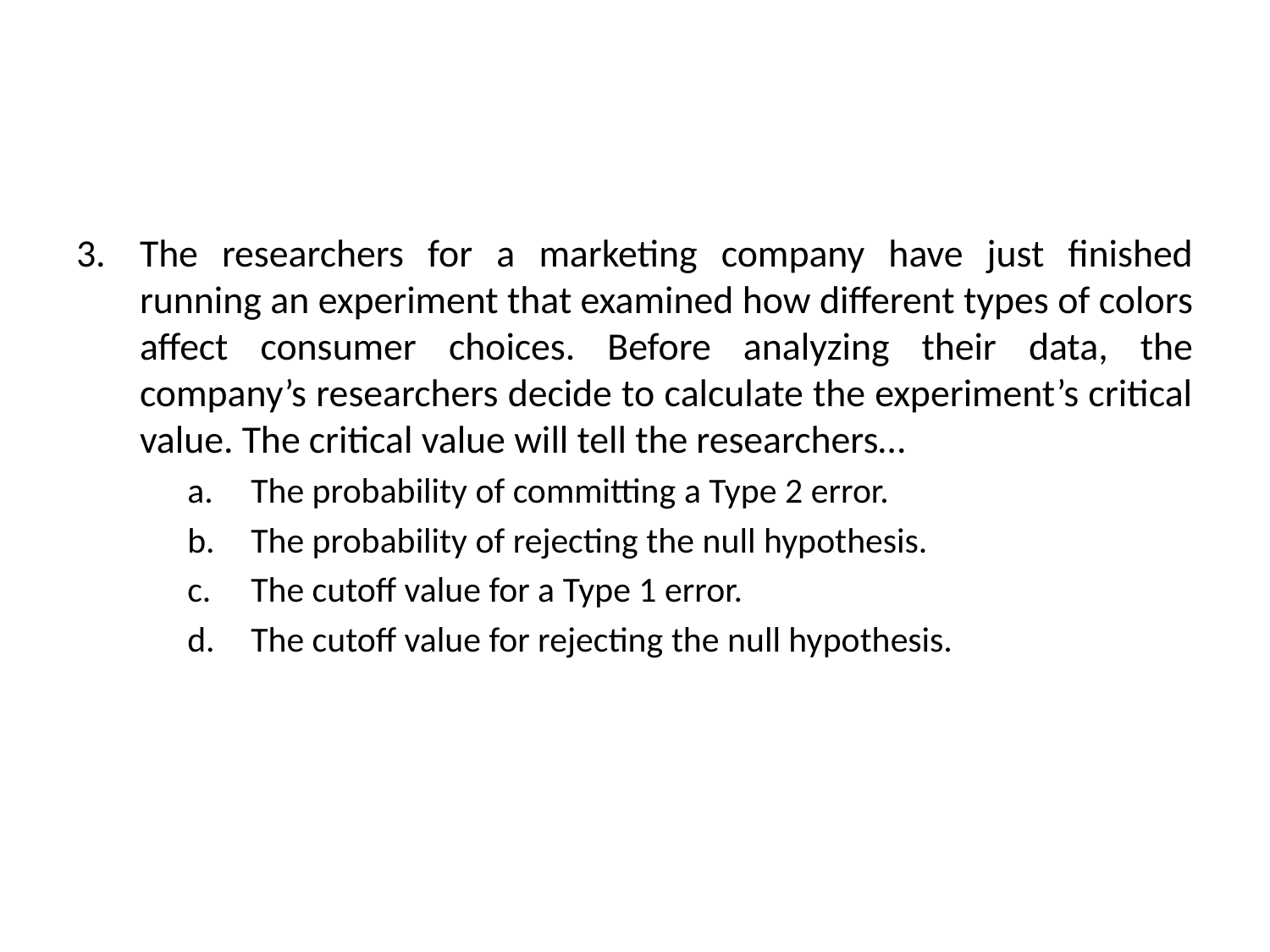

#
The researchers for a marketing company have just finished running an experiment that examined how different types of colors affect consumer choices. Before analyzing their data, the company’s researchers decide to calculate the experiment’s critical value. The critical value will tell the researchers…
The probability of committing a Type 2 error.
The probability of rejecting the null hypothesis.
The cutoff value for a Type 1 error.
The cutoff value for rejecting the null hypothesis.

## Slide 42
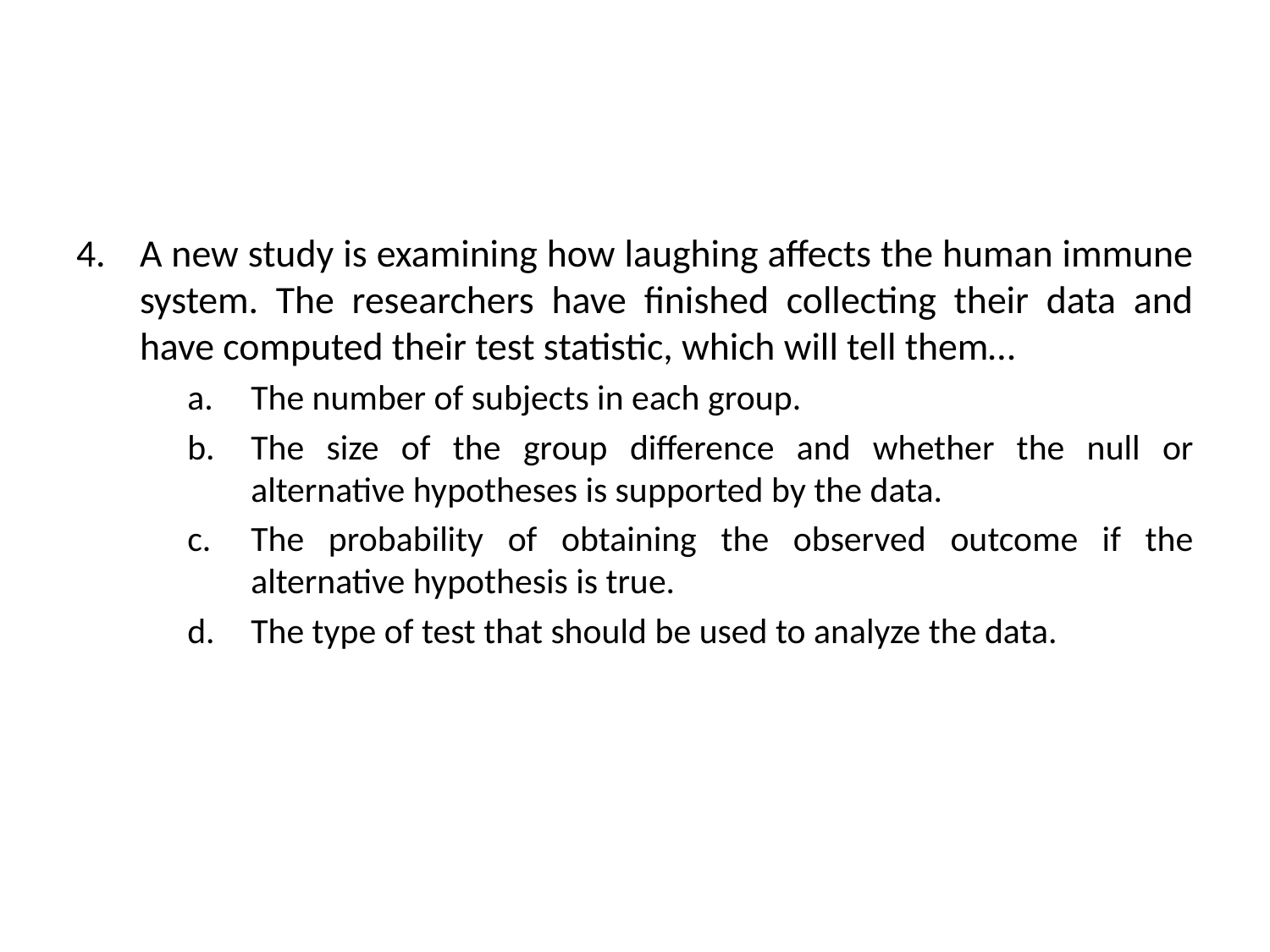

#
A new study is examining how laughing affects the human immune system. The researchers have finished collecting their data and have computed their test statistic, which will tell them…
The number of subjects in each group.
The size of the group difference and whether the null or alternative hypotheses is supported by the data.
The probability of obtaining the observed outcome if the alternative hypothesis is true.
The type of test that should be used to analyze the data.

## Slide 43
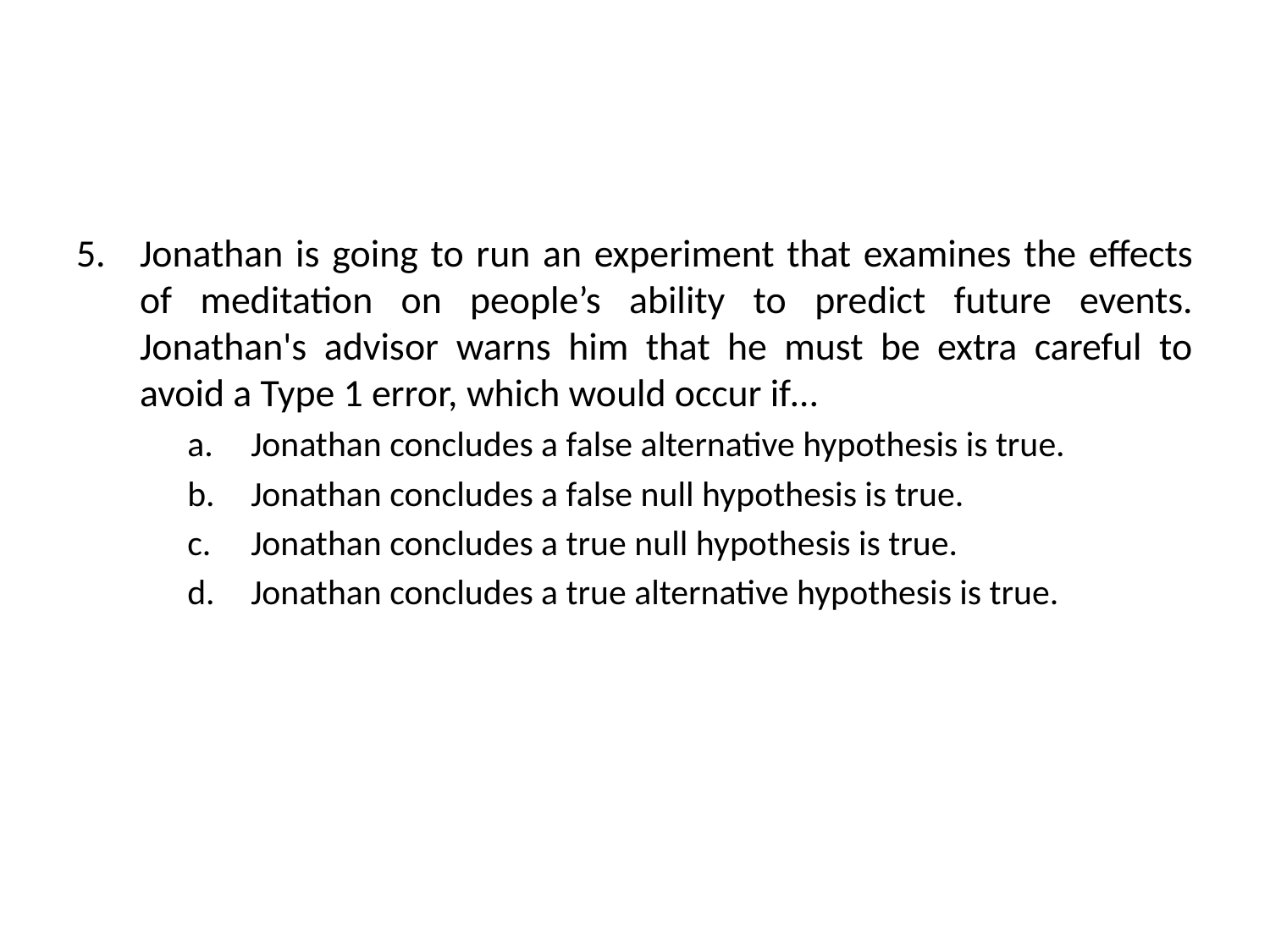

#
Jonathan is going to run an experiment that examines the effects of meditation on people’s ability to predict future events. Jonathan's advisor warns him that he must be extra careful to avoid a Type 1 error, which would occur if…
Jonathan concludes a false alternative hypothesis is true.
Jonathan concludes a false null hypothesis is true.
Jonathan concludes a true null hypothesis is true.
Jonathan concludes a true alternative hypothesis is true.

## Slide 44
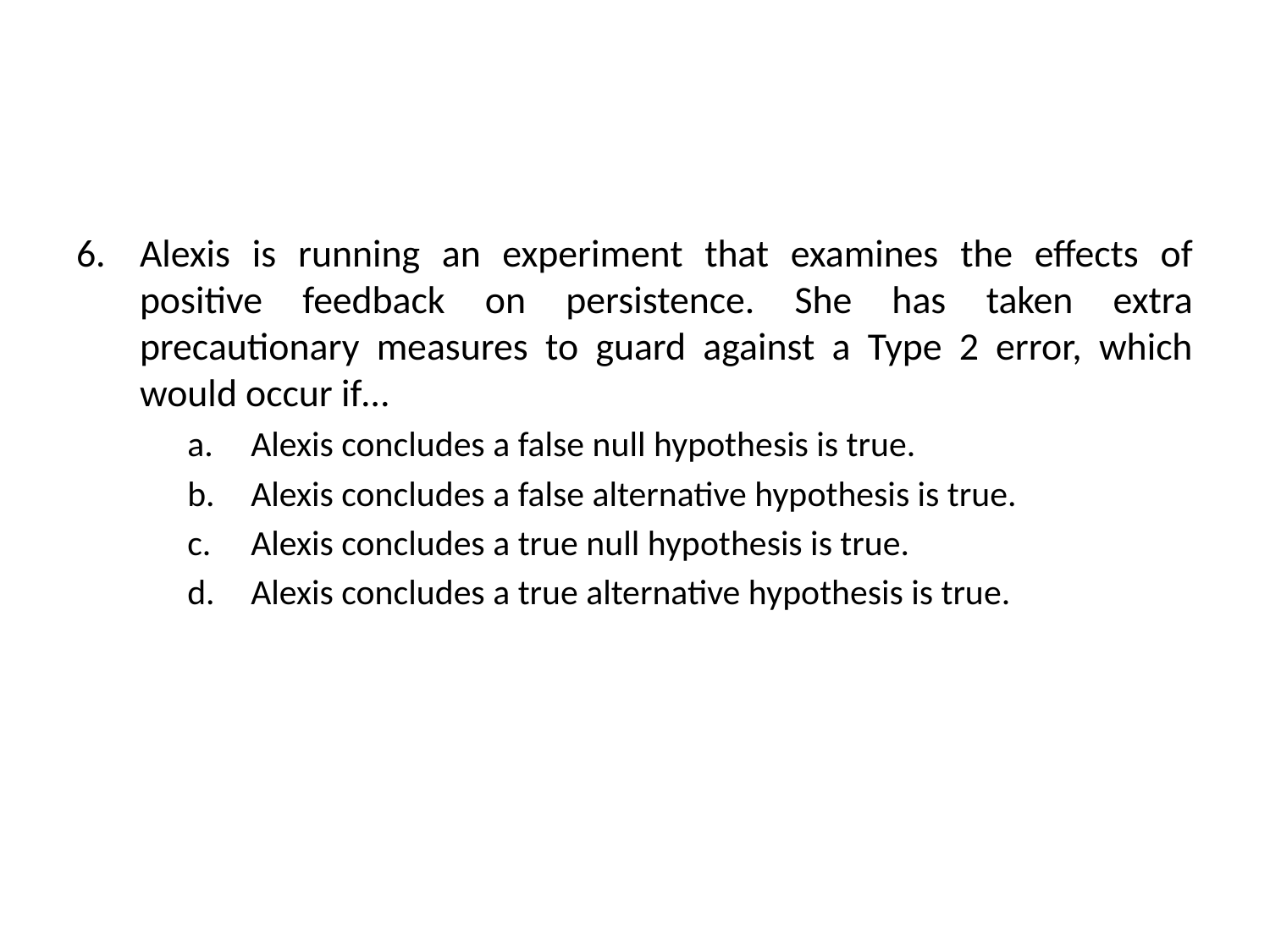

#
Alexis is running an experiment that examines the effects of positive feedback on persistence. She has taken extra precautionary measures to guard against a Type 2 error, which would occur if…
Alexis concludes a false null hypothesis is true.
Alexis concludes a false alternative hypothesis is true.
Alexis concludes a true null hypothesis is true.
Alexis concludes a true alternative hypothesis is true.

## Slide 45
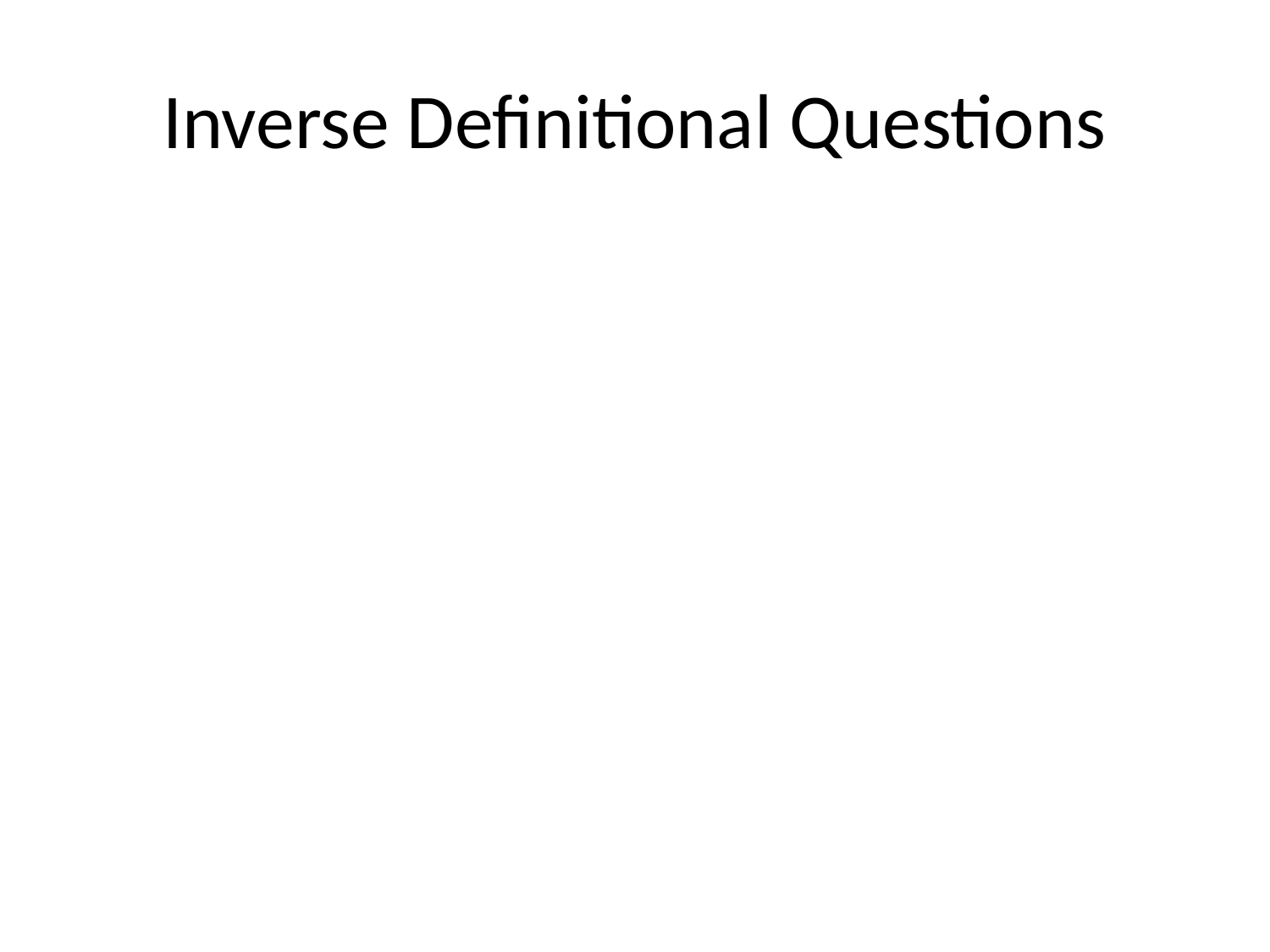

# Inverse Definitional Questions

## Slide 46
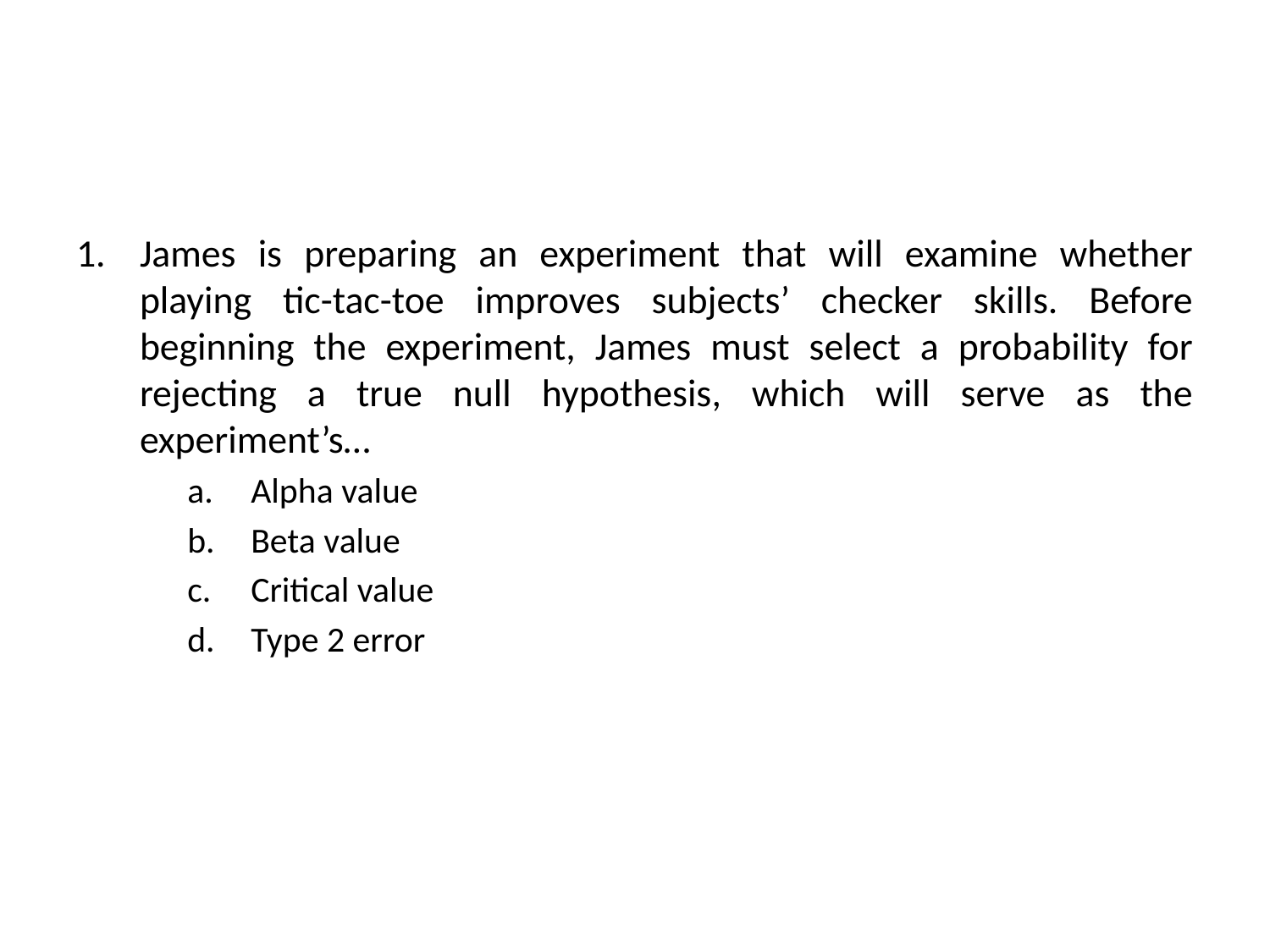

#
James is preparing an experiment that will examine whether playing tic-tac-toe improves subjects’ checker skills. Before beginning the experiment, James must select a probability for rejecting a true null hypothesis, which will serve as the experiment’s…
Alpha value
Beta value
Critical value
Type 2 error

## Slide 47
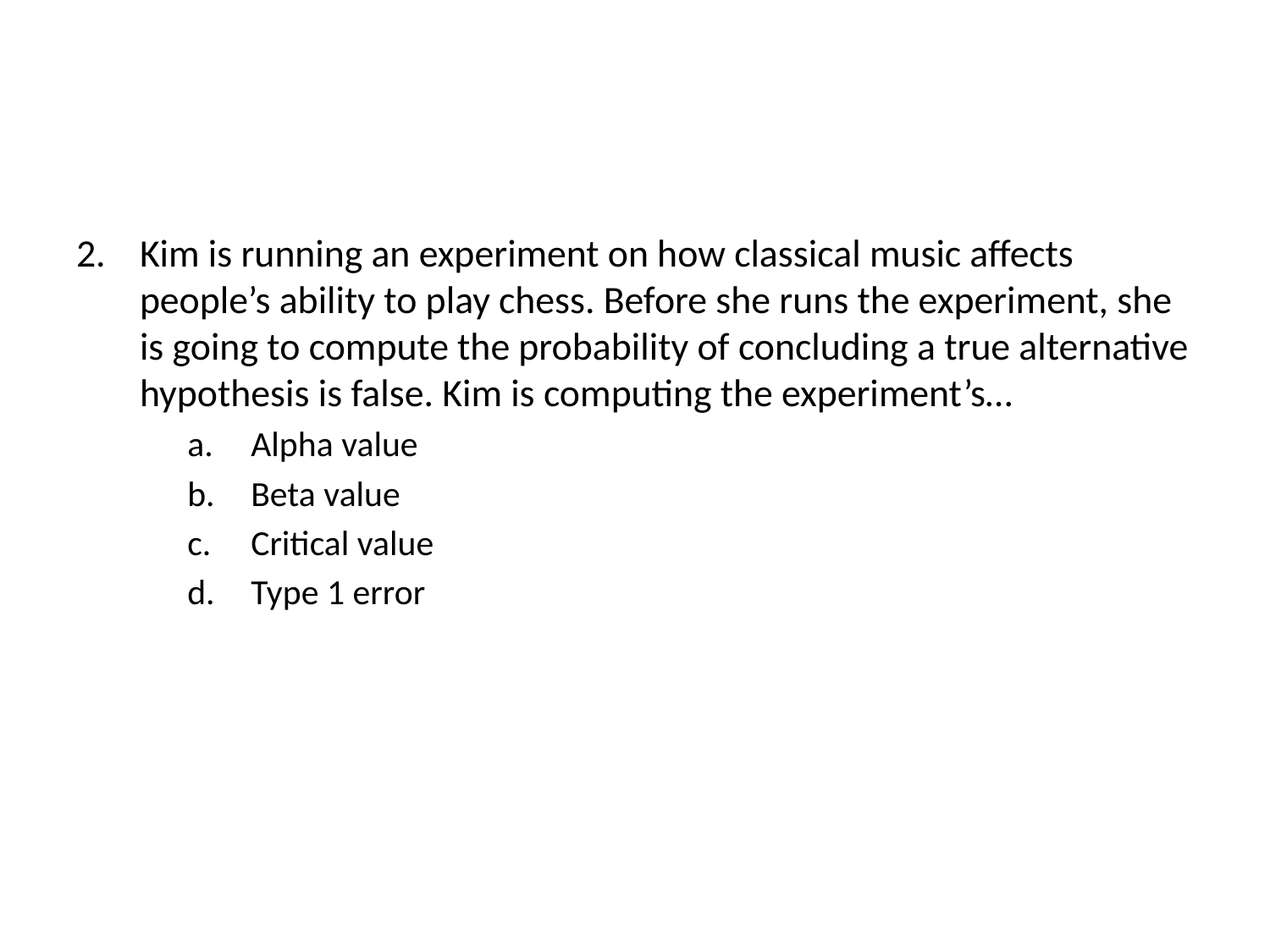

#
Kim is running an experiment on how classical music affects people’s ability to play chess. Before she runs the experiment, she is going to compute the probability of concluding a true alternative hypothesis is false. Kim is computing the experiment’s…
Alpha value
Beta value
Critical value
Type 1 error

## Slide 48
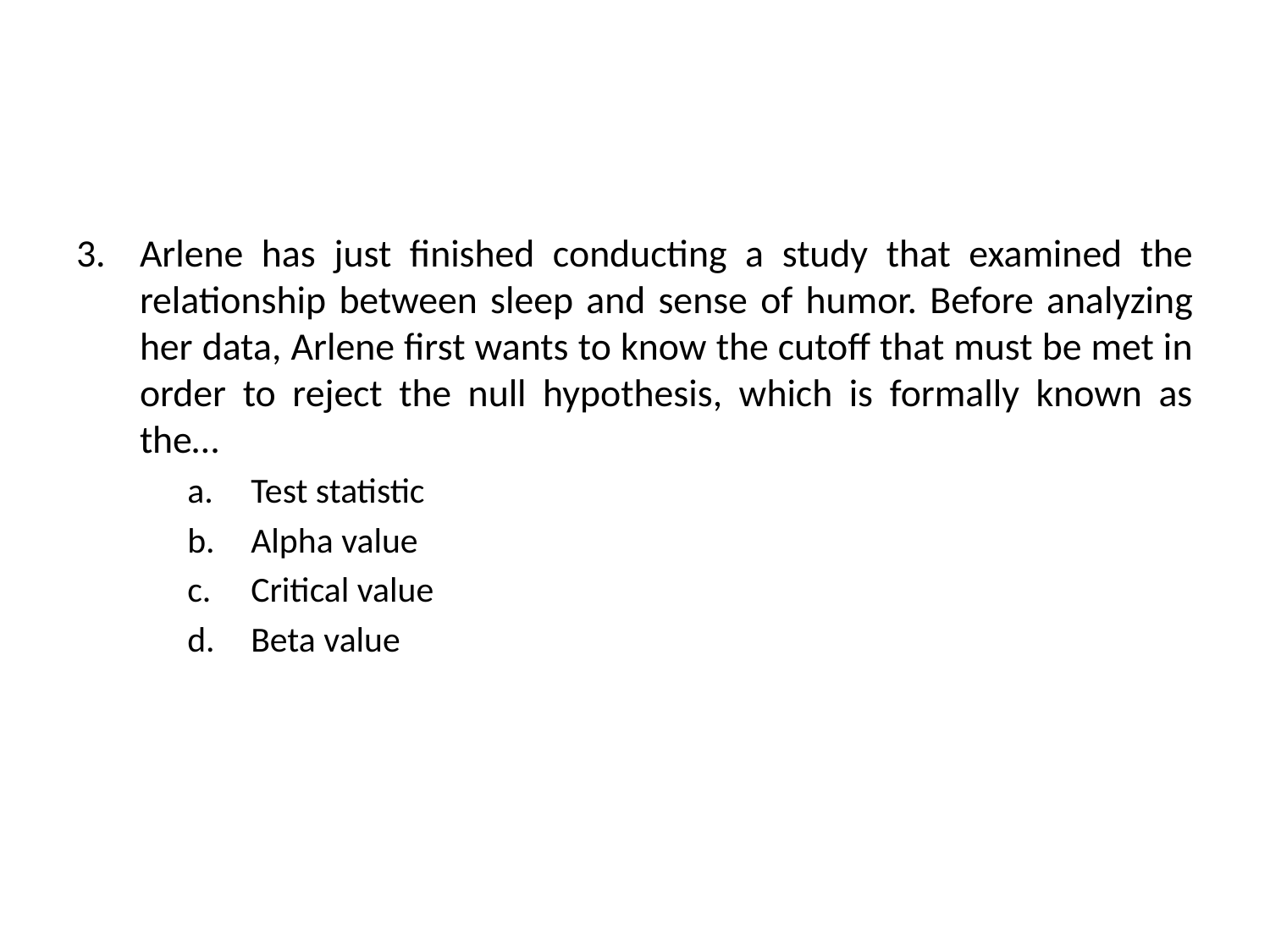

#
Arlene has just finished conducting a study that examined the relationship between sleep and sense of humor. Before analyzing her data, Arlene first wants to know the cutoff that must be met in order to reject the null hypothesis, which is formally known as the…
Test statistic
Alpha value
Critical value
Beta value

## Slide 49
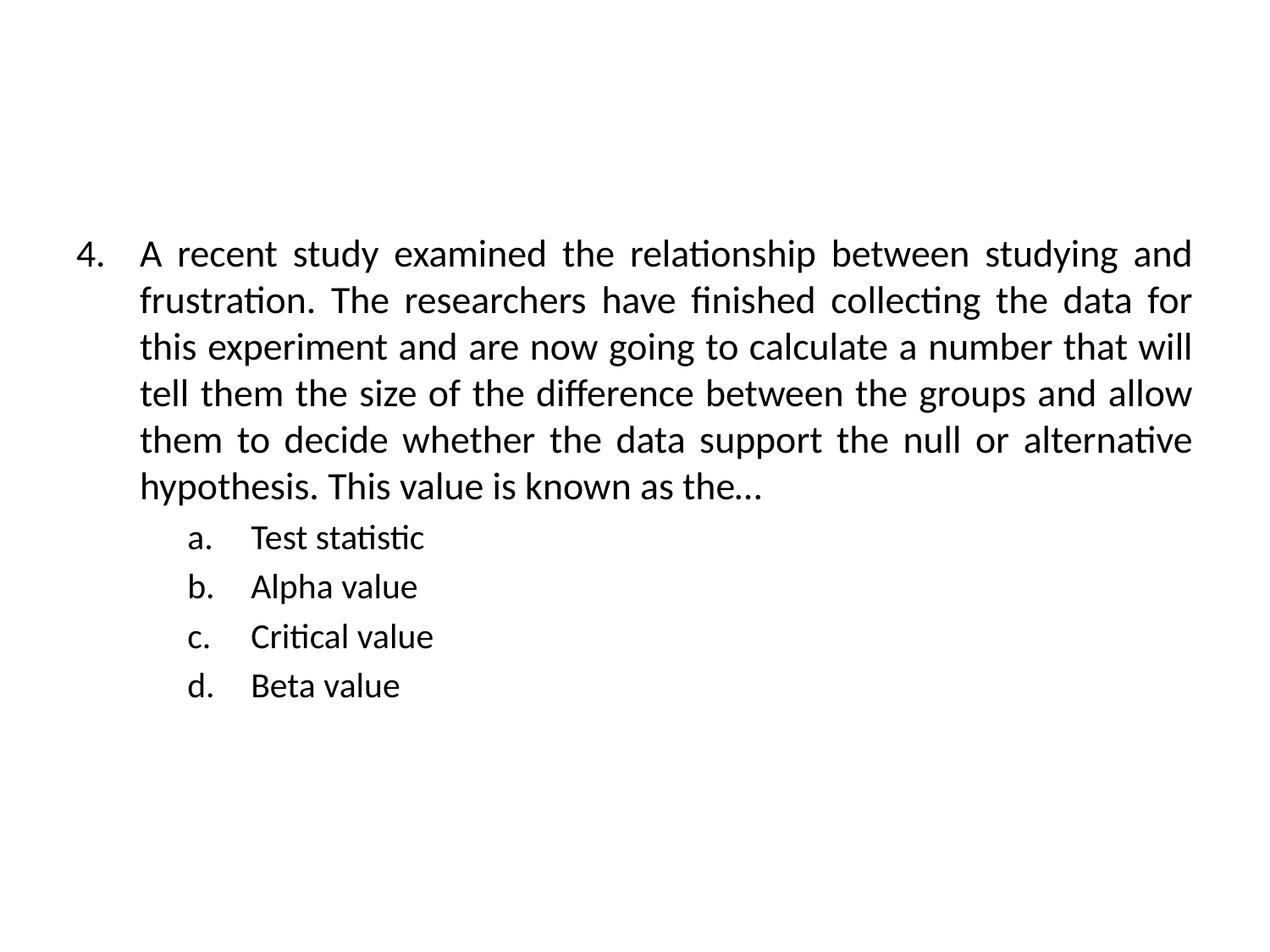

#
A recent study examined the relationship between studying and frustration. The researchers have finished collecting the data for this experiment and are now going to calculate a number that will tell them the size of the difference between the groups and allow them to decide whether the data support the null or alternative hypothesis. This value is known as the…
Test statistic
Alpha value
Critical value
Beta value

## Slide 50
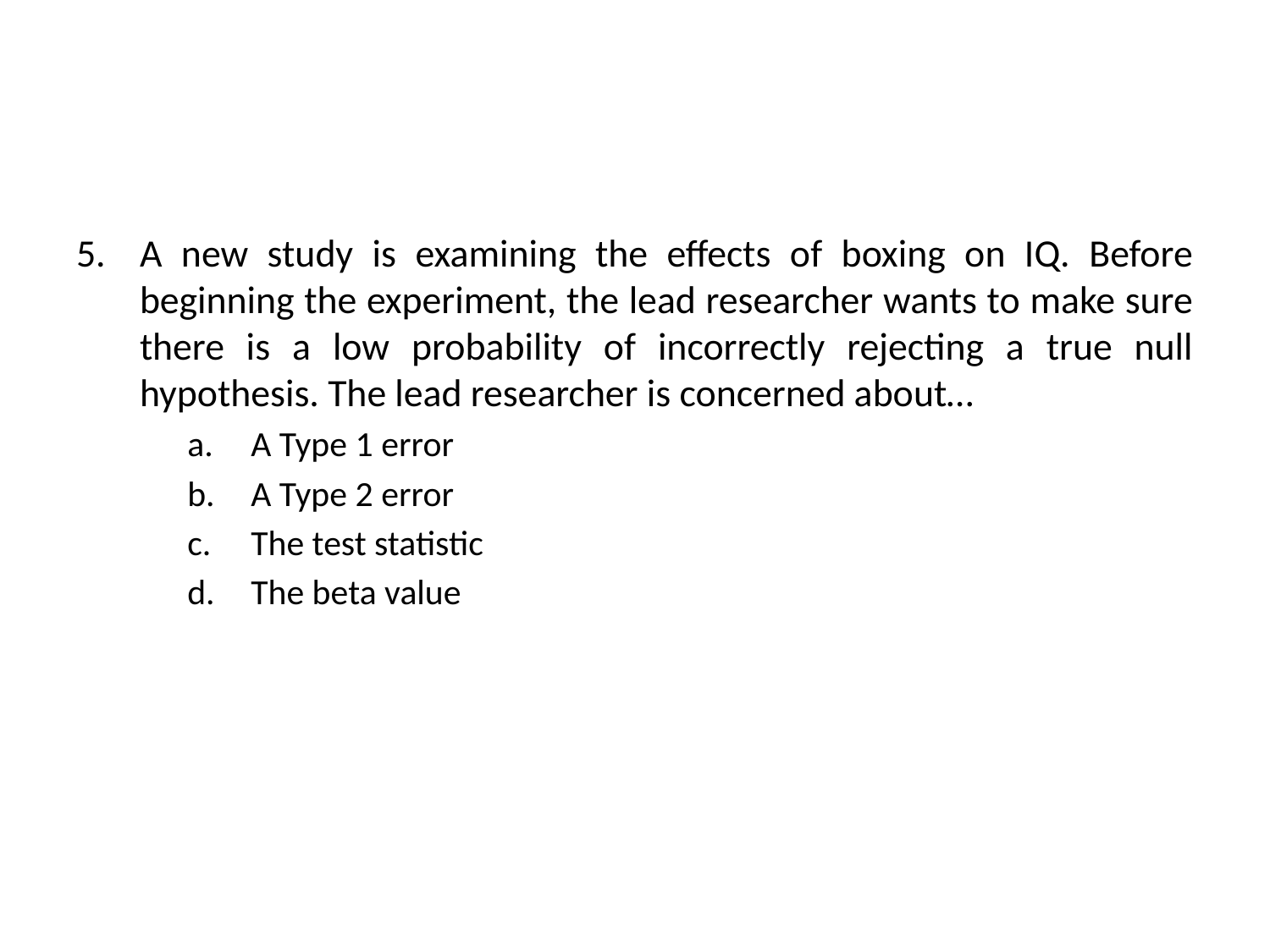

#
A new study is examining the effects of boxing on IQ. Before beginning the experiment, the lead researcher wants to make sure there is a low probability of incorrectly rejecting a true null hypothesis. The lead researcher is concerned about…
A Type 1 error
A Type 2 error
The test statistic
The beta value

## Slide 51
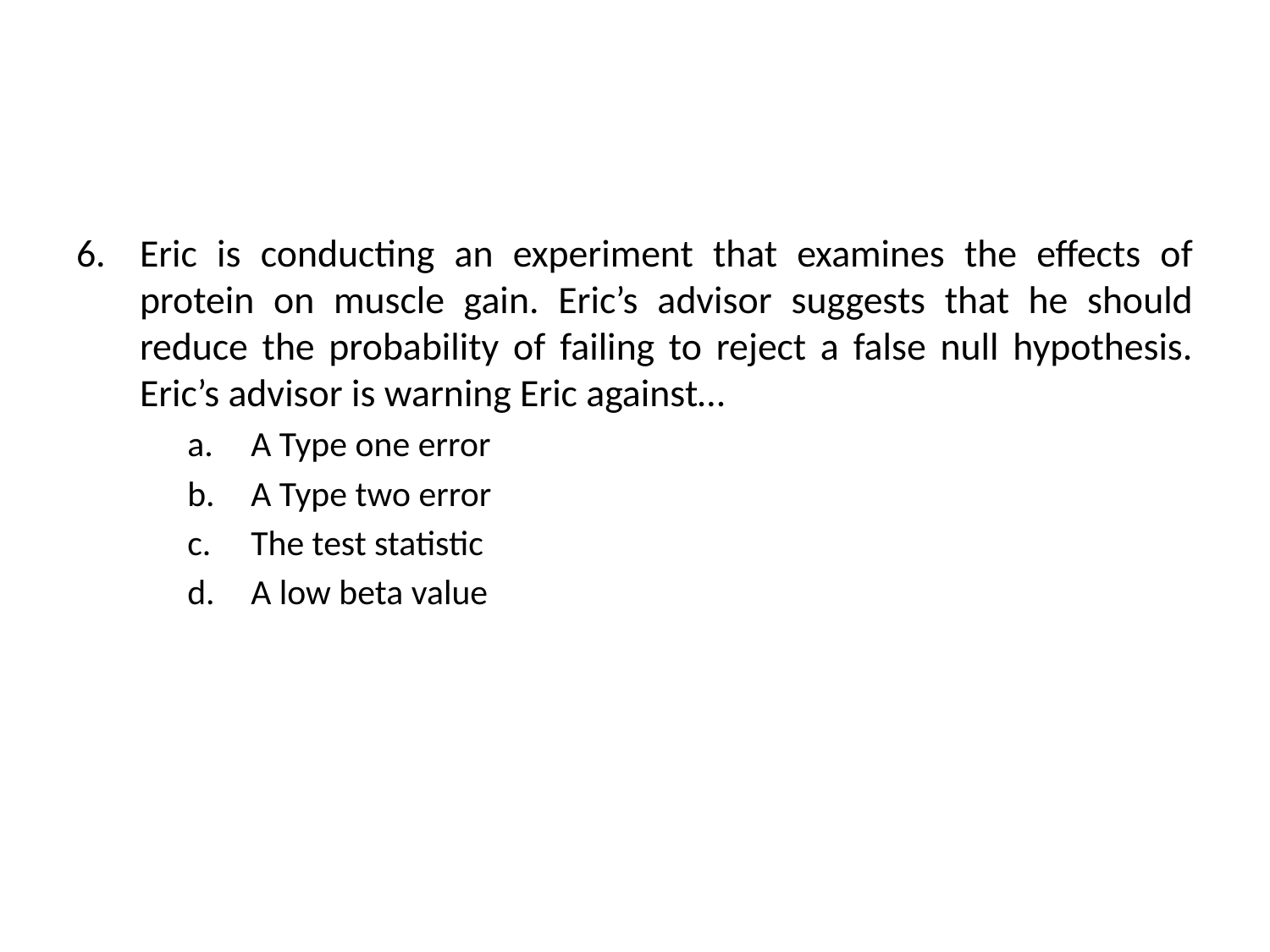

#
Eric is conducting an experiment that examines the effects of protein on muscle gain. Eric’s advisor suggests that he should reduce the probability of failing to reject a false null hypothesis. Eric’s advisor is warning Eric against…
A Type one error
A Type two error
The test statistic
A low beta value

## Slide 52
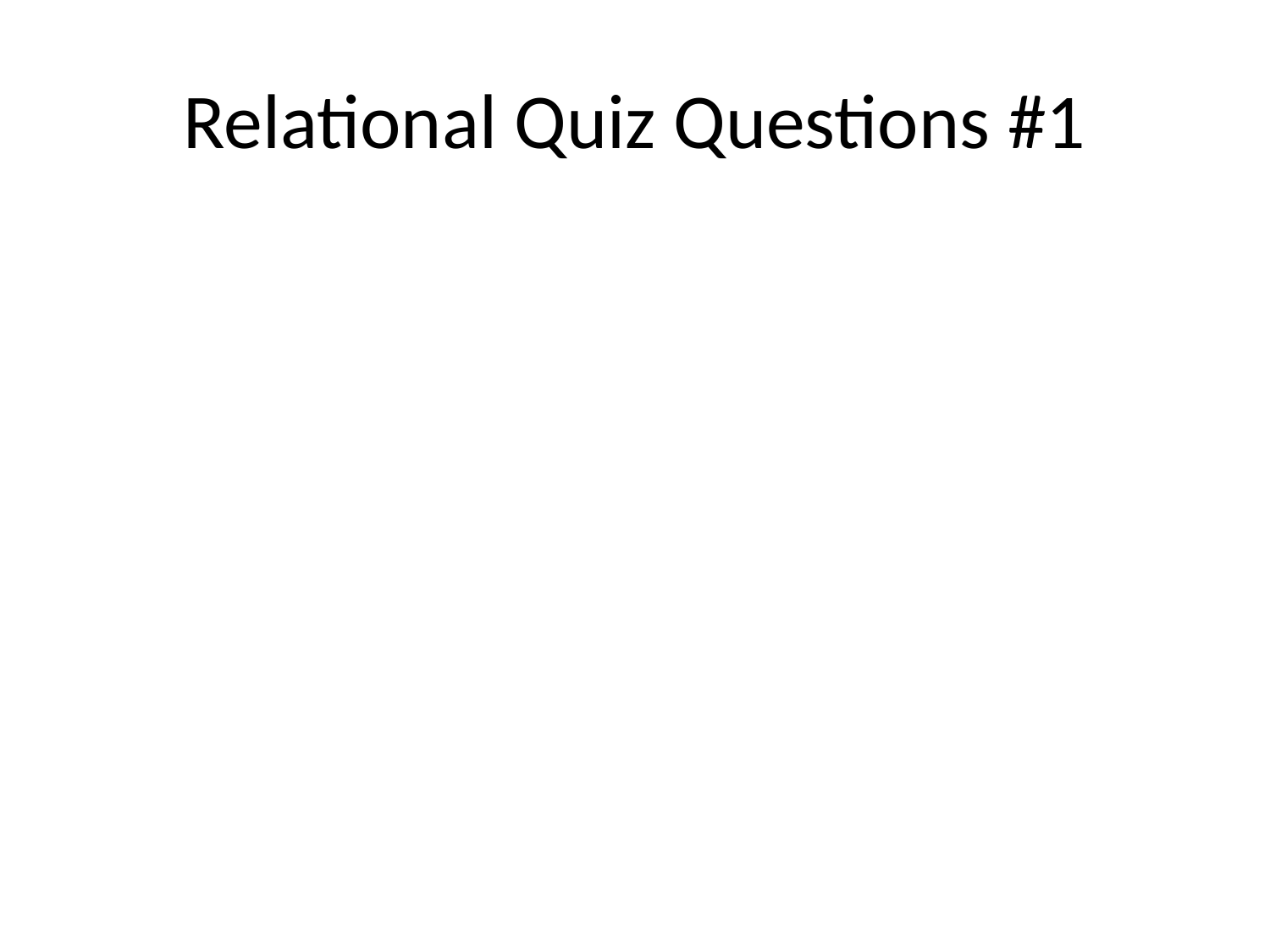

# Relational Quiz Questions #1

## Slide 53
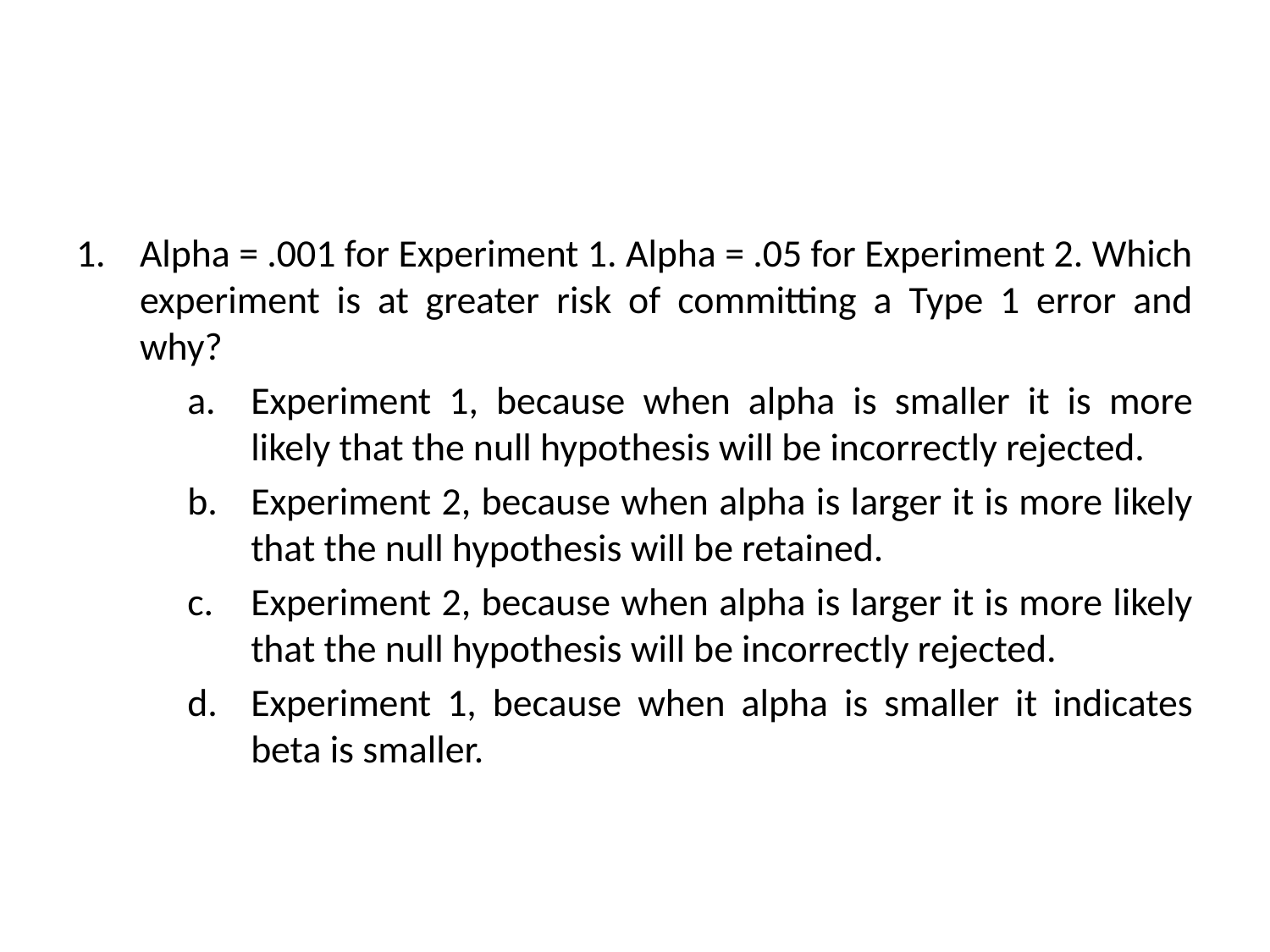

#
Alpha = .001 for Experiment 1. Alpha = .05 for Experiment 2. Which experiment is at greater risk of committing a Type 1 error and why?
Experiment 1, because when alpha is smaller it is more likely that the null hypothesis will be incorrectly rejected.
Experiment 2, because when alpha is larger it is more likely that the null hypothesis will be retained.
Experiment 2, because when alpha is larger it is more likely that the null hypothesis will be incorrectly rejected.
Experiment 1, because when alpha is smaller it indicates beta is smaller.

## Slide 54
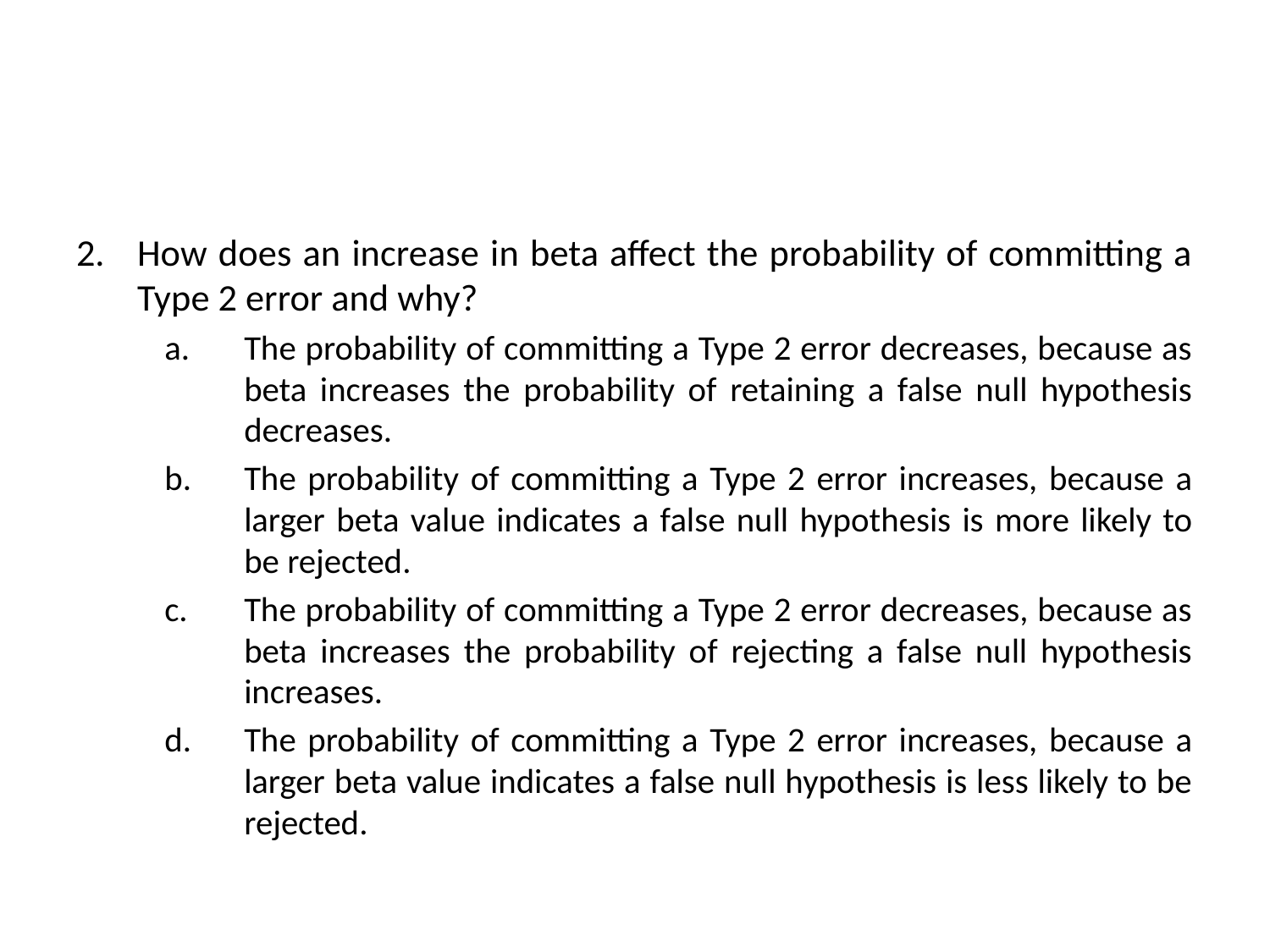

#
How does an increase in beta affect the probability of committing a Type 2 error and why?
The probability of committing a Type 2 error decreases, because as beta increases the probability of retaining a false null hypothesis decreases.
The probability of committing a Type 2 error increases, because a larger beta value indicates a false null hypothesis is more likely to be rejected.
The probability of committing a Type 2 error decreases, because as beta increases the probability of rejecting a false null hypothesis increases.
The probability of committing a Type 2 error increases, because a larger beta value indicates a false null hypothesis is less likely to be rejected.

## Slide 55
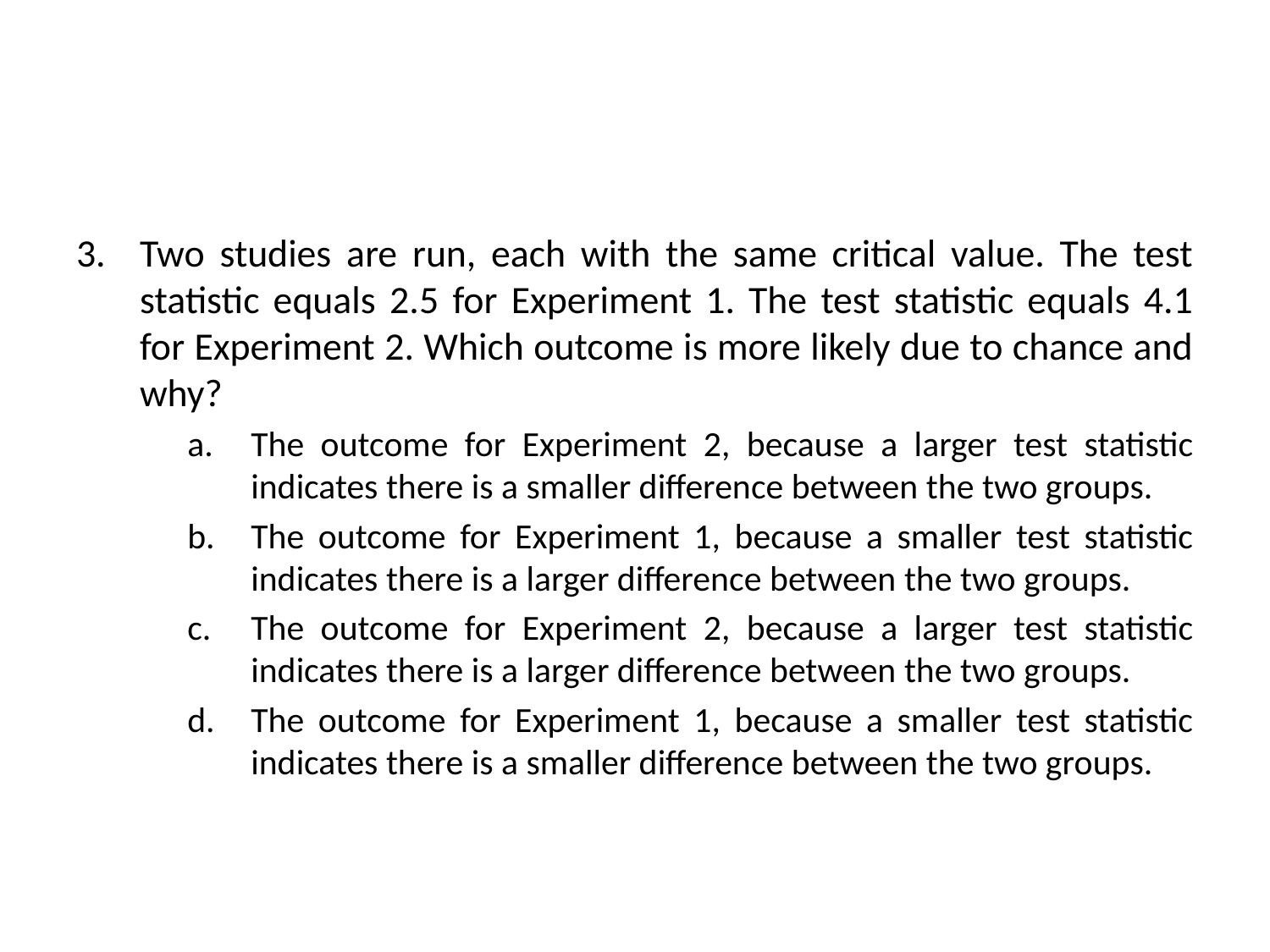

#
Two studies are run, each with the same critical value. The test statistic equals 2.5 for Experiment 1. The test statistic equals 4.1 for Experiment 2. Which outcome is more likely due to chance and why?
The outcome for Experiment 2, because a larger test statistic indicates there is a smaller difference between the two groups.
The outcome for Experiment 1, because a smaller test statistic indicates there is a larger difference between the two groups.
The outcome for Experiment 2, because a larger test statistic indicates there is a larger difference between the two groups.
The outcome for Experiment 1, because a smaller test statistic indicates there is a smaller difference between the two groups.

## Slide 56
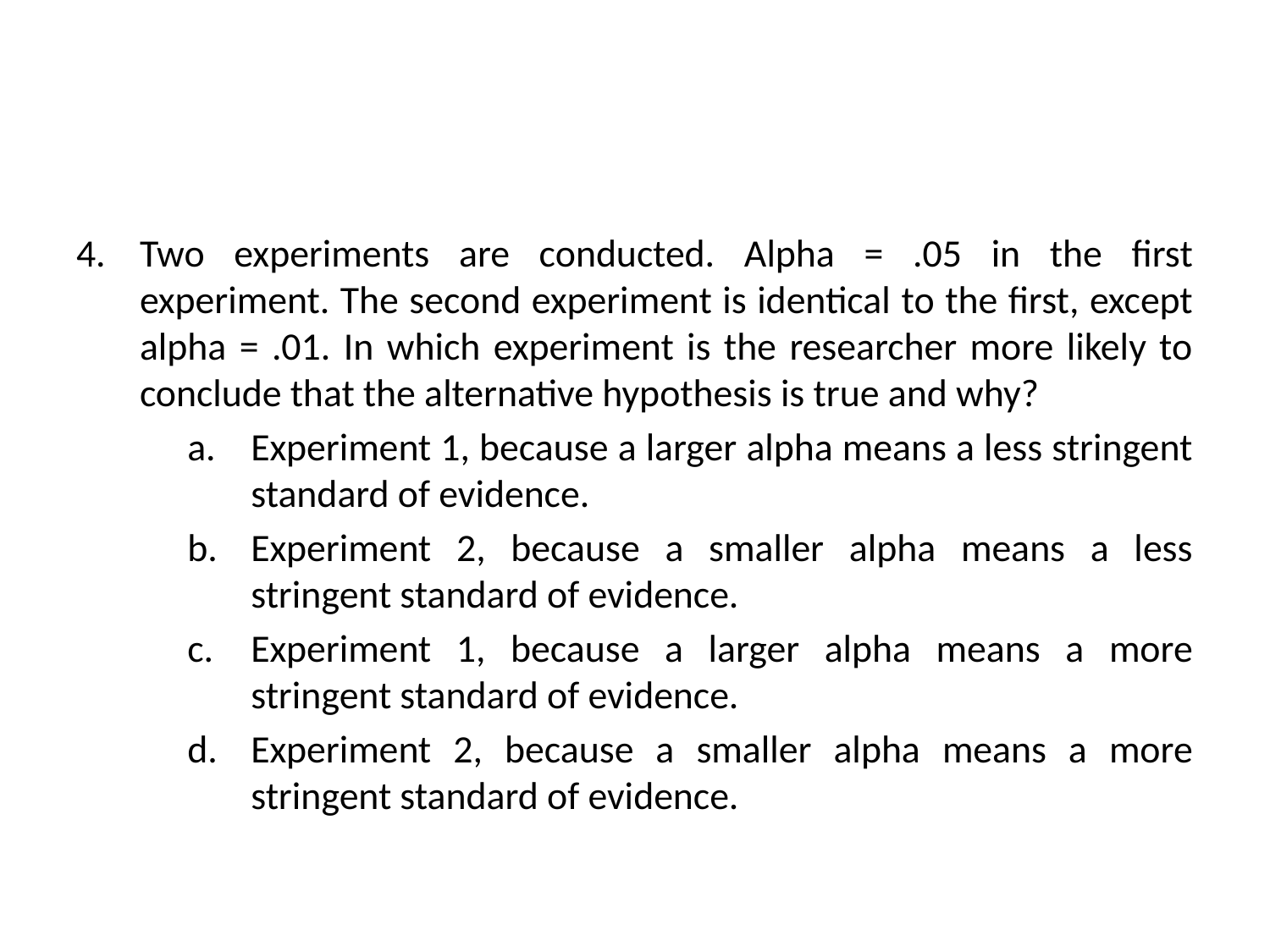

#
Two experiments are conducted. Alpha = .05 in the first experiment. The second experiment is identical to the first, except alpha = .01. In which experiment is the researcher more likely to conclude that the alternative hypothesis is true and why?
Experiment 1, because a larger alpha means a less stringent standard of evidence.
Experiment 2, because a smaller alpha means a less stringent standard of evidence.
Experiment 1, because a larger alpha means a more stringent standard of evidence.
Experiment 2, because a smaller alpha means a more stringent standard of evidence.

## Slide 57
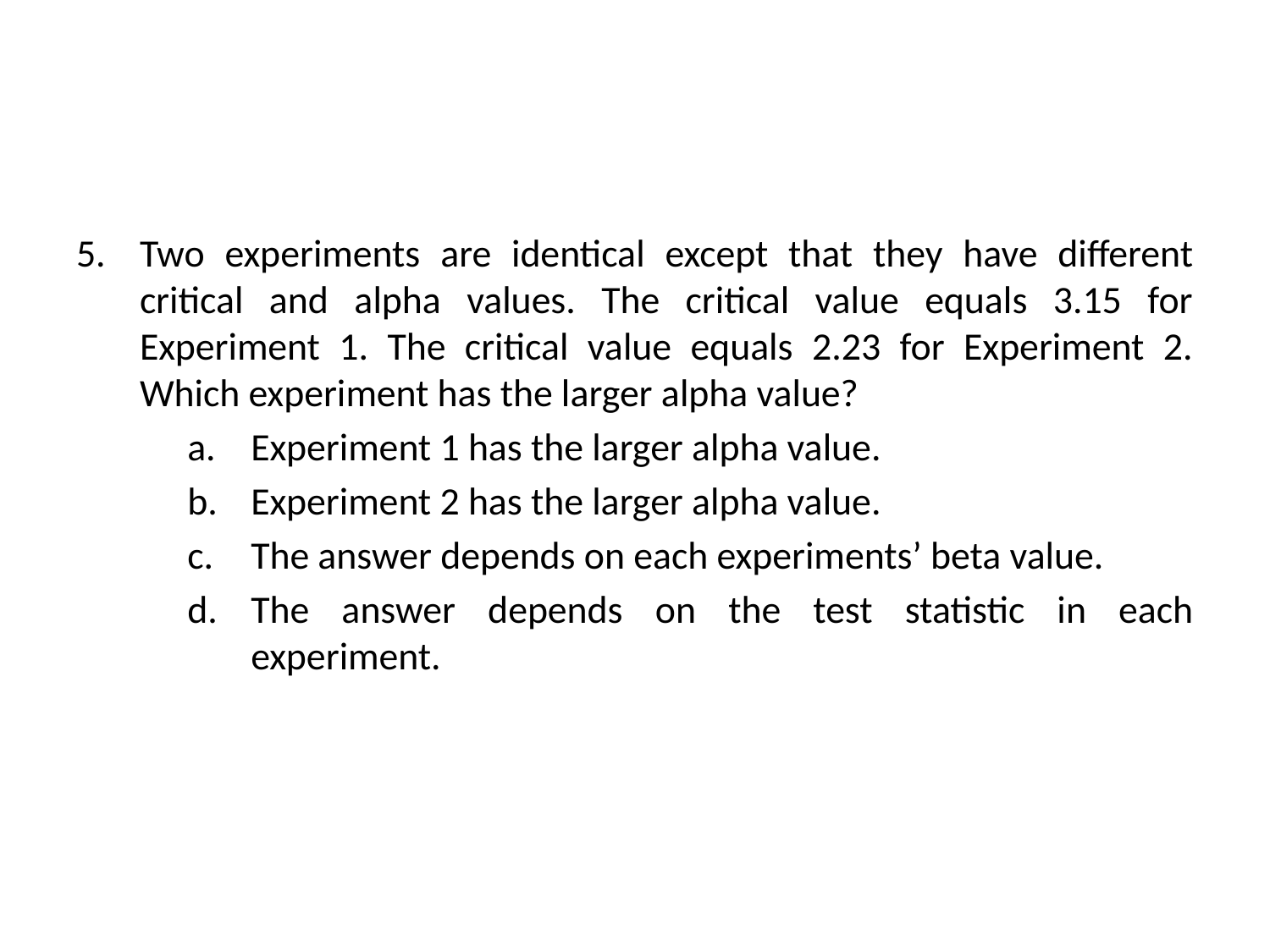

#
Two experiments are identical except that they have different critical and alpha values. The critical value equals 3.15 for Experiment 1. The critical value equals 2.23 for Experiment 2. Which experiment has the larger alpha value?
Experiment 1 has the larger alpha value.
Experiment 2 has the larger alpha value.
The answer depends on each experiments’ beta value.
The answer depends on the test statistic in each experiment.

## Slide 58
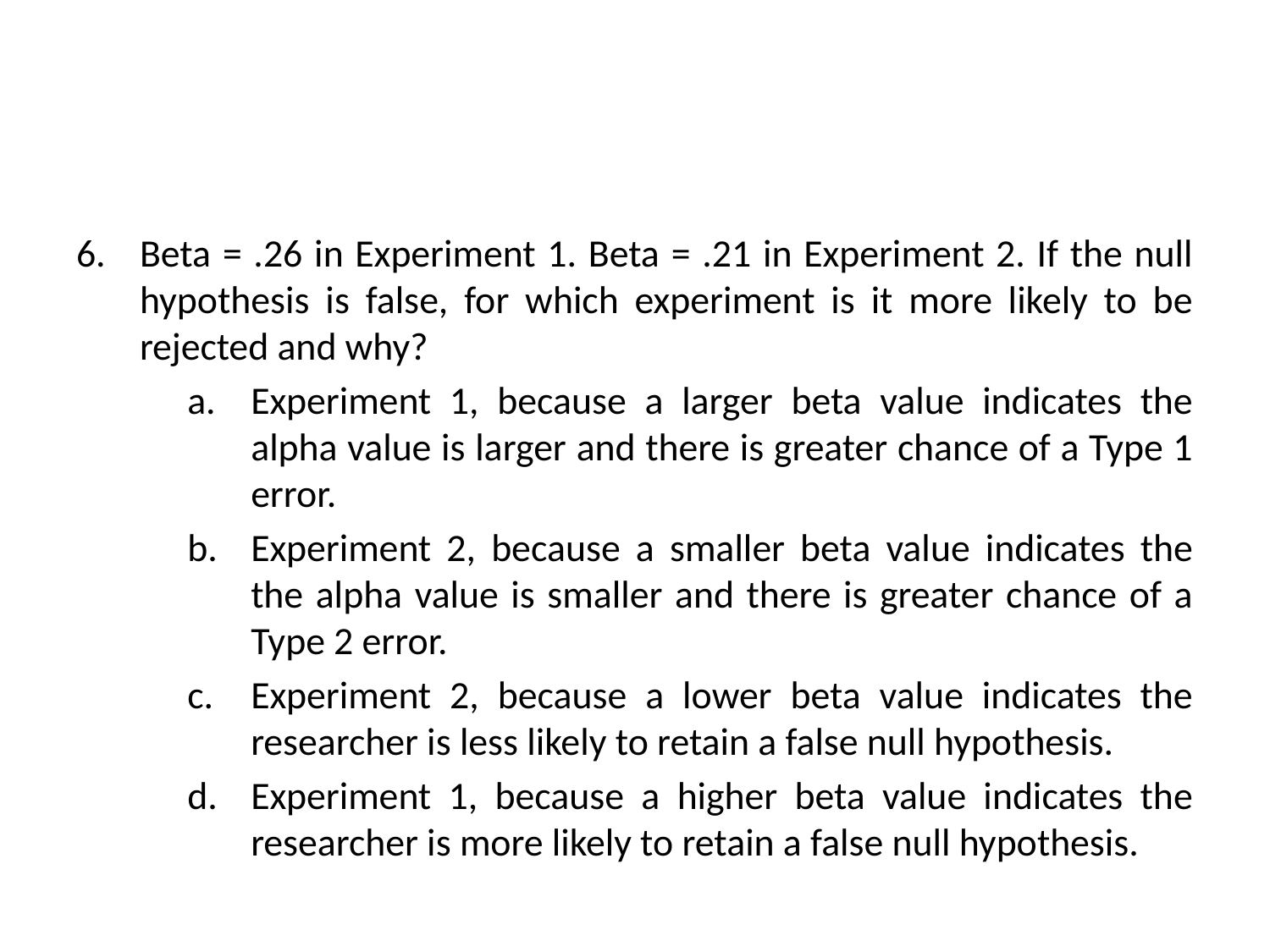

#
Beta = .26 in Experiment 1. Beta = .21 in Experiment 2. If the null hypothesis is false, for which experiment is it more likely to be rejected and why?
Experiment 1, because a larger beta value indicates the alpha value is larger and there is greater chance of a Type 1 error.
Experiment 2, because a smaller beta value indicates the the alpha value is smaller and there is greater chance of a Type 2 error.
Experiment 2, because a lower beta value indicates the researcher is less likely to retain a false null hypothesis.
Experiment 1, because a higher beta value indicates the researcher is more likely to retain a false null hypothesis.

## Slide 59
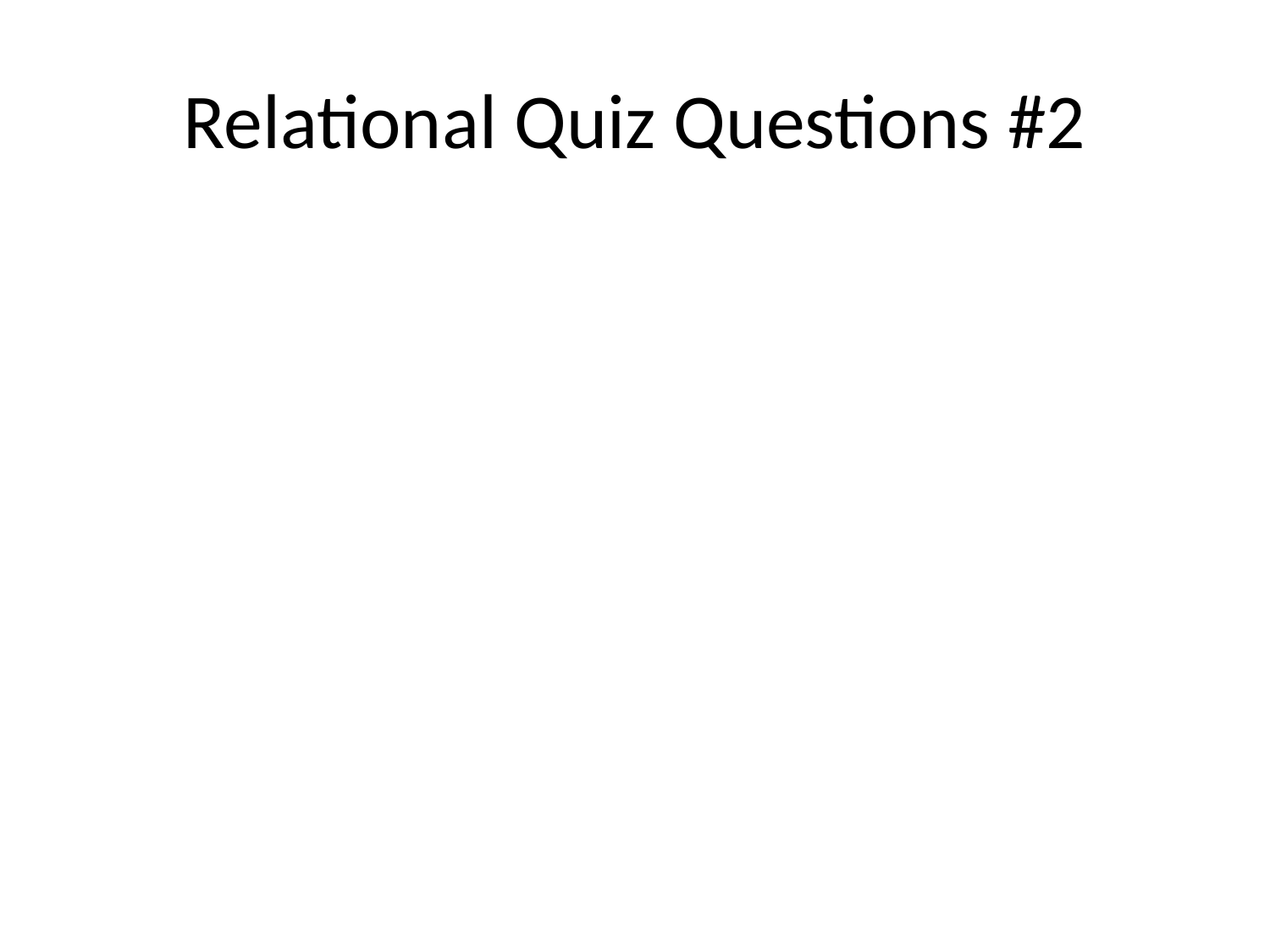

# Relational Quiz Questions #2

## Slide 60
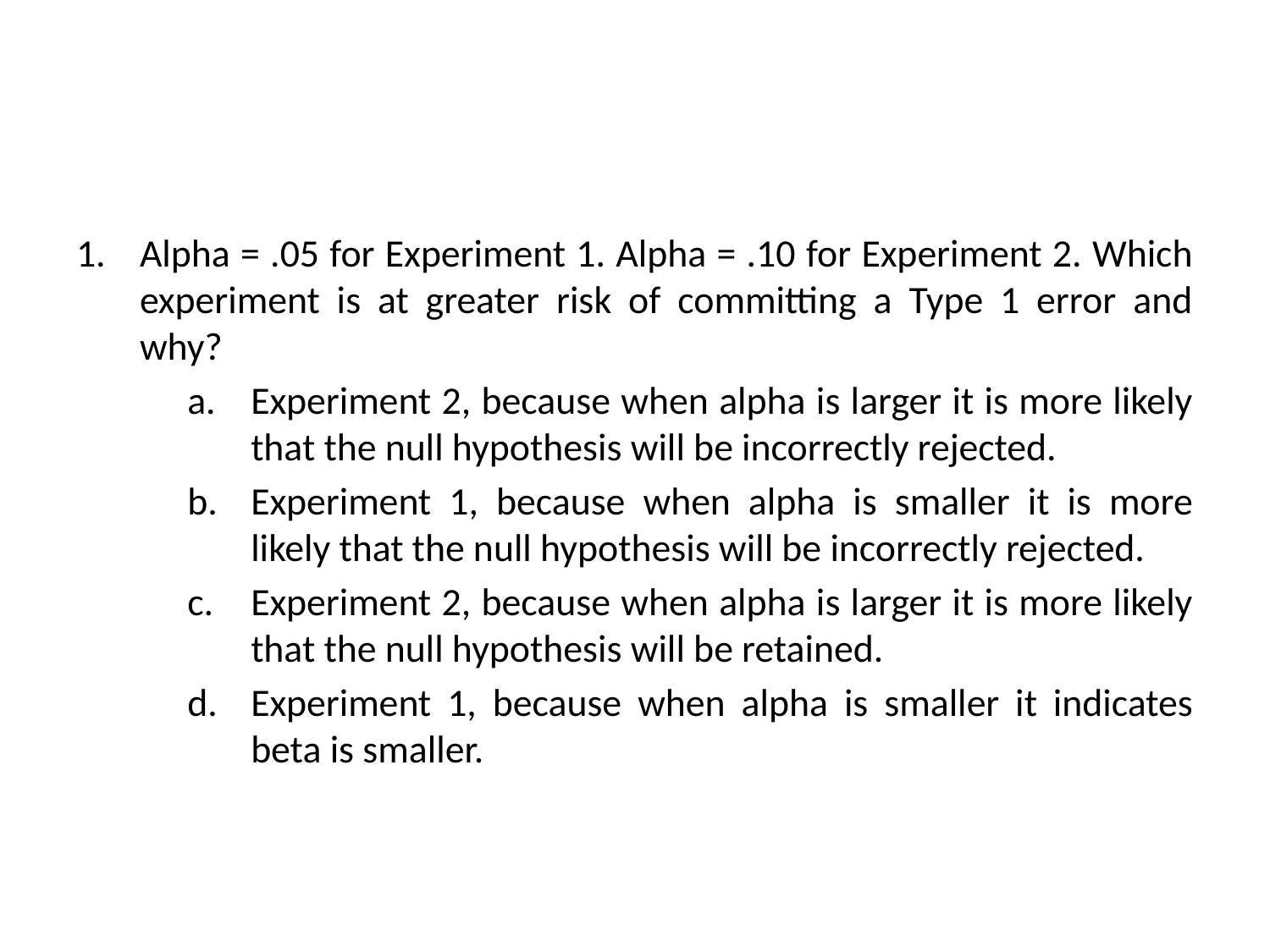

#
Alpha = .05 for Experiment 1. Alpha = .10 for Experiment 2. Which experiment is at greater risk of committing a Type 1 error and why?
Experiment 2, because when alpha is larger it is more likely that the null hypothesis will be incorrectly rejected.
Experiment 1, because when alpha is smaller it is more likely that the null hypothesis will be incorrectly rejected.
Experiment 2, because when alpha is larger it is more likely that the null hypothesis will be retained.
Experiment 1, because when alpha is smaller it indicates beta is smaller.

## Slide 61
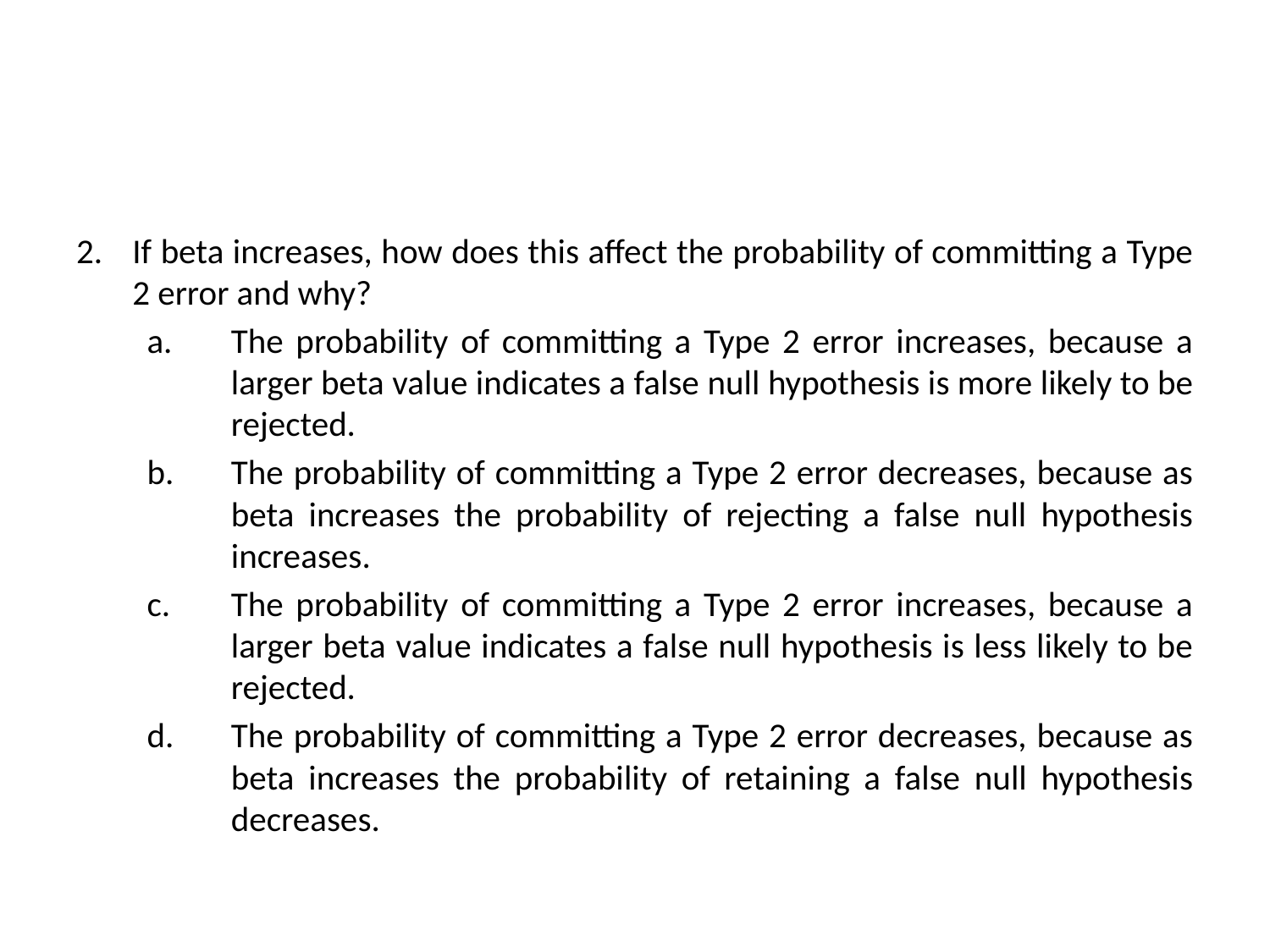

#
If beta increases, how does this affect the probability of committing a Type 2 error and why?
The probability of committing a Type 2 error increases, because a larger beta value indicates a false null hypothesis is more likely to be rejected.
The probability of committing a Type 2 error decreases, because as beta increases the probability of rejecting a false null hypothesis increases.
The probability of committing a Type 2 error increases, because a larger beta value indicates a false null hypothesis is less likely to be rejected.
The probability of committing a Type 2 error decreases, because as beta increases the probability of retaining a false null hypothesis decreases.

## Slide 62
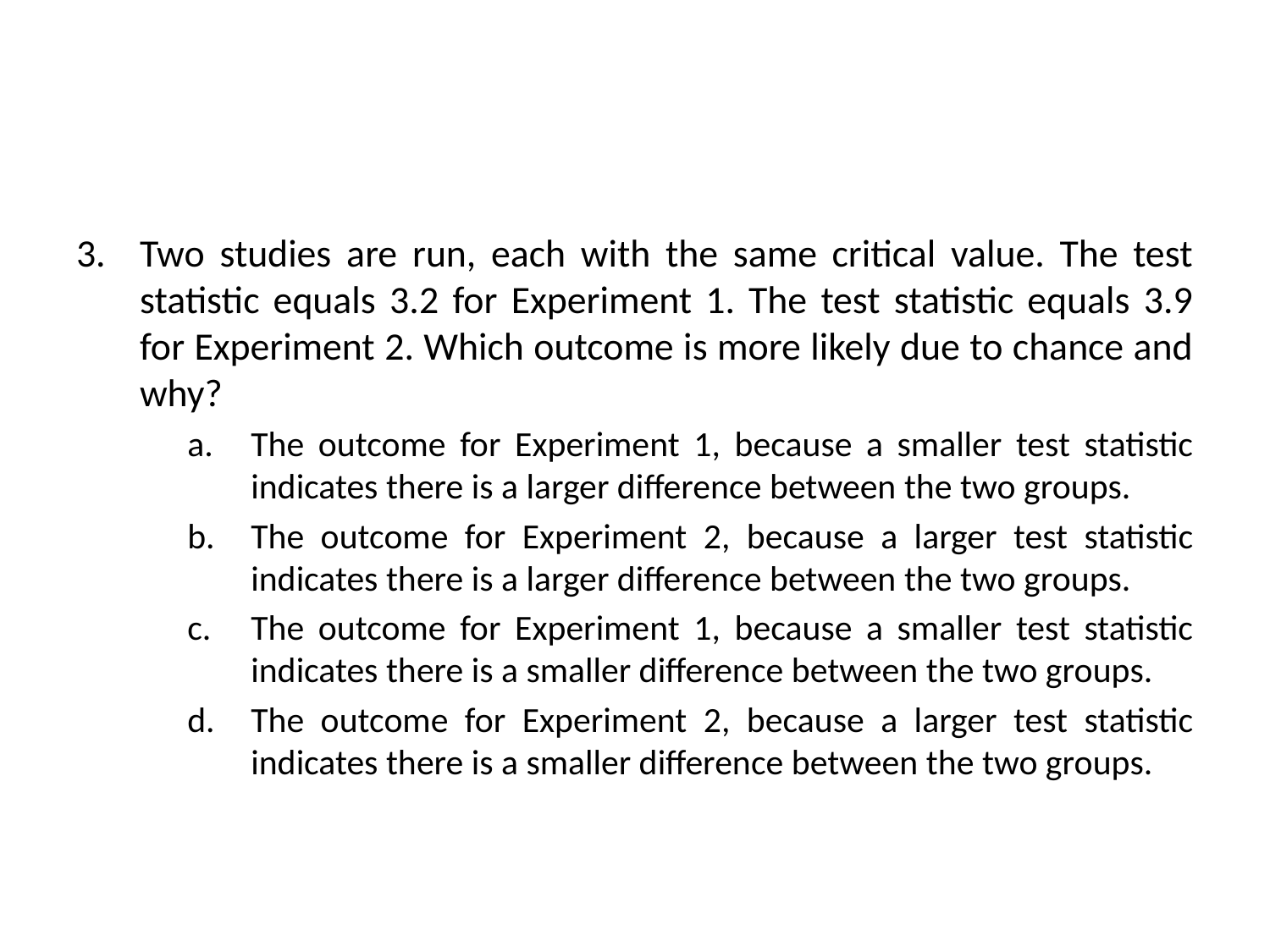

#
Two studies are run, each with the same critical value. The test statistic equals 3.2 for Experiment 1. The test statistic equals 3.9 for Experiment 2. Which outcome is more likely due to chance and why?
The outcome for Experiment 1, because a smaller test statistic indicates there is a larger difference between the two groups.
The outcome for Experiment 2, because a larger test statistic indicates there is a larger difference between the two groups.
The outcome for Experiment 1, because a smaller test statistic indicates there is a smaller difference between the two groups.
The outcome for Experiment 2, because a larger test statistic indicates there is a smaller difference between the two groups.

## Slide 63
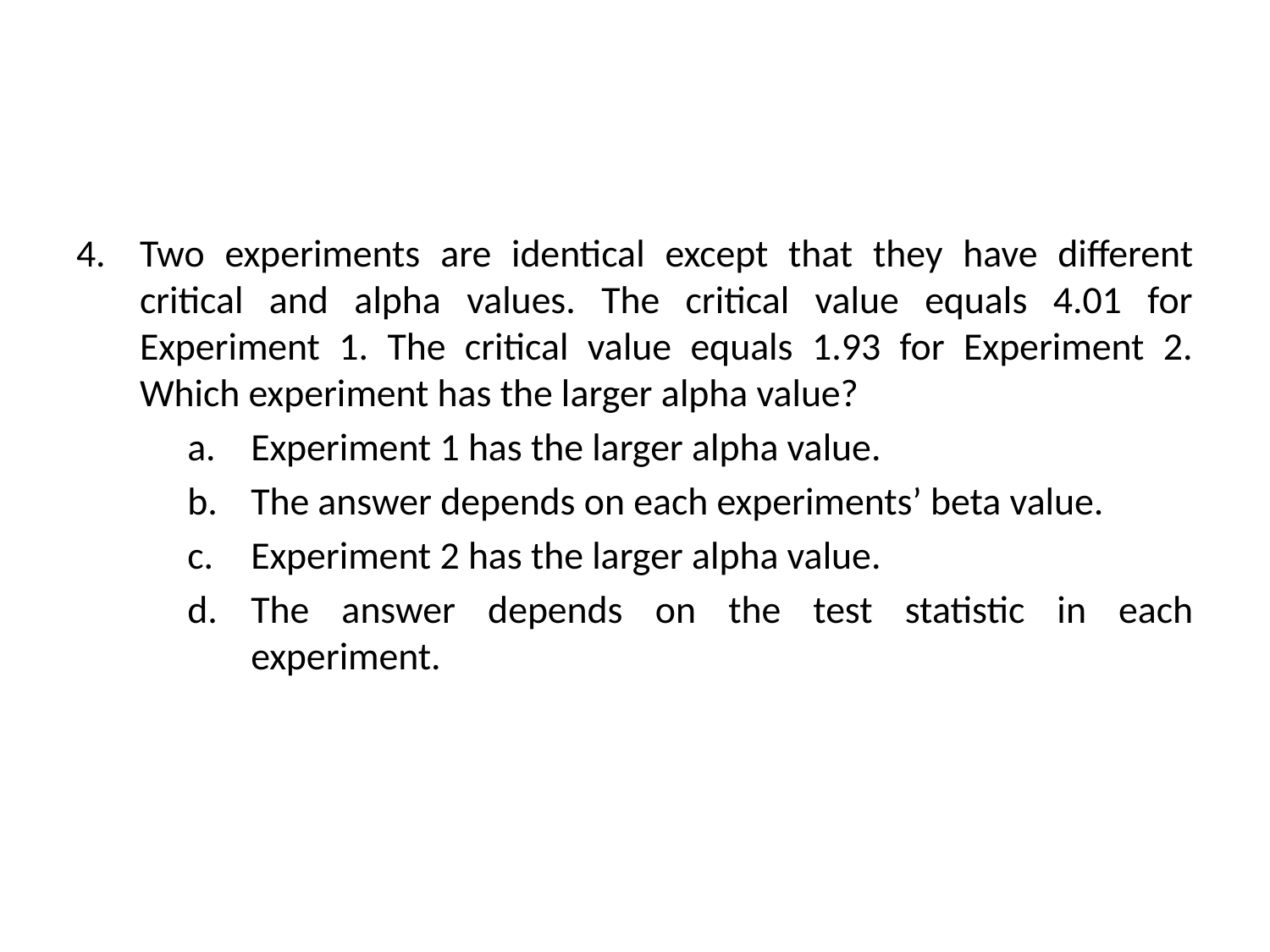

#
Two experiments are identical except that they have different critical and alpha values. The critical value equals 4.01 for Experiment 1. The critical value equals 1.93 for Experiment 2. Which experiment has the larger alpha value?
Experiment 1 has the larger alpha value.
The answer depends on each experiments’ beta value.
Experiment 2 has the larger alpha value.
The answer depends on the test statistic in each experiment.

## Slide 64
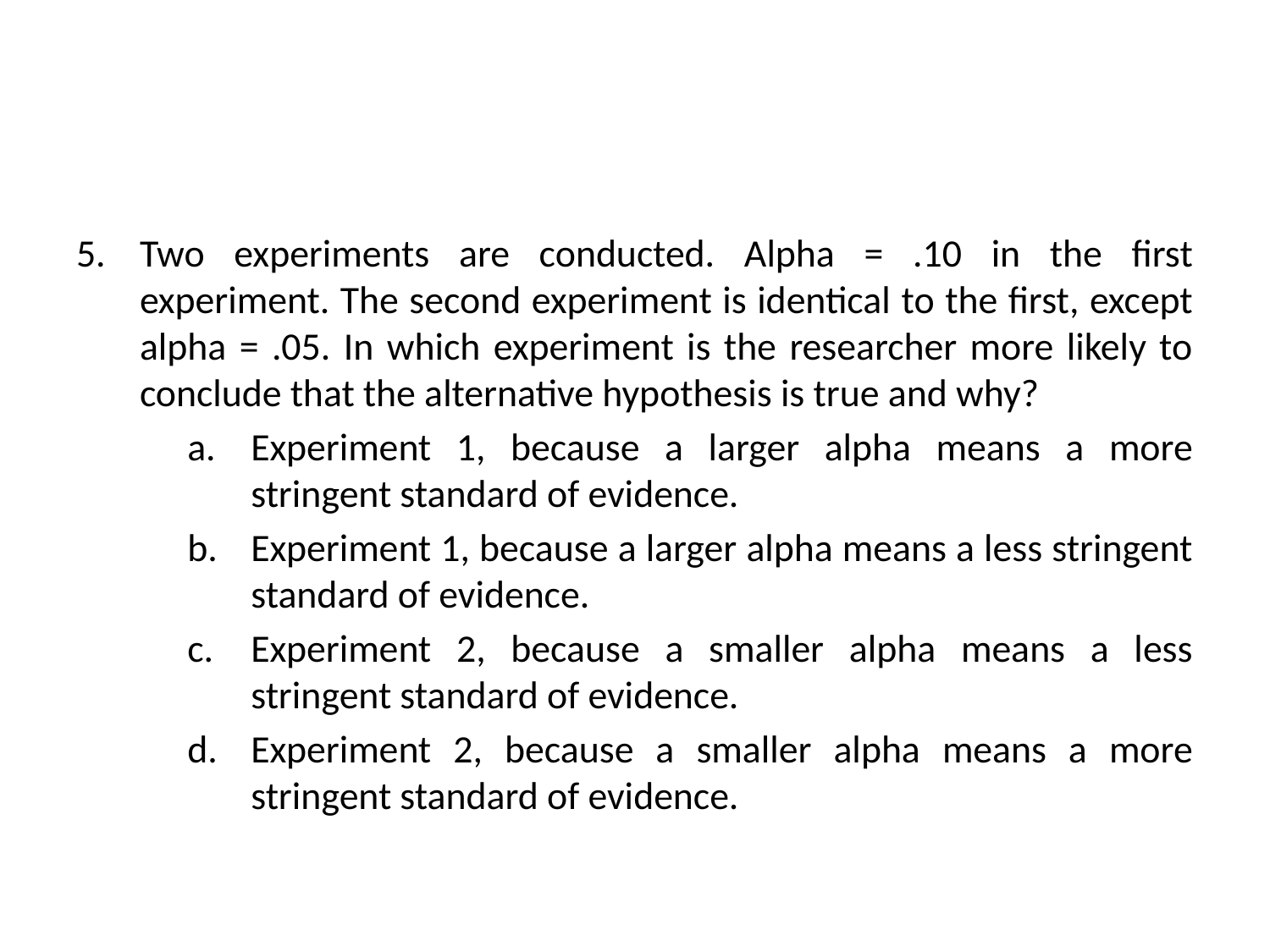

#
Two experiments are conducted. Alpha = .10 in the first experiment. The second experiment is identical to the first, except alpha = .05. In which experiment is the researcher more likely to conclude that the alternative hypothesis is true and why?
Experiment 1, because a larger alpha means a more stringent standard of evidence.
Experiment 1, because a larger alpha means a less stringent standard of evidence.
Experiment 2, because a smaller alpha means a less stringent standard of evidence.
Experiment 2, because a smaller alpha means a more stringent standard of evidence.

## Slide 65
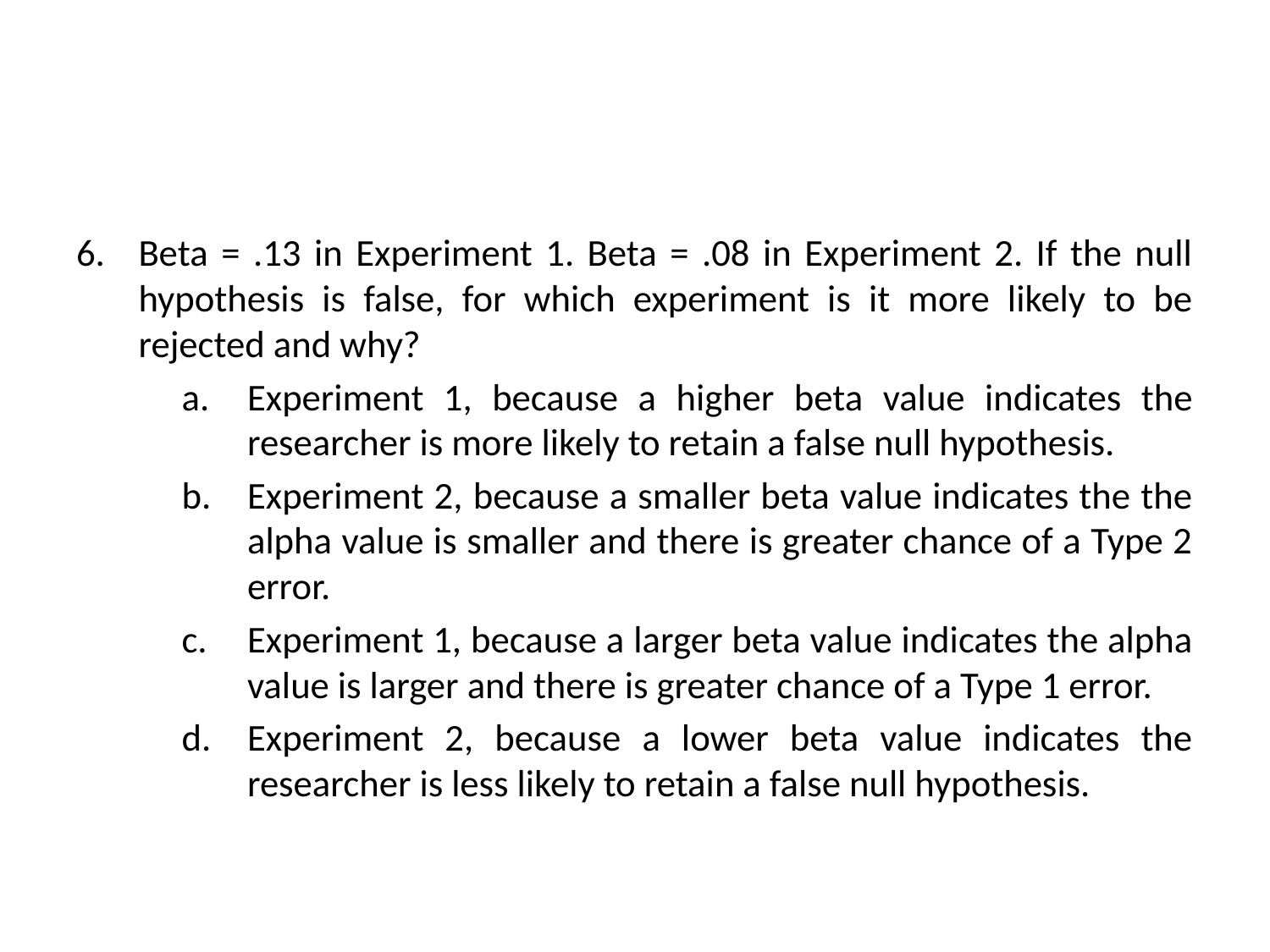

#
Beta = .13 in Experiment 1. Beta = .08 in Experiment 2. If the null hypothesis is false, for which experiment is it more likely to be rejected and why?
Experiment 1, because a higher beta value indicates the researcher is more likely to retain a false null hypothesis.
Experiment 2, because a smaller beta value indicates the the alpha value is smaller and there is greater chance of a Type 2 error.
Experiment 1, because a larger beta value indicates the alpha value is larger and there is greater chance of a Type 1 error.
Experiment 2, because a lower beta value indicates the researcher is less likely to retain a false null hypothesis.

## Slide 66
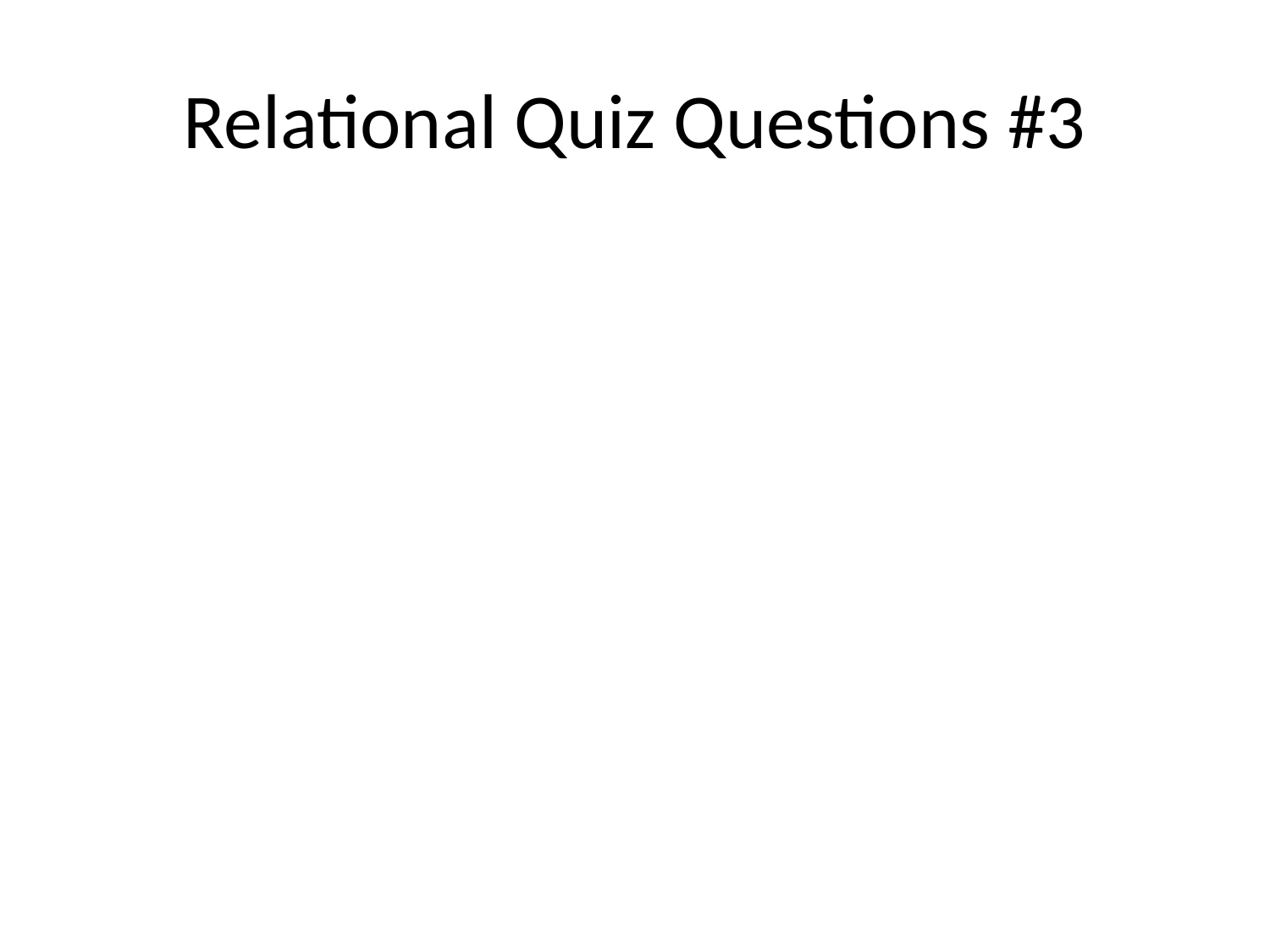

# Relational Quiz Questions #3

## Slide 67
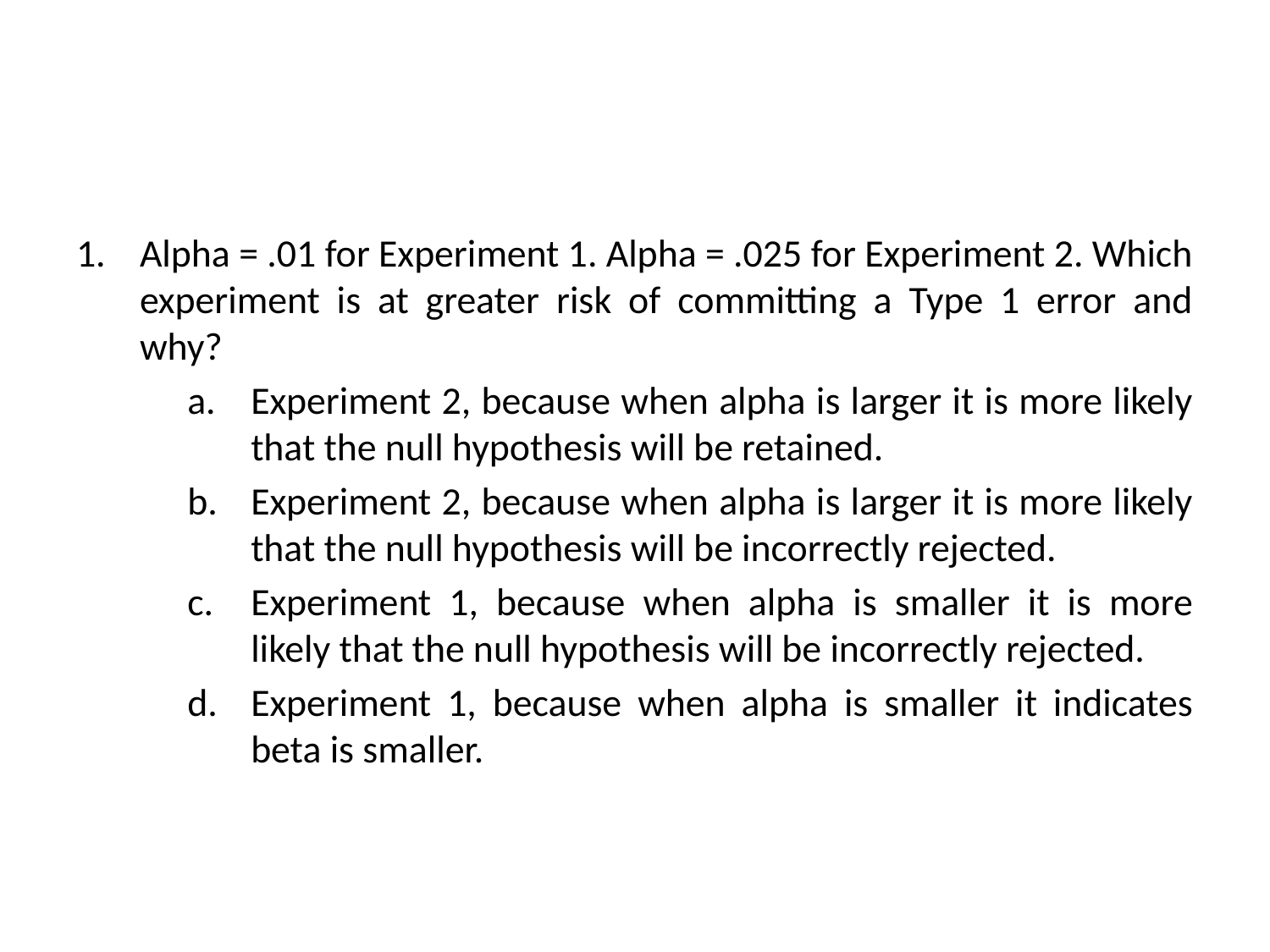

#
Alpha = .01 for Experiment 1. Alpha = .025 for Experiment 2. Which experiment is at greater risk of committing a Type 1 error and why?
Experiment 2, because when alpha is larger it is more likely that the null hypothesis will be retained.
Experiment 2, because when alpha is larger it is more likely that the null hypothesis will be incorrectly rejected.
Experiment 1, because when alpha is smaller it is more likely that the null hypothesis will be incorrectly rejected.
Experiment 1, because when alpha is smaller it indicates beta is smaller.

## Slide 68
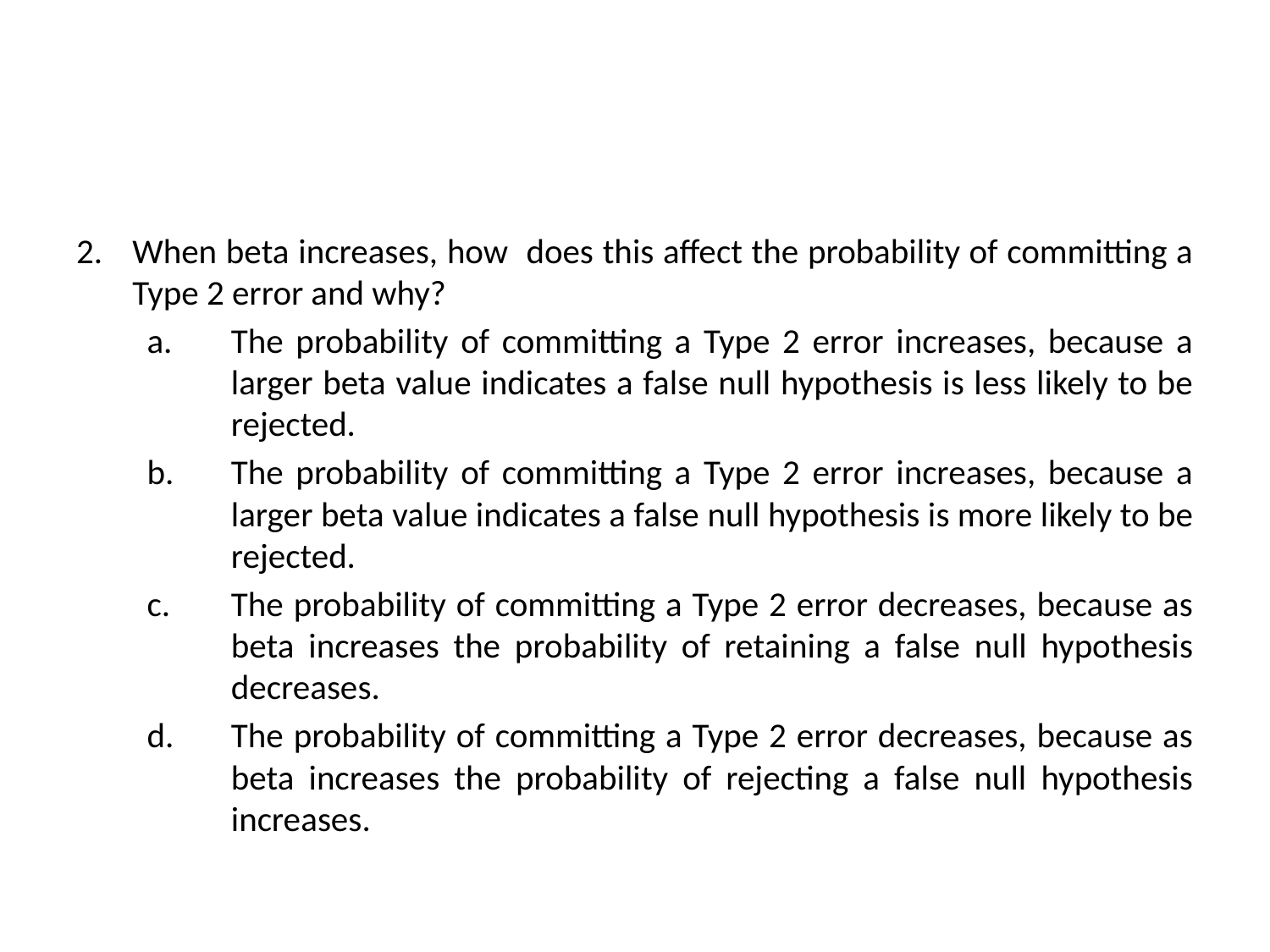

#
When beta increases, how does this affect the probability of committing a Type 2 error and why?
The probability of committing a Type 2 error increases, because a larger beta value indicates a false null hypothesis is less likely to be rejected.
The probability of committing a Type 2 error increases, because a larger beta value indicates a false null hypothesis is more likely to be rejected.
The probability of committing a Type 2 error decreases, because as beta increases the probability of retaining a false null hypothesis decreases.
The probability of committing a Type 2 error decreases, because as beta increases the probability of rejecting a false null hypothesis increases.

## Slide 69
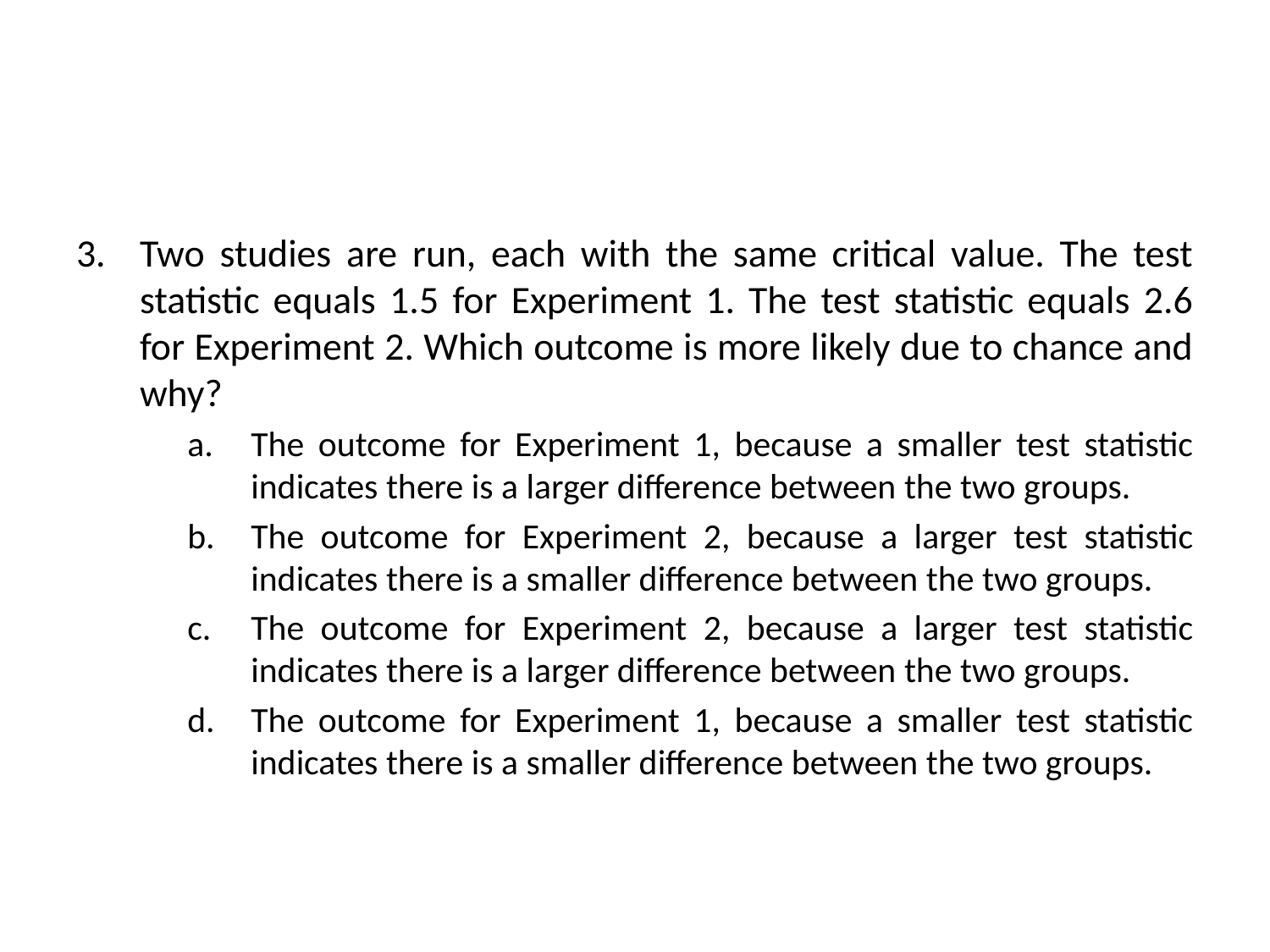

#
Two studies are run, each with the same critical value. The test statistic equals 1.5 for Experiment 1. The test statistic equals 2.6 for Experiment 2. Which outcome is more likely due to chance and why?
The outcome for Experiment 1, because a smaller test statistic indicates there is a larger difference between the two groups.
The outcome for Experiment 2, because a larger test statistic indicates there is a smaller difference between the two groups.
The outcome for Experiment 2, because a larger test statistic indicates there is a larger difference between the two groups.
The outcome for Experiment 1, because a smaller test statistic indicates there is a smaller difference between the two groups.

## Slide 70
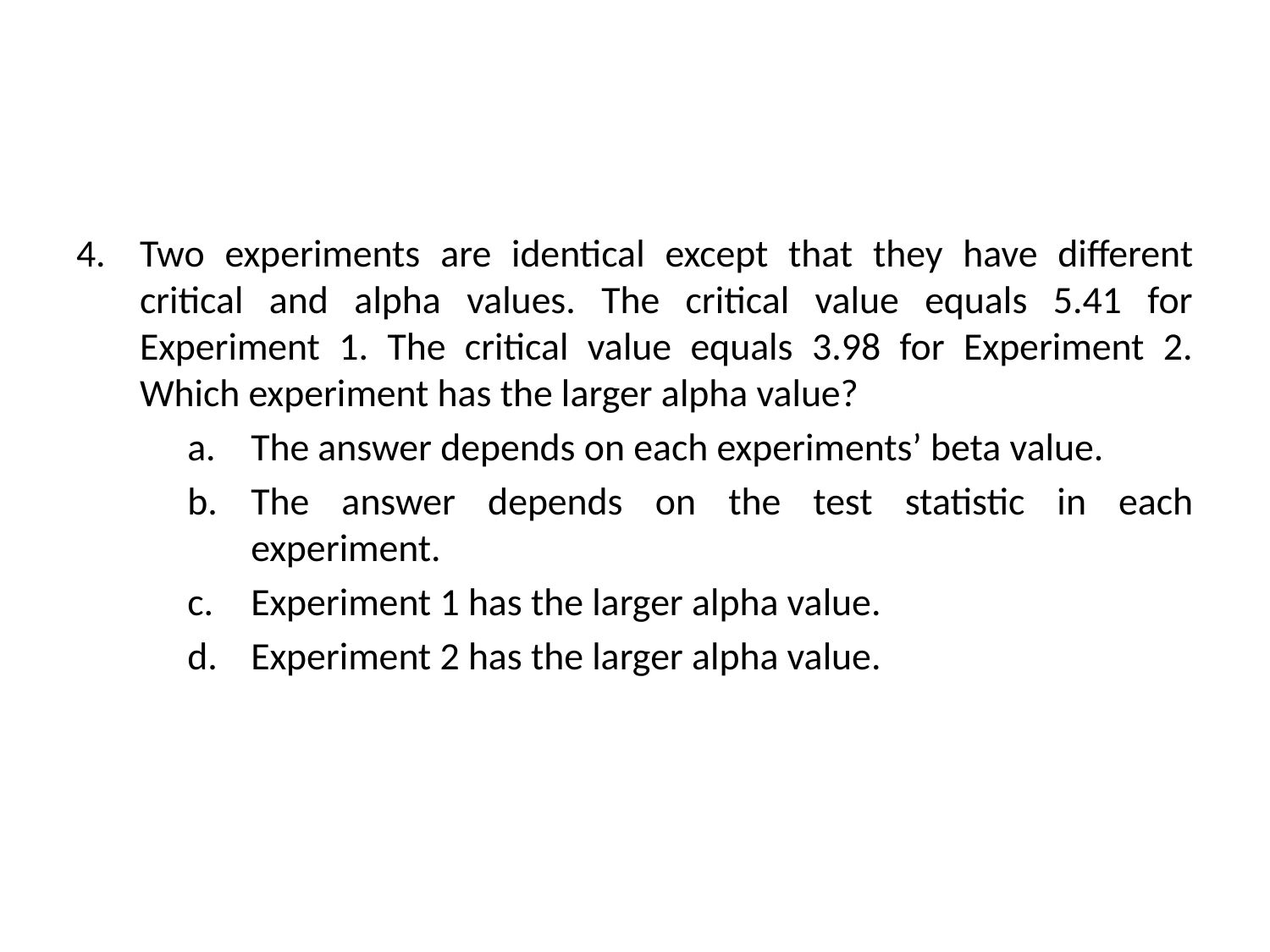

#
Two experiments are identical except that they have different critical and alpha values. The critical value equals 5.41 for Experiment 1. The critical value equals 3.98 for Experiment 2. Which experiment has the larger alpha value?
The answer depends on each experiments’ beta value.
The answer depends on the test statistic in each experiment.
Experiment 1 has the larger alpha value.
Experiment 2 has the larger alpha value.

## Slide 71
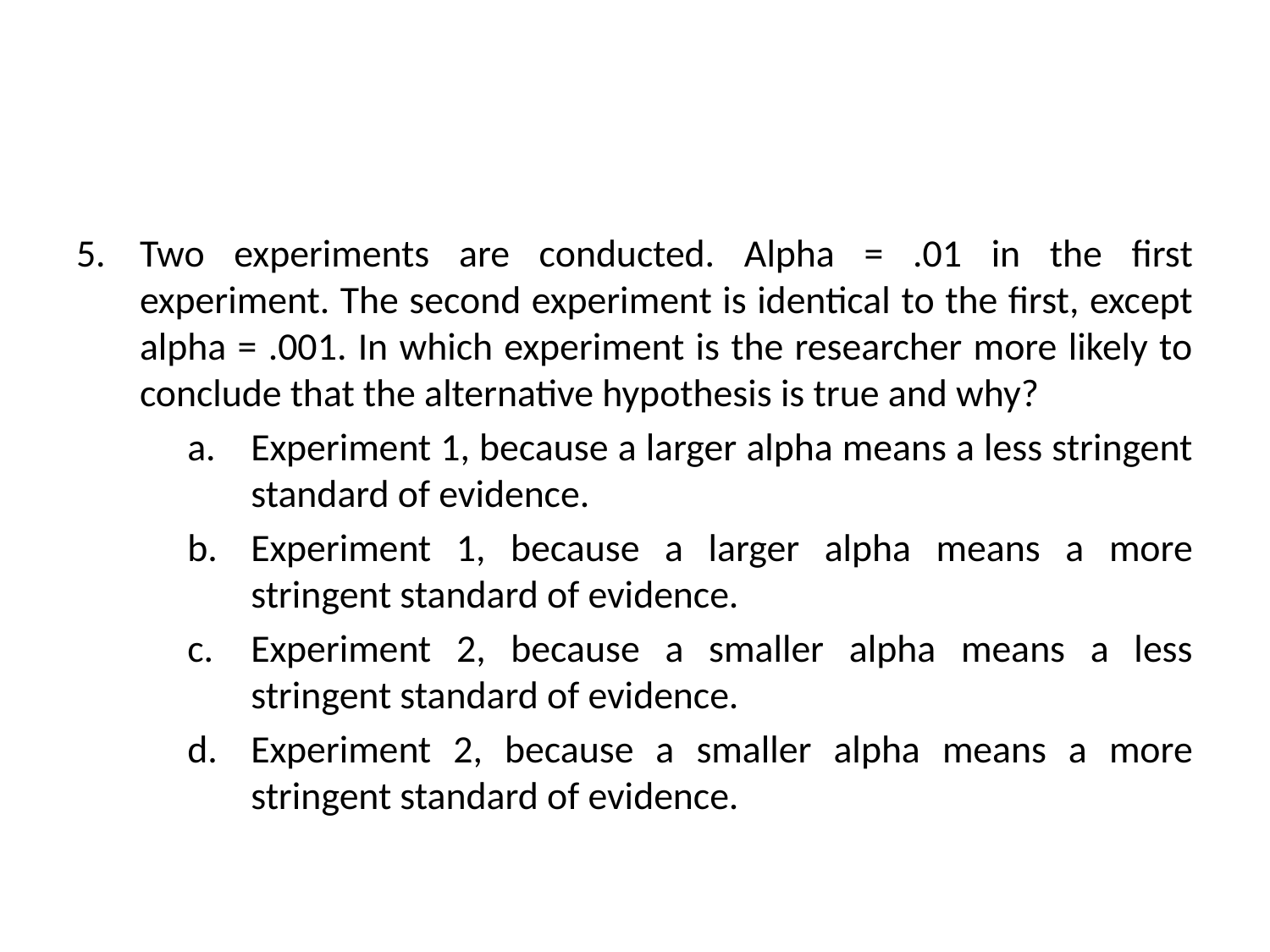

#
Two experiments are conducted. Alpha = .01 in the first experiment. The second experiment is identical to the first, except alpha = .001. In which experiment is the researcher more likely to conclude that the alternative hypothesis is true and why?
Experiment 1, because a larger alpha means a less stringent standard of evidence.
Experiment 1, because a larger alpha means a more stringent standard of evidence.
Experiment 2, because a smaller alpha means a less stringent standard of evidence.
Experiment 2, because a smaller alpha means a more stringent standard of evidence.

## Slide 72
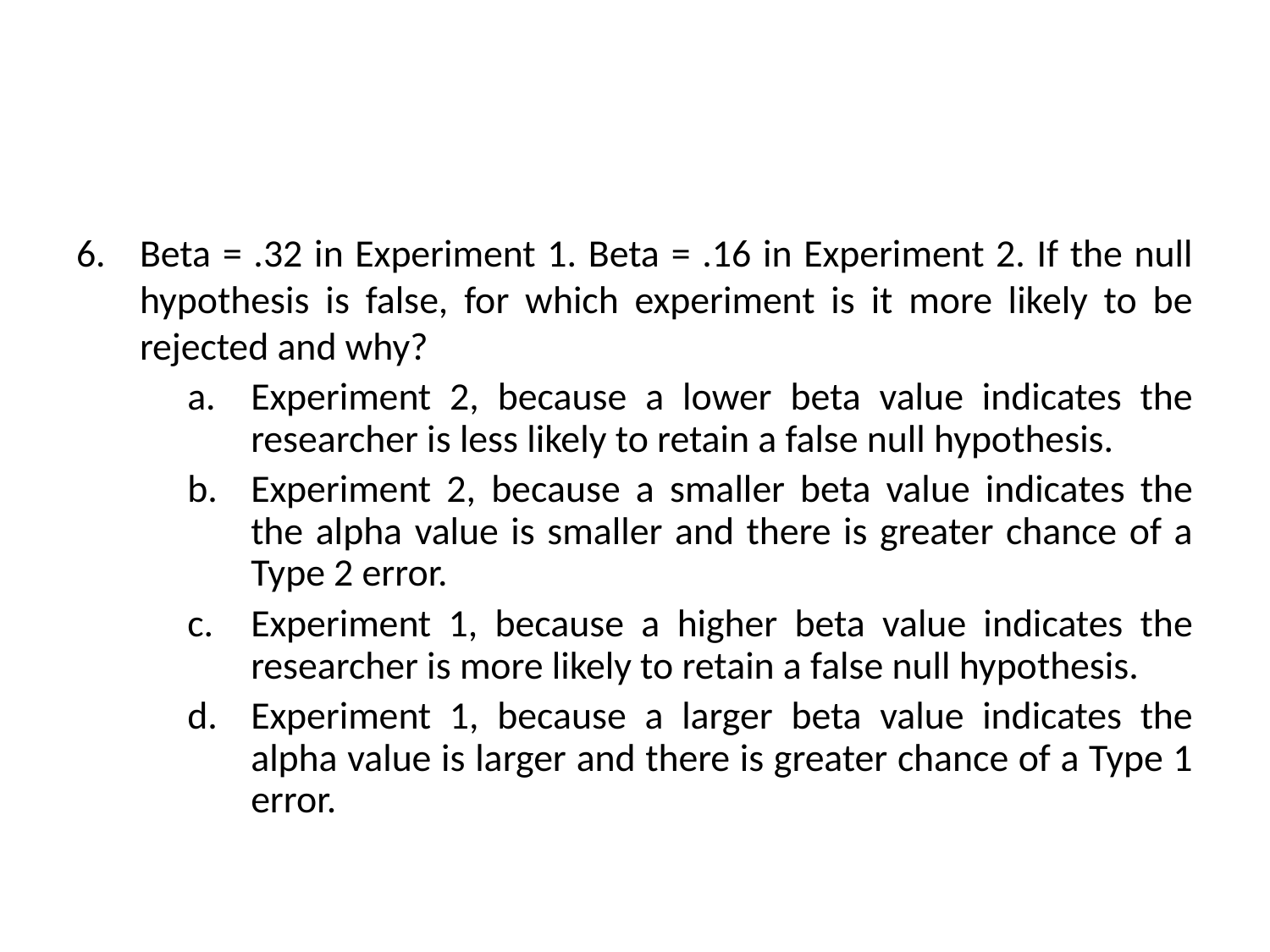

#
Beta = .32 in Experiment 1. Beta = .16 in Experiment 2. If the null hypothesis is false, for which experiment is it more likely to be rejected and why?
Experiment 2, because a lower beta value indicates the researcher is less likely to retain a false null hypothesis.
Experiment 2, because a smaller beta value indicates the the alpha value is smaller and there is greater chance of a Type 2 error.
Experiment 1, because a higher beta value indicates the researcher is more likely to retain a false null hypothesis.
Experiment 1, because a larger beta value indicates the alpha value is larger and there is greater chance of a Type 1 error.

## Slide 73
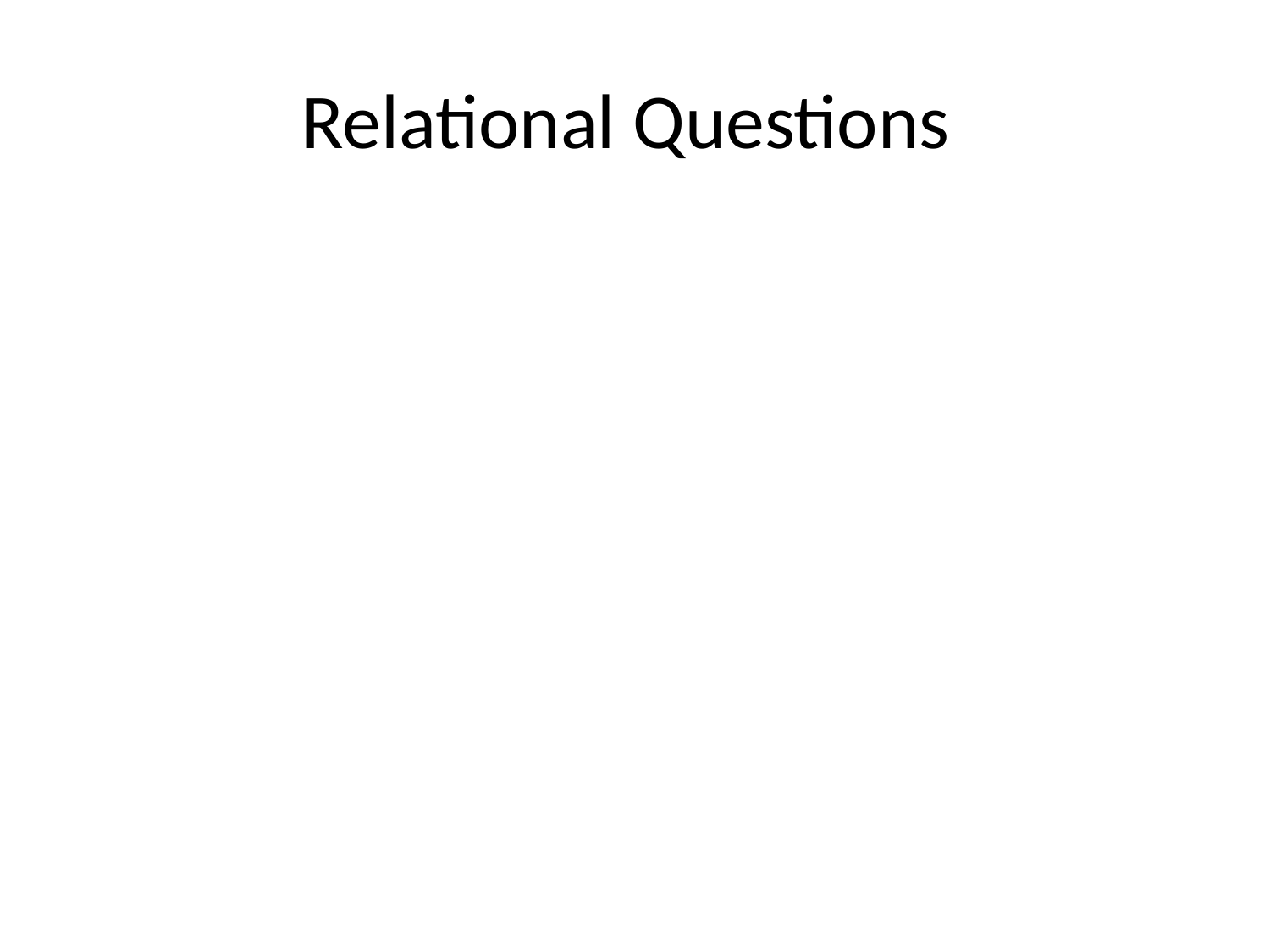

# Relational Questions

## Slide 74
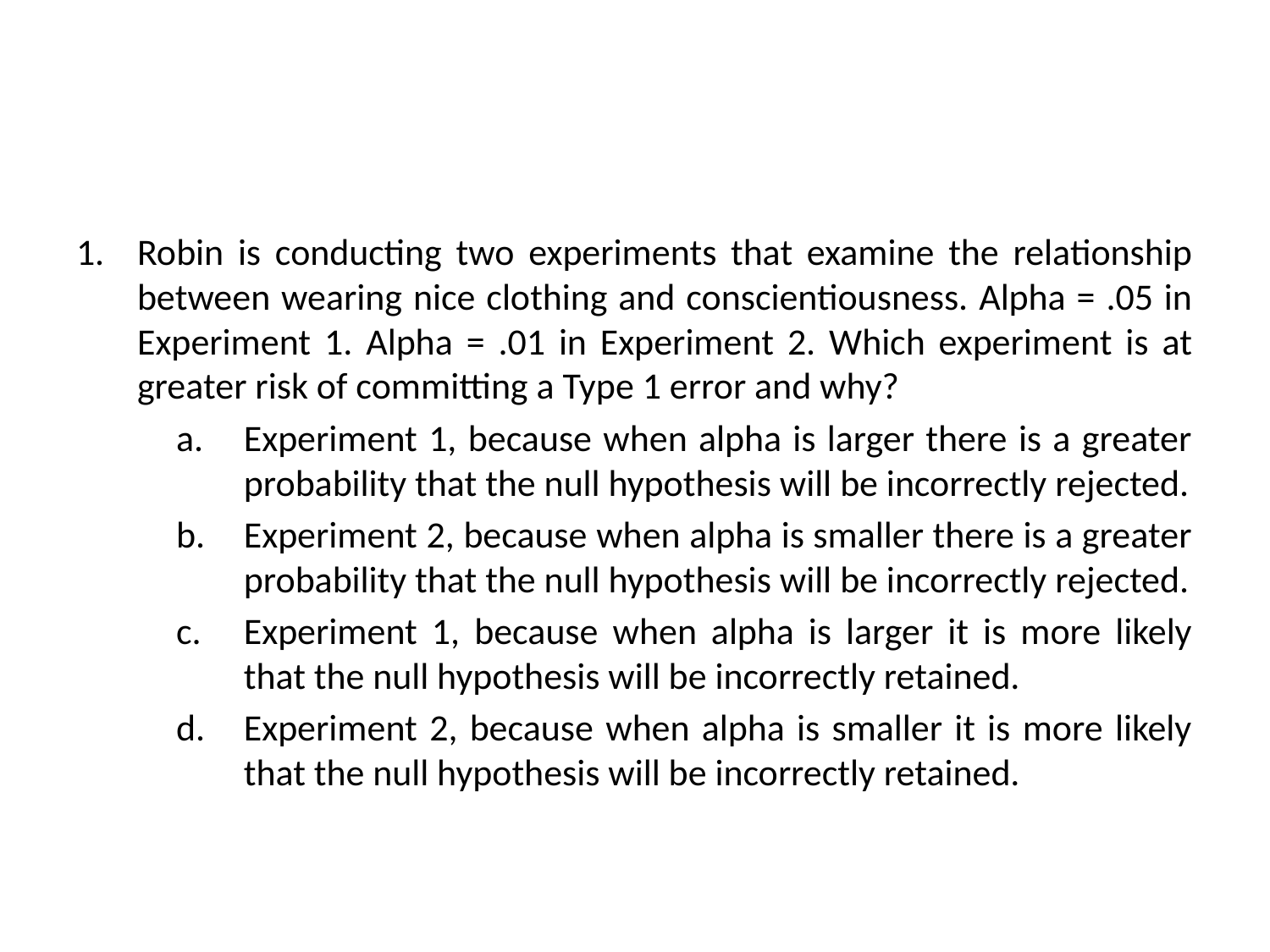

#
Robin is conducting two experiments that examine the relationship between wearing nice clothing and conscientiousness. Alpha = .05 in Experiment 1. Alpha = .01 in Experiment 2. Which experiment is at greater risk of committing a Type 1 error and why?
Experiment 1, because when alpha is larger there is a greater probability that the null hypothesis will be incorrectly rejected.
Experiment 2, because when alpha is smaller there is a greater probability that the null hypothesis will be incorrectly rejected.
Experiment 1, because when alpha is larger it is more likely that the null hypothesis will be incorrectly retained.
Experiment 2, because when alpha is smaller it is more likely that the null hypothesis will be incorrectly retained.

## Slide 75
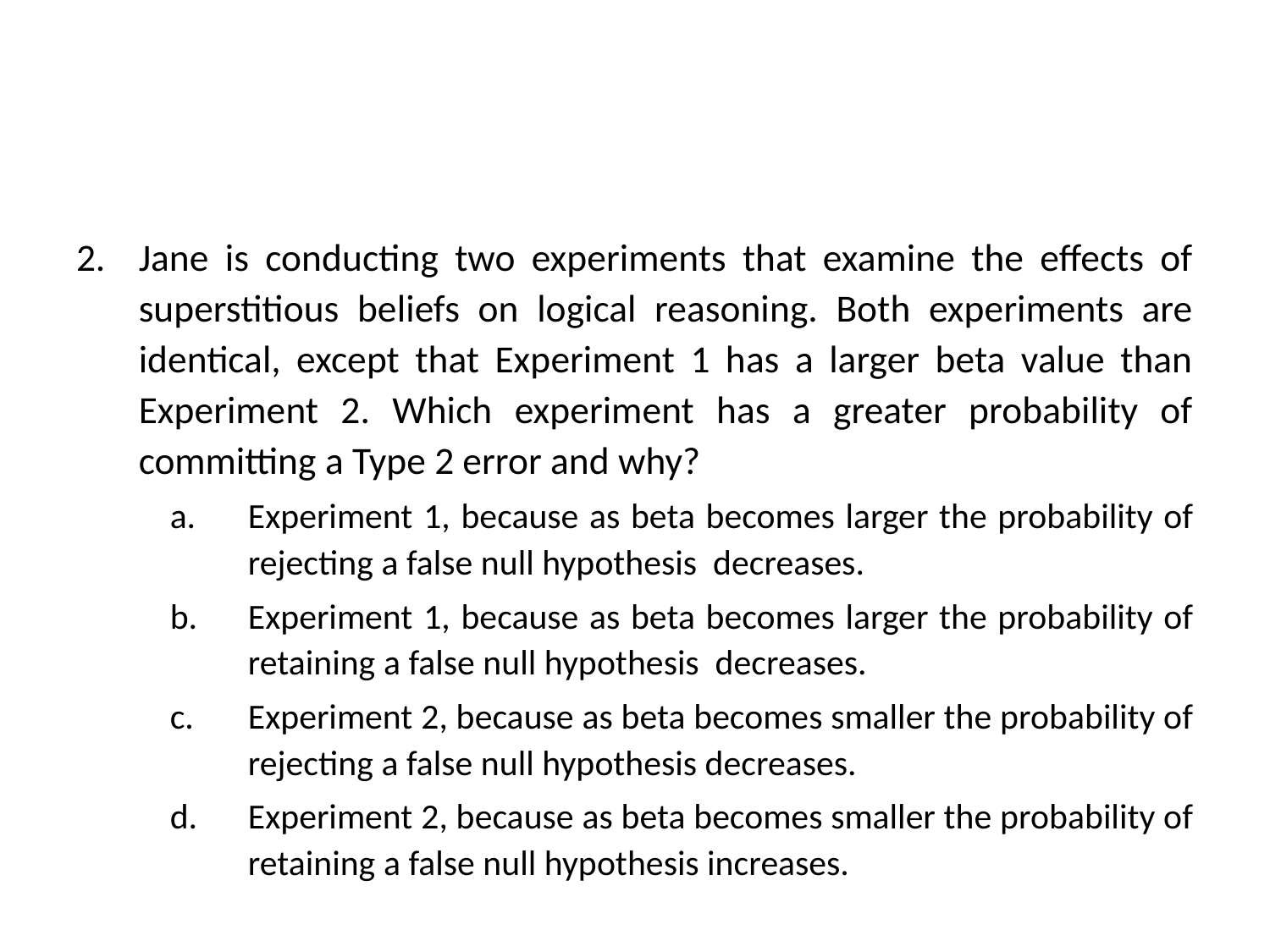

#
Jane is conducting two experiments that examine the effects of superstitious beliefs on logical reasoning. Both experiments are identical, except that Experiment 1 has a larger beta value than Experiment 2. Which experiment has a greater probability of committing a Type 2 error and why?
Experiment 1, because as beta becomes larger the probability of rejecting a false null hypothesis decreases.
Experiment 1, because as beta becomes larger the probability of retaining a false null hypothesis decreases.
Experiment 2, because as beta becomes smaller the probability of rejecting a false null hypothesis decreases.
Experiment 2, because as beta becomes smaller the probability of retaining a false null hypothesis increases.

## Slide 76
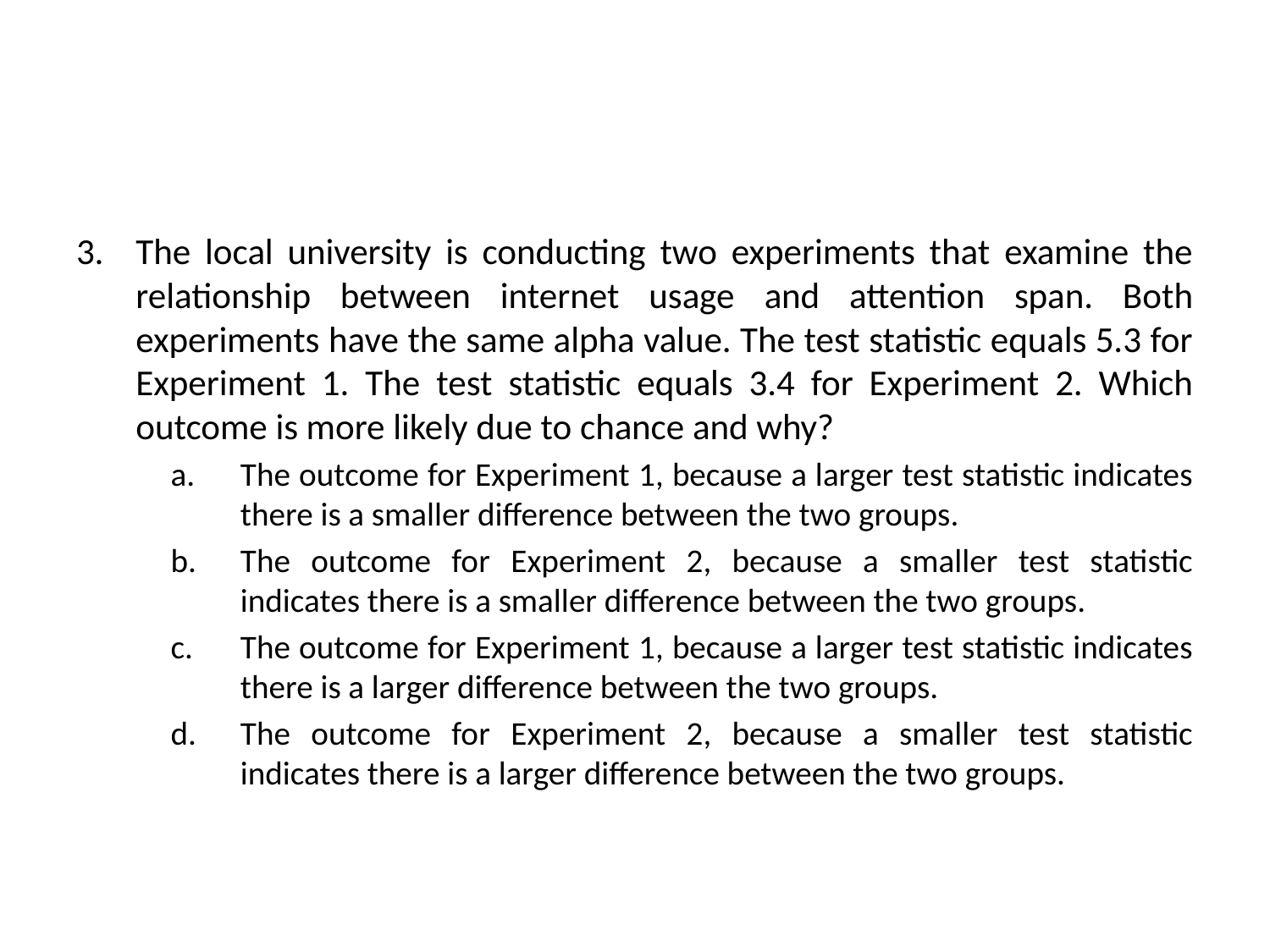

#
The local university is conducting two experiments that examine the relationship between internet usage and attention span. Both experiments have the same alpha value. The test statistic equals 5.3 for Experiment 1. The test statistic equals 3.4 for Experiment 2. Which outcome is more likely due to chance and why?
The outcome for Experiment 1, because a larger test statistic indicates there is a smaller difference between the two groups.
The outcome for Experiment 2, because a smaller test statistic indicates there is a smaller difference between the two groups.
The outcome for Experiment 1, because a larger test statistic indicates there is a larger difference between the two groups.
The outcome for Experiment 2, because a smaller test statistic indicates there is a larger difference between the two groups.

## Slide 77
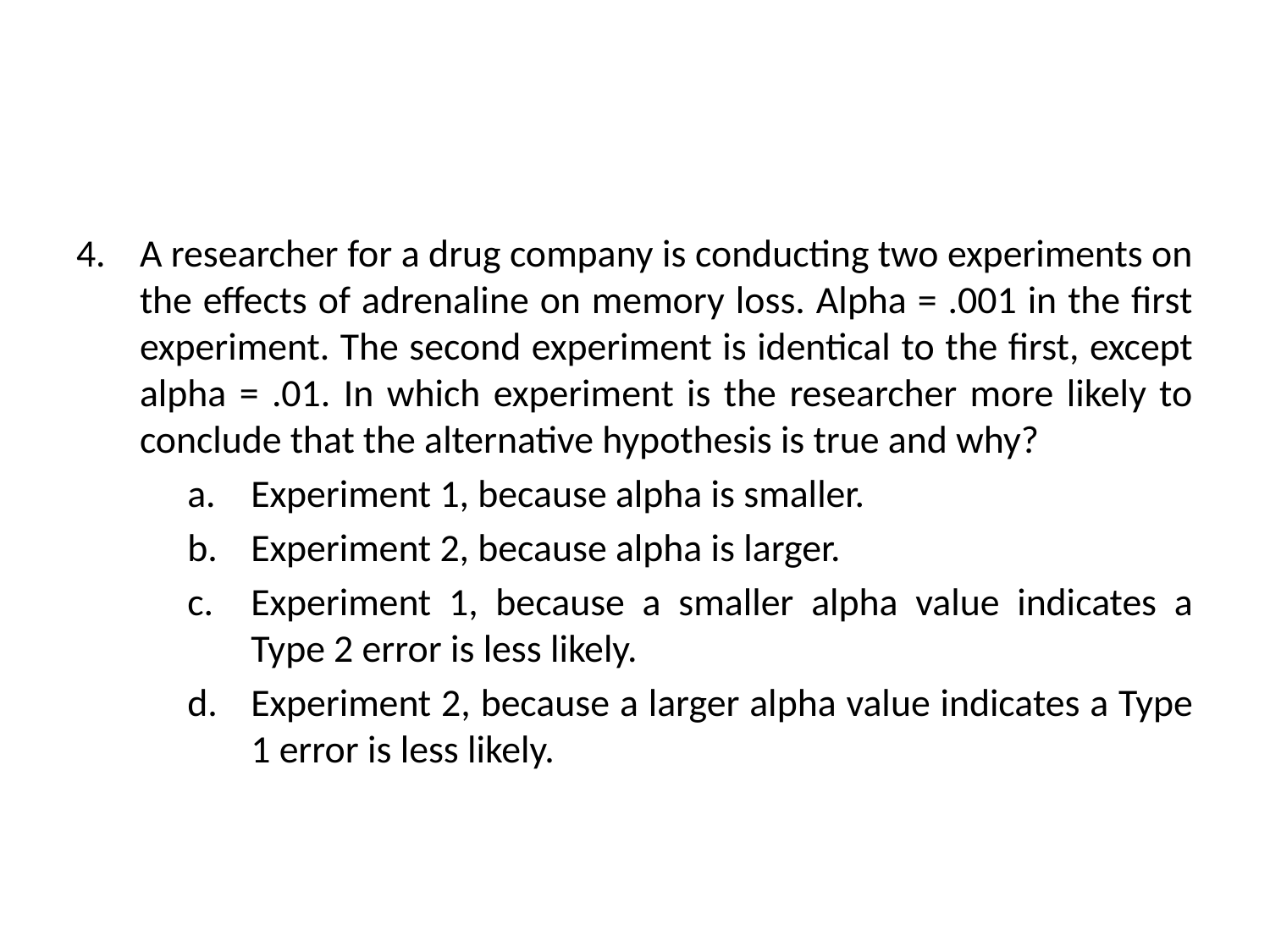

#
A researcher for a drug company is conducting two experiments on the effects of adrenaline on memory loss. Alpha = .001 in the first experiment. The second experiment is identical to the first, except alpha = .01. In which experiment is the researcher more likely to conclude that the alternative hypothesis is true and why?
Experiment 1, because alpha is smaller.
Experiment 2, because alpha is larger.
Experiment 1, because a smaller alpha value indicates a Type 2 error is less likely.
Experiment 2, because a larger alpha value indicates a Type 1 error is less likely.

## Slide 78
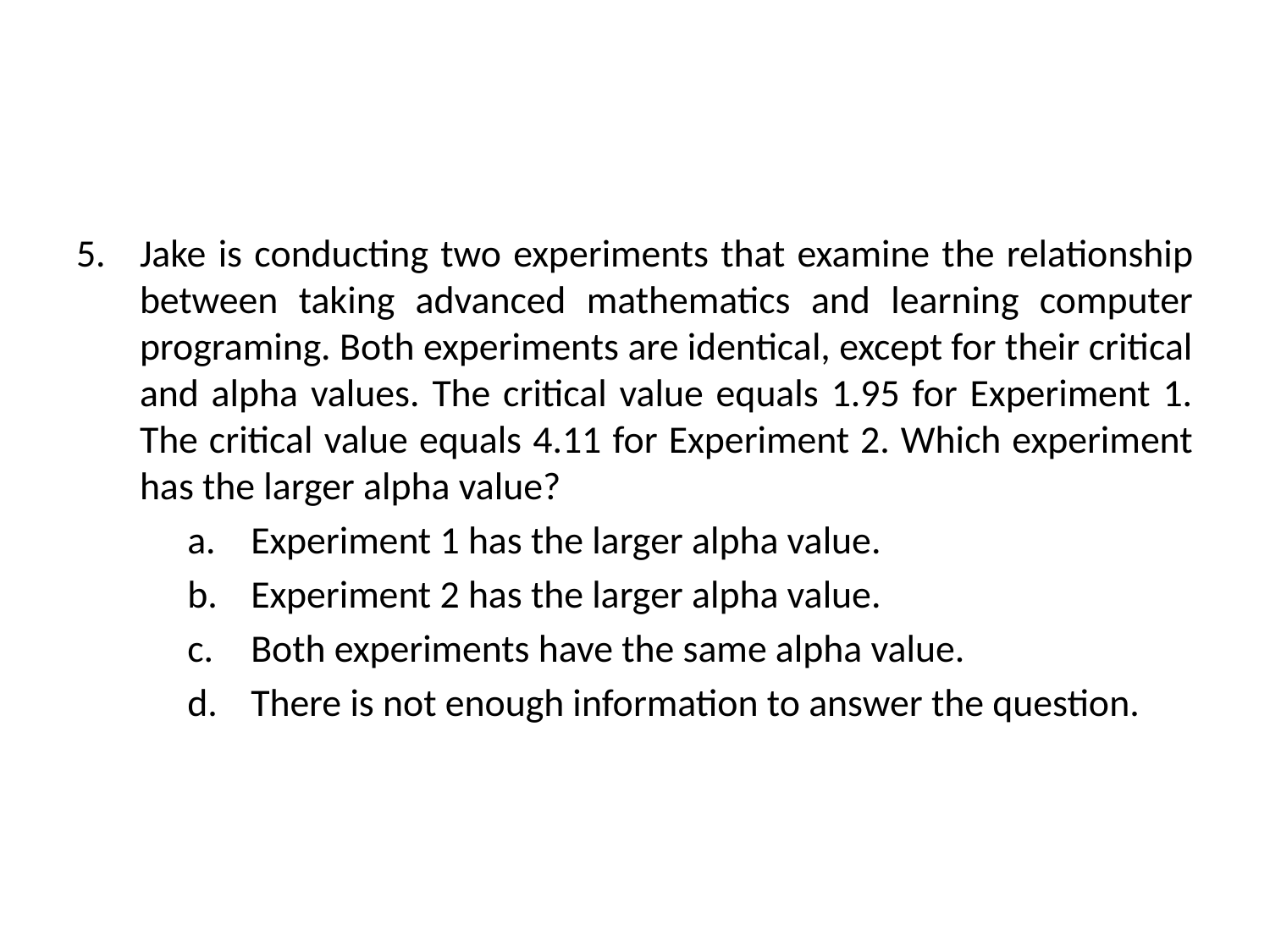

#
Jake is conducting two experiments that examine the relationship between taking advanced mathematics and learning computer programing. Both experiments are identical, except for their critical and alpha values. The critical value equals 1.95 for Experiment 1. The critical value equals 4.11 for Experiment 2. Which experiment has the larger alpha value?
Experiment 1 has the larger alpha value.
Experiment 2 has the larger alpha value.
Both experiments have the same alpha value.
There is not enough information to answer the question.

## Slide 79
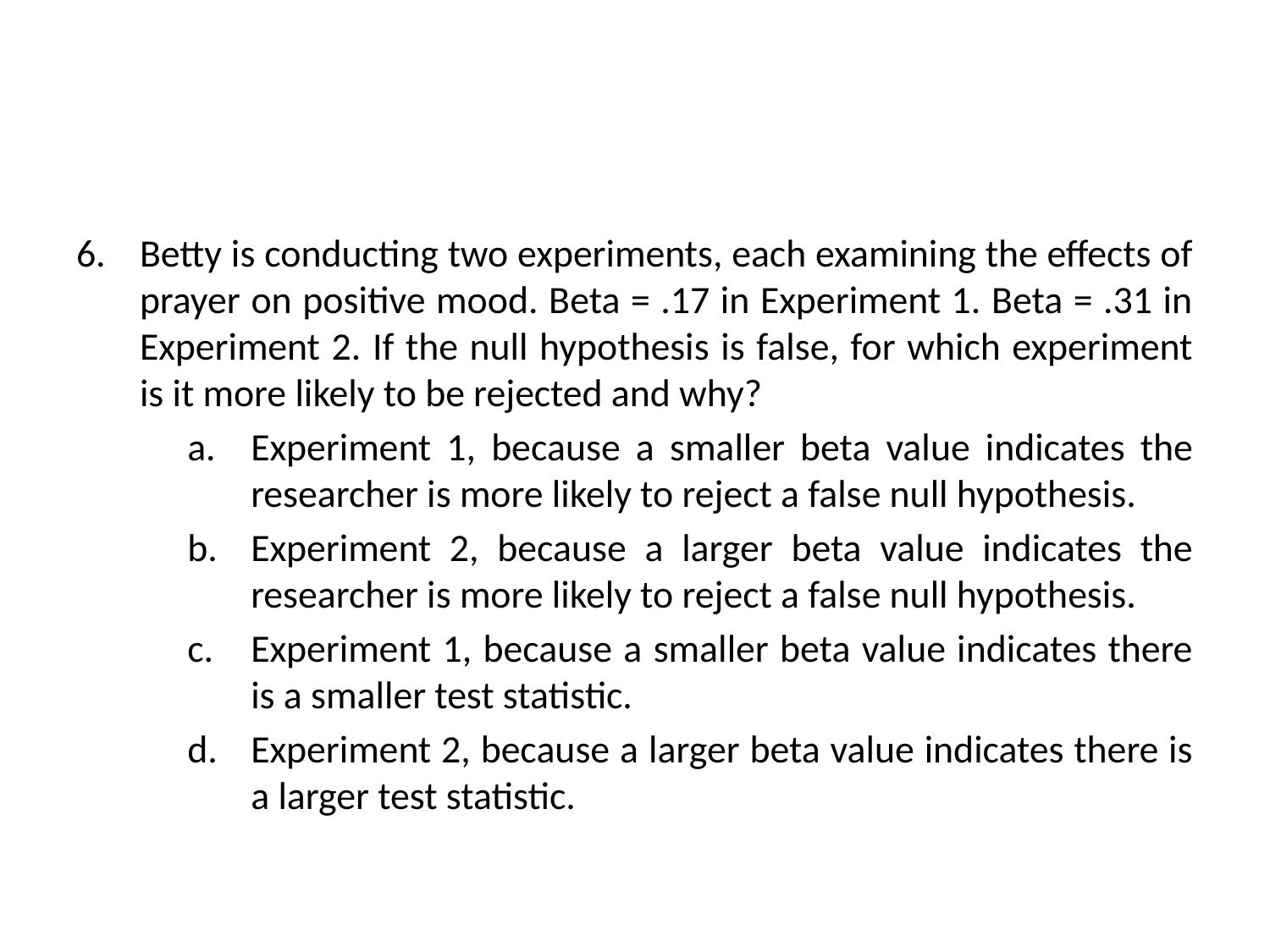

#
Betty is conducting two experiments, each examining the effects of prayer on positive mood. Beta = .17 in Experiment 1. Beta = .31 in Experiment 2. If the null hypothesis is false, for which experiment is it more likely to be rejected and why?
Experiment 1, because a smaller beta value indicates the researcher is more likely to reject a false null hypothesis.
Experiment 2, because a larger beta value indicates the researcher is more likely to reject a false null hypothesis.
Experiment 1, because a smaller beta value indicates there is a smaller test statistic.
Experiment 2, because a larger beta value indicates there is a larger test statistic.

## Slide 80
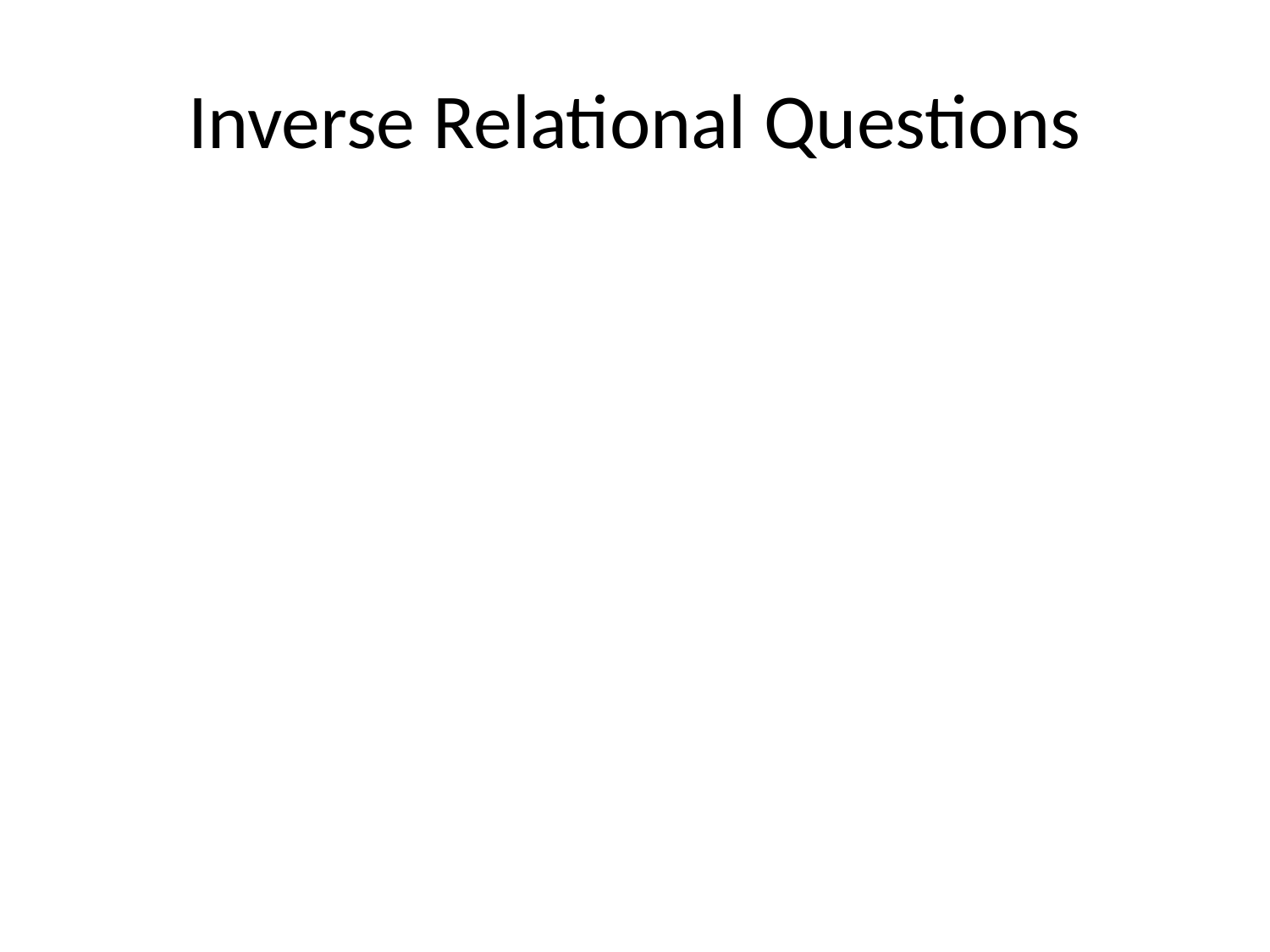

# Inverse Relational Questions

## Slide 81
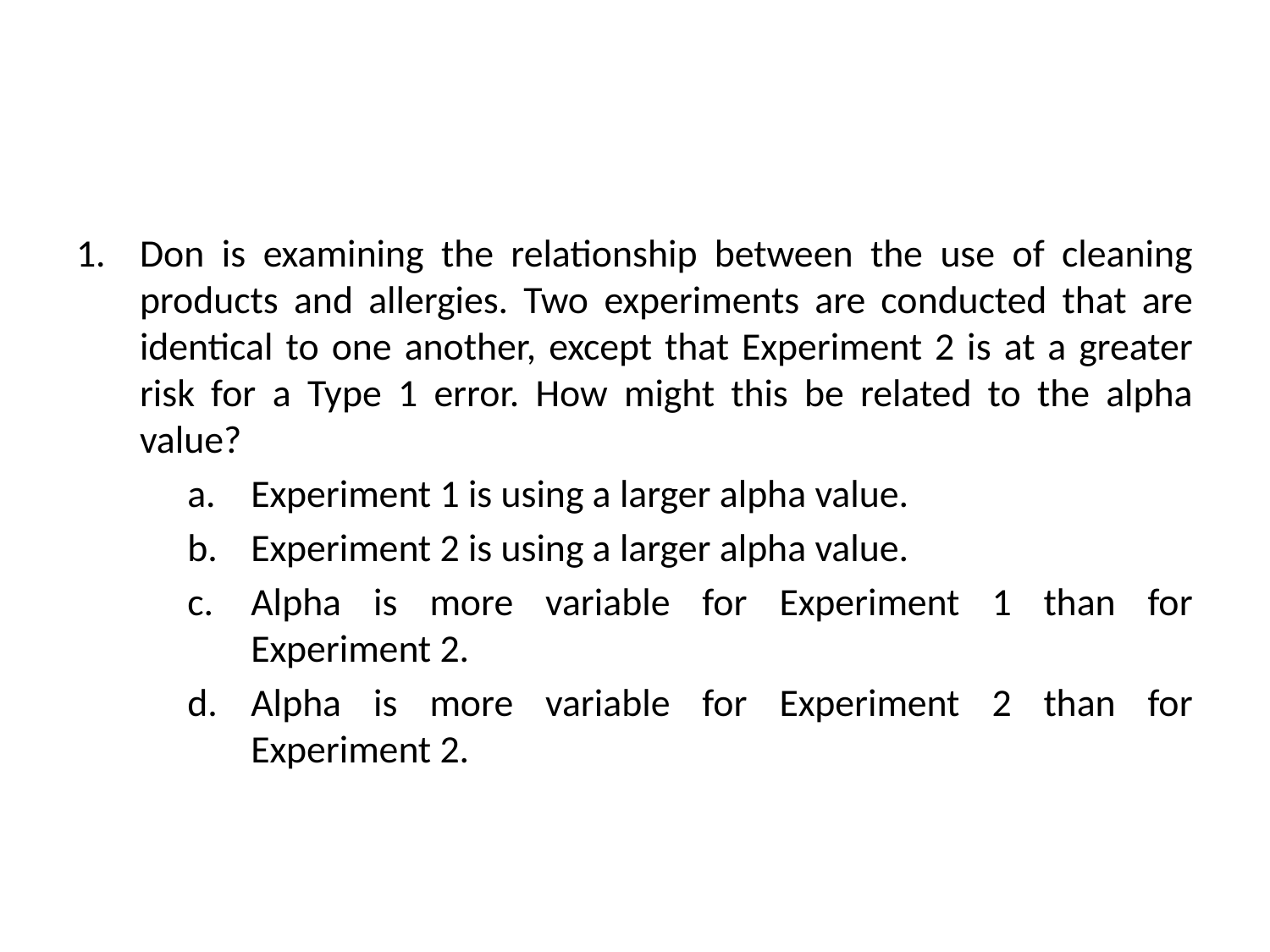

#
Don is examining the relationship between the use of cleaning products and allergies. Two experiments are conducted that are identical to one another, except that Experiment 2 is at a greater risk for a Type 1 error. How might this be related to the alpha value?
Experiment 1 is using a larger alpha value.
Experiment 2 is using a larger alpha value.
Alpha is more variable for Experiment 1 than for Experiment 2.
Alpha is more variable for Experiment 2 than for Experiment 2.

## Slide 82
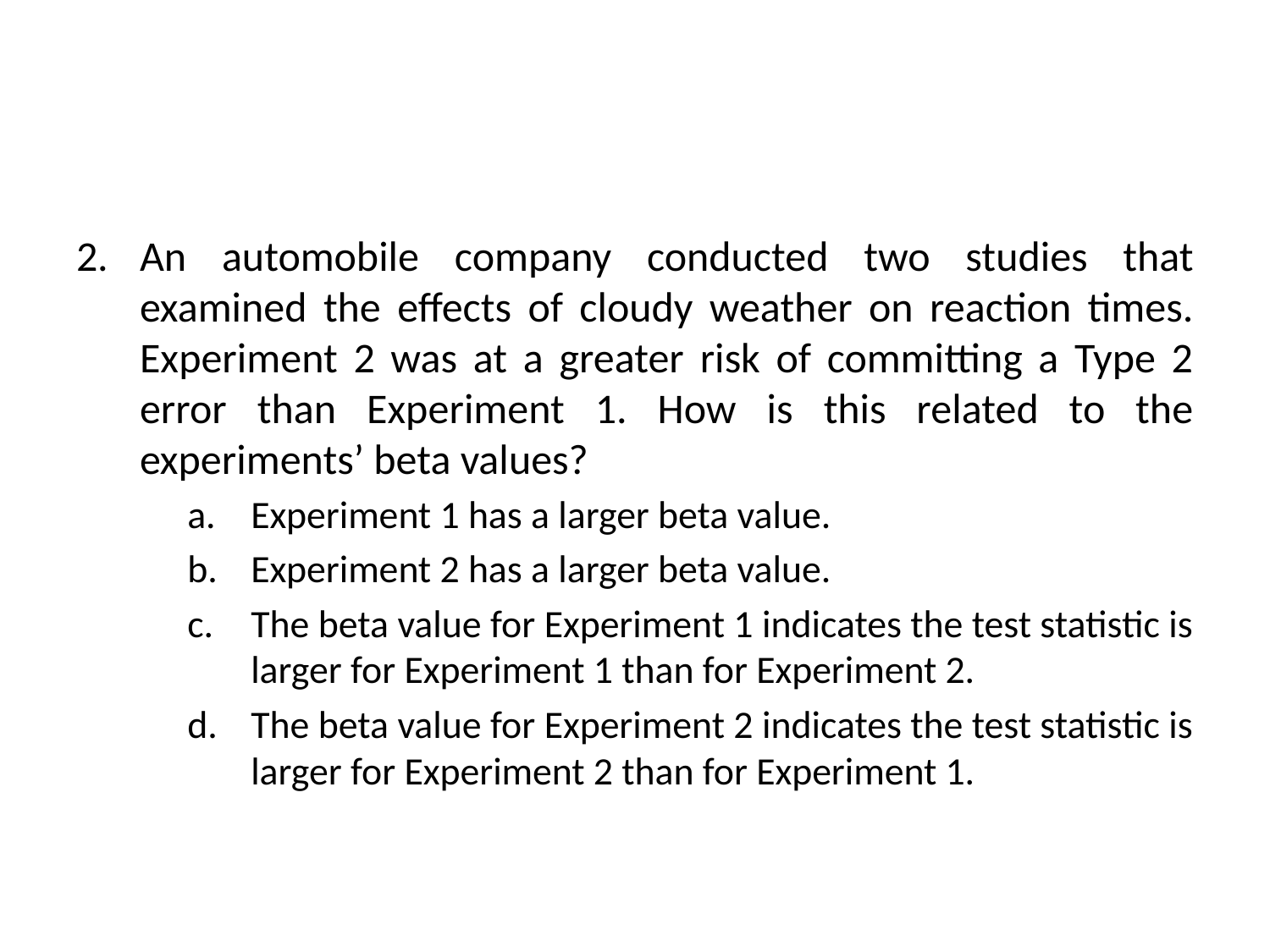

#
An automobile company conducted two studies that examined the effects of cloudy weather on reaction times. Experiment 2 was at a greater risk of committing a Type 2 error than Experiment 1. How is this related to the experiments’ beta values?
Experiment 1 has a larger beta value.
Experiment 2 has a larger beta value.
The beta value for Experiment 1 indicates the test statistic is larger for Experiment 1 than for Experiment 2.
The beta value for Experiment 2 indicates the test statistic is larger for Experiment 2 than for Experiment 1.

## Slide 83
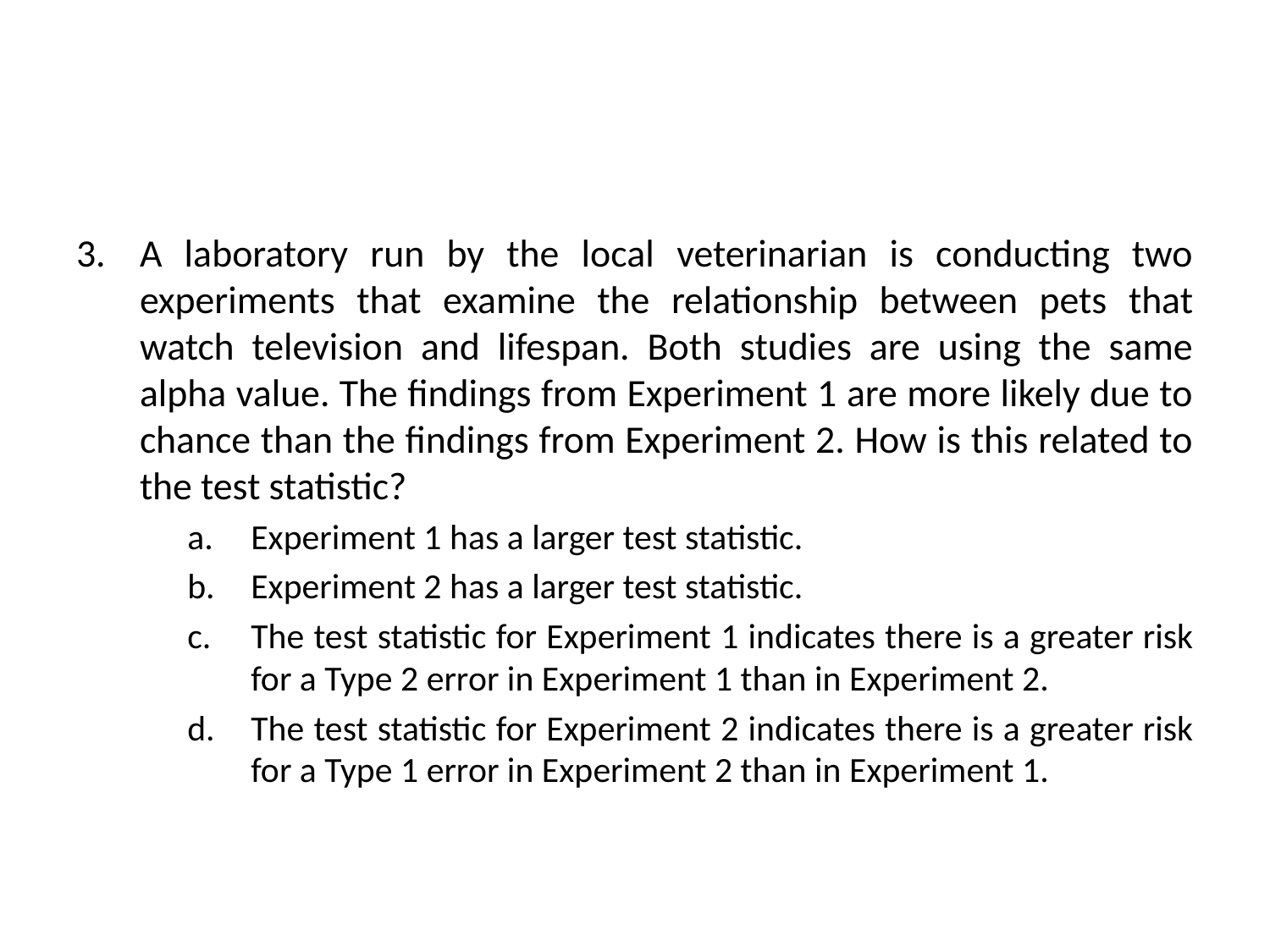

#
A laboratory run by the local veterinarian is conducting two experiments that examine the relationship between pets that watch television and lifespan. Both studies are using the same alpha value. The findings from Experiment 1 are more likely due to chance than the findings from Experiment 2. How is this related to the test statistic?
Experiment 1 has a larger test statistic.
Experiment 2 has a larger test statistic.
The test statistic for Experiment 1 indicates there is a greater risk for a Type 2 error in Experiment 1 than in Experiment 2.
The test statistic for Experiment 2 indicates there is a greater risk for a Type 1 error in Experiment 2 than in Experiment 1.

## Slide 84
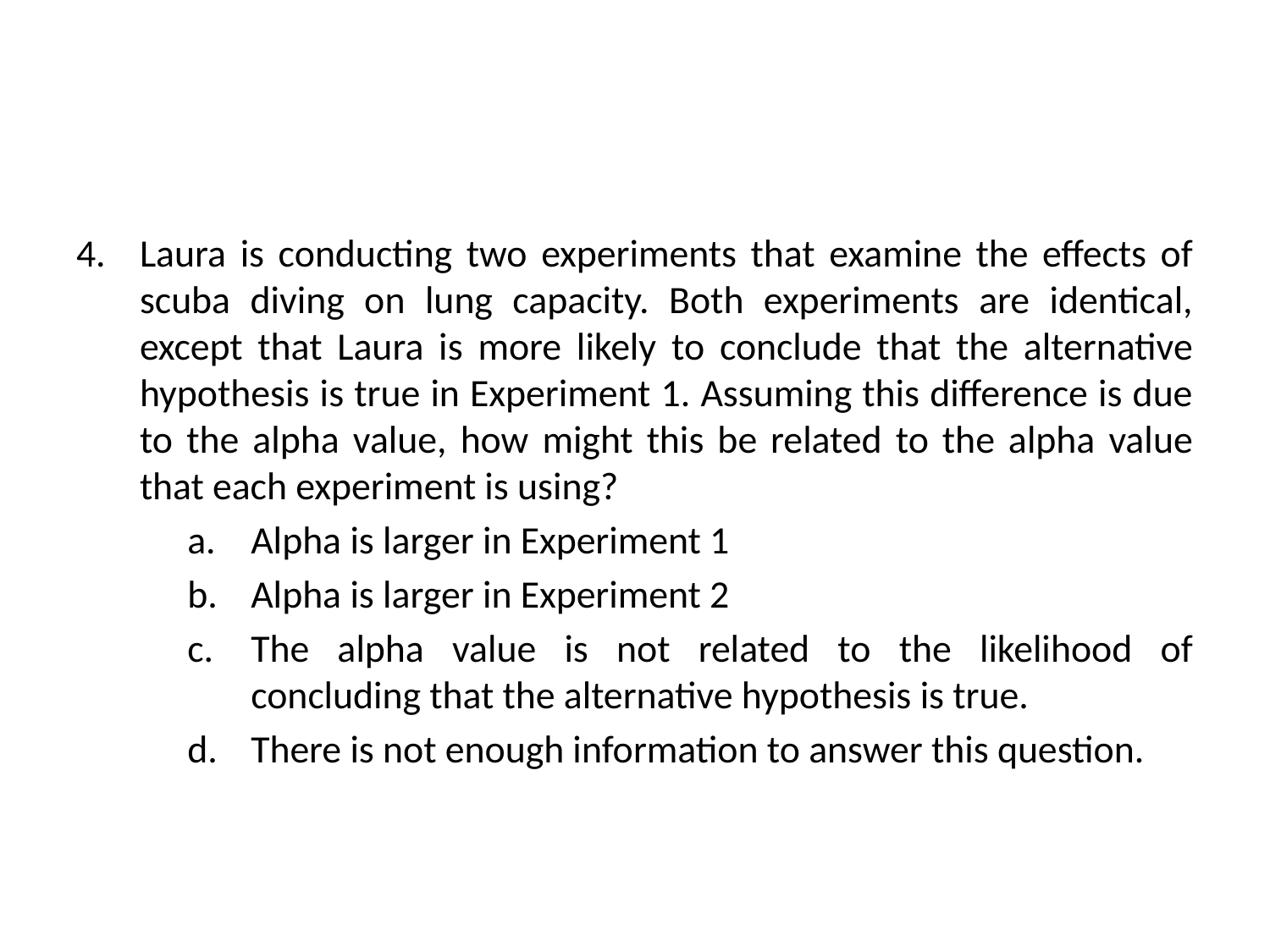

#
Laura is conducting two experiments that examine the effects of scuba diving on lung capacity. Both experiments are identical, except that Laura is more likely to conclude that the alternative hypothesis is true in Experiment 1. Assuming this difference is due to the alpha value, how might this be related to the alpha value that each experiment is using?
Alpha is larger in Experiment 1
Alpha is larger in Experiment 2
The alpha value is not related to the likelihood of concluding that the alternative hypothesis is true.
There is not enough information to answer this question.

## Slide 85
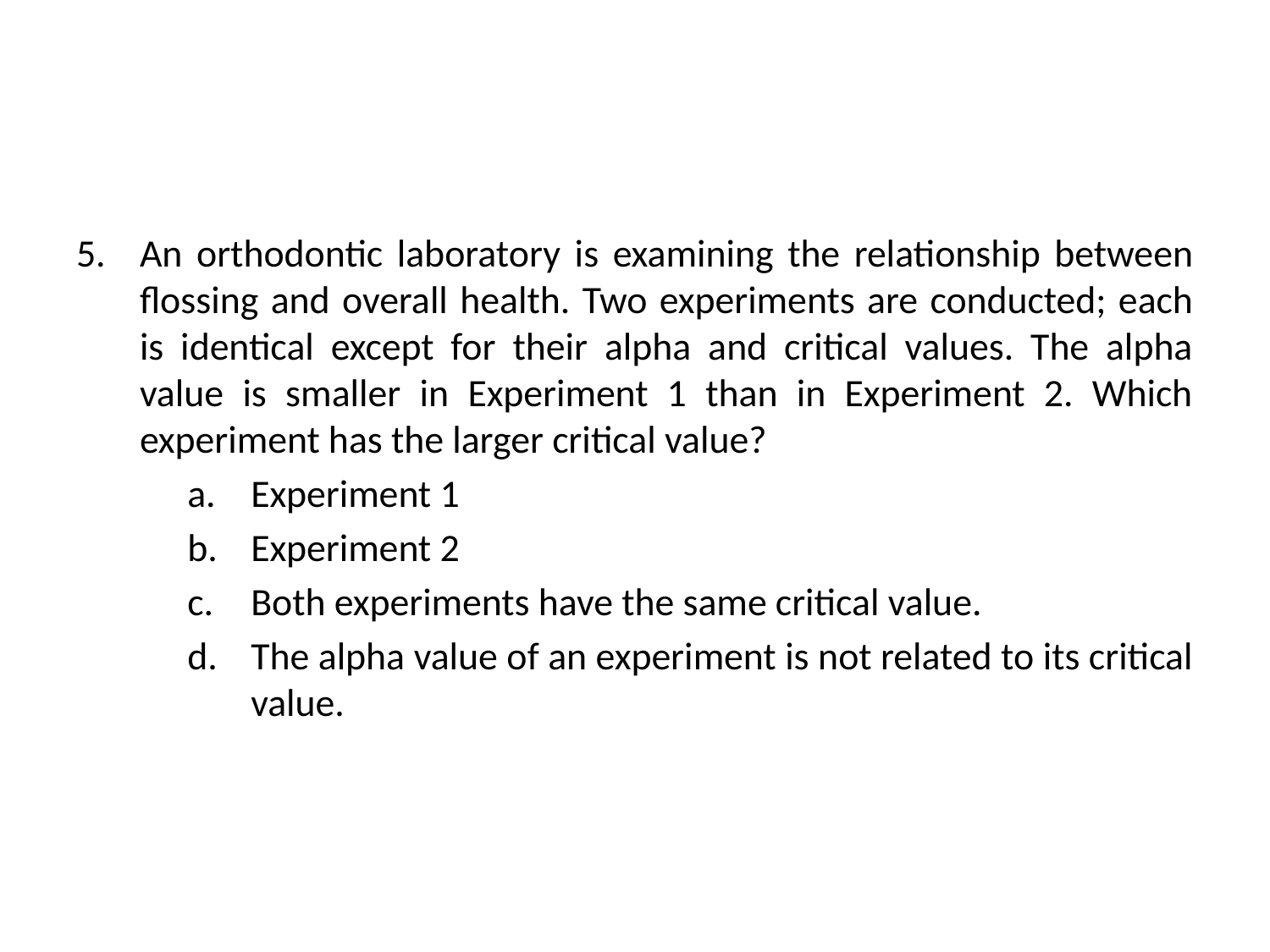

#
An orthodontic laboratory is examining the relationship between flossing and overall health. Two experiments are conducted; each is identical except for their alpha and critical values. The alpha value is smaller in Experiment 1 than in Experiment 2. Which experiment has the larger critical value?
Experiment 1
Experiment 2
Both experiments have the same critical value.
The alpha value of an experiment is not related to its critical value.

## Slide 86
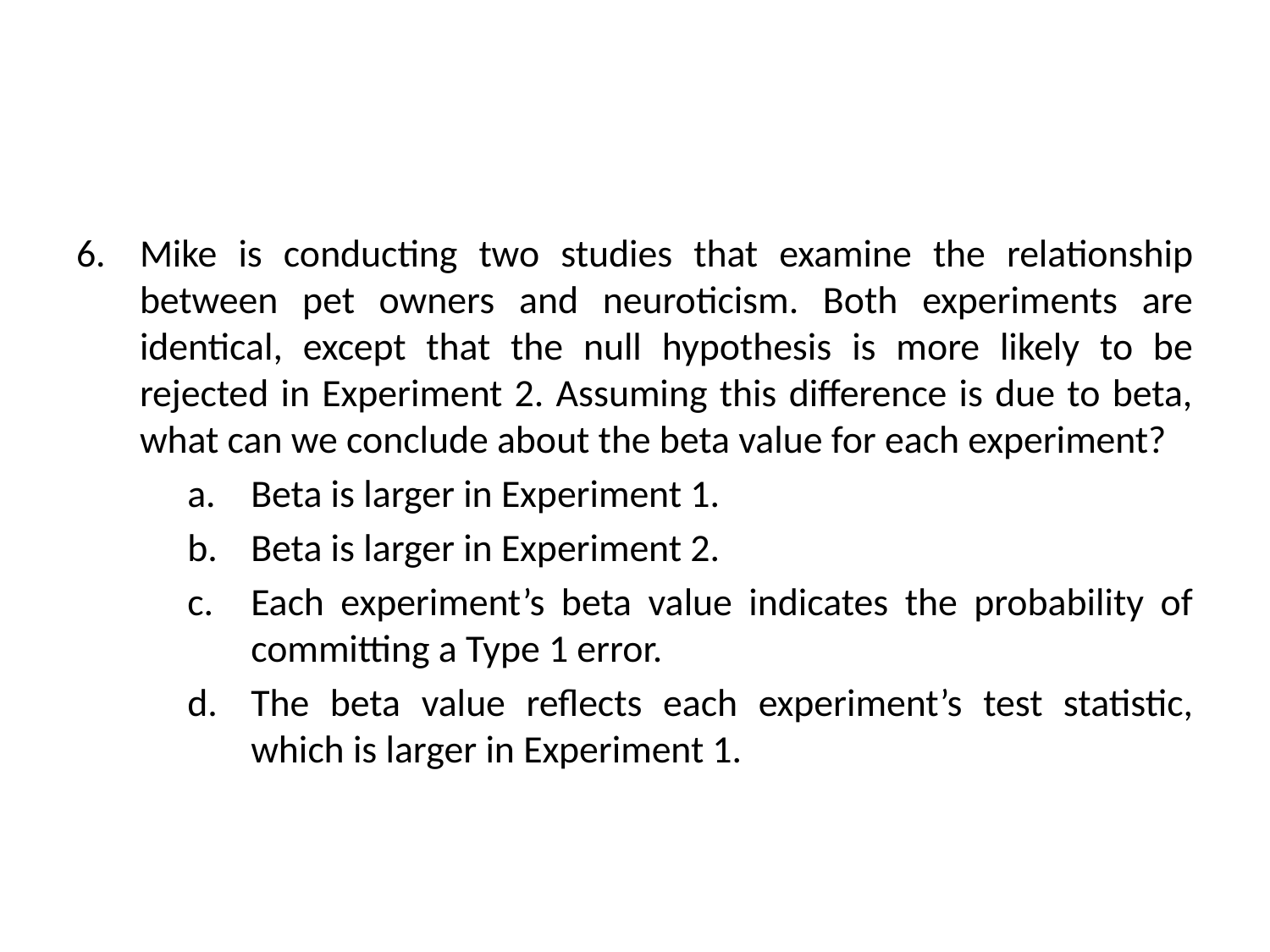

#
Mike is conducting two studies that examine the relationship between pet owners and neuroticism. Both experiments are identical, except that the null hypothesis is more likely to be rejected in Experiment 2. Assuming this difference is due to beta, what can we conclude about the beta value for each experiment?
Beta is larger in Experiment 1.
Beta is larger in Experiment 2.
Each experiment’s beta value indicates the probability of committing a Type 1 error.
The beta value reflects each experiment’s test statistic, which is larger in Experiment 1.

## Slide 87
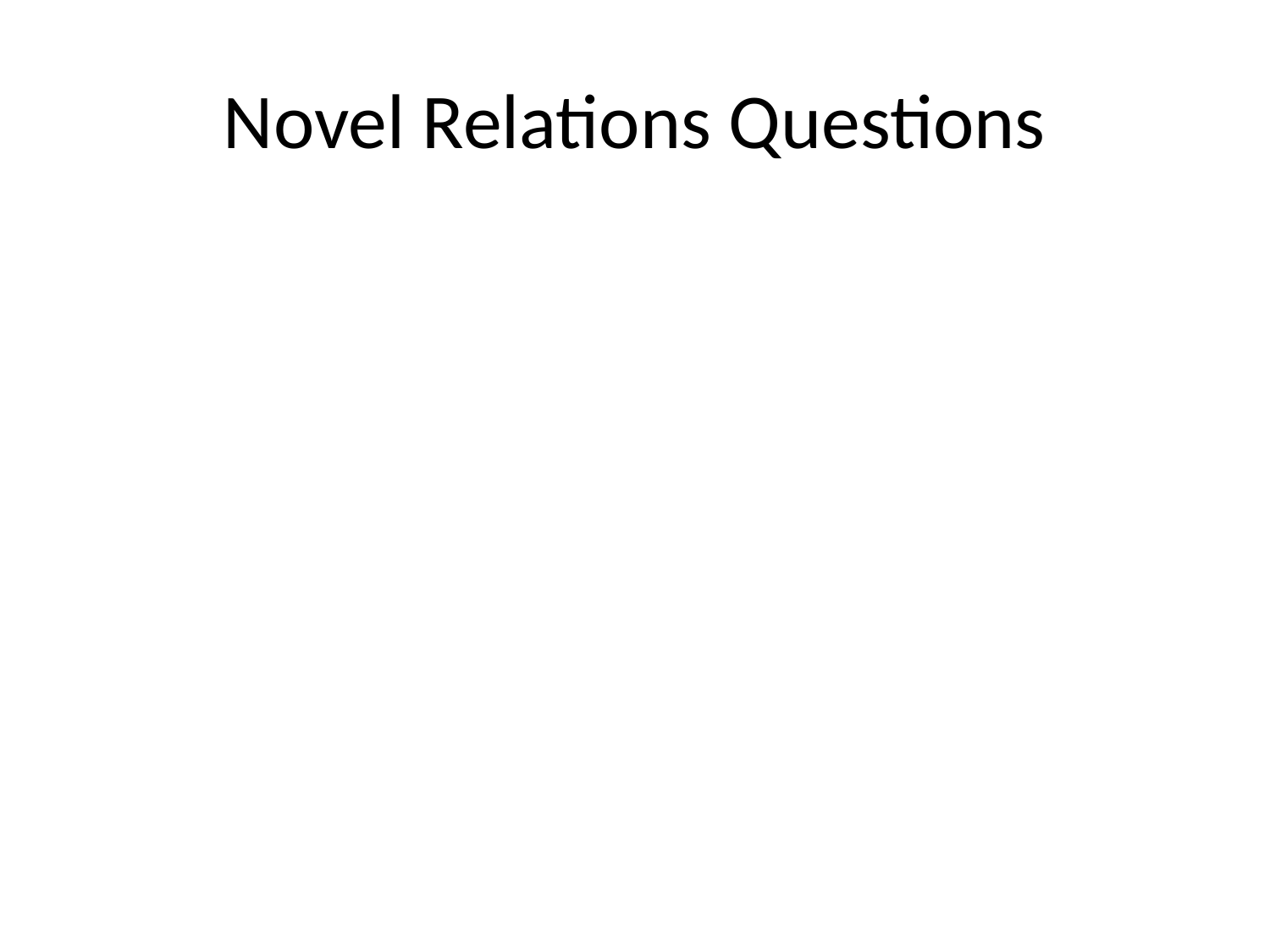

# Novel Relations Questions

## Slide 88
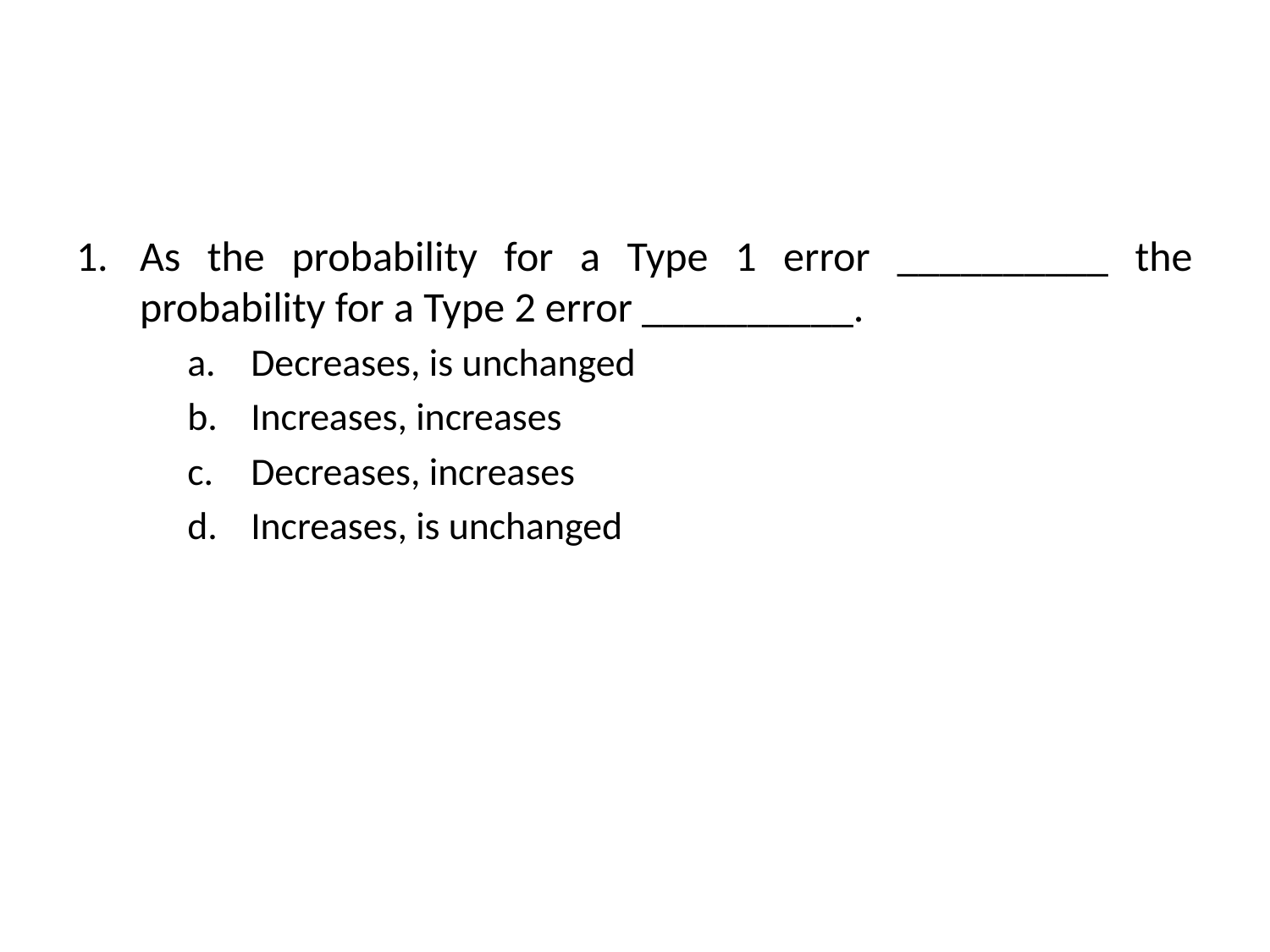

#
As the probability for a Type 1 error __________ the probability for a Type 2 error __________.
Decreases, is unchanged
Increases, increases
Decreases, increases
Increases, is unchanged

## Slide 89
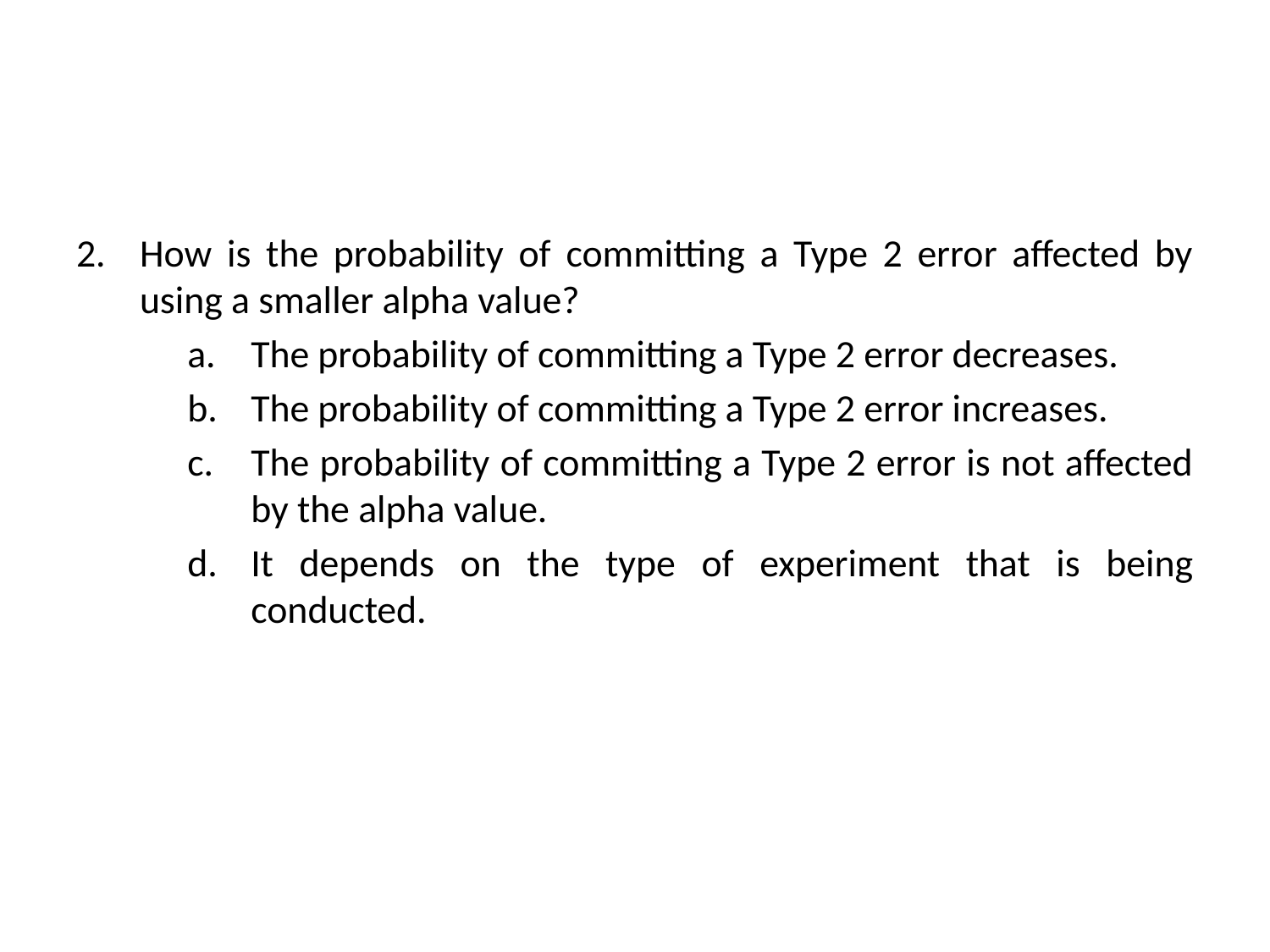

#
How is the probability of committing a Type 2 error affected by using a smaller alpha value?
The probability of committing a Type 2 error decreases.
The probability of committing a Type 2 error increases.
The probability of committing a Type 2 error is not affected by the alpha value.
It depends on the type of experiment that is being conducted.

## Slide 90
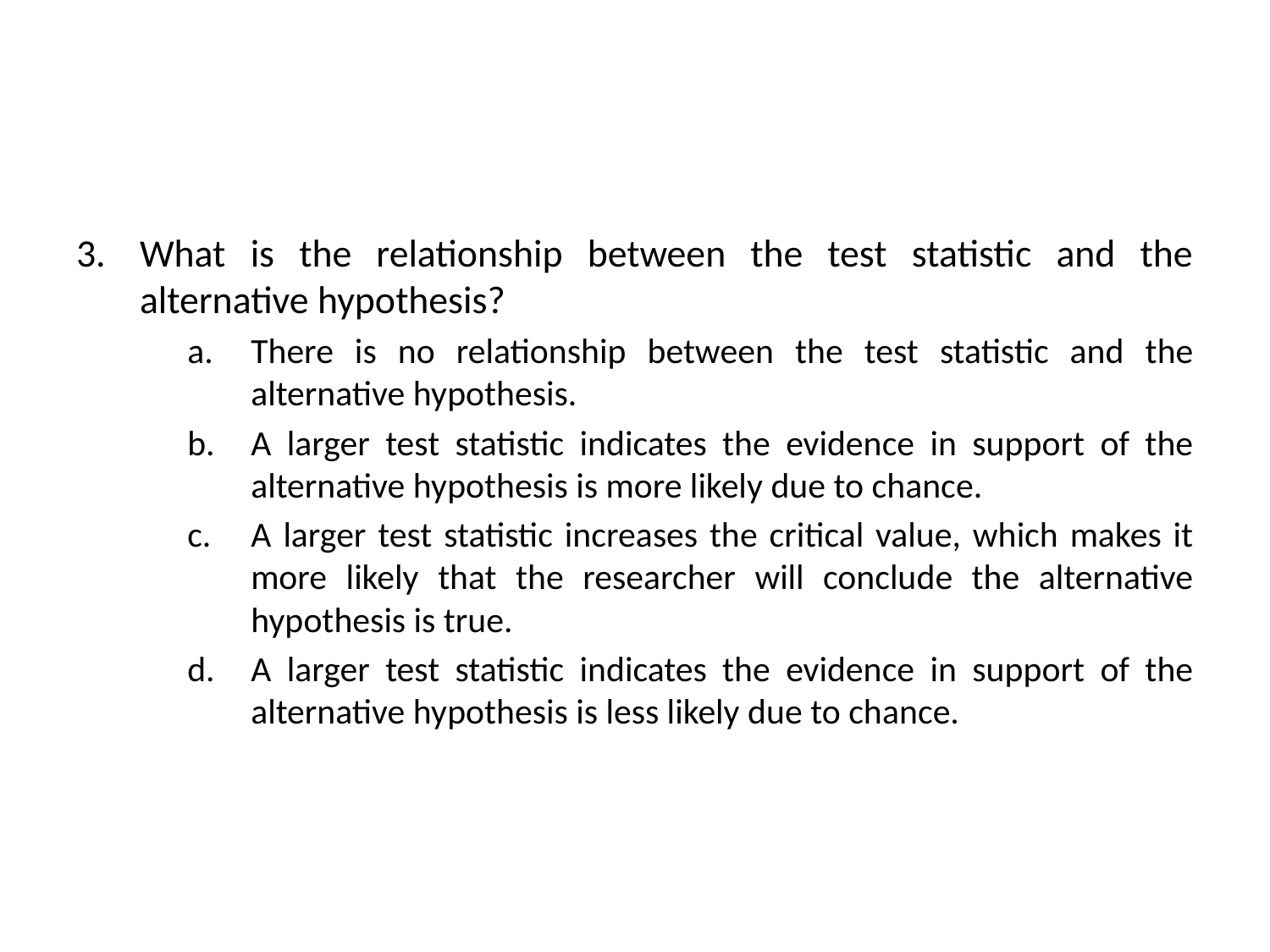

#
What is the relationship between the test statistic and the alternative hypothesis?
There is no relationship between the test statistic and the alternative hypothesis.
A larger test statistic indicates the evidence in support of the alternative hypothesis is more likely due to chance.
A larger test statistic increases the critical value, which makes it more likely that the researcher will conclude the alternative hypothesis is true.
A larger test statistic indicates the evidence in support of the alternative hypothesis is less likely due to chance.

## Slide 91
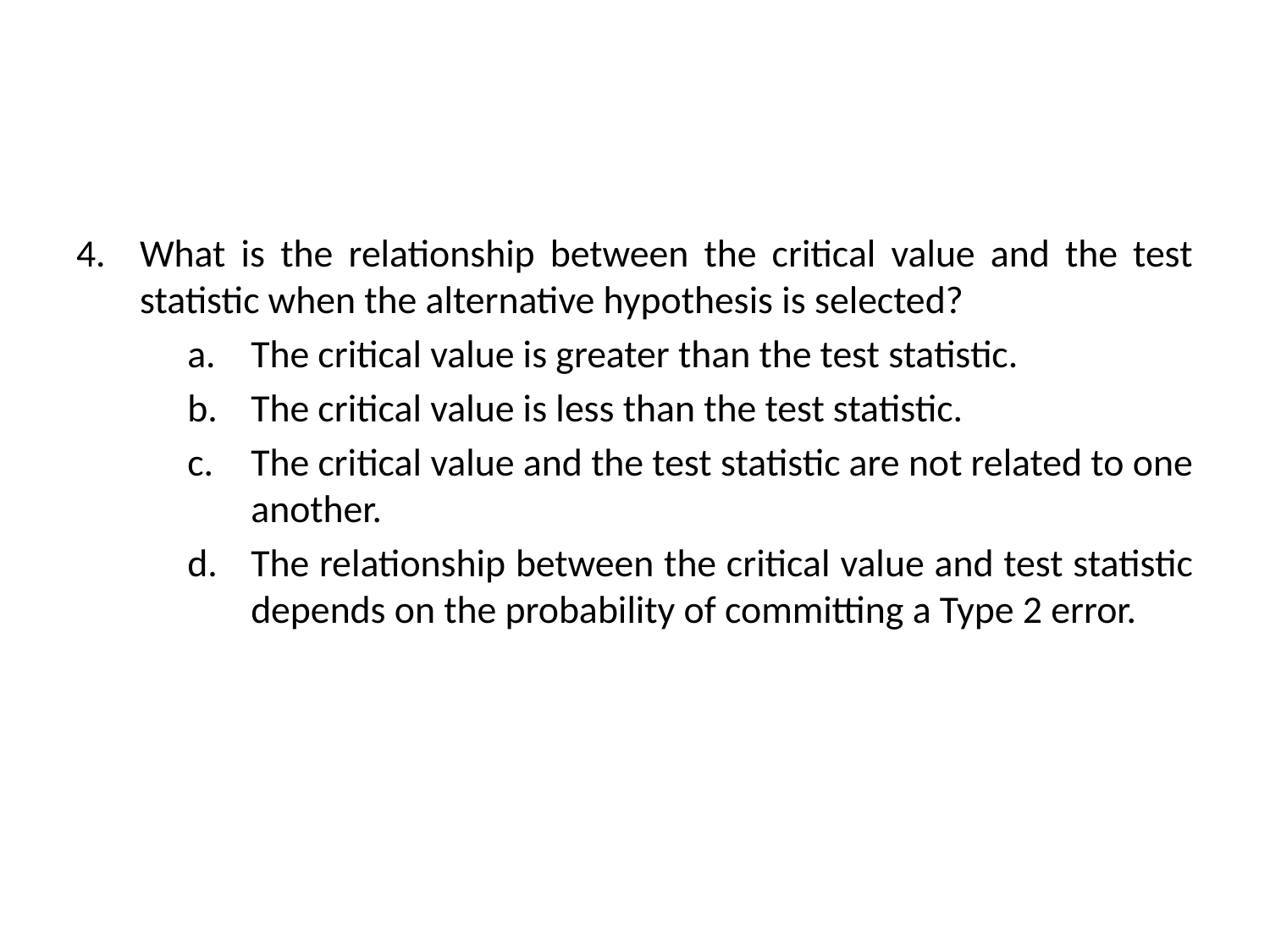

#
What is the relationship between the critical value and the test statistic when the alternative hypothesis is selected?
The critical value is greater than the test statistic.
The critical value is less than the test statistic.
The critical value and the test statistic are not related to one another.
The relationship between the critical value and test statistic depends on the probability of committing a Type 2 error.

## Slide 92
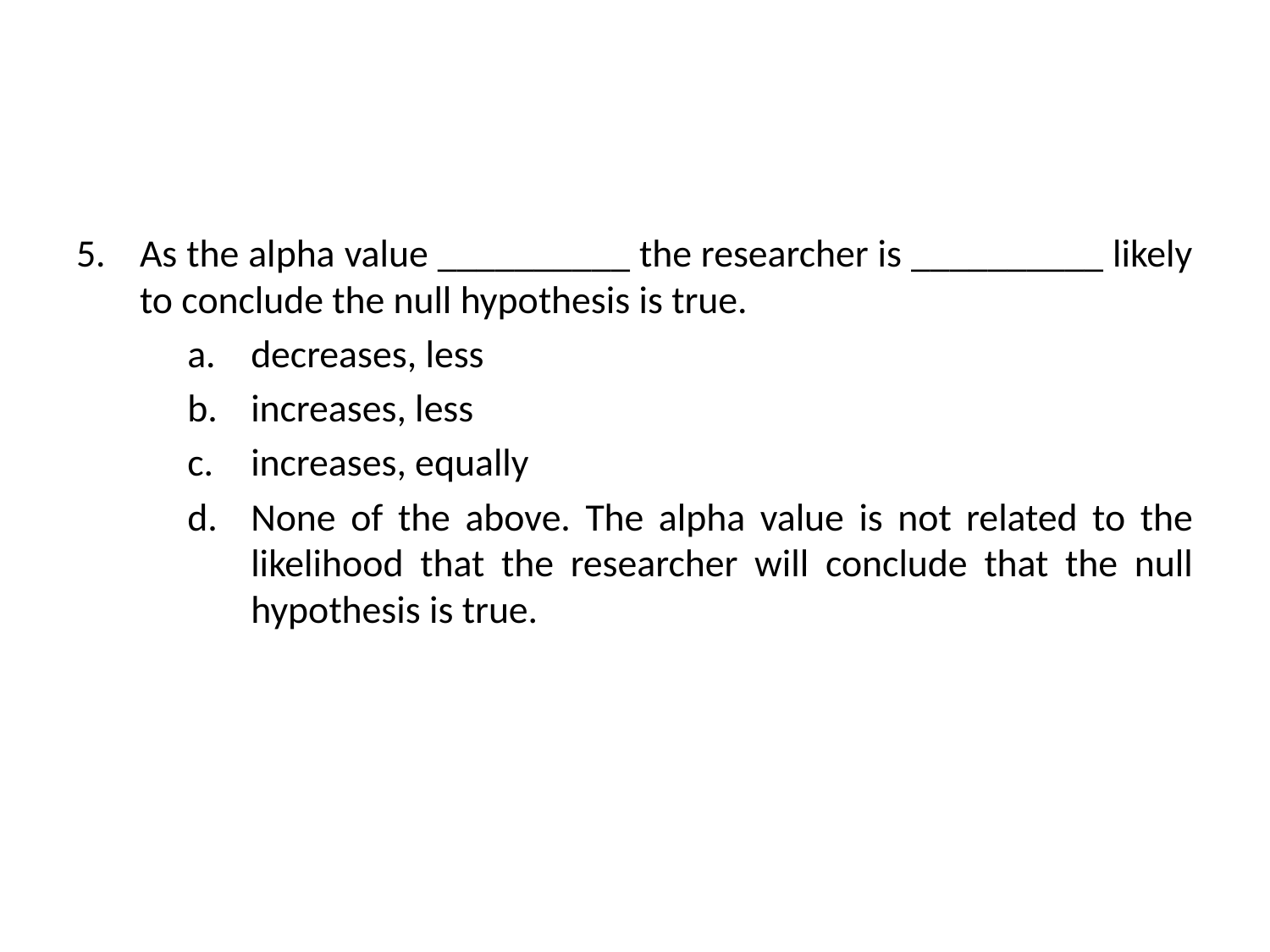

#
As the alpha value __________ the researcher is __________ likely to conclude the null hypothesis is true.
decreases, less
increases, less
increases, equally
None of the above. The alpha value is not related to the likelihood that the researcher will conclude that the null hypothesis is true.

## Slide 93
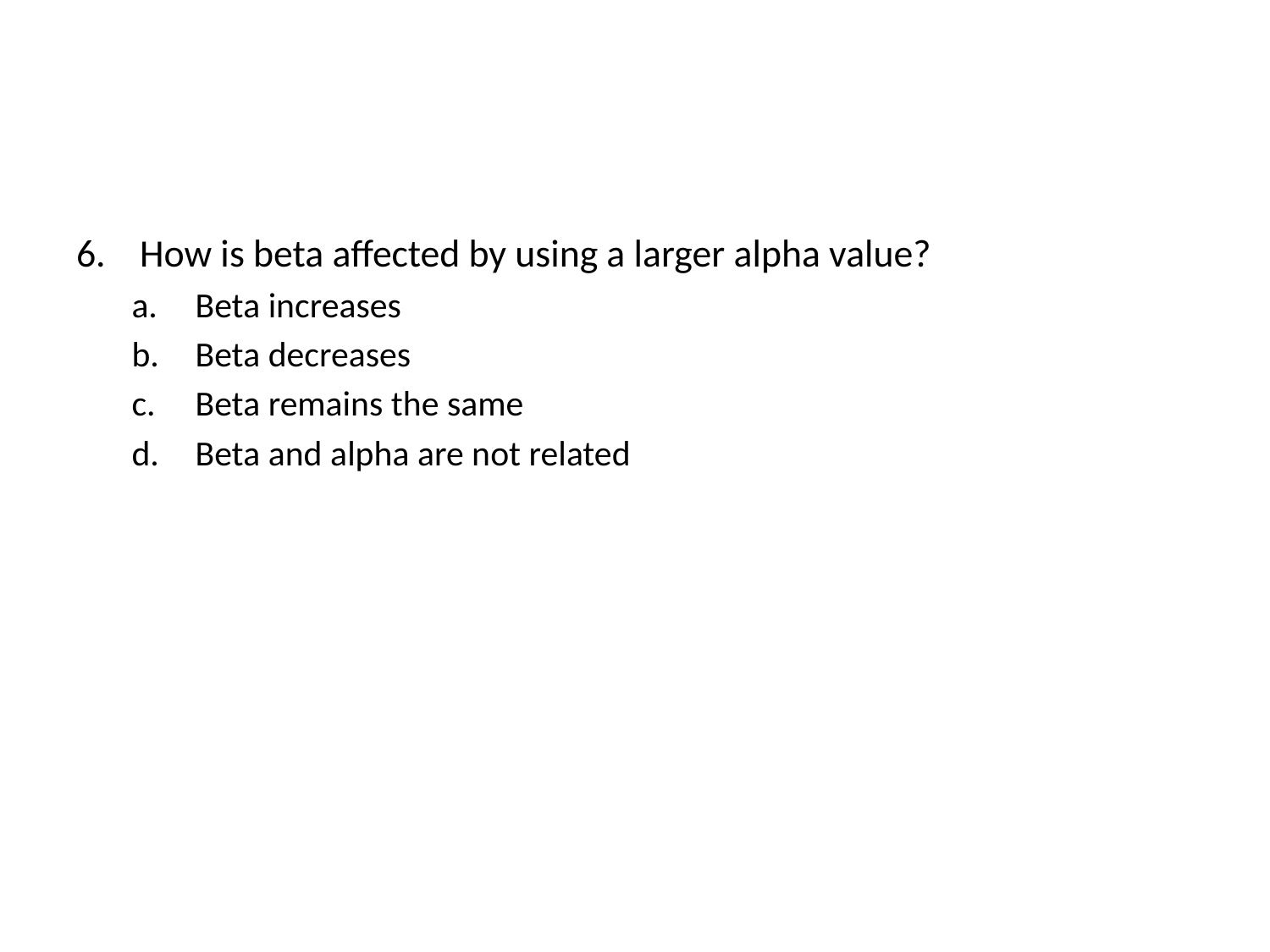

#
How is beta affected by using a larger alpha value?
Beta increases
Beta decreases
Beta remains the same
Beta and alpha are not related
